# A revised cranial description of *Massospondylus carinatus* Owen (Dinosauria: Sauropodomorpha) based on computed tomographic scans and a review of cranial characters for basal Sauropodomorpha

**DOI:** 10.7717/peerj.4224

**Published:** 2018-01-12

**Authors:** Kimberley E.J. Chapelle, Jonah N. Choiniere

**Affiliations:** Evolutionary Studies Institute and School of Geosciences, University of the Witwatersrand, Johannesburg, South Africa

**Keywords:** *Massospondylus*, Sauropodomorph, Computed tomography, Skull, Braincase, Phylogeny, Matrix, Characters, South Africa, Taxonomy

## Abstract

*Massospondylus carinatus* is a basal sauropodomorph dinosaur from the early Jurassic Elliot Formation of South Africa. It is one of the best-represented fossil dinosaur taxa, known from hundreds of specimens including at least 13 complete or nearly complete skulls. Surprisingly, the internal cranial anatomy of *M. carinatus* has never been described using computed tomography (CT) methods. Using CT scans and 3D digital representations, we digitally reconstruct the bones of the facial skeleton, braincase, and palate of a complete, undistorted cranium of *M. carinatus* (BP/1/5241). We describe the anatomical features of the cranial bones, and compare them to other closely related sauropodomorph taxa such as *Plateosaurus erlenbergiensis*, *Lufengosaurus huenei*, *Sarahsaurus aurifontanalis* and *Efraasia minor*. We identify a suite of character states of the skull and braincase for *M. carinatus* that sets it apart from other taxa, but these remain tentative due to the lack of comparative sauropodomorph braincase descriptions in the literature. Furthermore, we hypothesize 27 new cranial characters useful for determining relationships in non-sauropodan Sauropodomorpha, delete five pre-existing characters and revise the scores of several existing cranial characters to make more explicit homology statements. All the characters that we hypothesized or revised are illustrated. Using parsimony as an optimality criterion, we then test the relationships of *M. carinatus* (using BP/1/5241 as a specimen-level exemplar) in our revised phylogenetic data matrix.

## Introduction

*Massospondylus carinatus* Owen, 1854 is a basal non-sauropodan sauropodomorph dinosaur from the Early Jurassic, found in the upper Elliot to lower Clarens Formations of South Africa and Lesotho, as well as in comparable formations in Zimbabwe (Bordy & Eriksson, 2015; Cooper, 1981; Haughton, 1924; Kitching & Raath, 1984; Knoll, 2005; Olsen & Galton, 1984). It was one of the first dinosaurs ever described and is emblematic of the importance of South African palaeontology to the study of dinosaur evolution (Barrett, 2004; Cooper, 1981; Haughton, 1924; Kitching & Raath, 1984; McPhee et al., 2017; Owen, 1854; Yates & Barrett, 2010). Based on fossil collections records from museums in South Africa, London, Paris, and Zimbabwe, *M. carinatus* was the most abundant dinosaur in the upper Elliot Formation (Barrett, 2004; Gow, 1990; Gow, Kitching & Raath, 1990; Kitching & Raath, 1984; Owen, 1854; Reisz et al., 2005; Yates & Barrett, 2010).

Notable specimens of *M. carinatus* include the largest-known and neotype specimen BP/1/4934 that consists of a skull and near-complete postcranial skeleton, and a cluster of eggs with fully articulated embryos (BP/1/5347A) collected from the upper Elliot Formation in Golden Gate Highlands National Park in South Africa (Kitching, 1979; Reisz et al., 2005; Yates & Barrett, 2010). There are also at least 13 complete or near complete skulls referred to *M. carinatus* in collections around the world. These specimens, as well as other intermediate-sized *M. carinatus* fossils, have allowed researchers to make ontogenetic comparisons as well as comparisons to closely related taxa such as *Plateosaurus* (Gow, 1990; Prieto-Márquez & Norell, 2011; Reisz et al., 2005, 2010; Sues et al., 2004).

Despite the abundance of cranial material for the taxon, comparative research on *M. carinatus* is restricted by a lack of detailed internal cranial descriptions. Digital reconstructions of the *M. carinatus* endocast and inner ear (BP/1/4779) were figured in Sereno et al. (2007), but no descriptive details were provided. Although *M. carinatus* was first described in 1854, it wasn’t until 2004 that published complete and detailed cranial descriptions became available (Sues et al., 2004). Although attempts have been made to identify diagnostic characters for the taxon, it is not known to which taxonomic level these features are diagnostic when taken individually (Barrett, 2009; Sues et al., 2004; Yates & Barrett, 2010). Surprisingly, there are still no unambiguous cranial autapomorphies that diagnose the species or the genus (Sereno, 1999; Sues et al., 2004; Yates & Barrett, 2010). The only exclusive cranial autapomorphy of *Massospondylus* mentioned in the literature is the greatest transverse width of the skull exceeding the dorsoventral height of the skull by at least 10% (Reisz et al., 2005, 2010; Sereno, 1999; Sues et al., 2004). However, the origin of this character and its original description is uncertain and it is not true of several specimens, including the neotype (BP/1/4934) and the specimen presented in this study (BP/1/5241).

This research aims to produce a 3D representation of the skull and braincase of *M. carinatus*, to describe its cranial anatomy (including internal structures), and to compare these data to anatomical data on the skull of other sauropodomorphs. These data are then used to establish possible cranial autapomorphies of *M. carinatus*, test its phylogenetic position by comparing it to related taxa, and form a strong basis for future studies of the growth and development of this important dinosaur taxon. *M. carinatus* is one of the only sauropodomorphs for which a relatively complete size series is known, therefore using these new data in conjunction with scans of the other skulls could allow for the understanding of brain development and how the skull bones change in size and shape during growth.

## Materials and Methods

**Figure 1:**
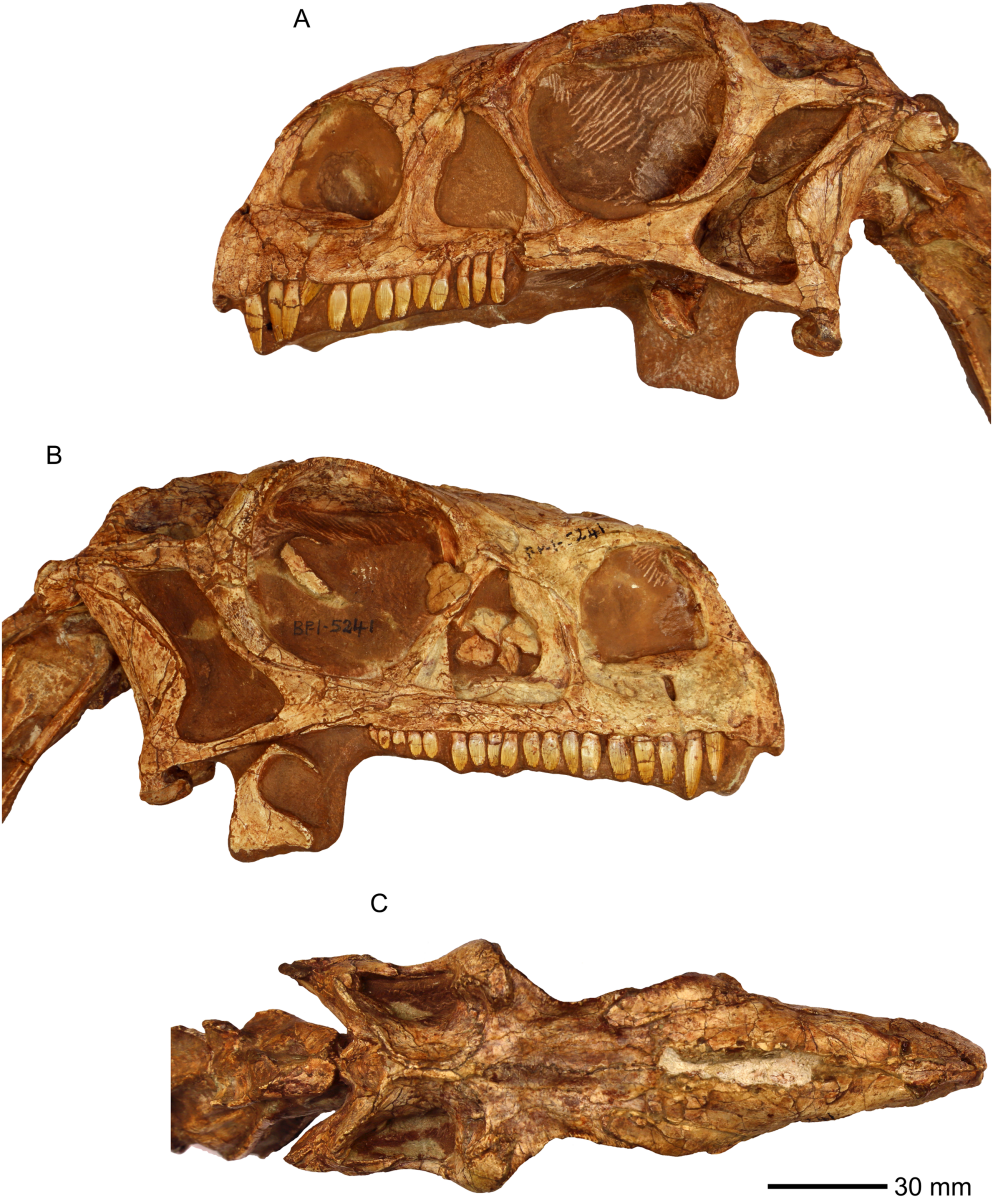
Photographs of the skull of BP/1/5241. (A) Left lateral view. (B) Right lateral view. (C) Dorsal view.

Scanning was conducted using the Wits Microfocus X-ray computed tomography (CT) facility of the Palaeosciences Centre at the University of the Witwatersrand. The facility uses a Nikon Metrology XTH 225/320 LC dual source industrial CT system. We attempted to scan the neotype skull (BP/1/4934), but separation between matrix and bone was insufficient to yield interpretable results. We therefore focused our efforts on BP/1/5241 (Fig. 1), a specimen approximately 14% smaller in cranial anteroposterior length than the neotype skull referred to *M. carinatus* (Gow, Kitching & Raath, 1990; Sues et al., 2004). This specimen is used as an exemplar for *M. carinatus* in our descriptions and comparisons. We find it likely that this specimen belongs to the same taxon as the neotype, because it agrees in nearly every comparable detail in anatomy. BP/1/5241 shares an autapomorphic feature with the holotype (see SYSTEMATIC PALAEONTOLOGY in results section). This specimen had excellent contrast between fossil bone and rock matrix. It was scanned at approximately 107 μm resolution, with the X-ray characteristics set at 100 kV and 680 μA, and a 1.8 mm thick copper filter applied. The resulting data dimensions were as follows: 1,000 × 1,000 × 1,000 with VoxelSize = 0.1068mm. Raw CT scan data (DICOM stack format) are available on the following permalink: http://www.morphosource.org/Detail/ProjectDetail/Show/project_id/426. The scans were digitally segmented using the software VG Studio Max v.2.1 (Volume Graphics, Heidelberg, Germany). Each cranial bone was individually segmented. The internal structures of the braincase, such as the inner ear and stapes were also digitally reconstructed. Reconstructing the endocast was beyond the scope of this research and will be presented elsewhere.

The individual bones were described in detail using standard comparative anatomical techniques. Skull material is rare in sauropodomorphs, and we focused our cranial comparisons on well-represented skulls of *Plateosaurus erlenbergiensis*, *Lufengosaurus huenei*, and *Sarahsaurus aurifontanalis*, which we were able to personally inspect (Barrett, Upchurch & Wang, 2005; Prieto-Márquez & Norell, 2011; Rowe, Sues & Reisz, 2010). We also provide comparisons where appropriate to other sauropodomorph taxa with cranial material, notably *Efraasia minor*, using published literature (Apaldetti et al., 2011, 2014; Barrett et al., 2007; Bronzati & Rauhut, 2017; Chatterjee & Zheng, 2002; Dzik, 2003; Ewer, 1965; Galton & Kermack, 2010; He, Li & Cai, 1988; Martínez, 2009; Martinez & Alcober, 2009; Martínez, Haro & Apaldetti, 2012; Ouyang & Ye, 2001; Sereno & Arcucci, 1994; Sereno, Martínez & Alcober, 2012; Sereno & Novas, 1993; Yates, 2010; Zhang & Yang, 1994). Sources for available comparative observations are listed in Table 1, and citations to specimen numbers and publications are provided in that table to save line space in the descriptive section.

**Table 1:** Table of comparative material.

Taxon	Observations and scores based on	Specimen number	Location of specimen
*Euparkeria*	Ewer (1965)	SAM 5867, holotype SAM 6047A, R527A	Iziko South African Museum, Cape Town, South Africa D. M. S. Watson Collection, University College London, London, United Kingdom
*Marasuchus lilloensis*	Sereno & Arcucci (1994)	PVL 3872	Instituto Miguel Lillo, Universidad de Tucuman, Tucuman, Argentina
*Aardonyx celestae*	Personal specimen examination	BP/1/6254, BP/1/6505, BP/1/6584, BP/1/6334, holotypes	Evolutionary Studies Institute, University of the Witwatersrand, Johannesburg, South Africa
*Adeopapposaurus mognai*	Martínez (2009)	PVSJ610 and PVSJ568	Instituto y Museo de Ciencias Naturales, Universidad Nacional de San Juan, San Juan, Argentina
*Anchisaurus polyzelus*	Yates (2010) and personal specimen examination	YPM 1883	Peabody Museum of Natural History, Yale University, New Haven, United States
*Coloradisaurus brevis*	Apaldetti et al. (2014)	PVL 3967, holotype	Instituto Miguel Lillo, Universidad de Tucuman, Tucuman, Argentina
*Efraasia minor*	Bronzati & Rauhut (2017)	SMNS 12667	Staatliches Museum für Naturkunde, Stuttgart, Germany
*Eoraptor lunensis*	Sereno, Martínez & Alcober (2012)	PVSJ 512	Instituto y Museo de Ciencias Naturales, Universidad Nacional de San Juan, San Juan, Argentina
*Herrerasaurus ischigualastensis*	Sereno & Novas (1993)	PVSJ 407	Instituto y Museo de Ciencias Naturales, Universidad Nacional de San Juan, San Juan, Argentina
*Jingshanosaurus xinwaensis*	Zhang & Yang (1994)	LV 003, holotype	Museum of Lufeng Dinosaurs, Yunnan, China
*Leyesaurus marayensis*	Apaldetti et al. (2011)	PVSJ 706, holotype	Instituto y Museo de Ciencias Naturales, Universidad Nacional de San Juan, San Juan, Argentina
*Lufengosaurus huenei*	Personal specimen examination	IVPP V15	Institute of Vertebrate Palaeontology, Beijing, China
*Mamenchisaurus youngi*	Ouyang & Ye (2001)	ZDM0083, holotype	Zigong Dinosaur Museum, Zigong, China
*Massospondylus kaalae*	Personal specimen examination	SAMPK-K1325, holotype	Iziko South African Museum, Cape Town, South Africa
*Melanorosaurus readi*	Personal specimen examination	NMQR 3314, holotype	National Museum, Bloemfontein, South Africa
*Omeisaurus tianfuensis*	He, Li & Cai (1988)	ZDM T5702	Zigong Dinosaur Museum, Zigong, China
*Panphagia protos*	Martinez & Alcober (2009) and Martínez, Haro & Apaldetti (2012)	PVSJ 874	Instituto y Museo de Ciencias Naturales, Universidad Nacional de San Juan, San Juan, Argentina
*Pantydraco caducus*	Galton & Kermack (2010)	NHMUK RU P24, holotype	National History Museum, London, United Kingdom
*Plateosaurus erlenbergiensis*	Personal specimen examination	AMNH FARB 6810	American Museum of Natural History, New York, United States of America
*Sarahsaurus aurifontanalis*	Personal specimen examination	MCZ 8893	Museum of Comparative Zoology, Harvard University, Boston, United States of America
*Shunosaurus lii*	Chatterjee & Zheng (2002)	ZG65430	Zigong Dinosaur Museum, Zigong, China
*Silesaurus opolensis*	Dzik (2003)	ZPAL Ab III	Institute of Paleobiology of the Polish Academy of Sciences in Warsaw, Poland
*Yunnanosaurus huangi*	Barrett et al. (2007)	NGMJ 004546, holotype	Nanjing Geological Museum, Nanjing, China

**Note:**

References, specimen numbers and collections used for comparative purposes.

BP/1/5241 was scored for the cranial characters in the basal sauropodomorph data matrix used by Yates et al. (2010), which is modified from the earlier matrix used by Yates (2007). This matrix comprises 353 characters, of which 120 regard craniodental homologies. It includes original characters as well as characters acquired and/or modified from 19 other sources (Barrett, Upchurch & Wang, 2005; Benton et al., 2000; Galton, 1990; Galton & Bakker, 1985; Galton & Upchurch, 2004; Gauthier, 1986; Holtz, 1994; Langer, 2004; Leal et al., 2004; Rauhut, 2003; Sereno, 1999; Sereno et al., 1993, 1996; Upchurch, 1995, 1998; Wilson, 2002; Wilson & Sereno, 1998; Yates, 2003a, 2003b, 2003c). We added 27 new cranial characters and deleted five pre-existing characters to optimize the number of characters useful for determining relationships in non-sauropodan Sauropodomorpha. We also revised the scores of several existing cranial characters to make more explicit homology statements (Supplemental Information 1).

Scores for the characters were stored and managed with Mesquite v3.04 (Maddison & Maddison, 2015). BP/1/5241 was used as a specimen-level exemplar for *M. carinatus*. The completed matrix was exported as a TNT file for heuristic searches for optimal tree topologies under the parsimony criterion. Analyses were conducted in TNT ver. 1.5 (Goloboff, Farris & Nixon, 2008). First, using the ‘Stabilize Consensus’ option in the ‘New Technology Search’ using sectorial searches and tree fusing, with the consensus stabilized five times. Trees obtained using this analysis were then submitted to an additional round of ‘traditional search’ swapping using tree bisection-reconstruction (TBR) and stopping when maxtrees hit.

## Results

### Skull

All the cranial bones are essentially complete and lie approximately in life position, except for some palatal bones such as the ectopterygoid, which are slightly disarticulated (Figs. 1–53; Supplemental Information 2).

**Figure 2:**
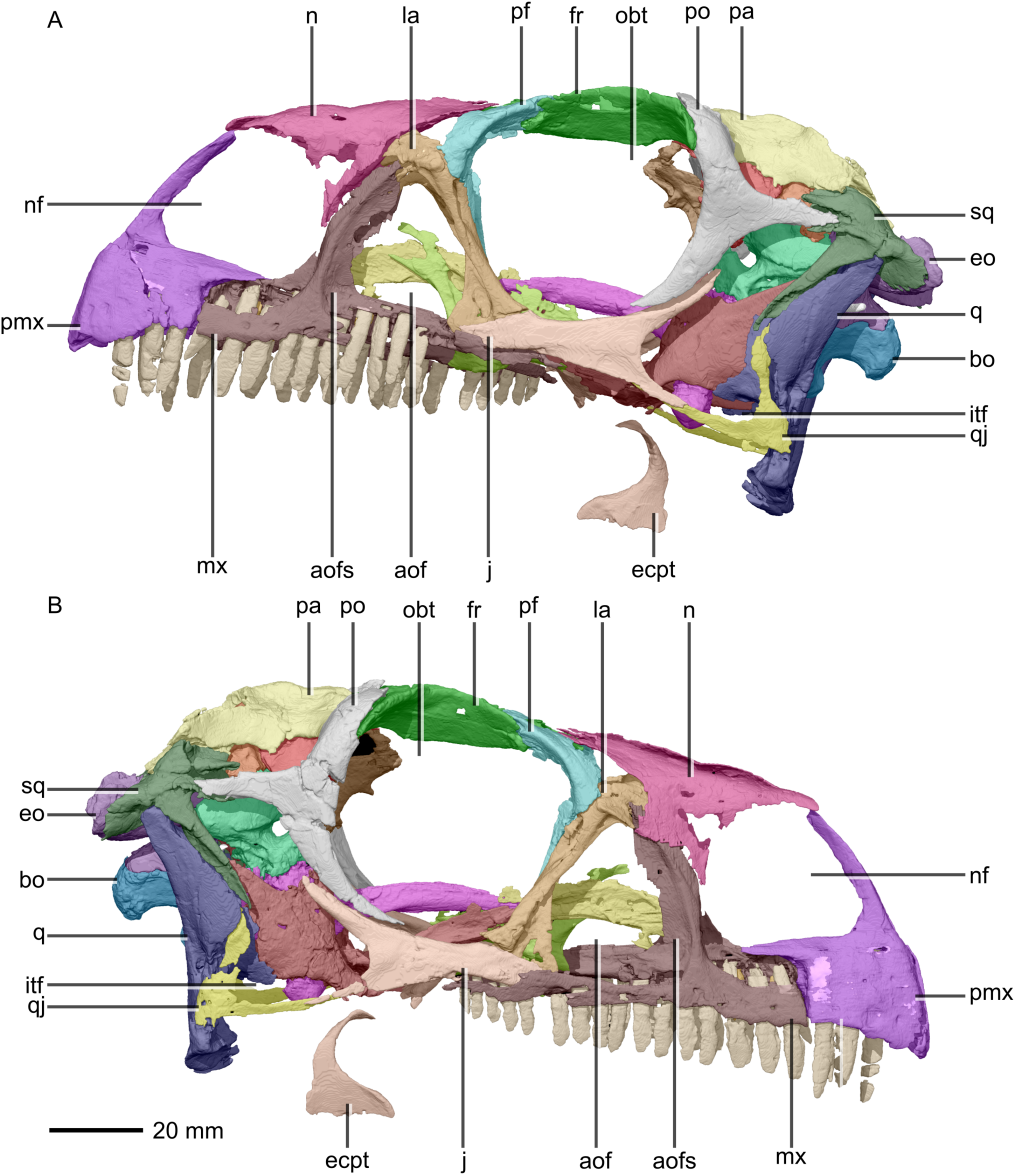
Reconstructed skull of BP/1/5241. (A) Left lateral view. (B) Right lateral view. aof, antorbital fenestra; aofs, antorbital fossa; bo, basioccipital; ecpt, ectopterygoid; eo, exoccipital; fr, frontal; itf, infratemporal fenestra; j, jugal; la, lacrimal; mx, maxilla; n, nasal; nf, narial fenestra; obt, orbit; pa, parietal; pf, prefrontal; pmx, premaxilla; po, postorbital; q, quadrate; qj, quadratojugal; sq, squamosal.

**Figure 3:**
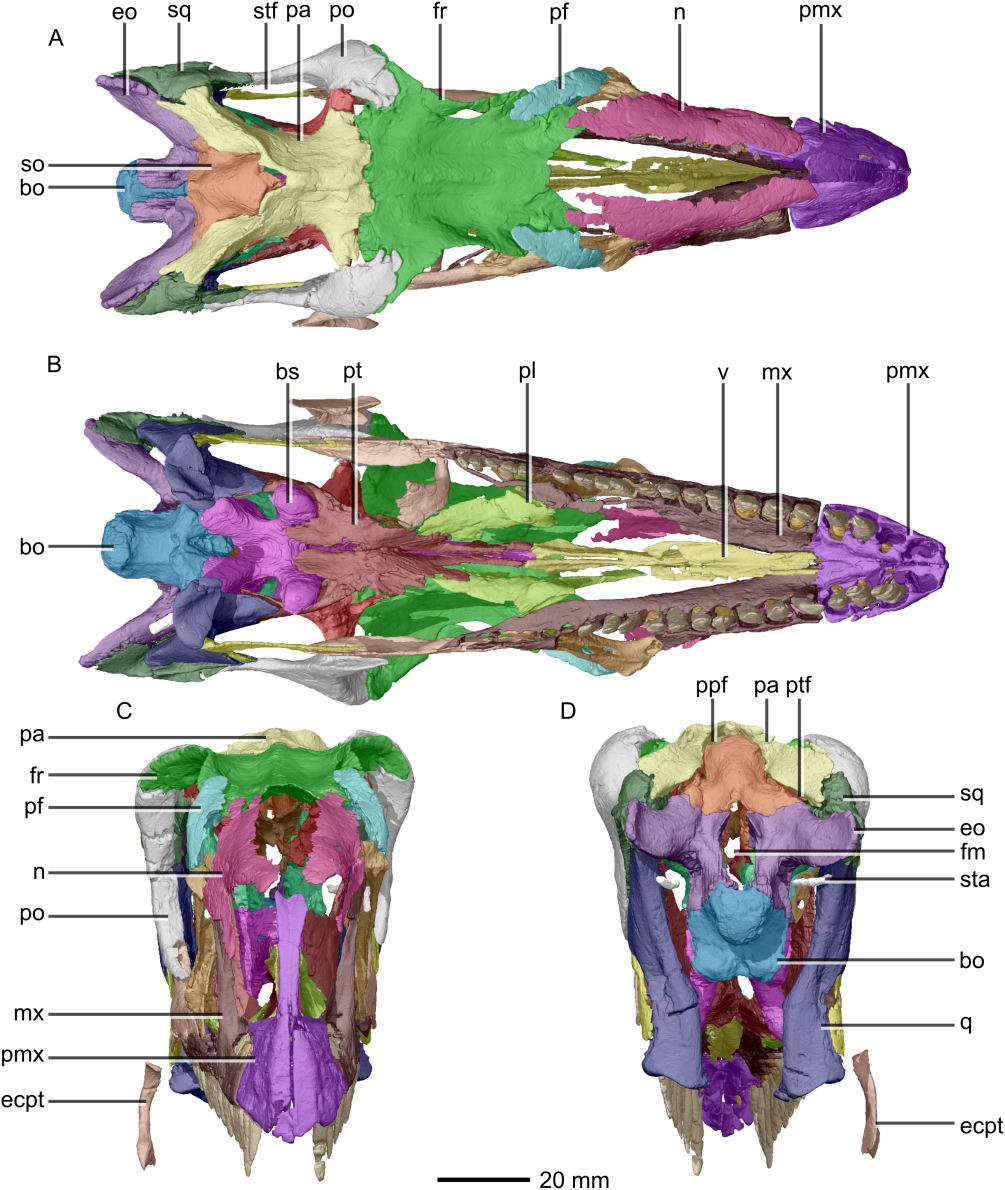
Reconstructed skull of BP/1/5241. (A) Dorsal view. (B) Ventral view. (C) Anterior view. (D) Posterior view. bo, basioccipital; bs, basisphenoid; ecpt, ectopterygoid; eo, exoccipital; fr, frontal; mx, maxilla; n, nasal; pa, parietal; pf, prefrontal; pl, palatine; pmx, premaxilla; po, postorbital; ppf, postparietal fenestra; pt, pterygoid; q, quadrate; so, supraoccipital; sq, squamosal; sta, stapes; stf, supratemporal fenestra; v, vomer.

**Figure 4:**
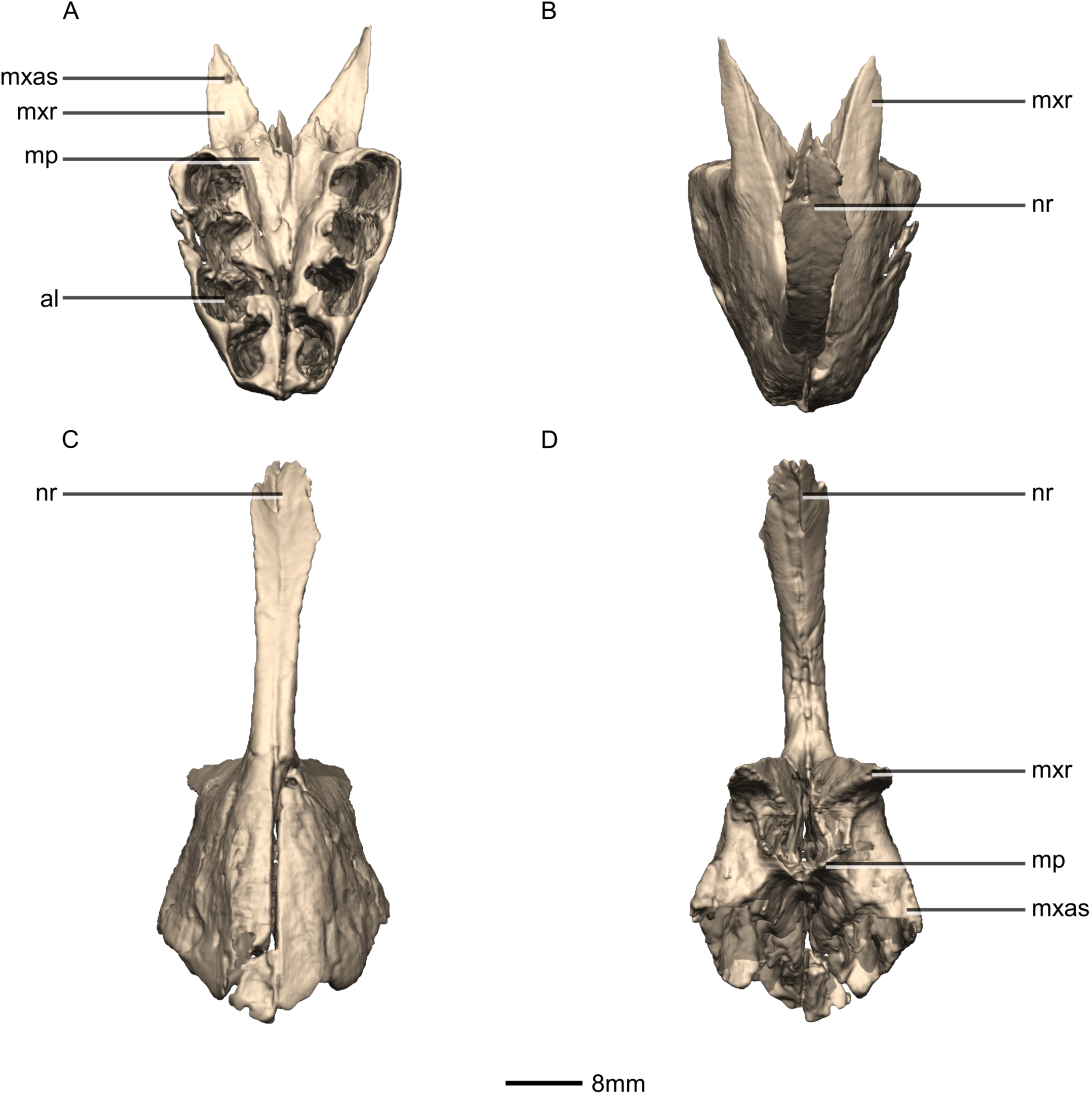
Reconstructed premaxilla of BP/1/5241. (A) Ventral view. (B) Dorsal view. (C) Anterior view. (D) Posterior view. al, alveoli; mp, medial process; mxas, maxillary articular surface; mxr, maxillary ramus; nr, nasal ramus.

**Figure 5:**
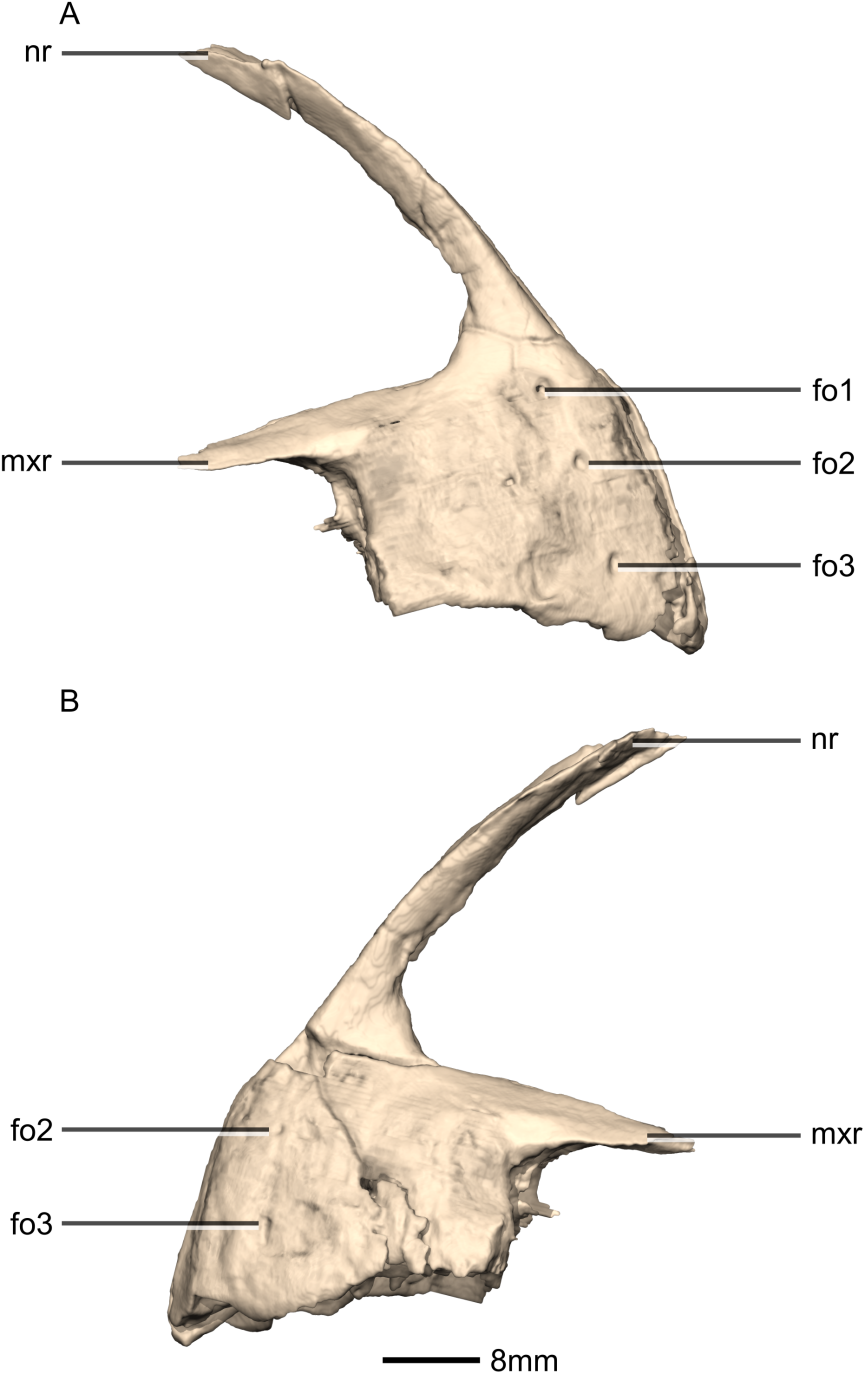
Reconstructed premaxilla of BP/1/5241. (A) Right lateral view. (B) Left lateral view. fo, foramen; mxr, maxillary ramus; nr, nasal ramus.

**Figure 6:**
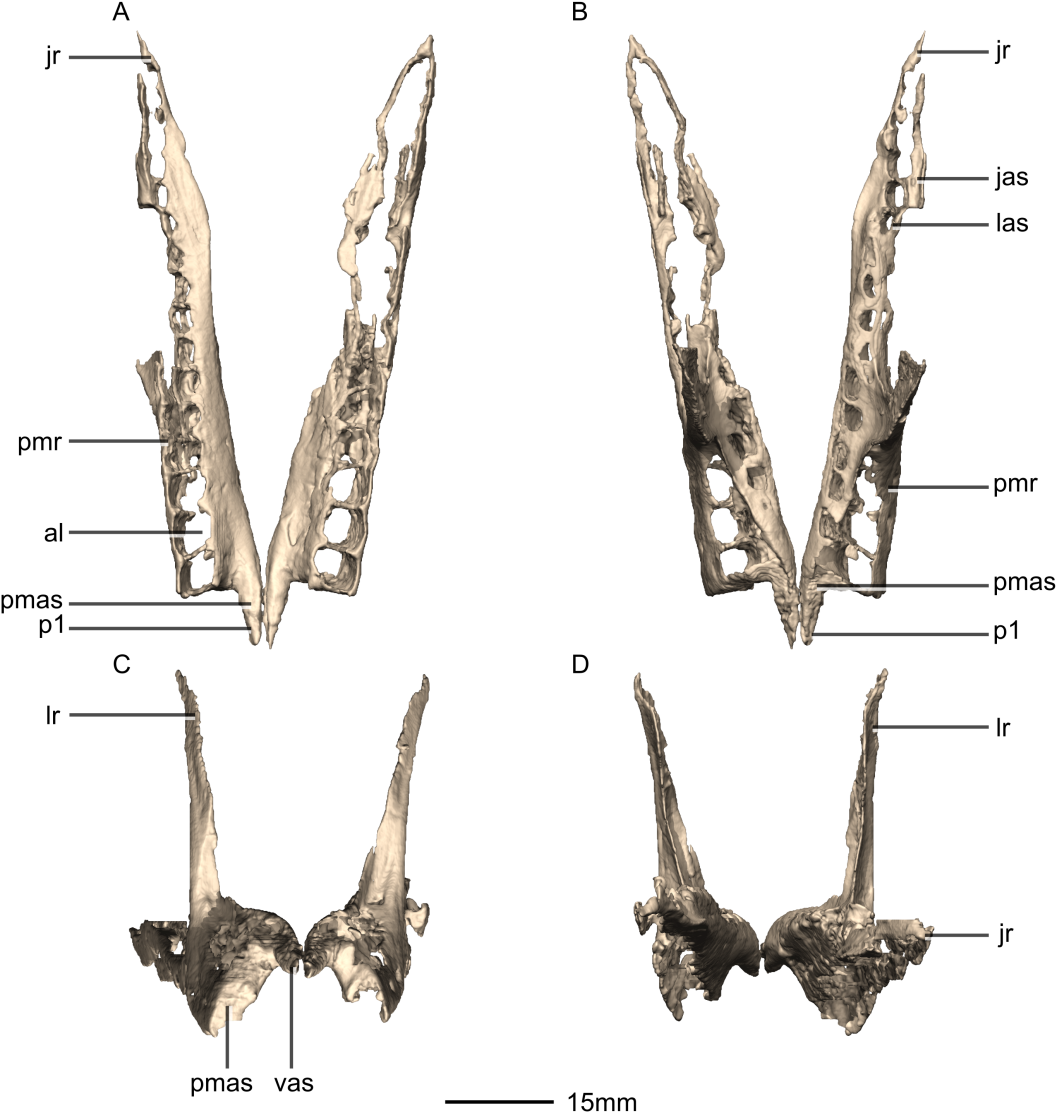
Reconstructed left and right maxillae of BP/1/5241. (A) Ventral view. (B) Dorsal view. (C) Anterior view. (D) Posterior view. al, alveoli; jas, jugal articular surface; jr, jugal ramus; las, lacrimal articular surface; lr, lacrimal ramus; p, process; pmas, premaxillary articular surface; pmr, premaxillary ramus; vas, vomerine articular surface.

**Figure 7:**
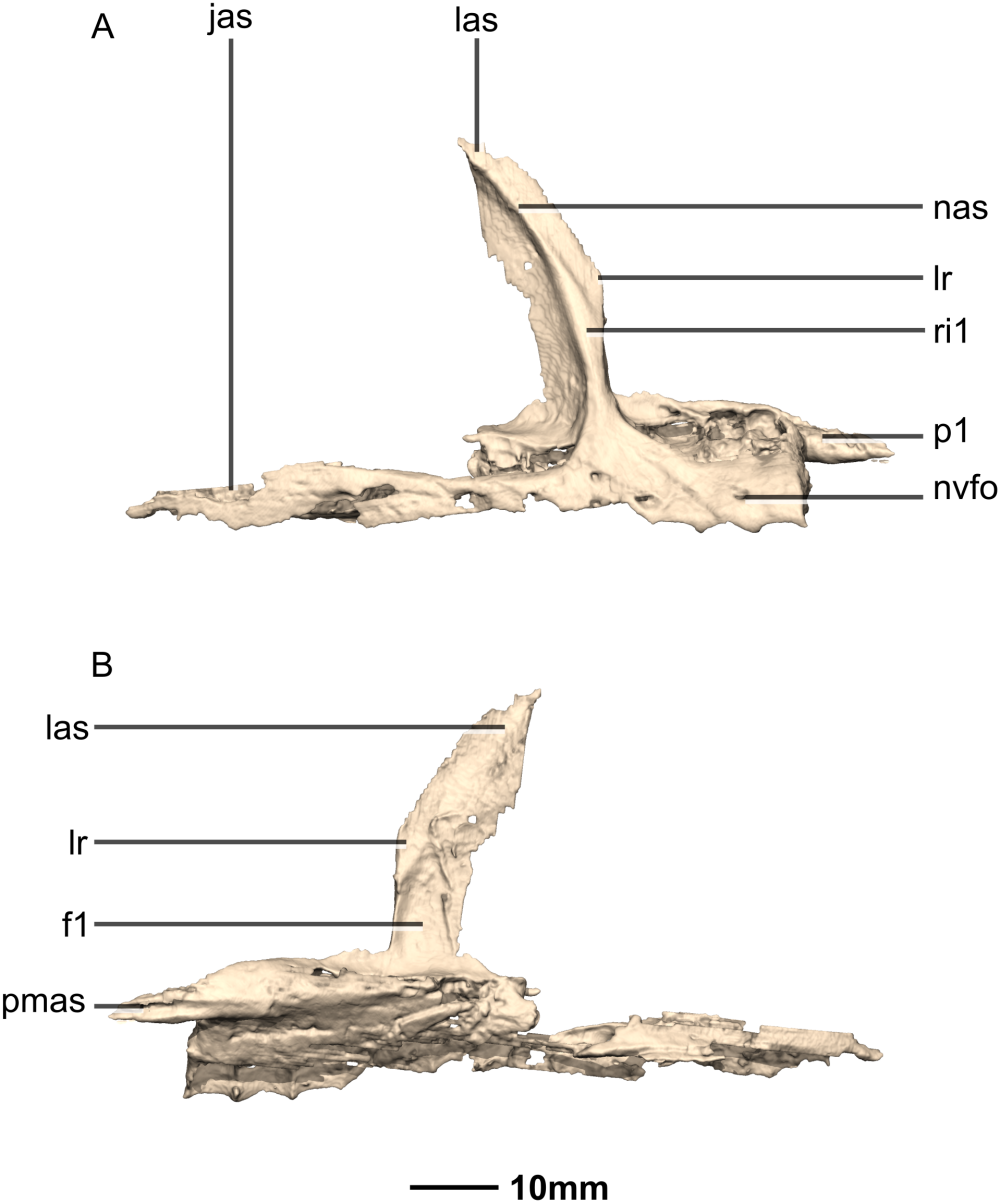
Reconstructed right maxilla of BP/1/5241. (A) Right lateral view. (B) Right medial view. f, fossa; jas, jugal articular surface; las, lacrimal articular surface; lr, lacrimal ramus; nas, nasal articular surface; nvfo, neurovascular foramina; p, process; pmas, premaxillary articular surface; ri, ridge.

**Figure 8:**
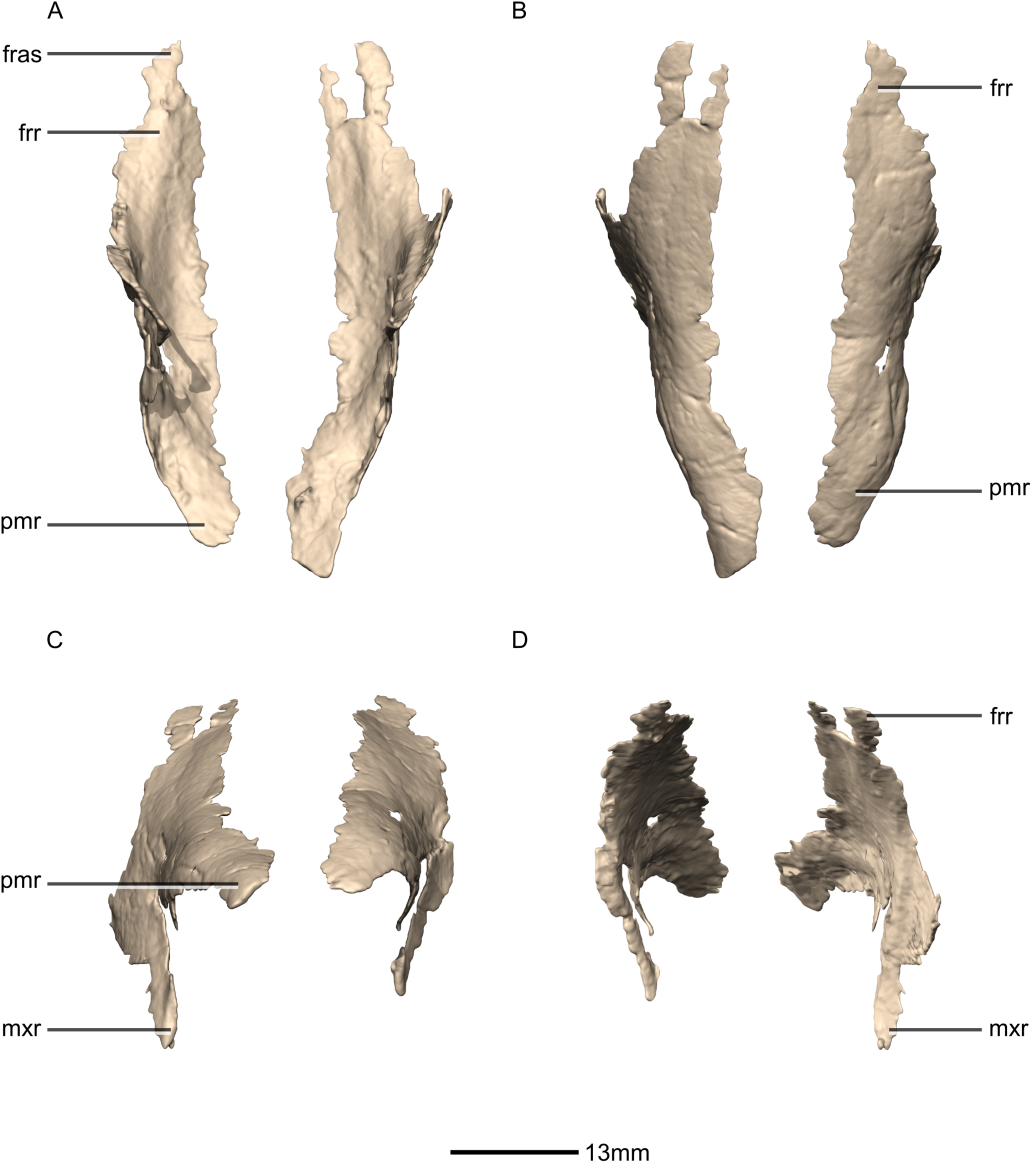
Reconstructed left and right nasals of BP/1/5241. (A) Ventral view. (B) Dorsal view. (C) Anterior view. (D) Posterior view. frr, frontal ramus; fras, frontal articular surface; mxr, maxillary ramus; pmr, premaxillary ramus.

**Figure 9:**
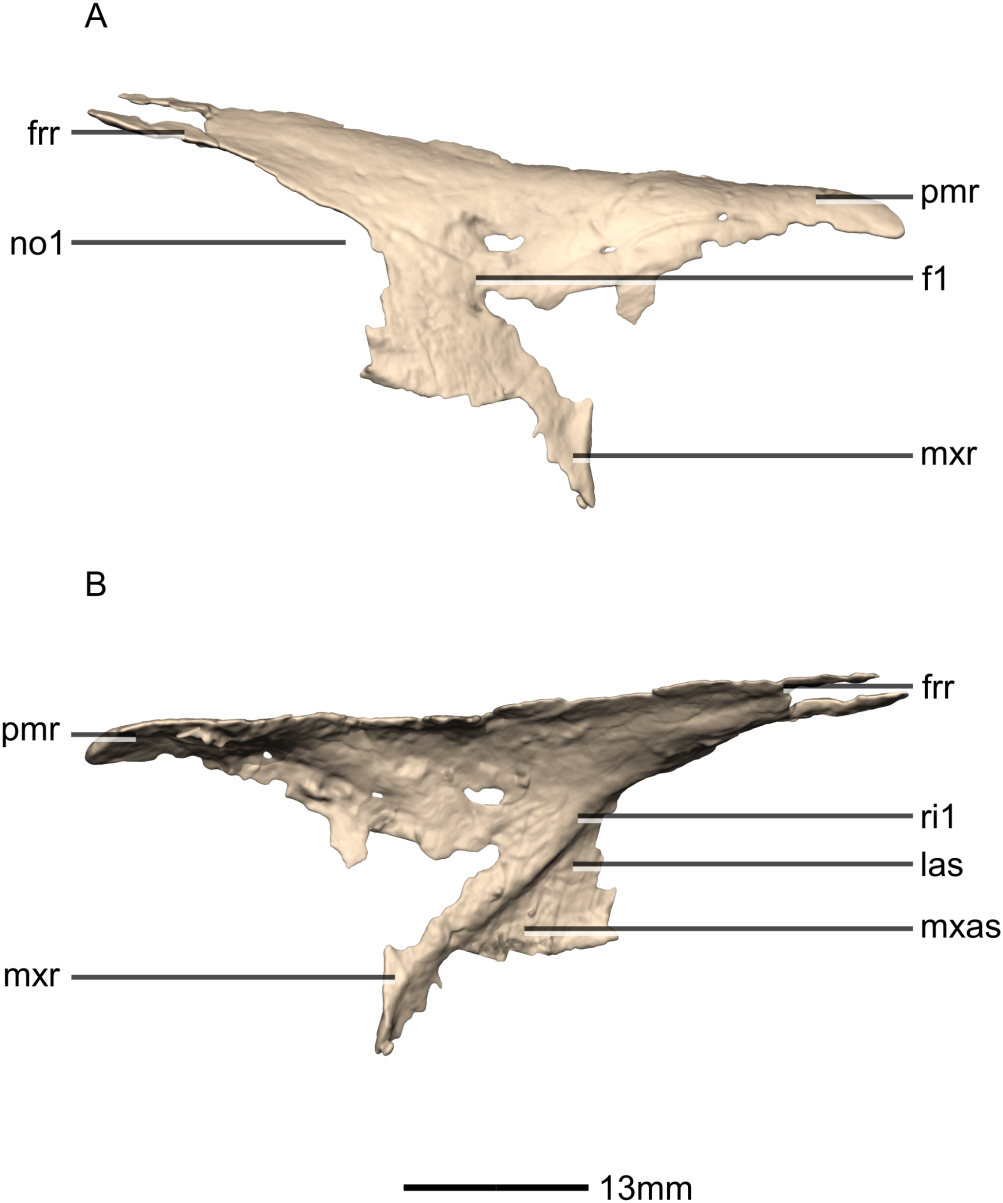
Reconstructed right nasal of BP/1/5241. (A) Right lateral view. (B) Right medial view. f, fossa; frr, frontal ramus; las, lacrimal articular surface; mxas, maxilla articular surface; mxr, maxillary ramus; no, notch; pmr, premaxillary ramus; ri, ridge.

**Figure 10:**
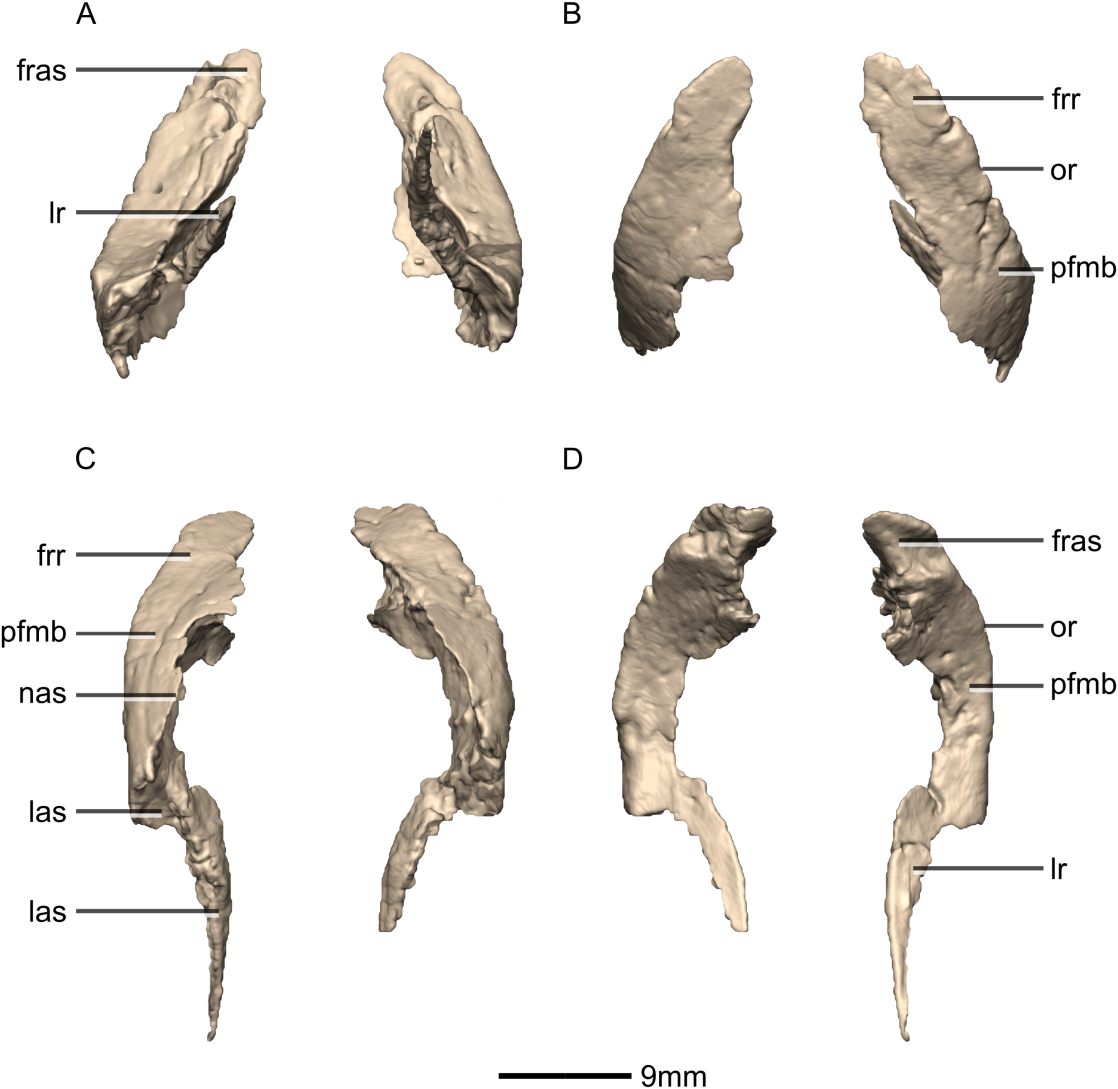
Reconstructed left and right prefrontals of BP/1/5241. (A) Ventral view. (B) Dorsal view. (C) Anterior view. (D) Posterior view. frr, frontal ramus; fras, frontal articular surface; las, lacrimal articular surface; lr, lacrimal ramus; nas, nasal articular surface; or, orbital rim; pfmb, prefrontal main body.

**Figure 11:**
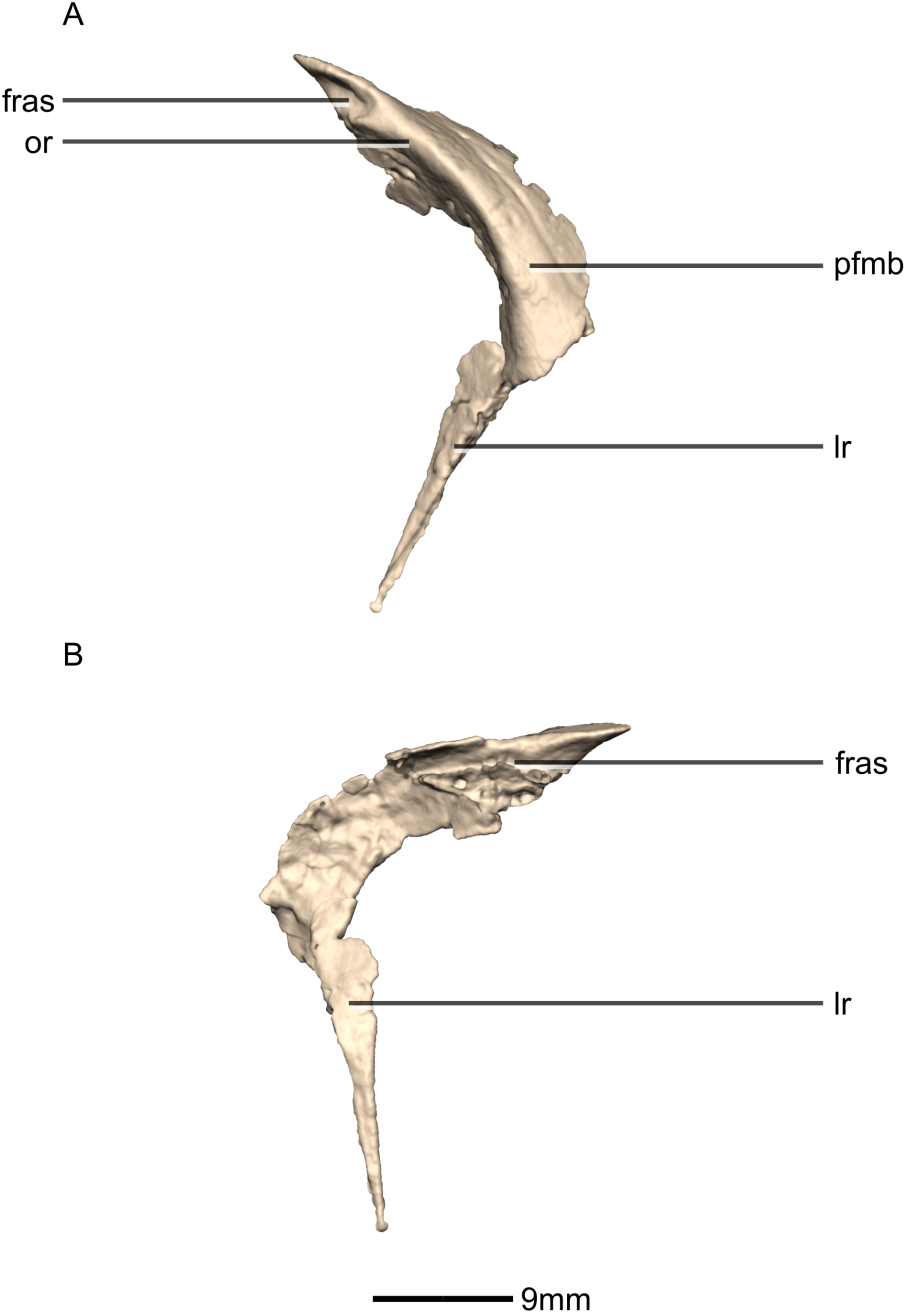
Reconstructed right prefrontal of BP/1/5241. (A) Right lateral view. (B) Right medial view. fras, frontal articular surface; lr, lacrimal ramus; or, orbital rim; pfmb, prefrontal main body.

**Figure 12:**
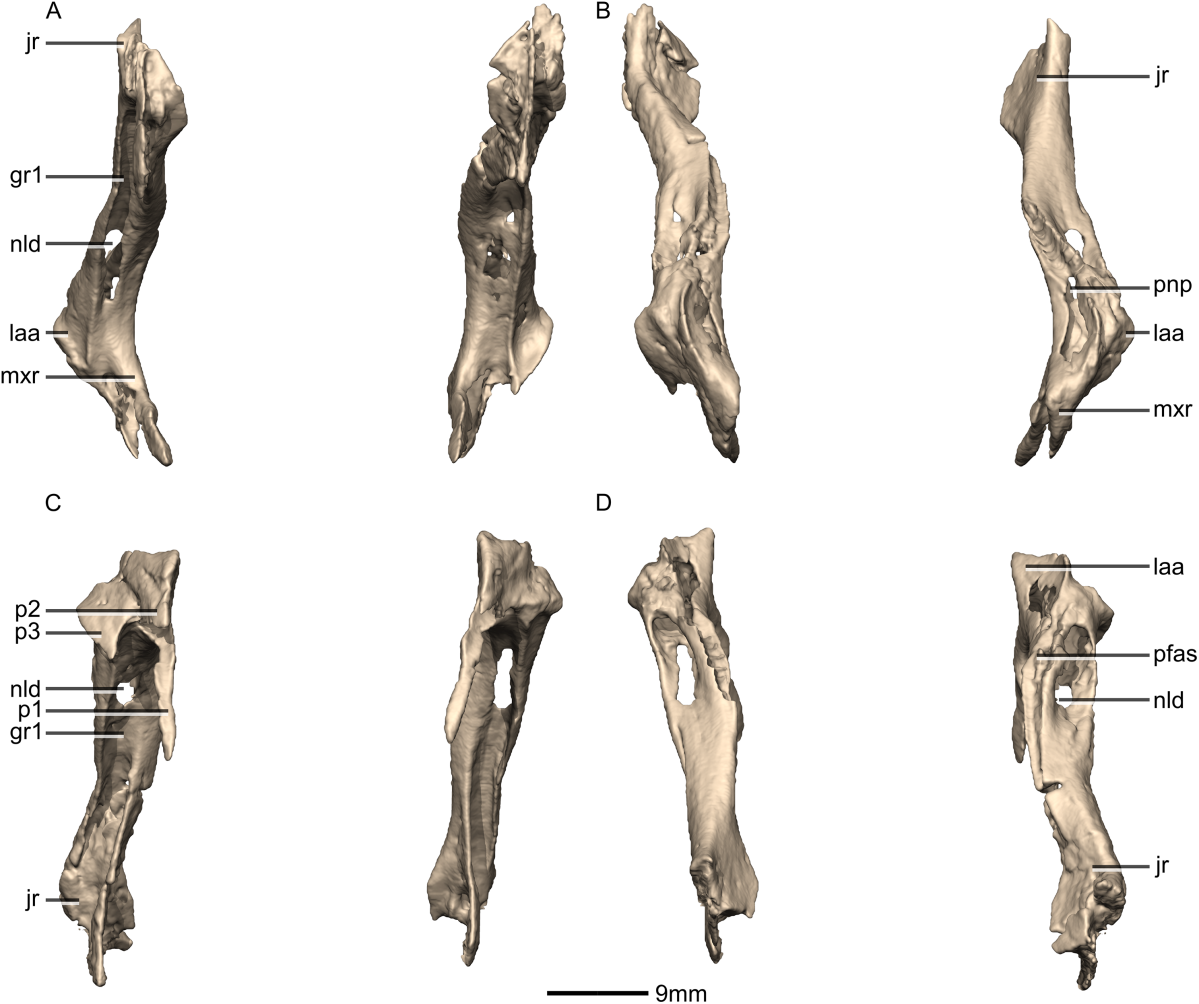
Reconstructed left and right lacrimals of BP/1/5241. (A) Ventral view. (B) Dorsal view. (C) Anterior view. (D) Posterior view. gr, groove; jr, jugal ramus; laa, lacrimal angle; mxr, maxillary ramus; nld, nasolacrimal duct; p, process; pfas, prefrontal articular surface; pnp, pneumatic pocket.

**Figure 13:**
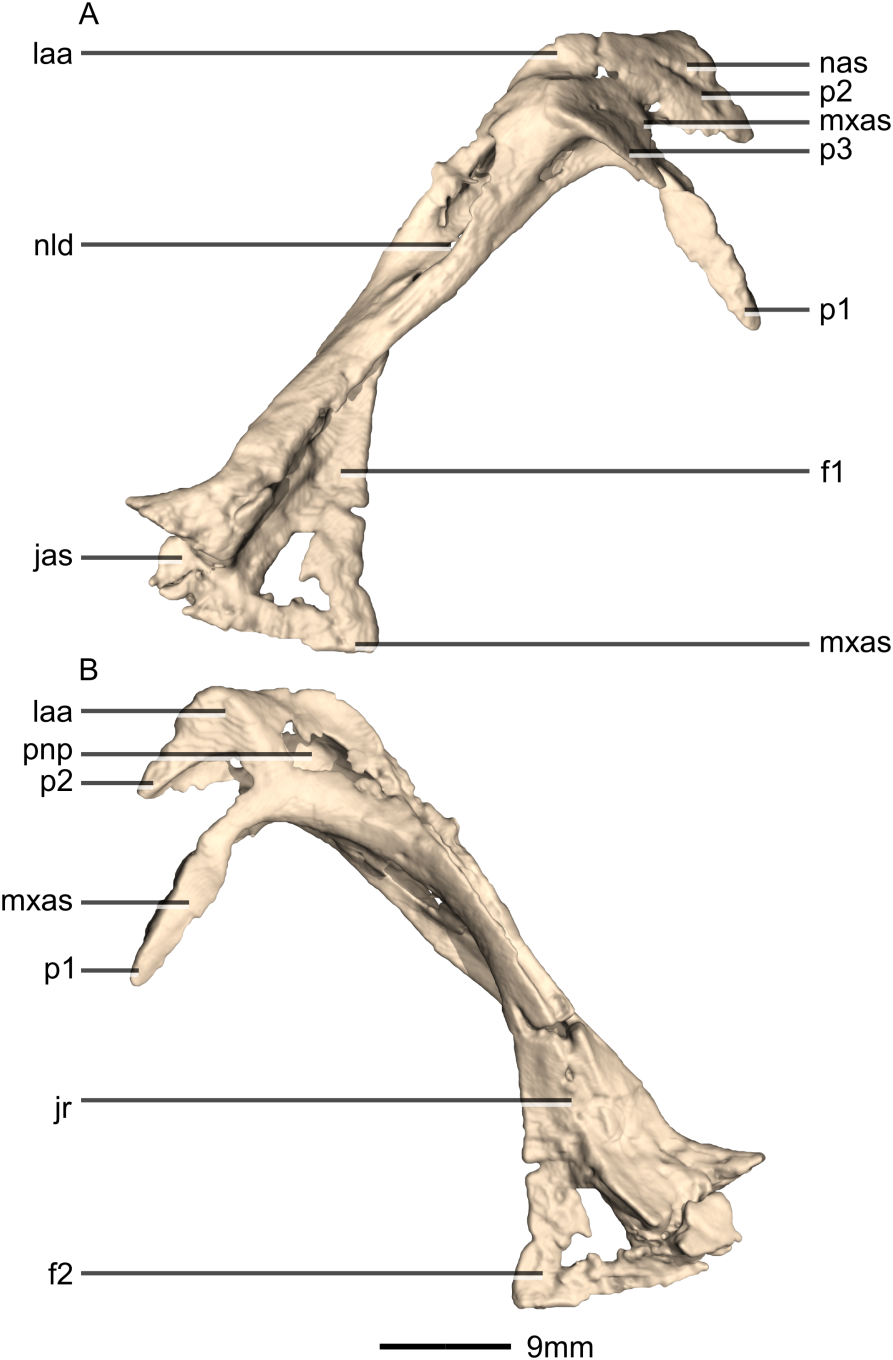
Reconstructed right lacrimal of BP/1/5241. (A) Right lateral view. (B) Right medial view. f, fossa; jas, jugal articular surface; jr, jugal ramus; laa, lacrimal angle; mxas, maxilla articular surface; nas, nasal articular surface; p, process; pnp, pneumatic pocket.

**Figure 14:**
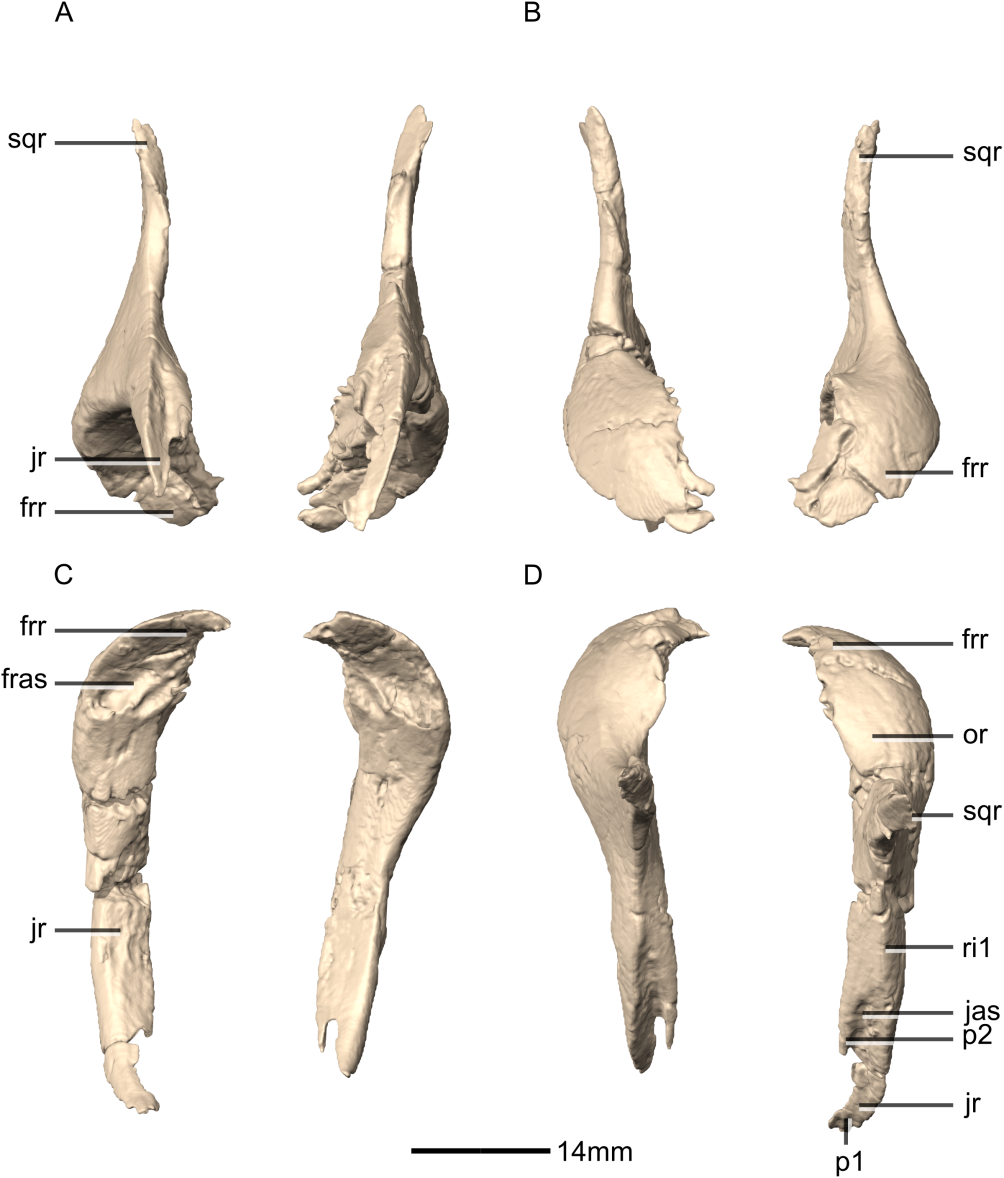
Reconstructed left and right postorbitals of BP/1/5241. (A) Ventral view. (B) Dorsal view. (C) Anterior view. (D) Posterior view. fras, frontal articular surface; frr, frontal ramus; jas, jugal articular surface; jr, jugal ramus; or, orbital rim; p, process; ri, ridge; sqr, squamosal ramus.

**Figure 15:**
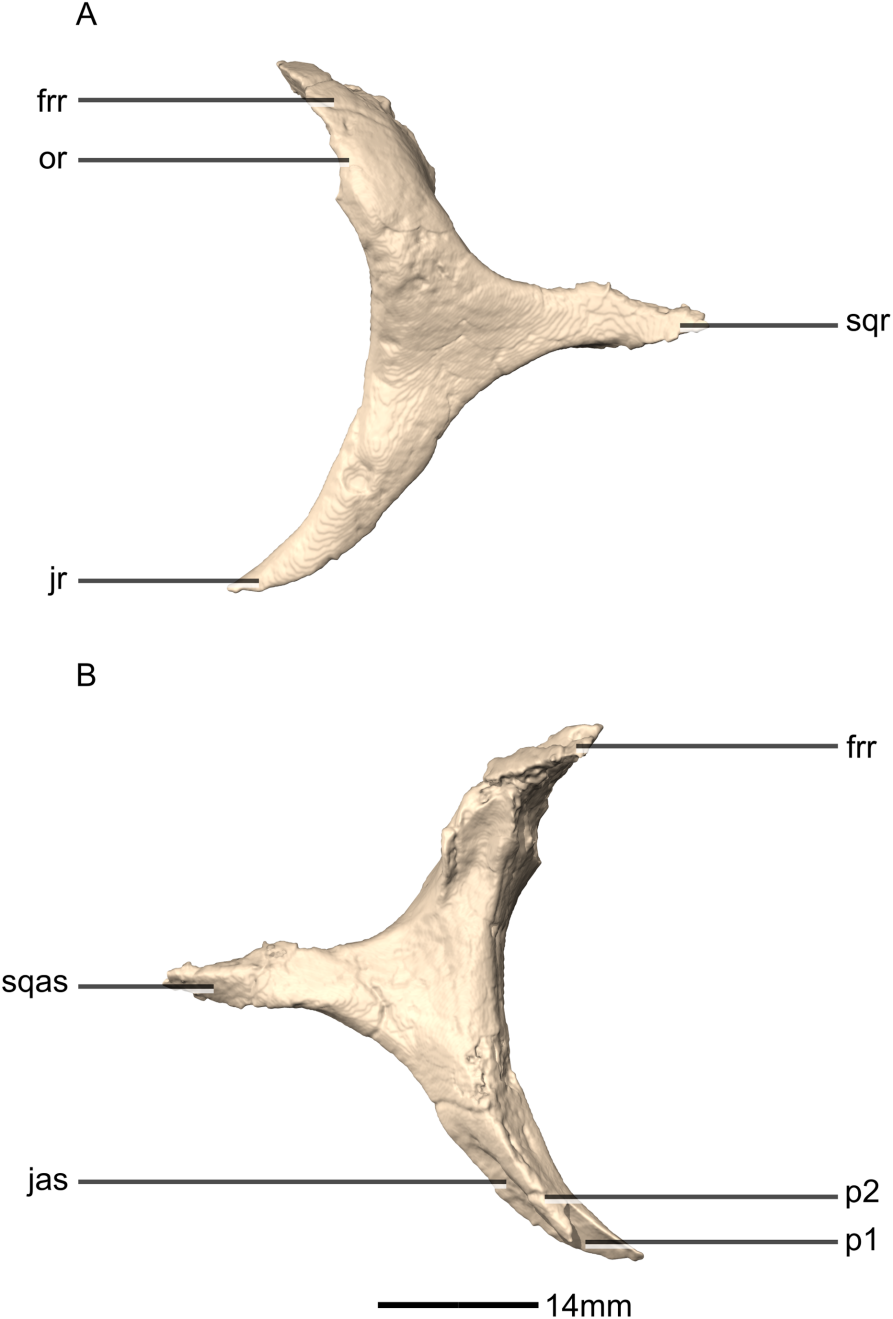
Reconstructed left postorbital of BP/1/5241. (A) Left lateral view. (B) Left medial view. frr, frontal ramus; jas, jugal articular surface; jr, jugal ramus; or, orbital rim; p, process; sqas, squamosal articular surface; sqr, squamosal ramus.

**Figure 16:**
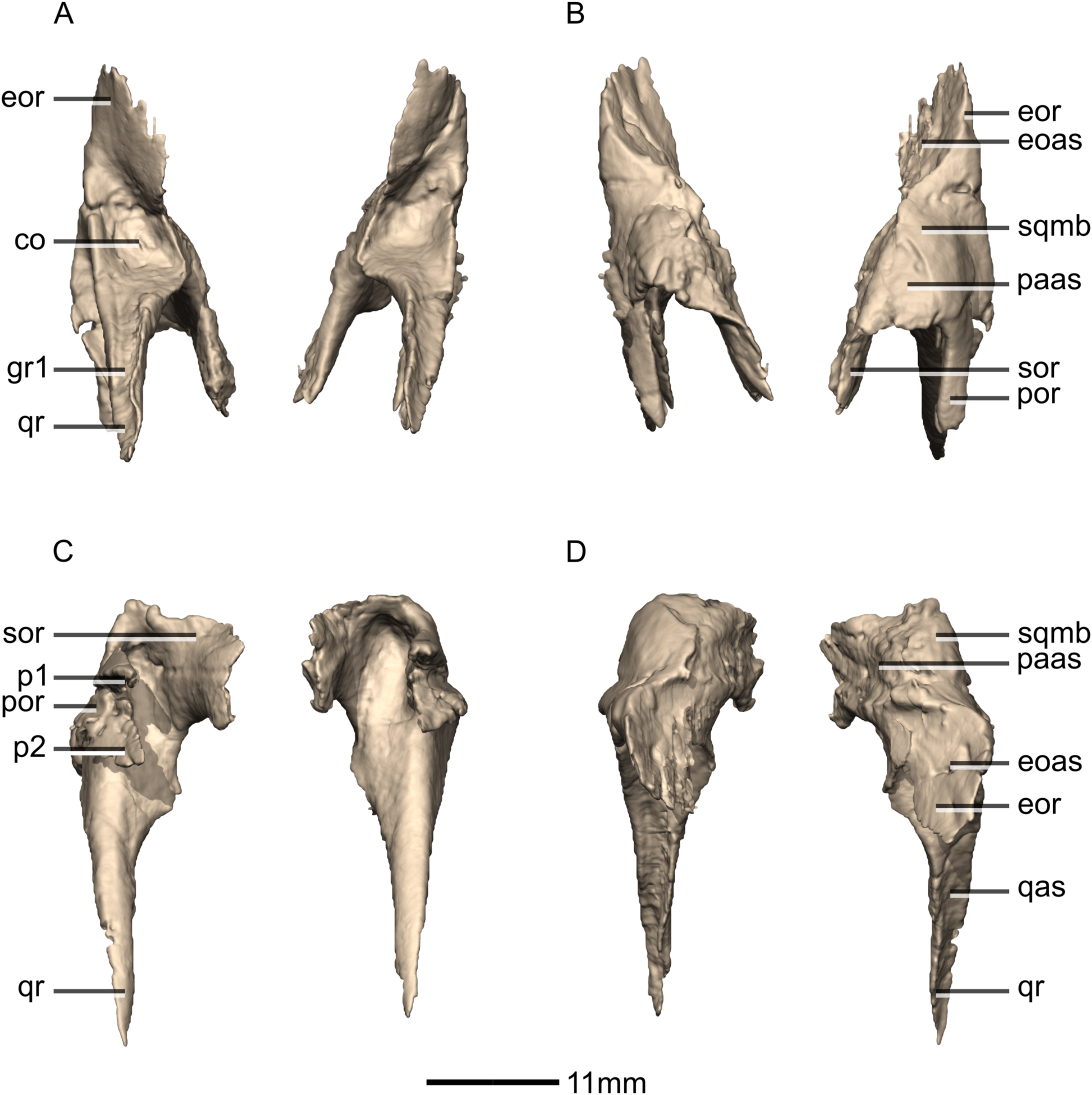
Reconstructed left and right squamosals of BP/1/5241. (A) Ventral view. (B) Dorsal view. (C) Anterior view. (D) Posterior view. co, cotyle; eoas, exoccipital articular surface; eor, exoccipital ramus; gr, groove; paas, parietal articular surface; por, postorbital ramus; p, process; qas, quadrate articular surface; qr, quadrate ramus; sor, supraoccipital ramus; sqmb, squamosal body.

**Figure 17:**
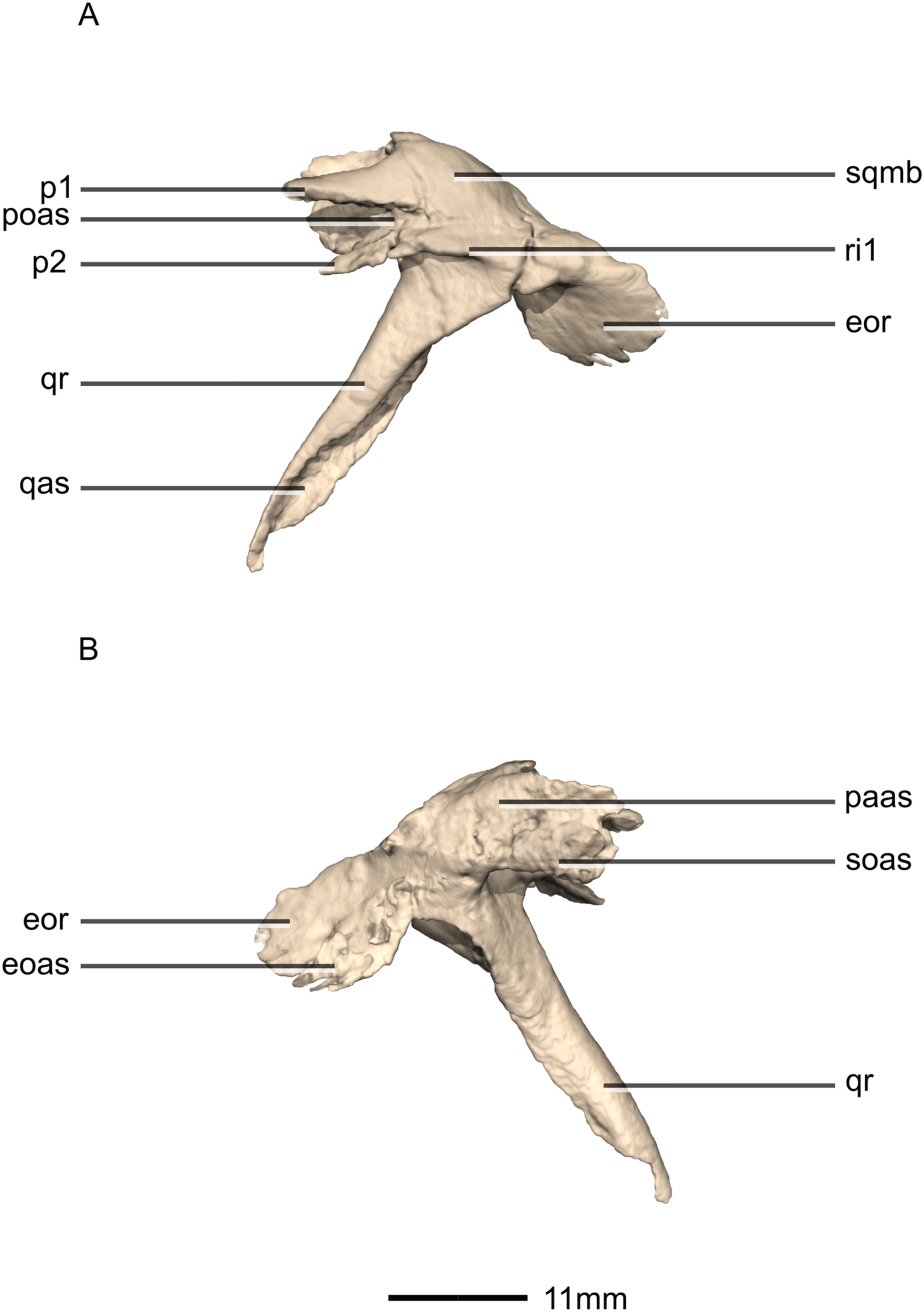
Reconstructed left squamosal of BP/1/5241. (A) Left lateral view. (B) Left medial view. eoas, exoccipital articular surface; eor, exoccipital ramus; paas, parietal articular surface; poas, postorbital articular surface; p, process; qas, quadrate articular surface; qr, quadrate ramus; ri, ridge; soas, supraoccipital articular surface; sqmb, squamosal body.

**Figure 18:**
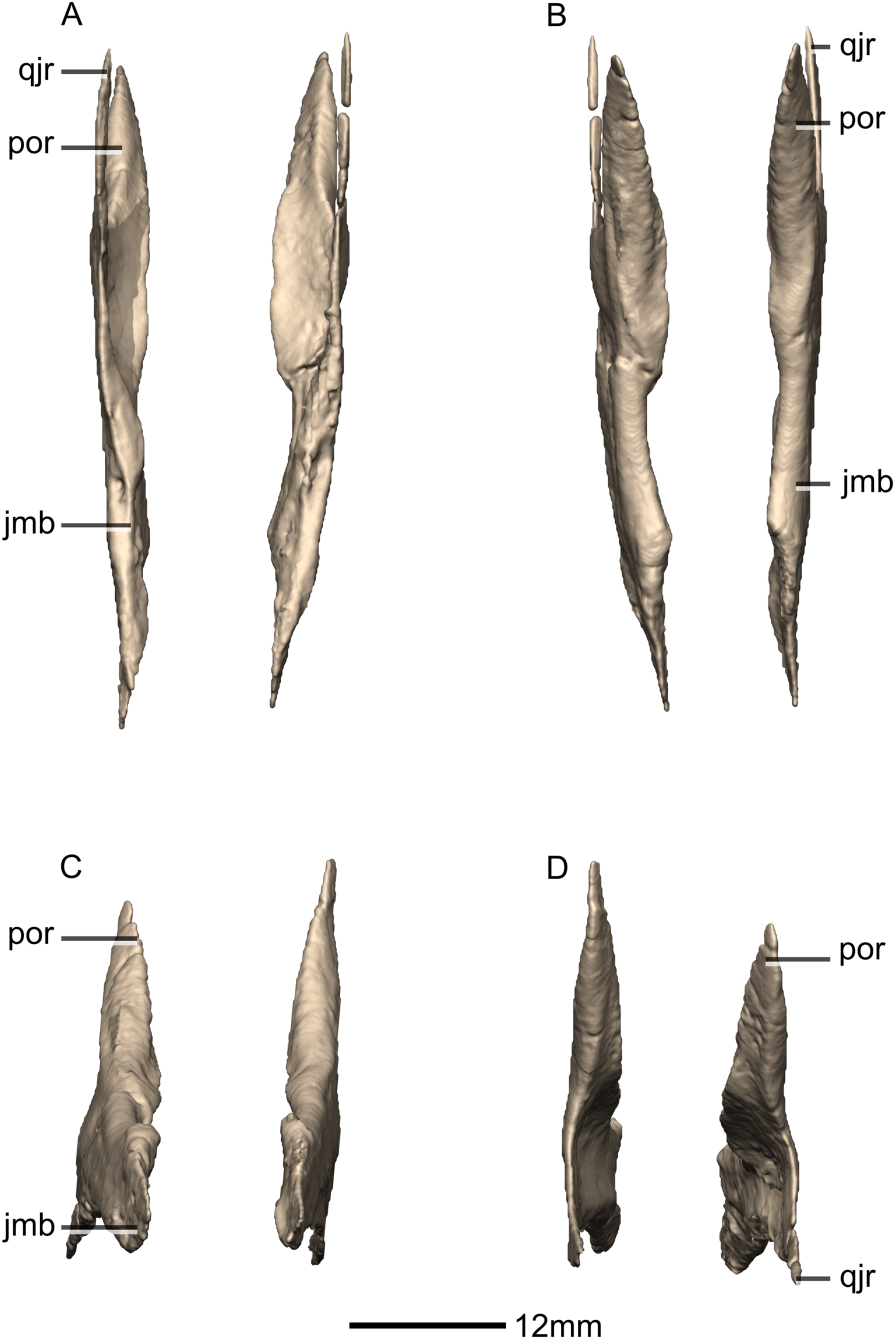
Reconstructed left and right jugals of BP/1/5241. (A) Ventral view. (B) Dorsal view. (C) Anterior view. (D) Posterior view. jmb, jugal main body; por, postorbital ramus; qjr, quadratojugal ramus.

**Figure 19:**
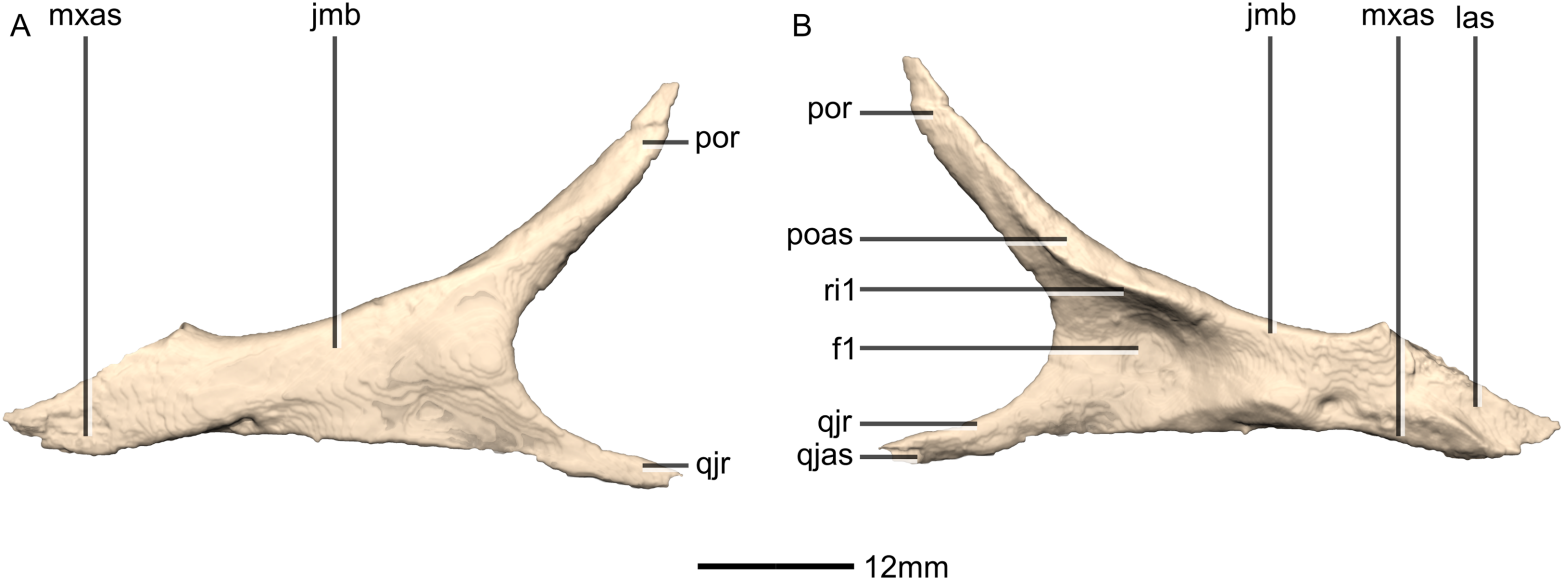
Reconstructed left jugal of BP/1/5241. (A) Left lateral view. (B) Left medial view. f, fossa; jmb, jugal main body; las, lacrimal articular surface; mxas, maxilla articular surface; poas, postorbital articular surface; por, postorbital ramus; qjas, quadratojugal articular surface; qjr, quadratojugal ramus; ri, ridge.

**Figure 20:**
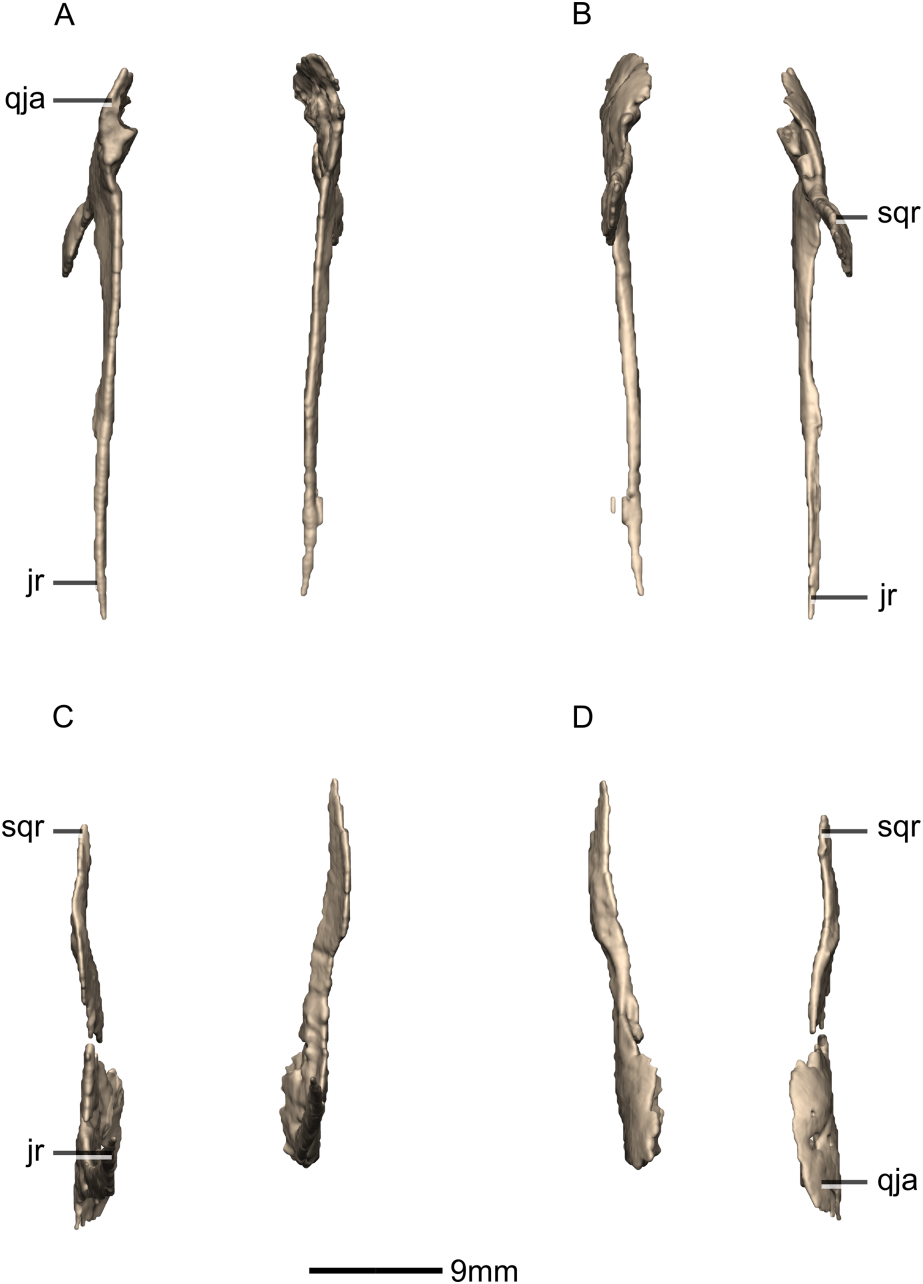
Reconstructed left and right quadratojugals of BP/1/5241. (A) Ventral view. (B) Dorsal view. (C) Anterior view. (D) Posterior view. jr, jugal ramus; qja, quadratojugal angle; sqr, squamosal ramus.

**Figure 21:**
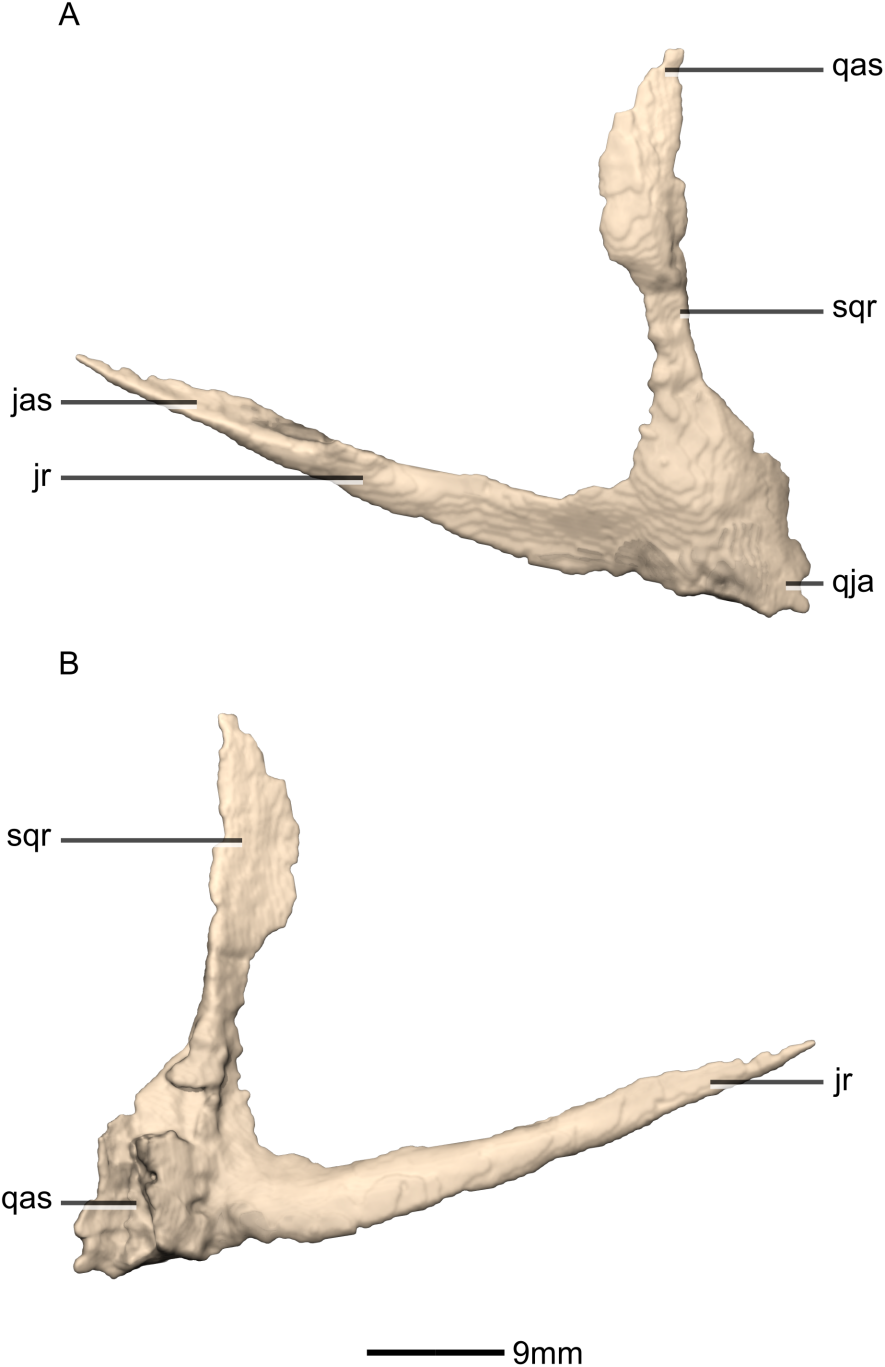
Reconstructed left quadratojugal of BP/1/5241. (A) Left lateral view. (B) Left medial view. jas, jugal articular surface; jr, jugal ramus; qas, quadrate articular surface; qja, quadratojugal angle; sqr, squamosal ramus.

**Figure 22:**
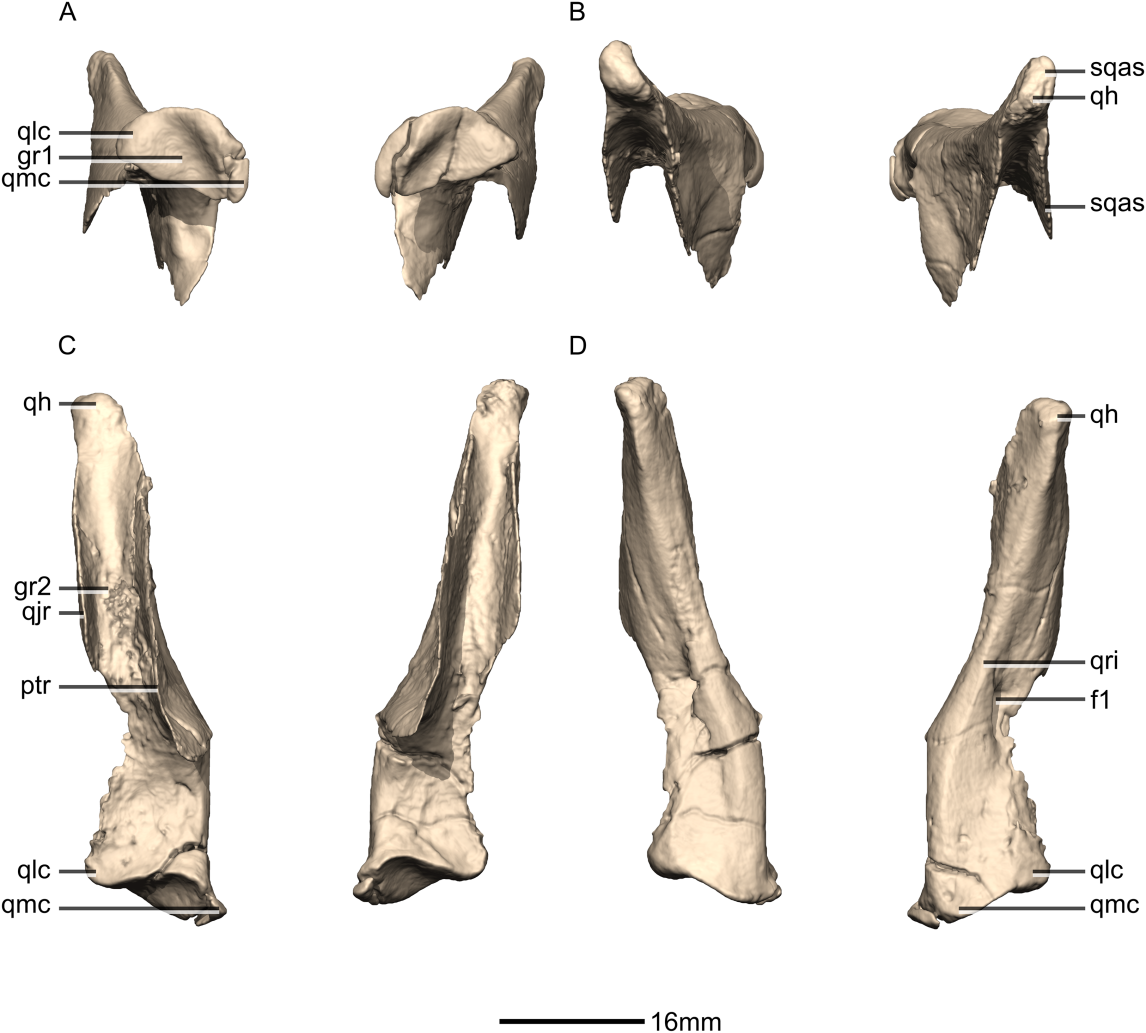
Reconstructed left and right quadrates of BP/1/5241. (A) Ventral view. (B) Dorsal view. (C) Anterior view. (D) Posterior view. f, fossa; gr, groove; ptr, pterygoid ramus; qh, quadrate head; qjr, quadratojugal ramus; qlc, quadrate lateral condyle; qmc, quadrate medial condyle; qri, quadrate ridge; sqas, squamosal articular surface.

**Figure 23:**
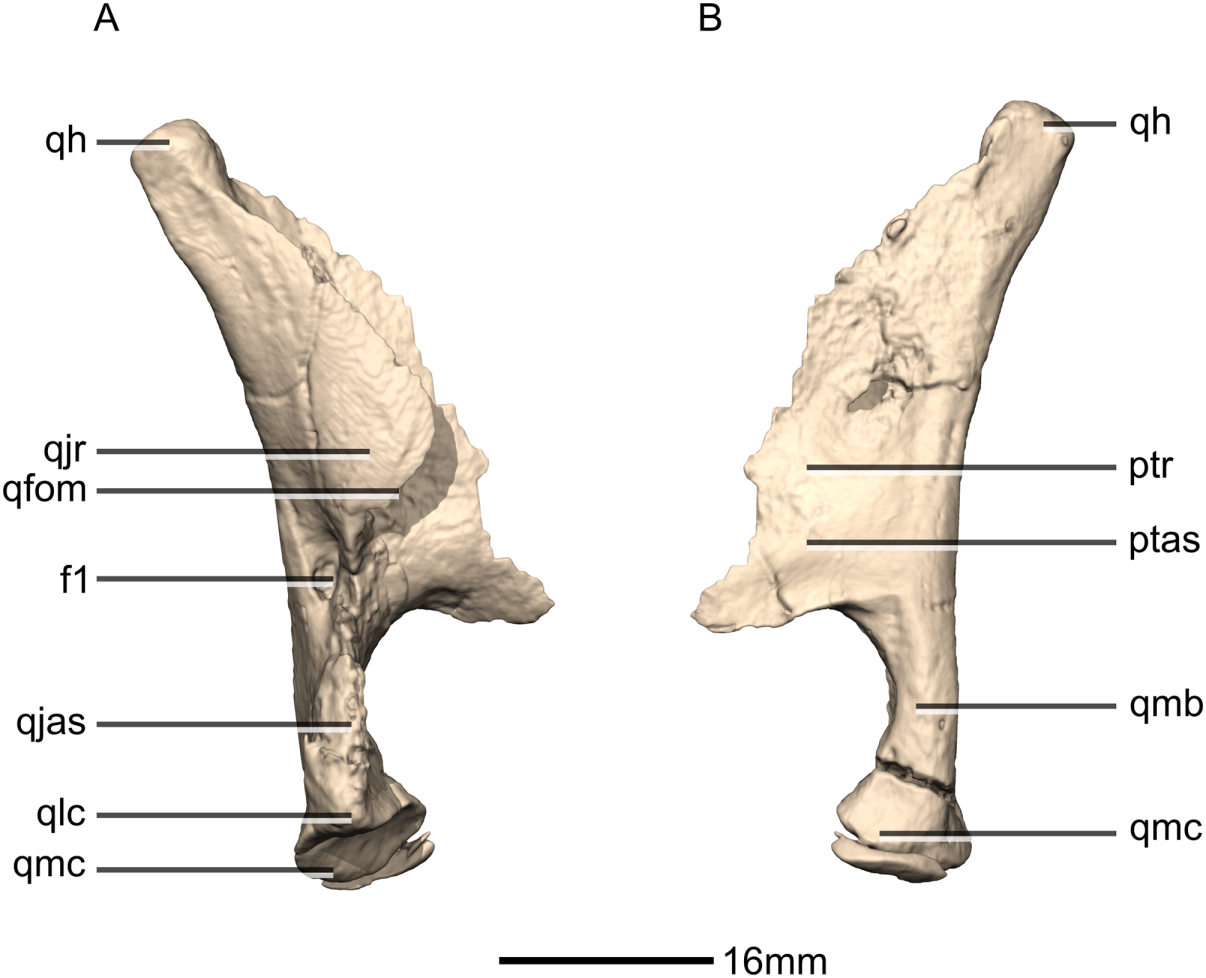
Reconstructed right quadrate of BP/1/5241. (A) Right lateral view. (B) Right medial view. f, fossa; ptas, pterygoid articular surface; ptr, pterygoid ramus; qfom, quadrate foramen margin; qh, quadrate head; qjas, quadratojugal articular surface; qjr, quadratojugal ramus; qlc, quadrate lateral condyle; qmb, quadrate main body; qmc, quadrate medial condyle.

**Figure 24:**
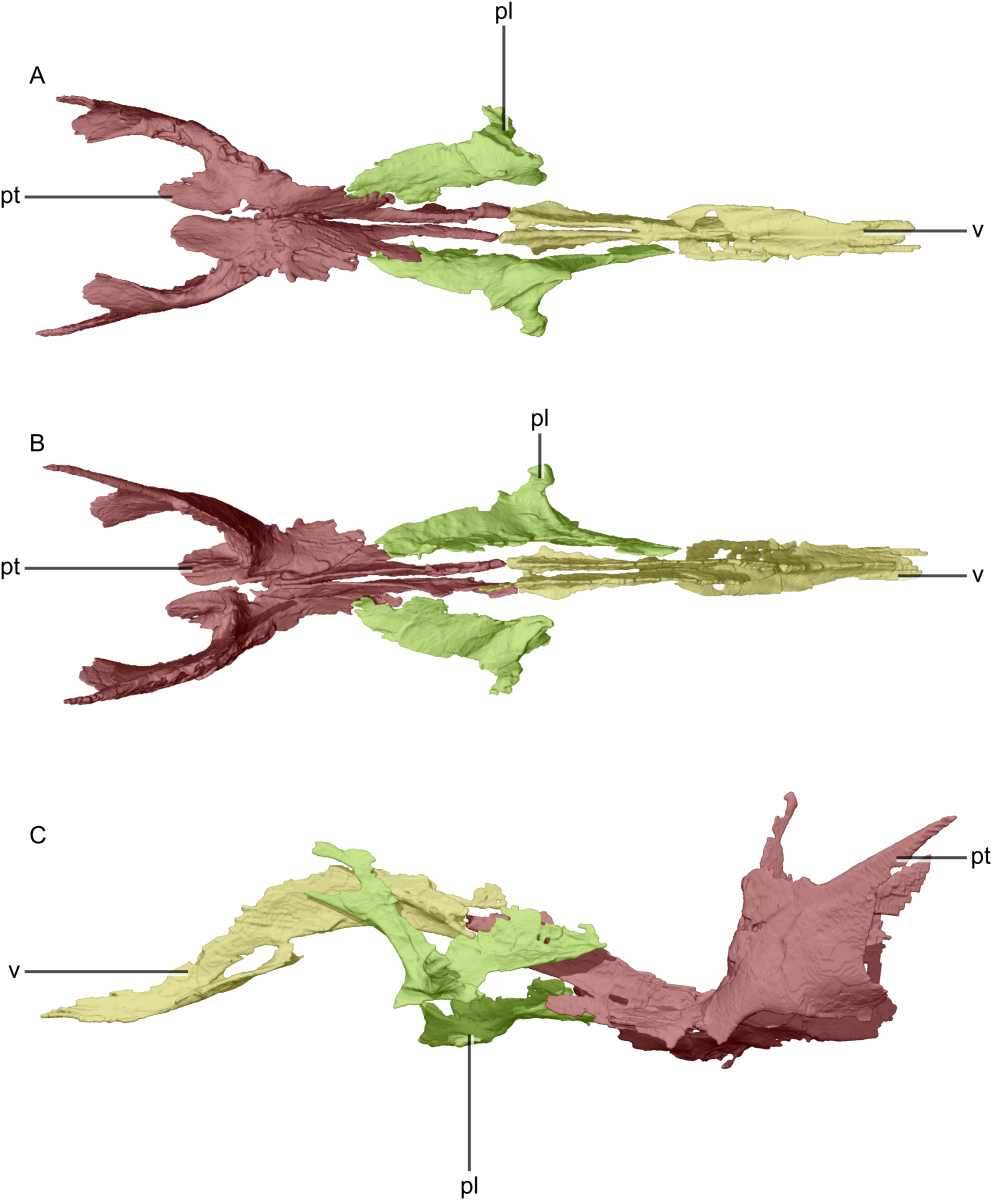
Reconstructed palate of BP/1/5241. (A) Ventral view. (B) Dorsal view. (C) Left lateral view. pl, palatine; pt, pterygoid; v, vomer.

**Figure 25:**
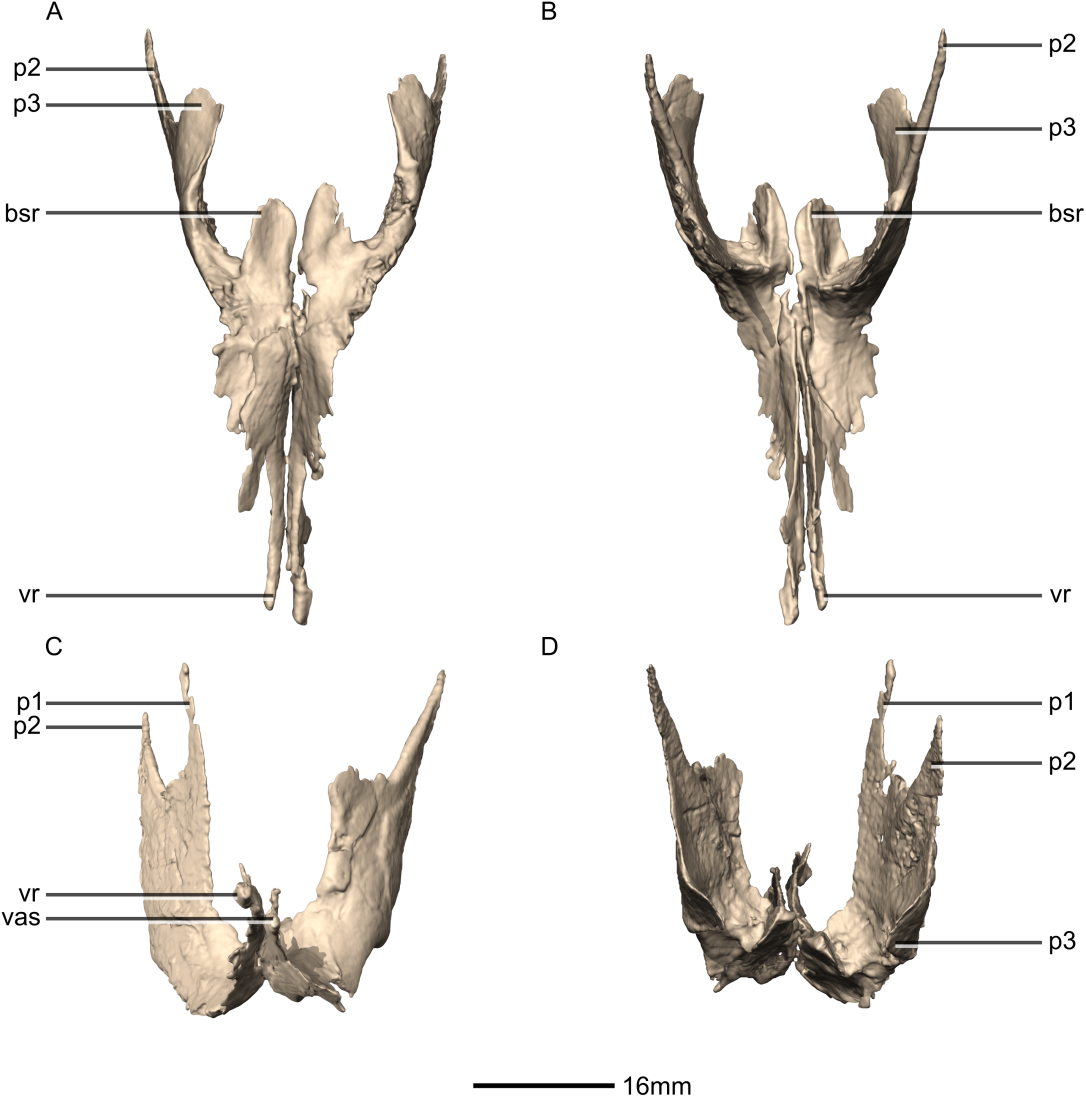
Reconstructed left and right pterygoids of BP/1/5241. (A) Ventral view. (B) Dorsal view. (C) Anterior view. (D) Posterior view. bsr, basisphenoid ramus; p, process; vas, vomer articular surface; vr, vomerine ramus.

**Figure 26:**
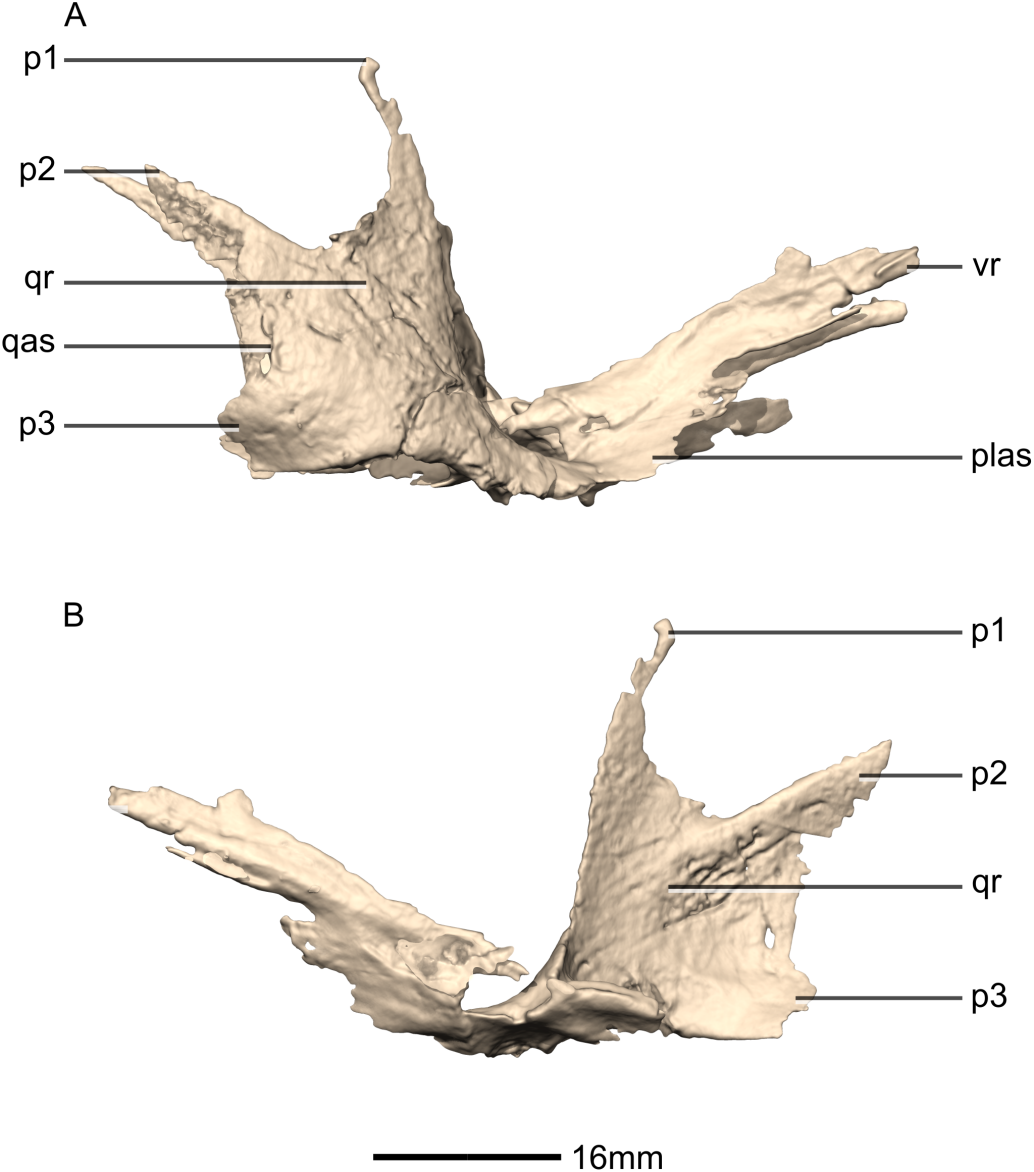
Reconstructed right pterygoid of BP/1/5241. (A) Right lateral view. (B) Right medial view. p, process; plas, palate articular surface; qas, quadrate articular surface; qr, quadrate ramus; vr, vomerine ramus.

**Figure 27:**
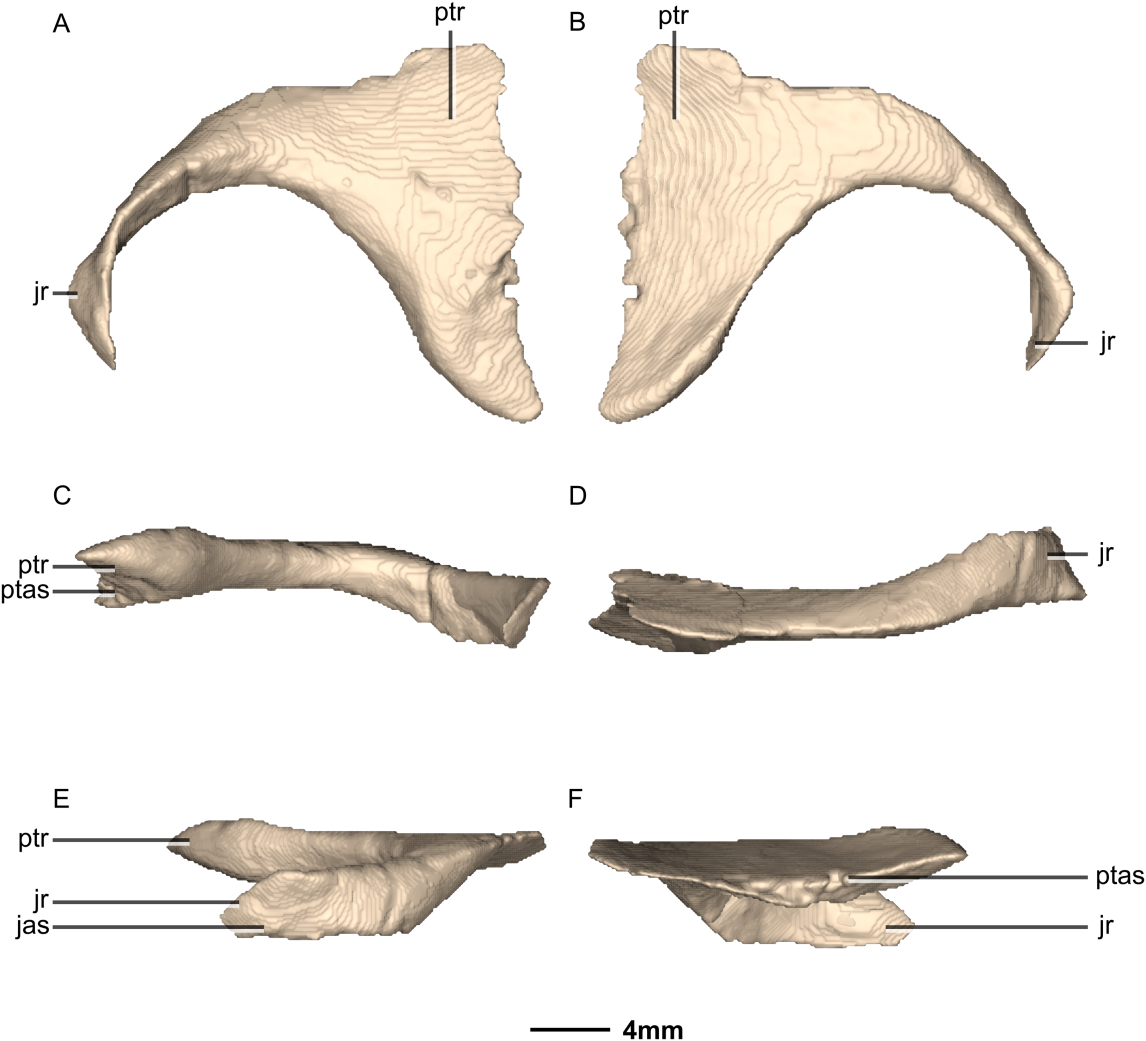
Reconstructed left ectopterygoid of BP/1/5241. (A) Ventral view. (B) Dorsal view. (C) Posterior view. (D) Anterior view. (E) Left lateral view. (F) Left medial view. jas, jugal articular surface; jr, jugal ramus; ptas, pterygoid articular surface; ptr, pterygoid ramus.

**Figure 28:**
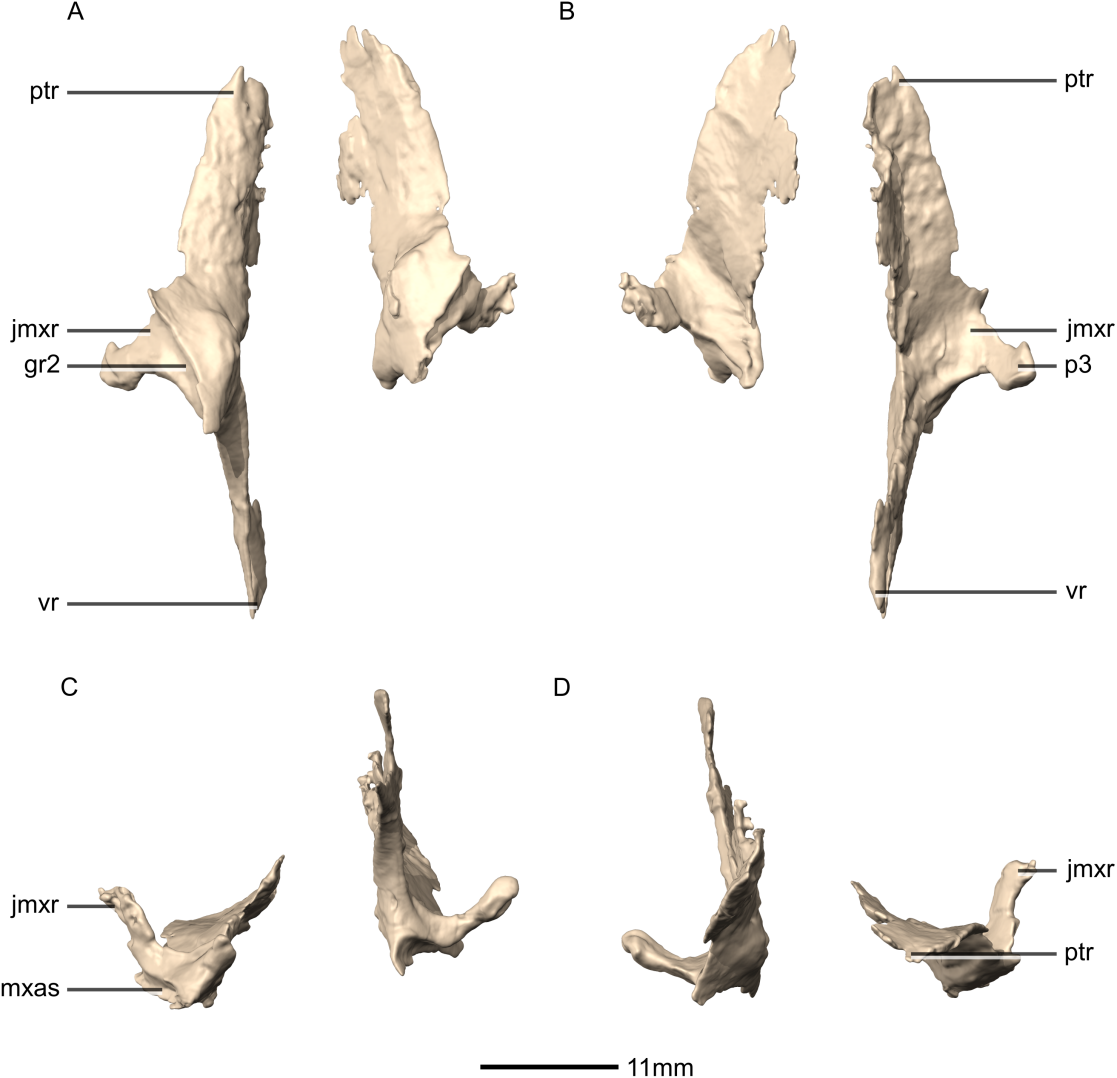
Reconstructed left and right palatines of BP/1/5241. (A) Ventral view. (B) Dorsal view. (C) Anterior view. (D) Posterior view. gr, groove; jmxr, jugomaxillary ramus; mxas, maxilla articular surface; p, process; ptr, pterygoid ramus; vr, vomerine ramus.

**Figure 29:**
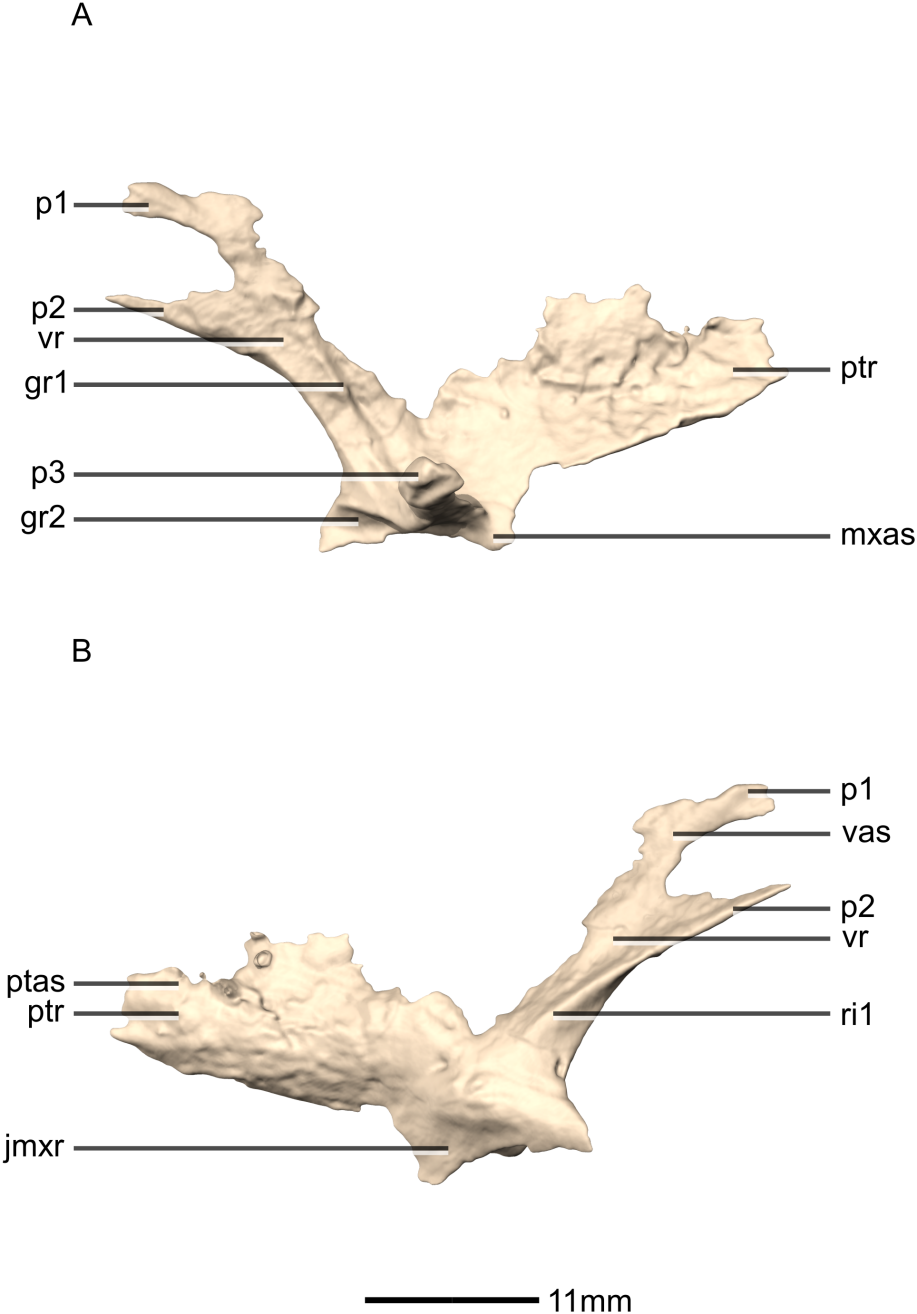
Reconstructed left palatine of BP/1/5241. (A) Left lateral view. (B) Left medial view. gr, groove; jmxr, jugomaxillary ramus; mxas, maxilla articular surface; p, process; ptas, pterygoid articular surface; ptr, pterygoid ramus; ri, ridge; vas, vomer articular surface; vr, vomerine ramus.

**Figure 30:**
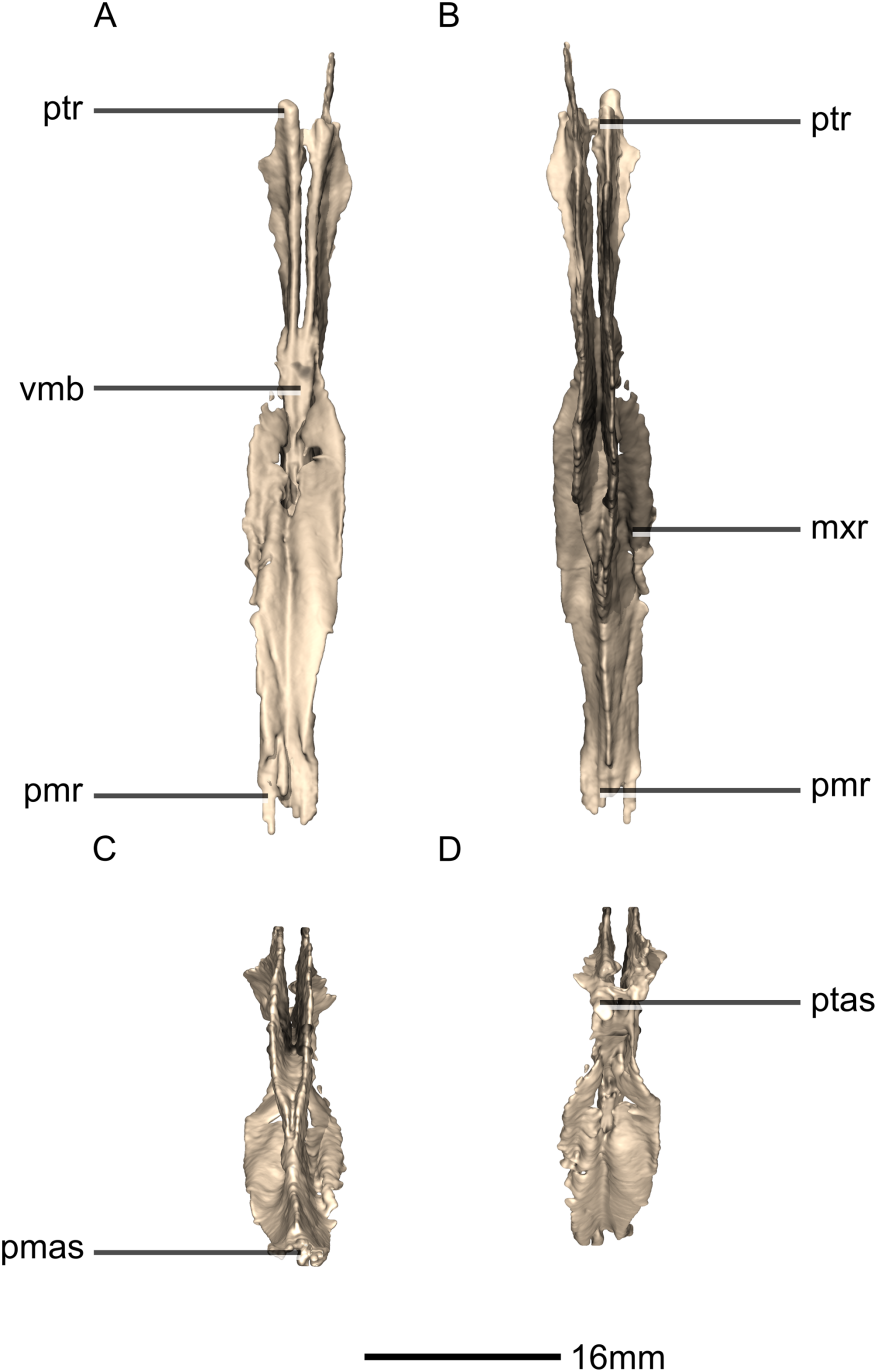
Reconstructed fused left and right vomers of BP/1/5241. (A) Ventral view. (B) Dorsal view. (C) Anterior view. (D) Posterior view. mxr, maxillary ramus; pmas, premaxilla articular surface; pmr, premaxilla ramus; ptas, pterygoid articular surface; ptr, pterygoid ramus; vmb, vomer main body.

**Figure 31:**
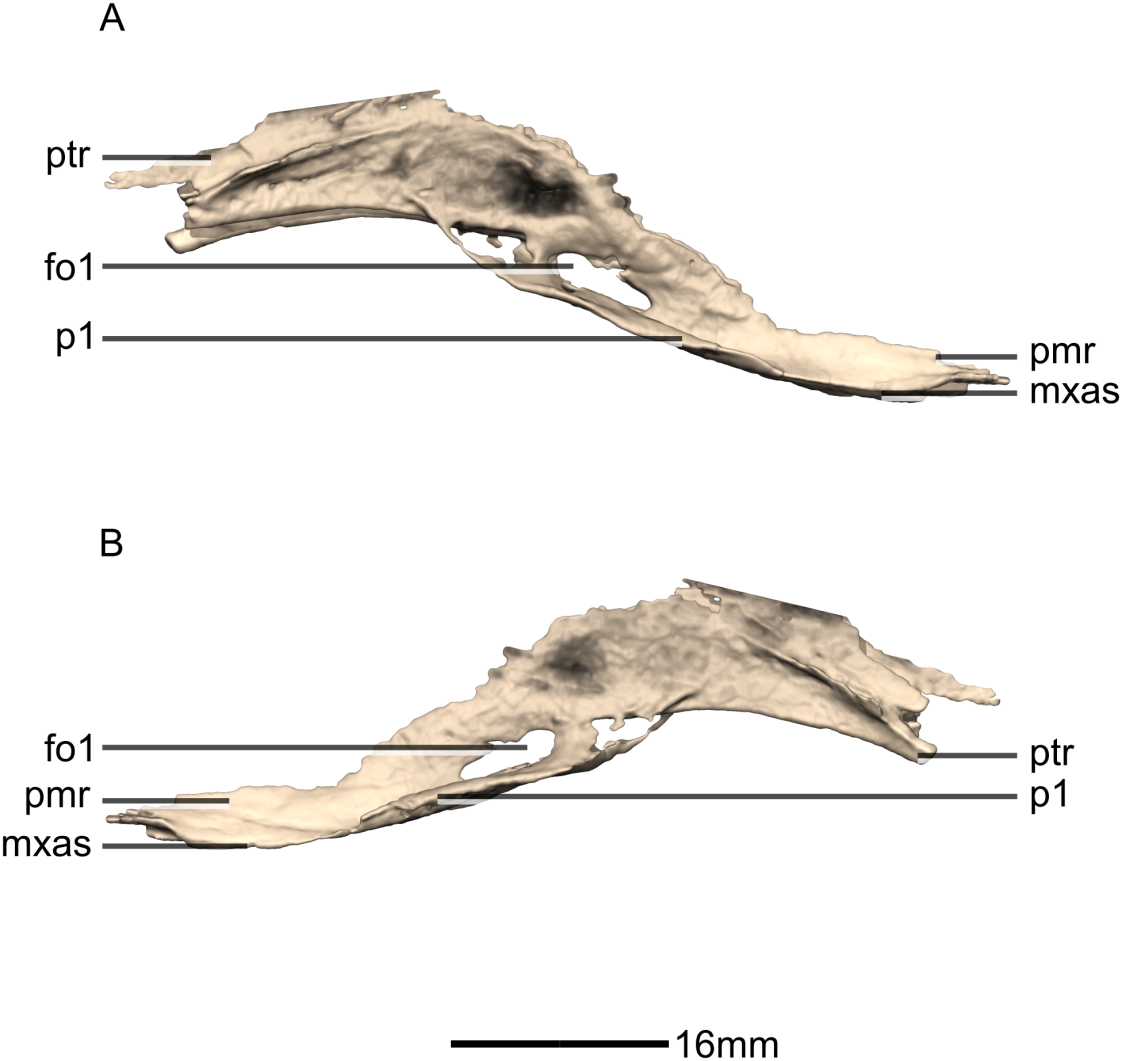
Reconstructed fused left and right vomers of BP/1/5241. (A) Right lateral view. (B) Left lateral view. fo, foramen; mxas, maxillary articular surface; pmr, premaxilla ramus; p, process; ptr, pterygoid ramus.

**Figure 32:**
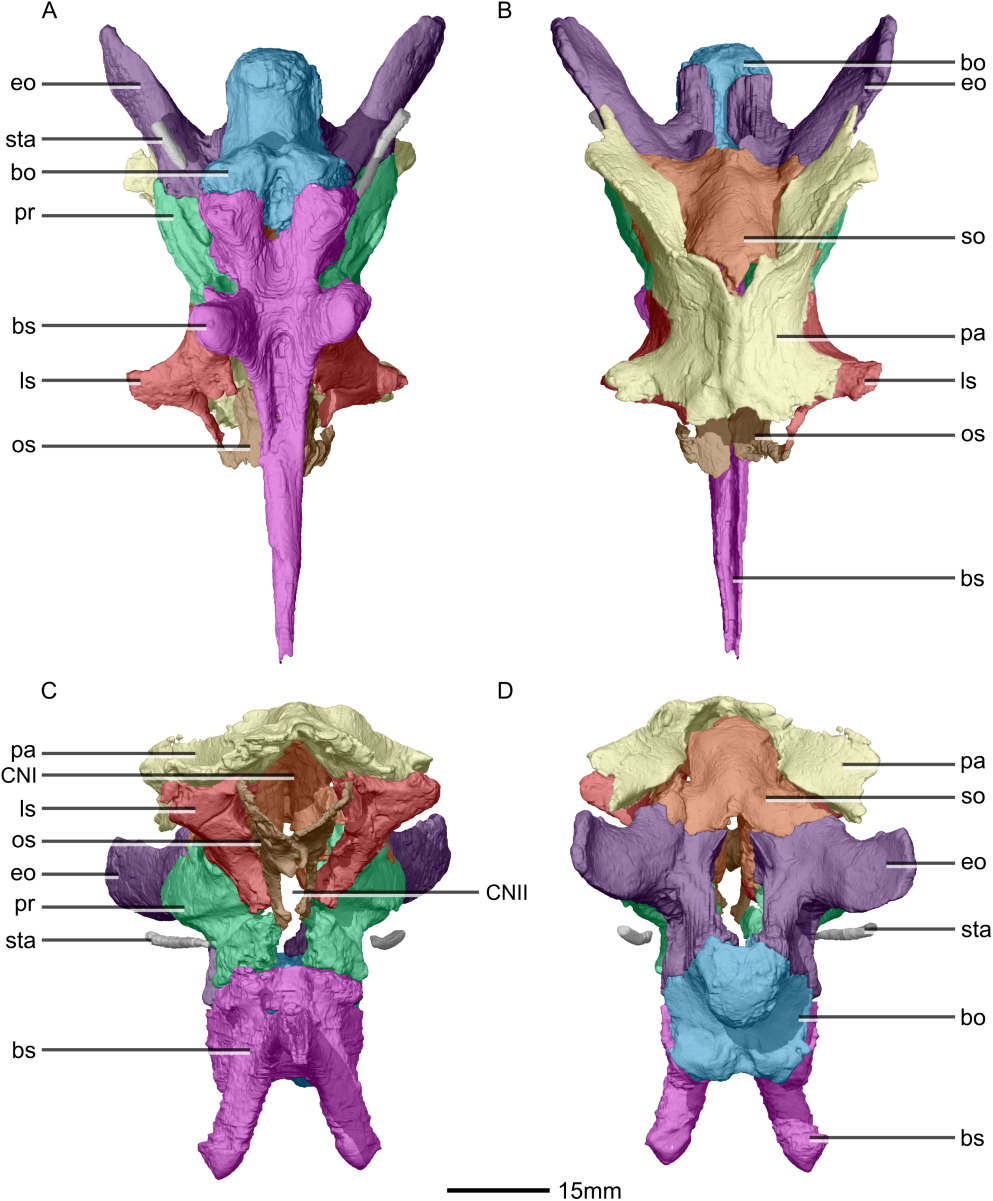
Reconstructed braincase of BP/1/5241 (excluding the frontal for better visualization of internal braincase morphologies not visible when studying the specimen externally). (A) Ventral view. (B) Dorsal view. (C) Anterior view. (D) Posterior view. bo, basioccipital; bs, basisphenoid; CN, cranial nerve passage; eo, exoccipital; ls, laterosphenoid; os, orbitosphenoid; pa, parietal; pr, prootic; so, supraoccipital; sta, stapes.

**Figure 33:**
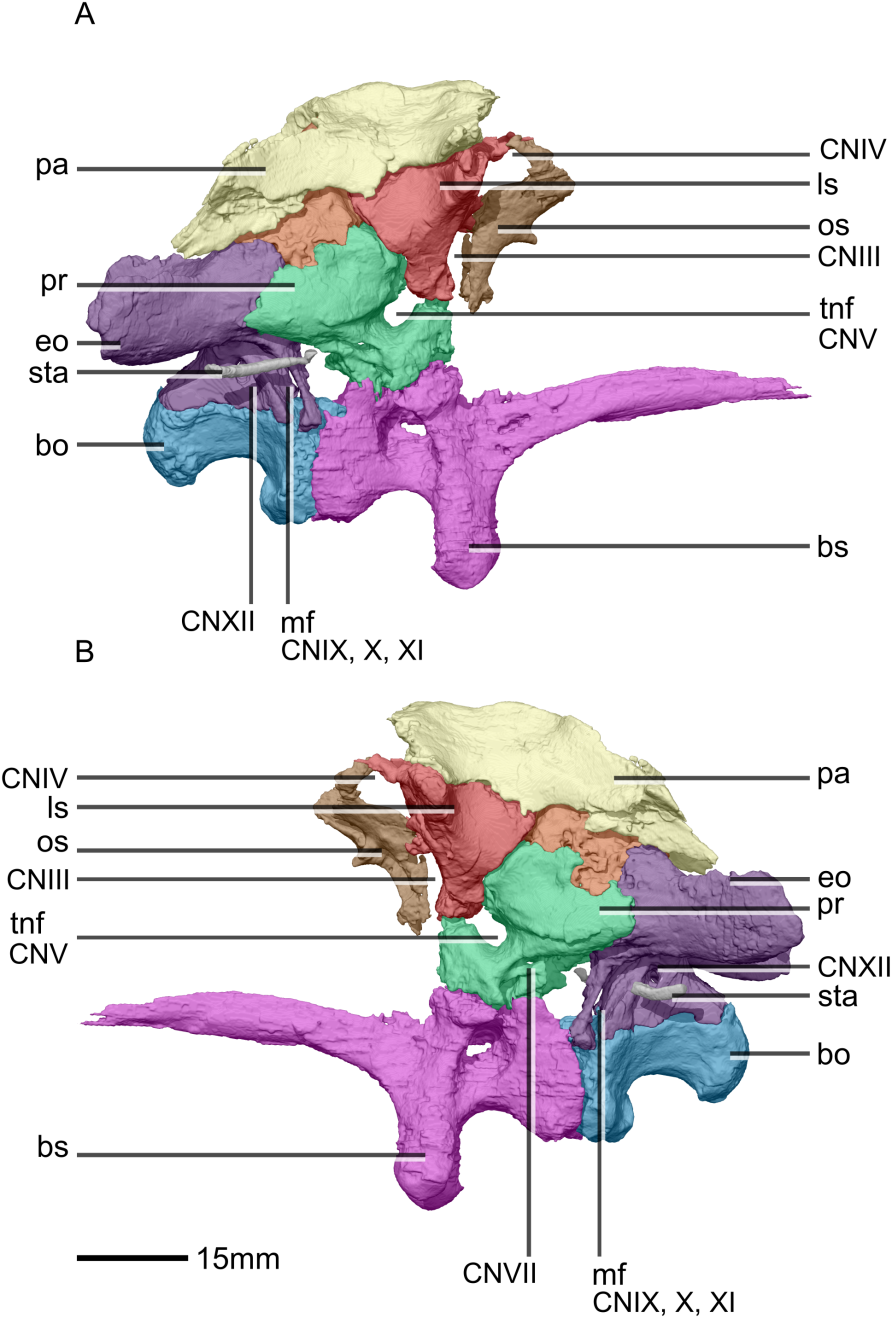
Reconstructed braincase of BP/1/5241 (excluding the frontal for better visualization of internal braincase morphologies not visible when studying the specimen externally). (A) Right lateral view. (B) Left lateral view. bo, basioccipital; bs, basisphenoid; CN, cranial nerve passage; eo, exoccipital; ls, laterosphenoid; mf, metotic fissure; os, orbitosphenoid; pa, parietal; pr, prootic; sta, stapes; tnf, trigeminal nerve foramen.

**Figure 34:**
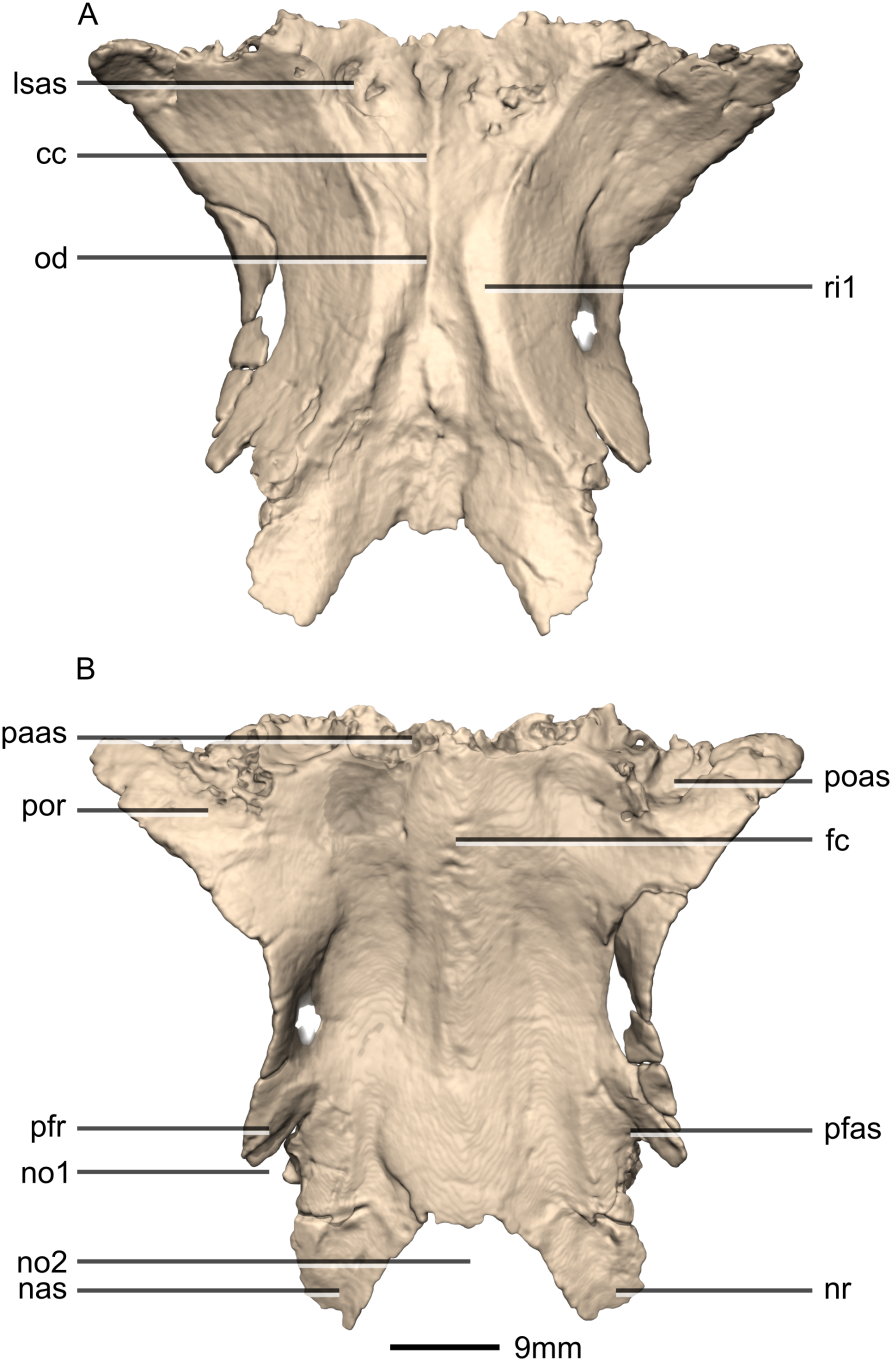
Reconstructed fused left and right frontals of BP/1/5241. (A) Ventral view. (B) Dorsal view. cc, cerebral cavity; fc, frontal crest; lsas, laterosphenoid articular surface; nas, nasal articular surface; no, notch; nr, nasal ramus; od, olfactory depression; paas, parietal articular surface; pfas, prefrontal articular surface; pfr, prefrontal ramus; poas, postorbital articular surface; por, postorbital ramus; ri, ridge.

**Figure 35:**
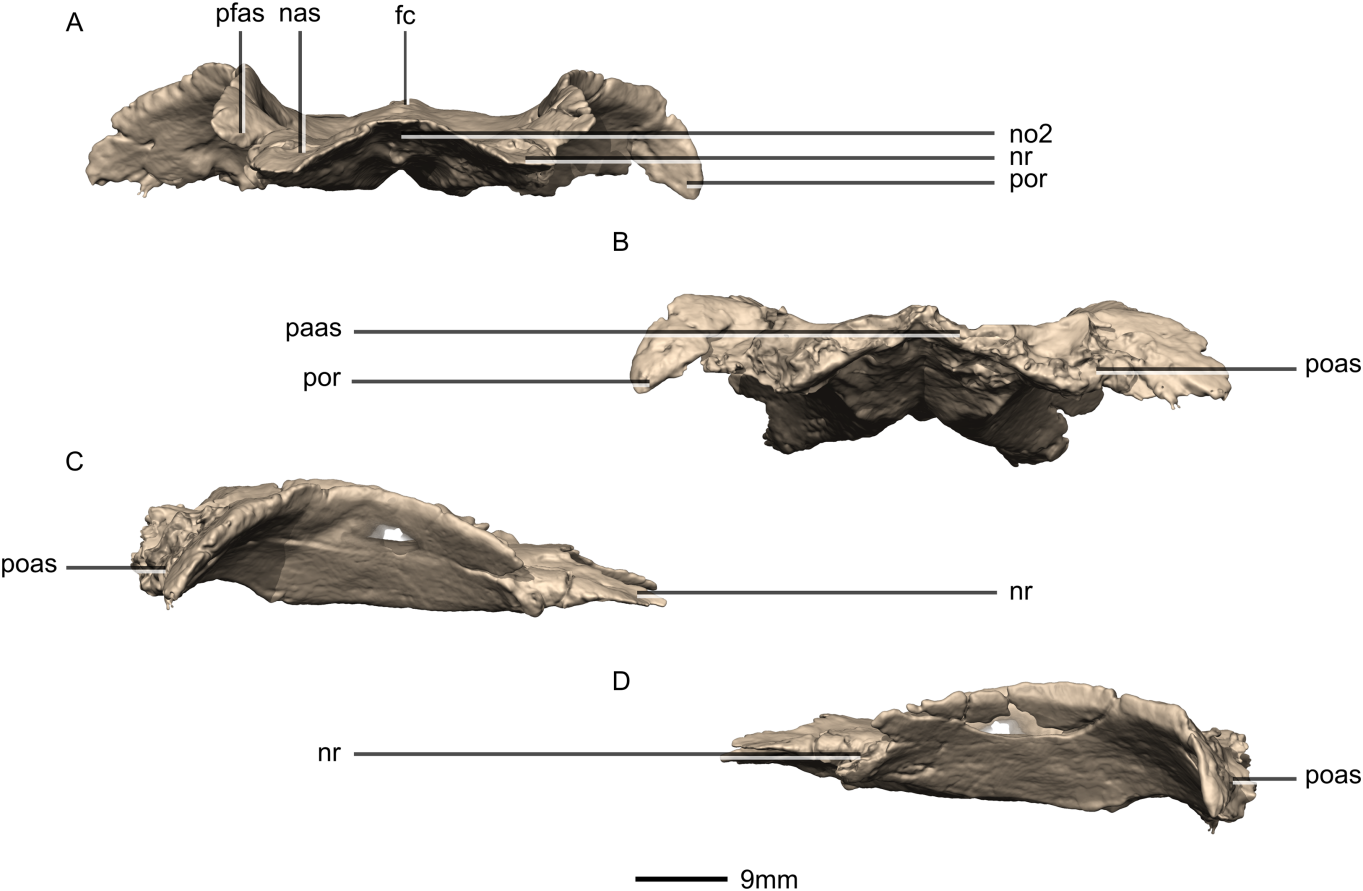
Reconstructed fused left and right frontals of BP/1/5241. (A) Anterior view. (B) Posterior view. (C) Right lateral view. (D) Left lateral view. fc, frontal crest; nas, nasal articular surface; no, notch; nr, nasal ramus; paas, parietal articular surface; pfas, prefrontal articular surface; poas, postorbital articular surface; por, postorbital ramus.

**Figure 36:**
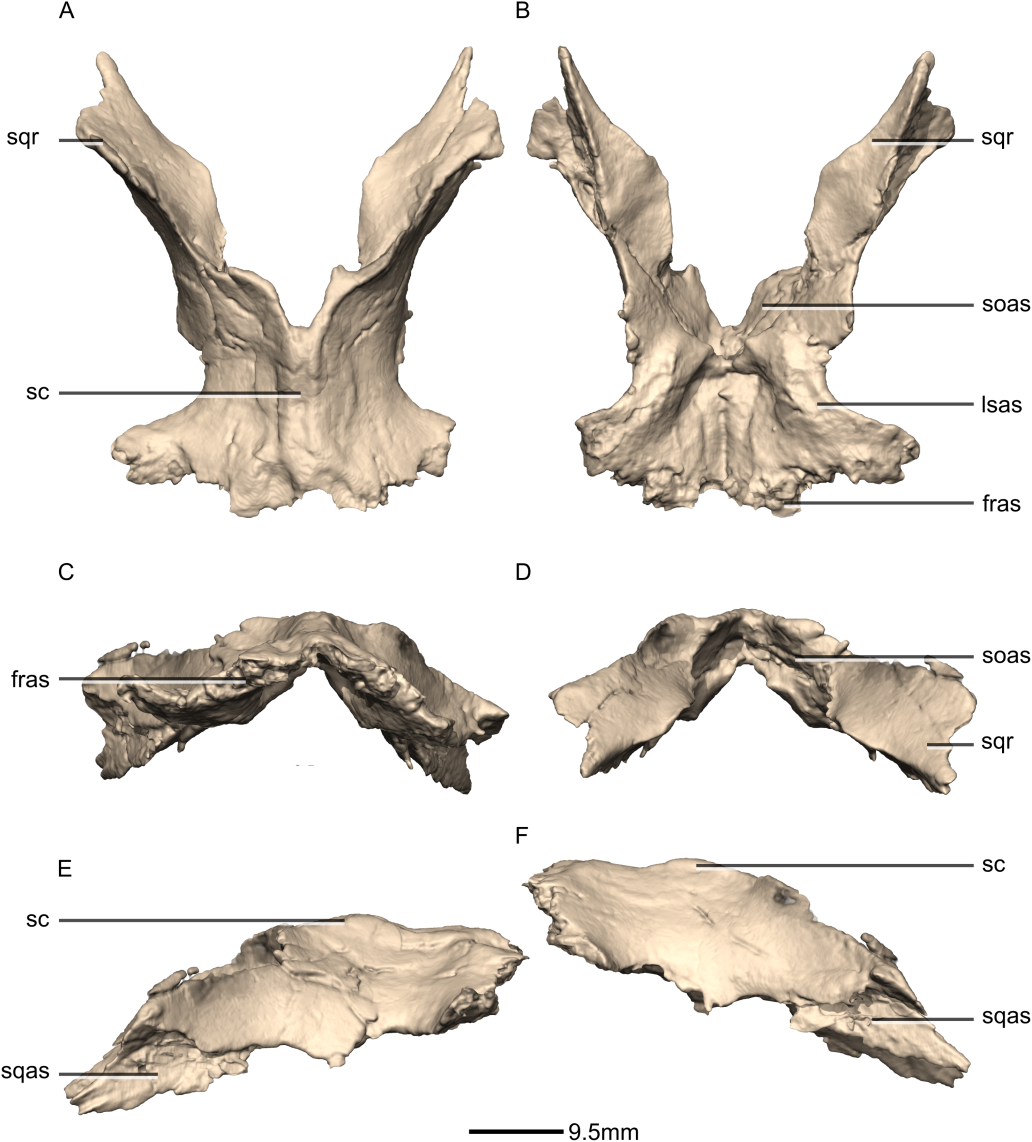
Reconstructed fused left and right parietals of BP/1/5241. (A) Dorsal view. (B) Ventral view. (C) Anterior view. (D) Posterior view. (E) Right lateral view. (F) Left lateral view. fras, frontal articular surface; lsas, laterosphenoid articular surface; sc, sagittal crest; soas, supraoccipital articular surface; sqas, squamosal articular surface; sqr, squamosal ramus.

**Figure 37:**
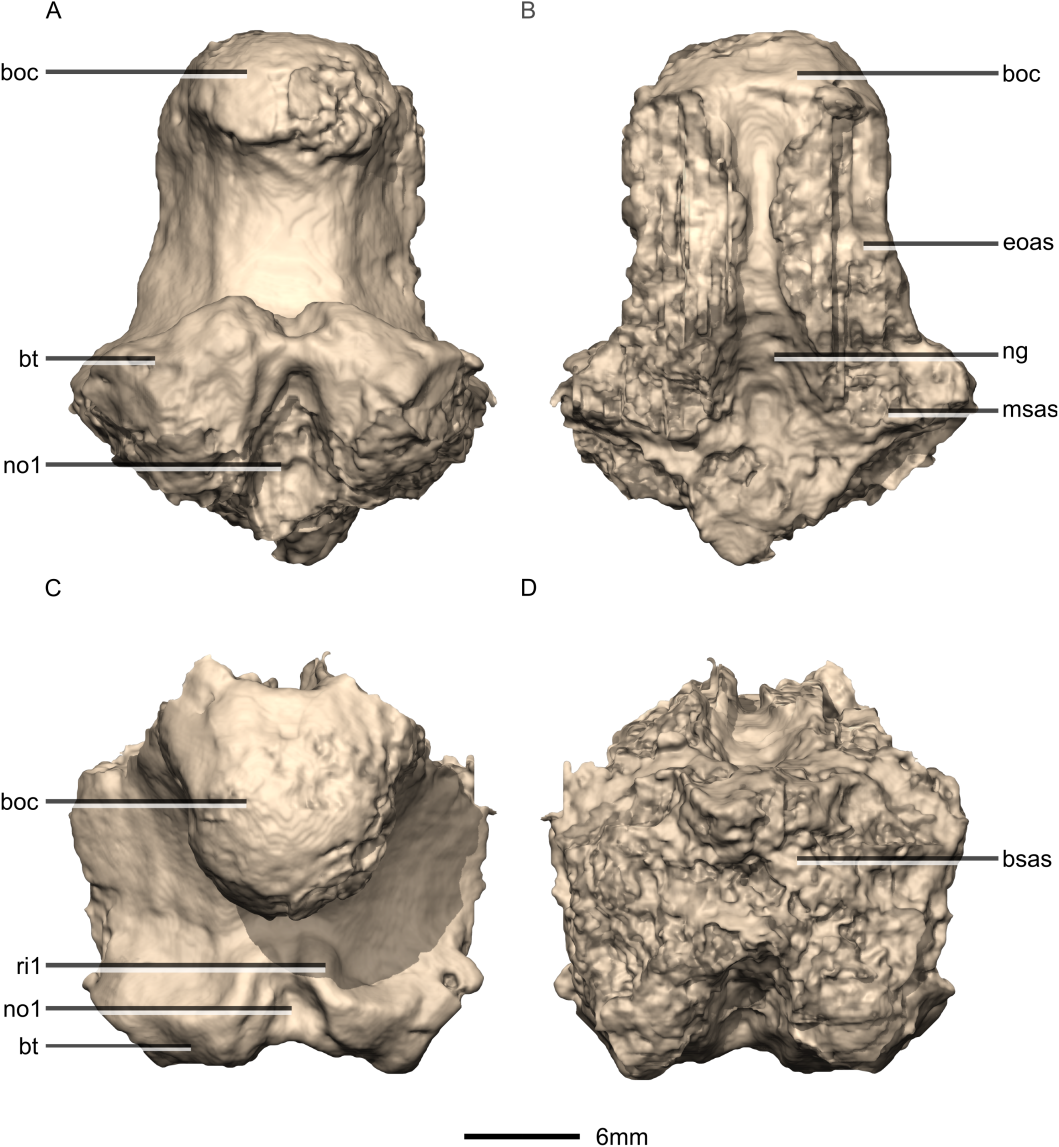
Reconstructed basioccipital of BP/1/5241. (A) Ventral view. (B) Dorsal view. (C) Posterior view. (D) Anterior view. boc, basioccipital condyle; bsas, basisphenoid articular surface; bt, basal tubera; eoas, exoccipital articular surface; msas, metotic strut articular surface; ng, neural groove; no, notch; ri, ridge.

**Figure 38:**
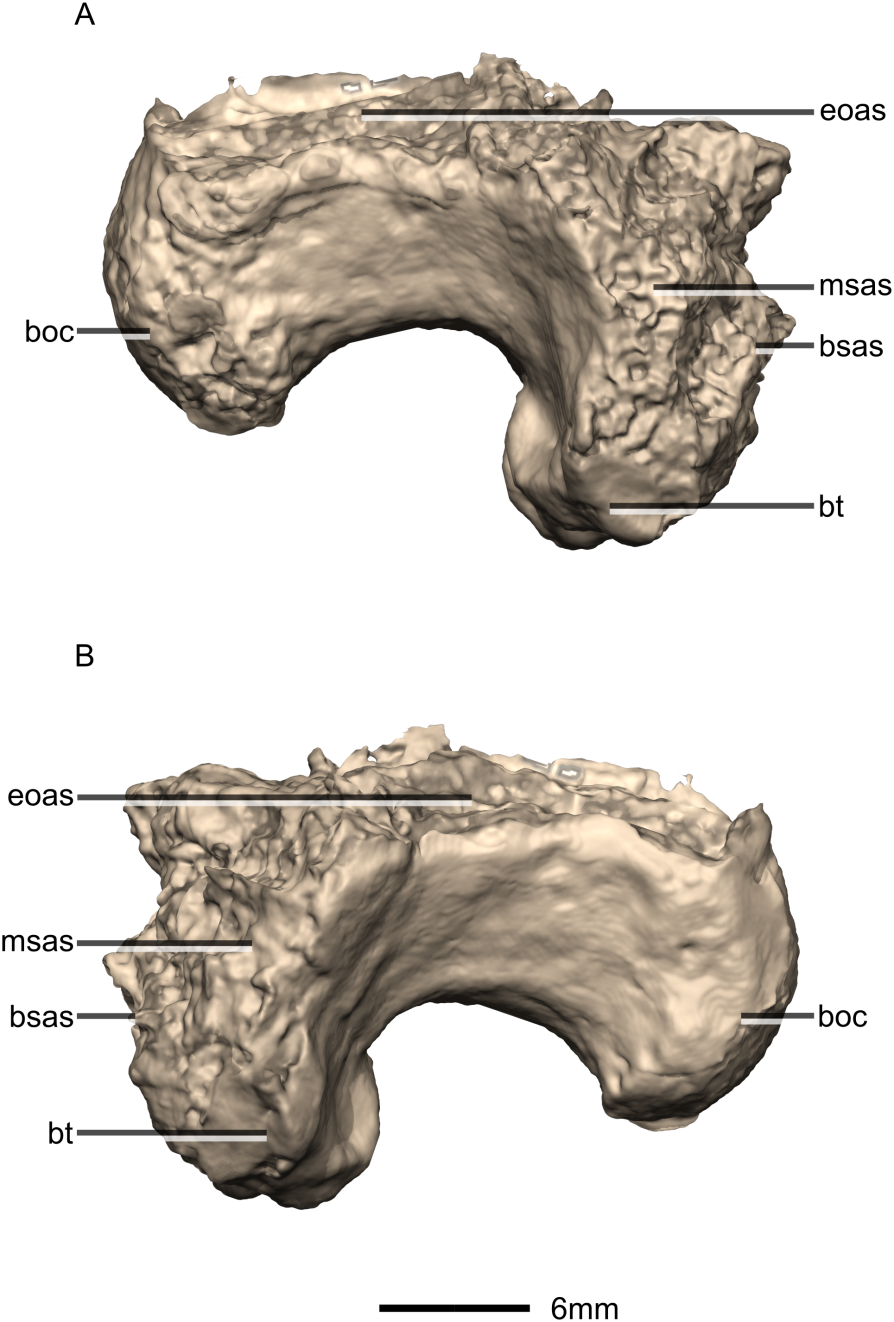
Reconstructed basioccipital of BP/1/5241. (A) Right lateral view. (B) Left lateral view. boc, basioccipital condyle; bsas, basisphenoid articular surface; bt, basal tubera; eoas, exoccipital articular surface; msas, metotic strut articular surface.

**Figure 39:**
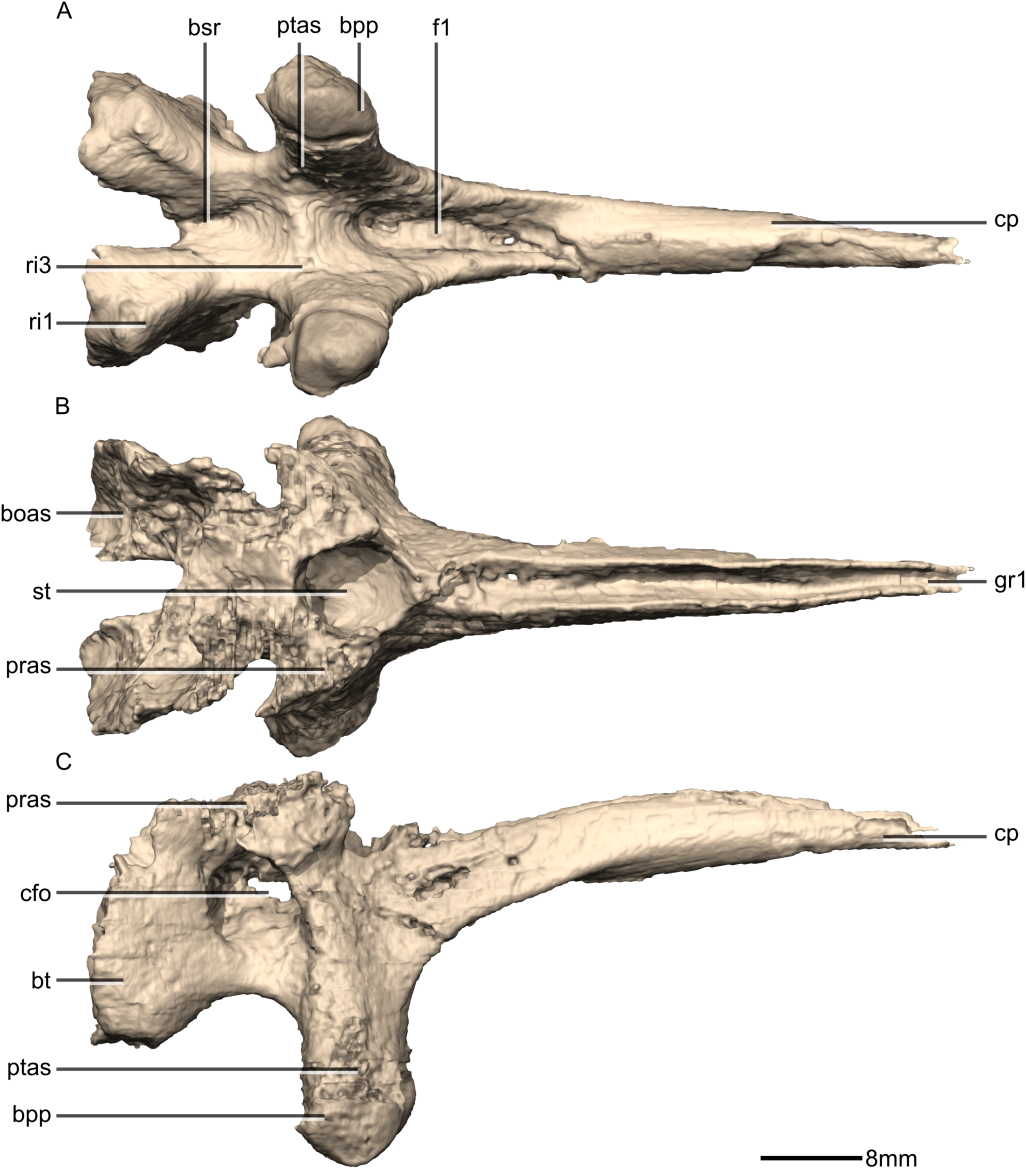
Reconstructed basisphenoid/parasphenoid of BP/1/5241. (A) Ventral view. (B) Dorsal view. (C) Right lateral view. boas, basioccipital articular surface; bpp, basipterygoid processes; bsr, basisphenoid recess; bt, basal tubera; cfo, carotid foramen; cp, cultriform process; f, fossa; gr, groove; pras, prootic articular surface; ptas, pterygoid articular surface; ri, ridge; st, sella turcica.

**Figure 40:**
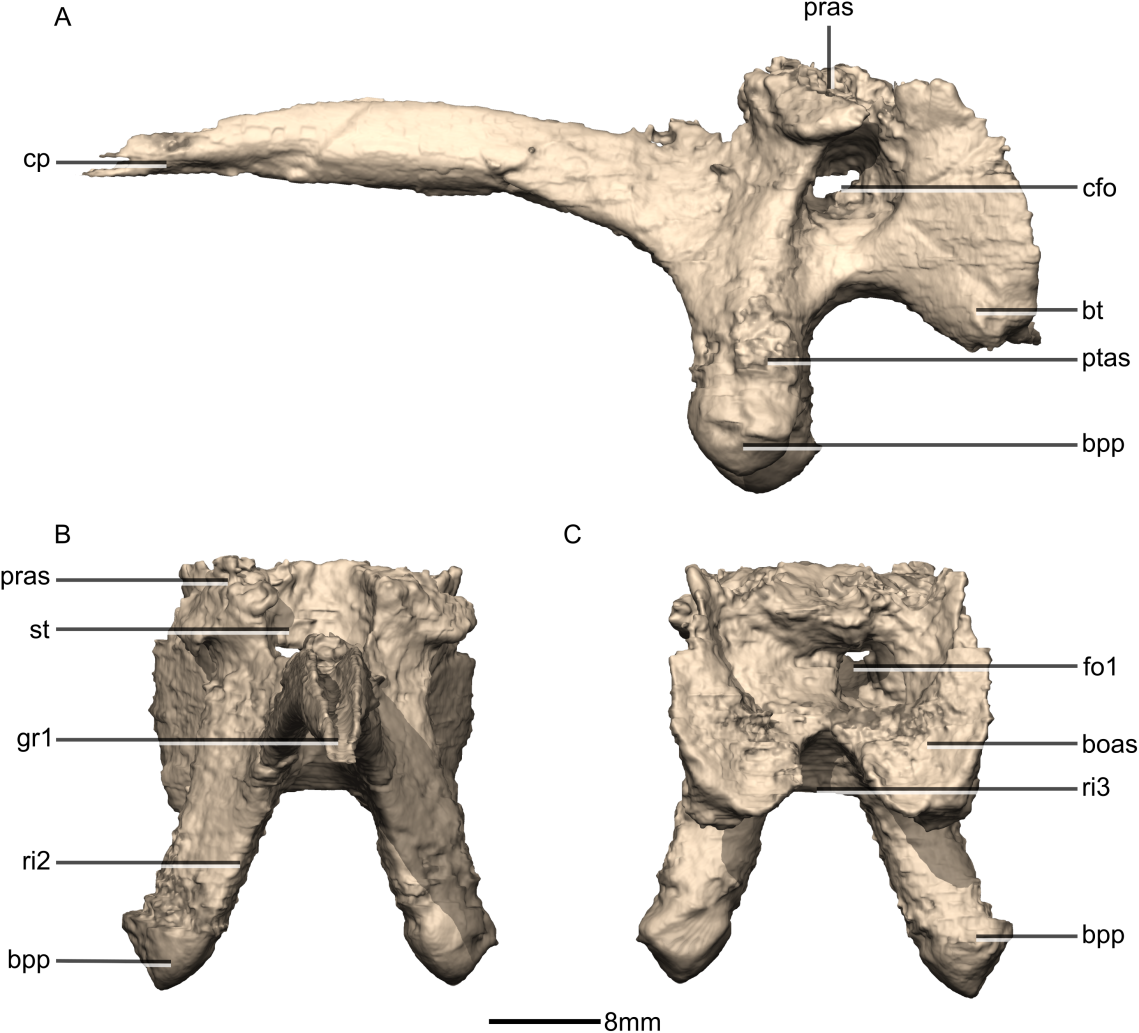
Reconstructed basisphenoid/parasphenoid of BP/1/5241. (A) Left lateral view. (B) Anterior view. (C) Posterior view. boas, basioccipital articular surface; bpp, basipterygoid processes; bt, basal tubera; cfo, carotid foramen; cp, cultriform process; fo, foramen; gr, groove; pras, prootic articular surface; ptas, pterygoid articular surface; ri, ridge; st, sella turcica.

**Figure 41:**
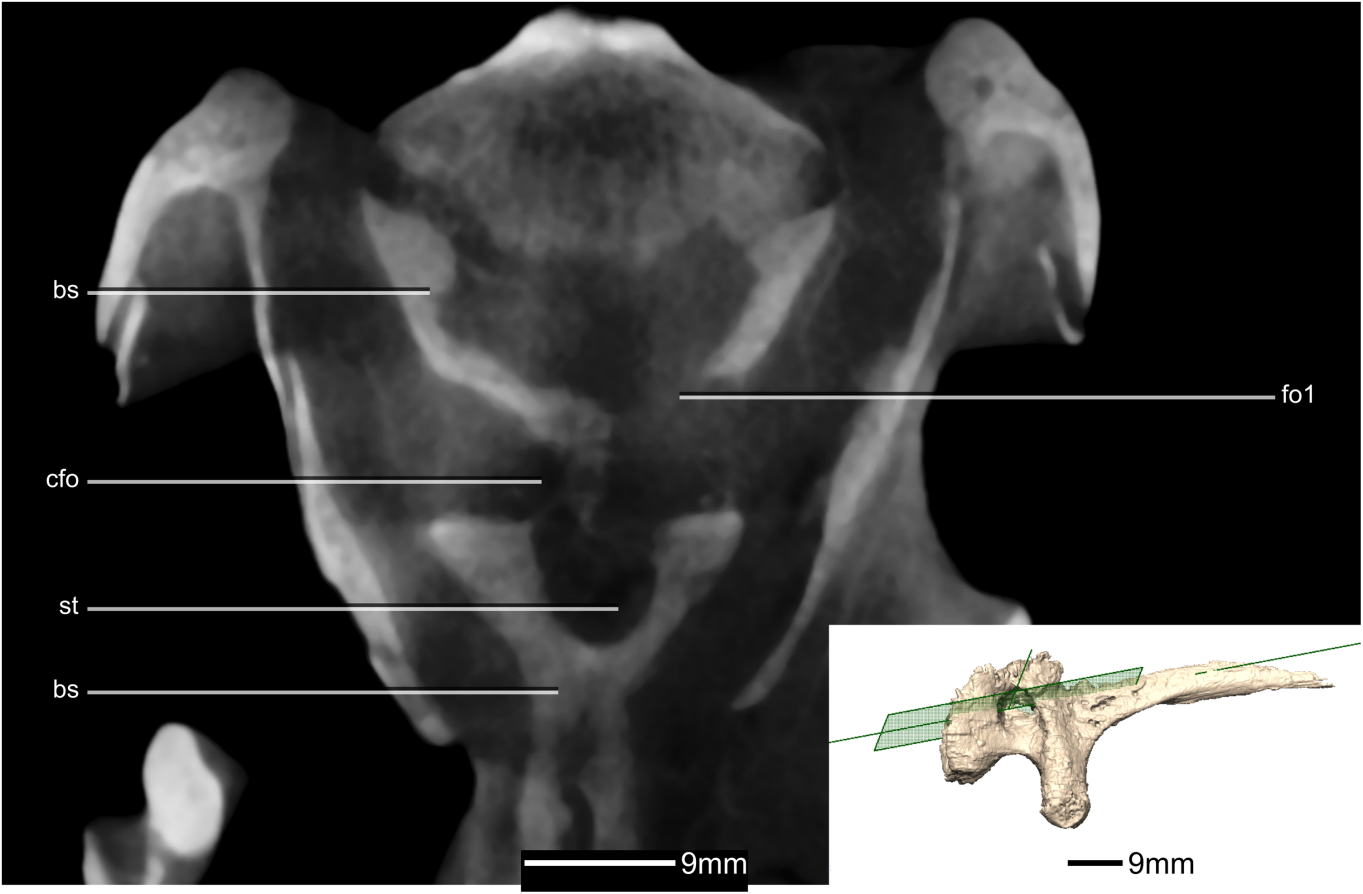
Cross section of the basisphenoid showing pneumatic pockets. Inset is the exact location of the slice. bs, basisphenoid; cfo, carotid foramen; fo, foramen; gr, groove; st, sella turcica.

**Figure 42:**
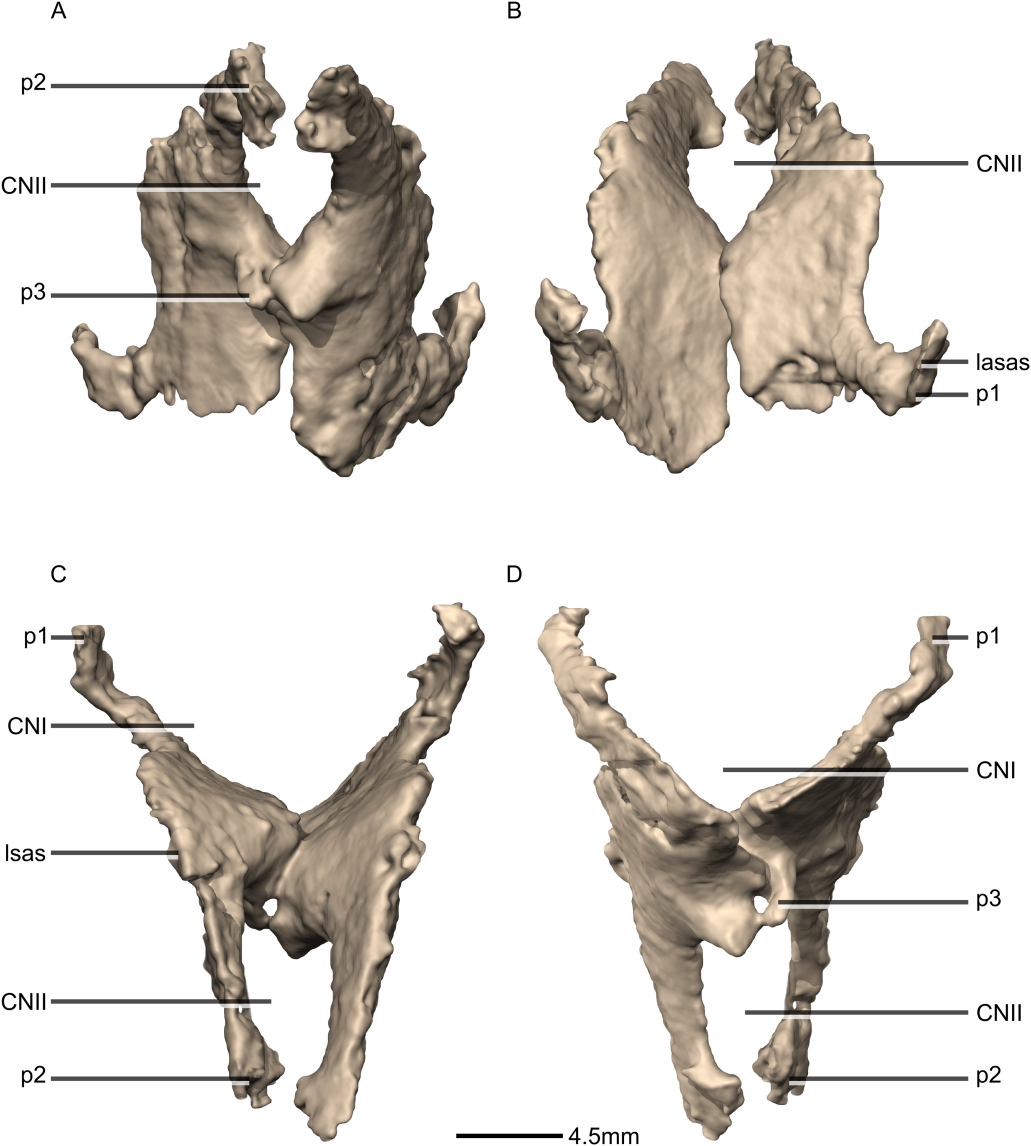
Reconstructed fused left and right orbitosphenoids of BP/1/5241. (A) Ventral view. (B) Dorsal view. (C) Posterior view. (D) Anterior view. CN, cranial nerve passage; lasas, laterosphenoid articular surface; p, process.

**Figure 43:**
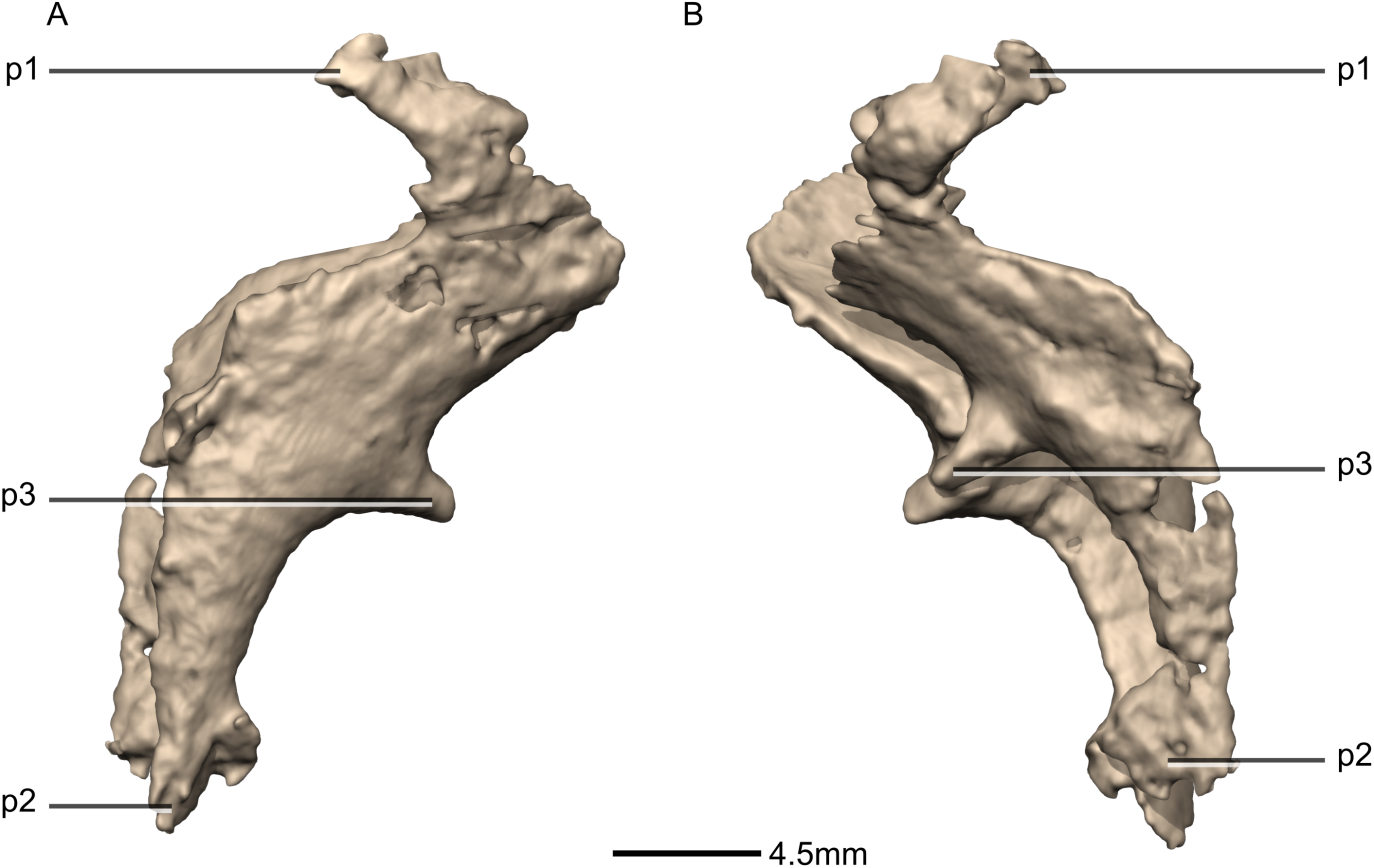
Reconstructed fused left and right orbitosphenoids of BP/1/5241. (A) Right lateral view. (B) Left lateral view. p, process.

**Figure 44:**
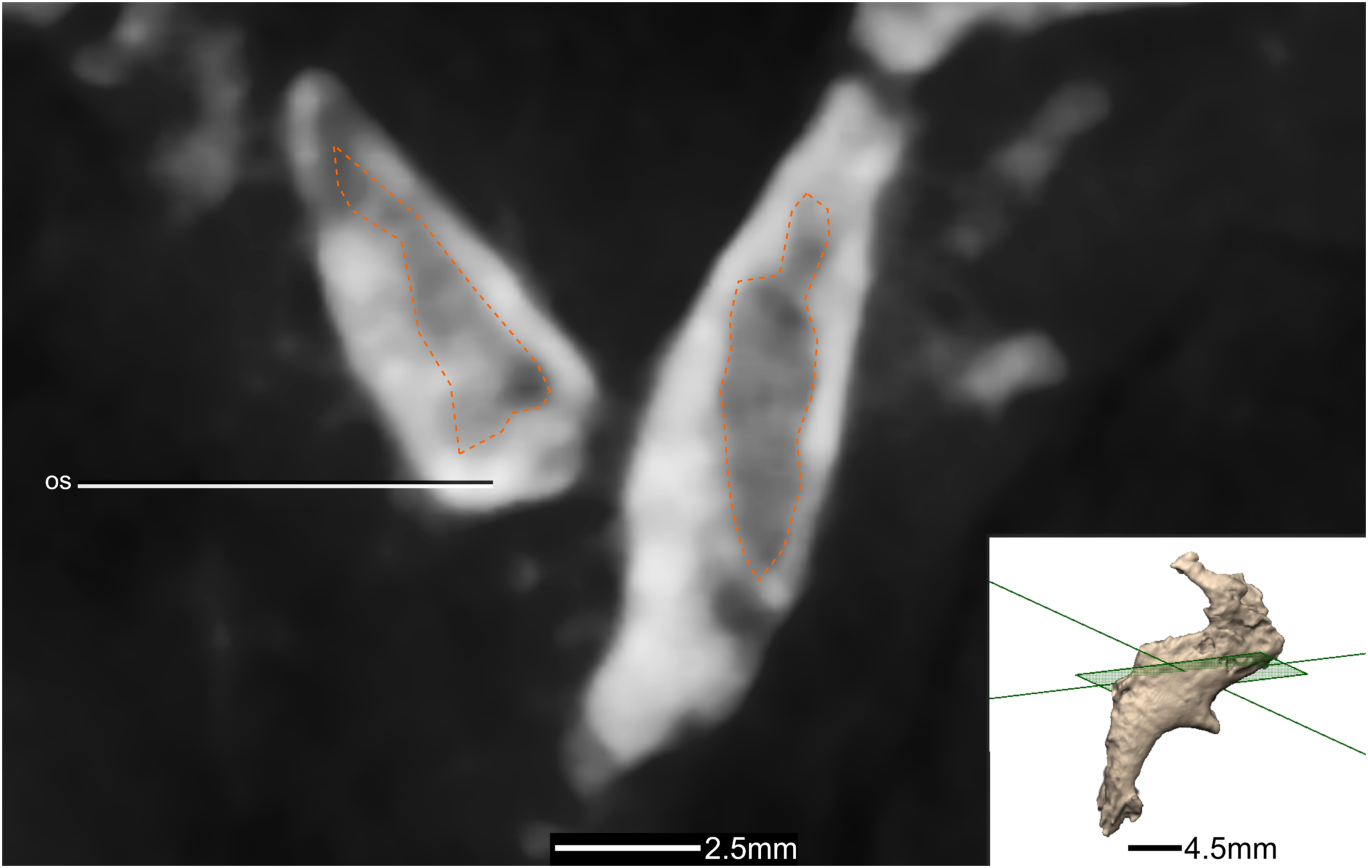
Cross section of the orbitosphenoids showing hollow internal structure. Outline of the hollowed-out section in orange. Inset is the exact location of the slice. os, orbitosphenoid.

**Figure 45:**
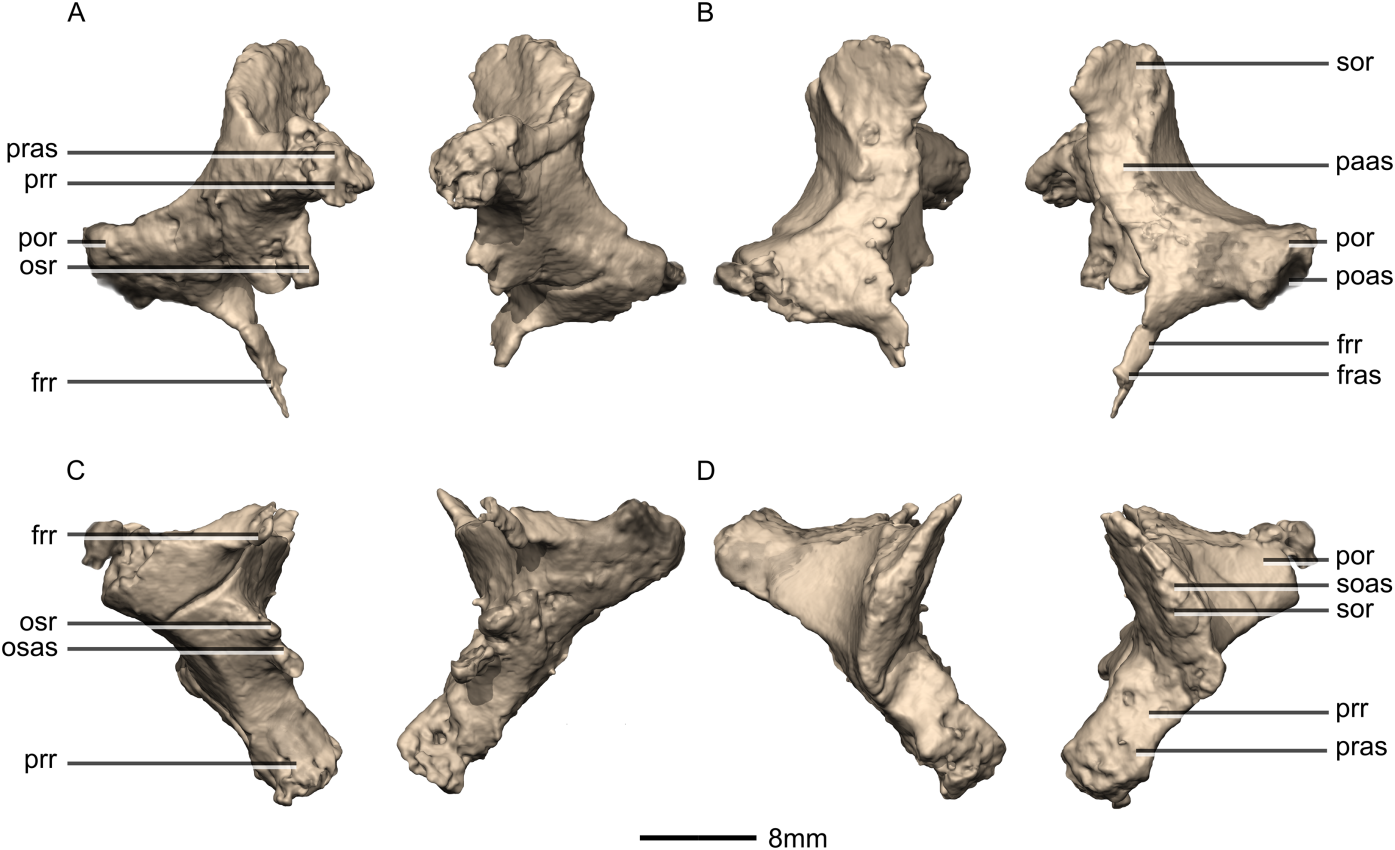
Reconstructed left and right laterosphenoids of BP/1/5241. (A) Ventral view. (B) Dorsal view. (C) Anterior view. (D) Posterior view. frr, frontal ramus; fras, frontal articular surface; osas, orbitosphenoid articular surface; osr, orbitosphenoid ramus; paas, parietal articular surface; poas, postorbital articular surface; por, postorbital ramus; pras, prootic articular surface; prr, prootic ramus; sor, supraoccipital ramus; soas, supraoccipital articular surface.

**Figure 46:**
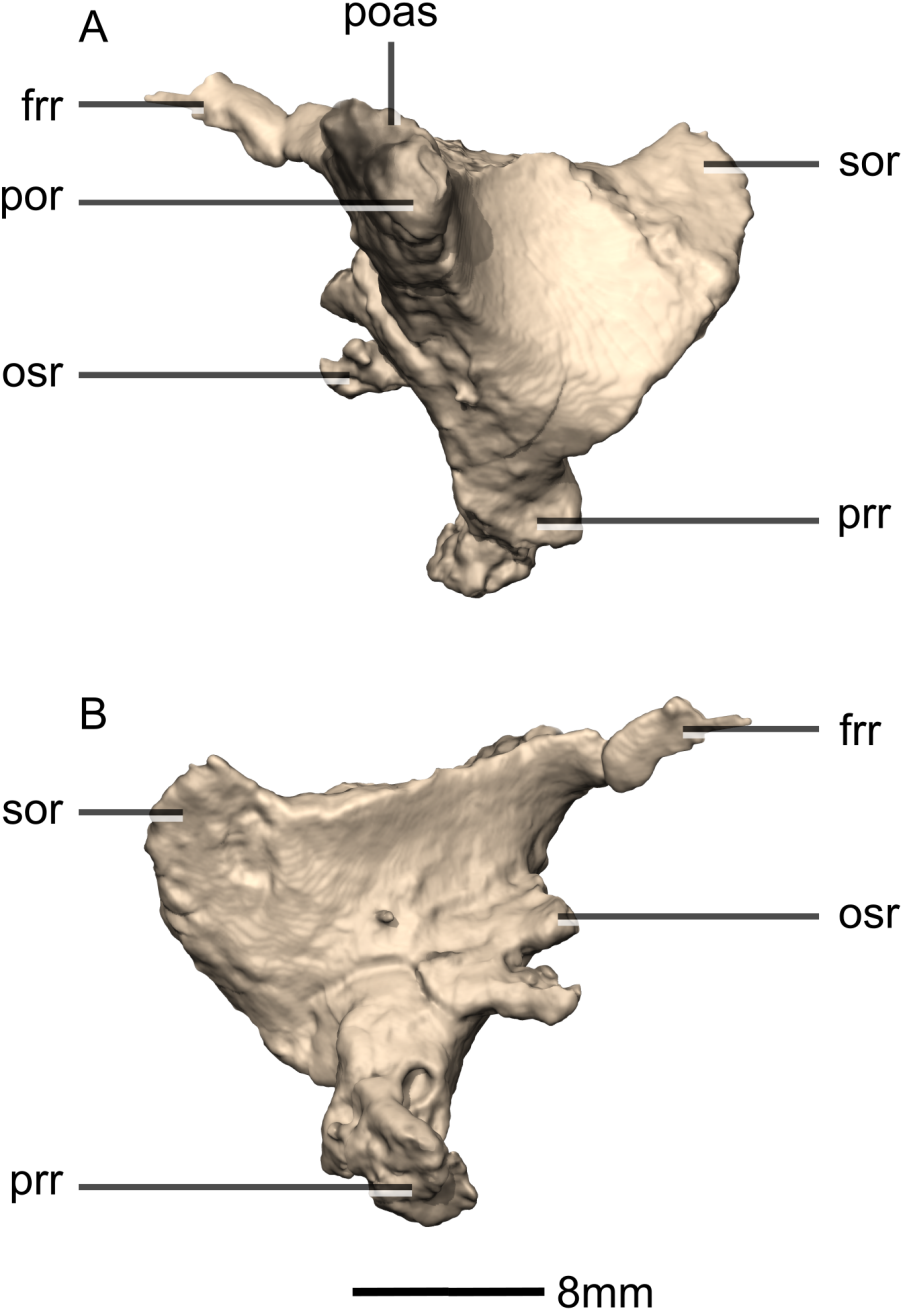
Reconstructed left laterosphenoids of BP/1/5241. (A) Left lateral view. (B) Left medial view. frr, frontal ramus; osr, orbitosphenoid ramus; poas, postorbital articular surface; por, postorbital ramus; prr, prootic ramus; sor, supraoccipital ramus.

**Figure 47:**
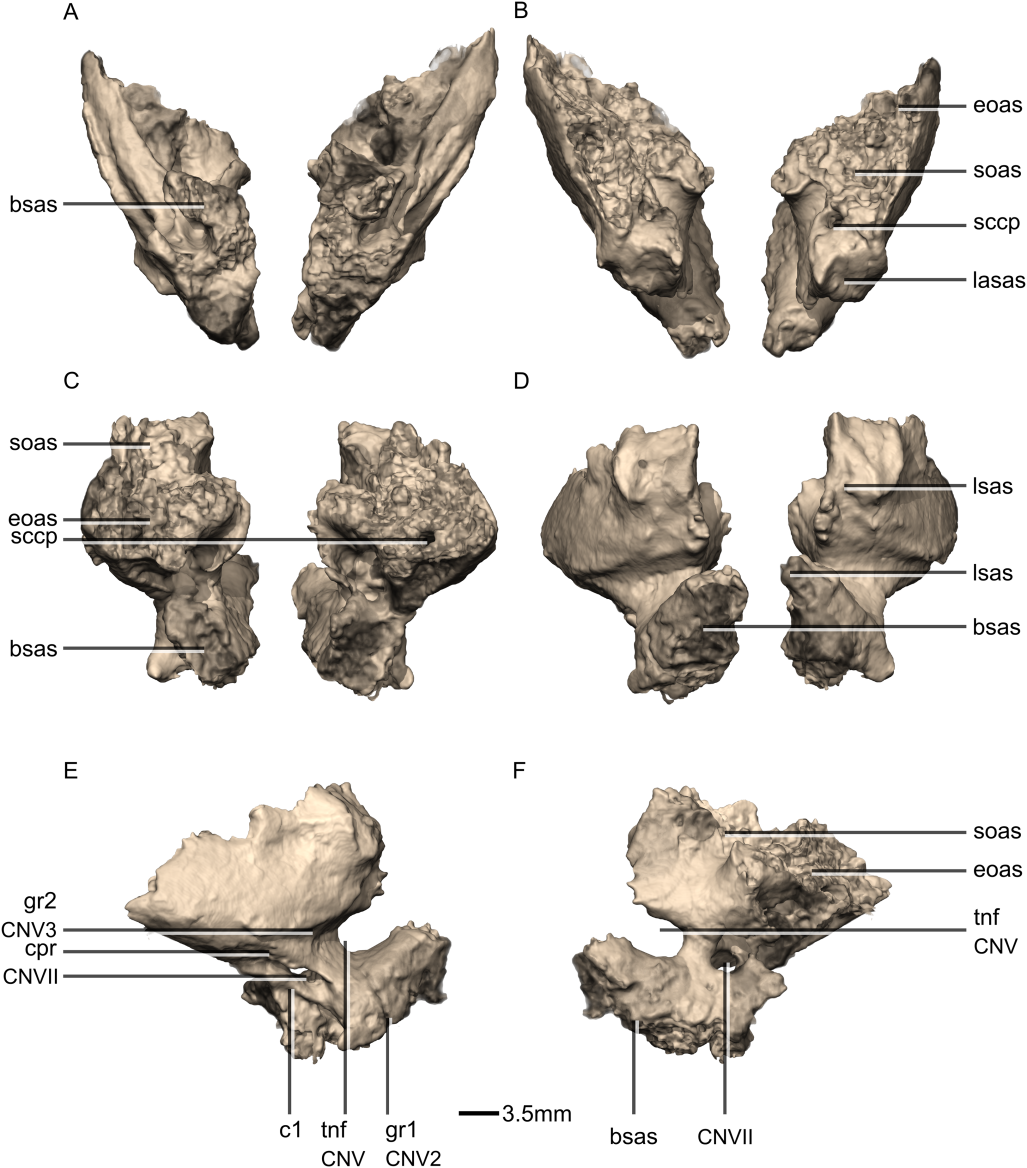
Reconstructed left and right prootics of BP/1/5241. (A) Ventral view. (B) Dorsal view. (C) Posterior view. (D) Anterior view. (E) Right lateral view. (F) Right medial view. bsas, basisphenoid articular surface; c, crista; CN, cranial nerve passage; cpr, crista prootica; eoas, exoccipital articular surface; gr, groove; lsas, laterosphenoid articular surface; sccp, semicircular canal passage; soas, supraoccipital articular surface; tnf, trigeminal nerve foramen.

**Figure 48:**
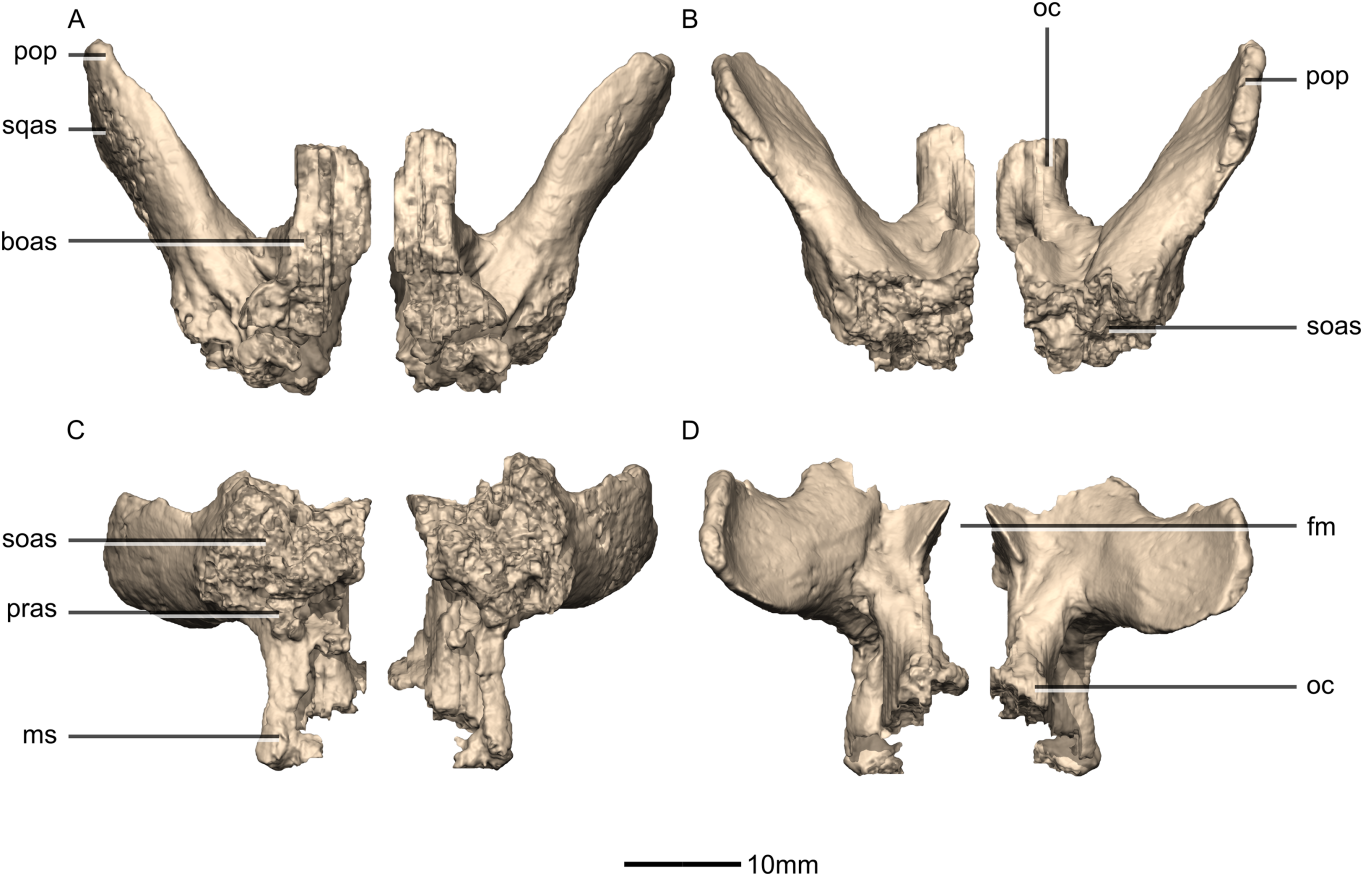
Reconstructed left and right exoccipital/opisthotics of BP/1/5241. (A) Ventral view. (B) Dorsal view. (C) Anterior view. (D) Posterior view. boas, basioccipital articular surface; fm, foramen magnum; ms, metotic strut; oc, occipital condyle; pop, paroccipital process; pras, prootic articular surface; soas, supraoccipital articular surface; sqas, squamosal articular surface.

**Figure 49:**
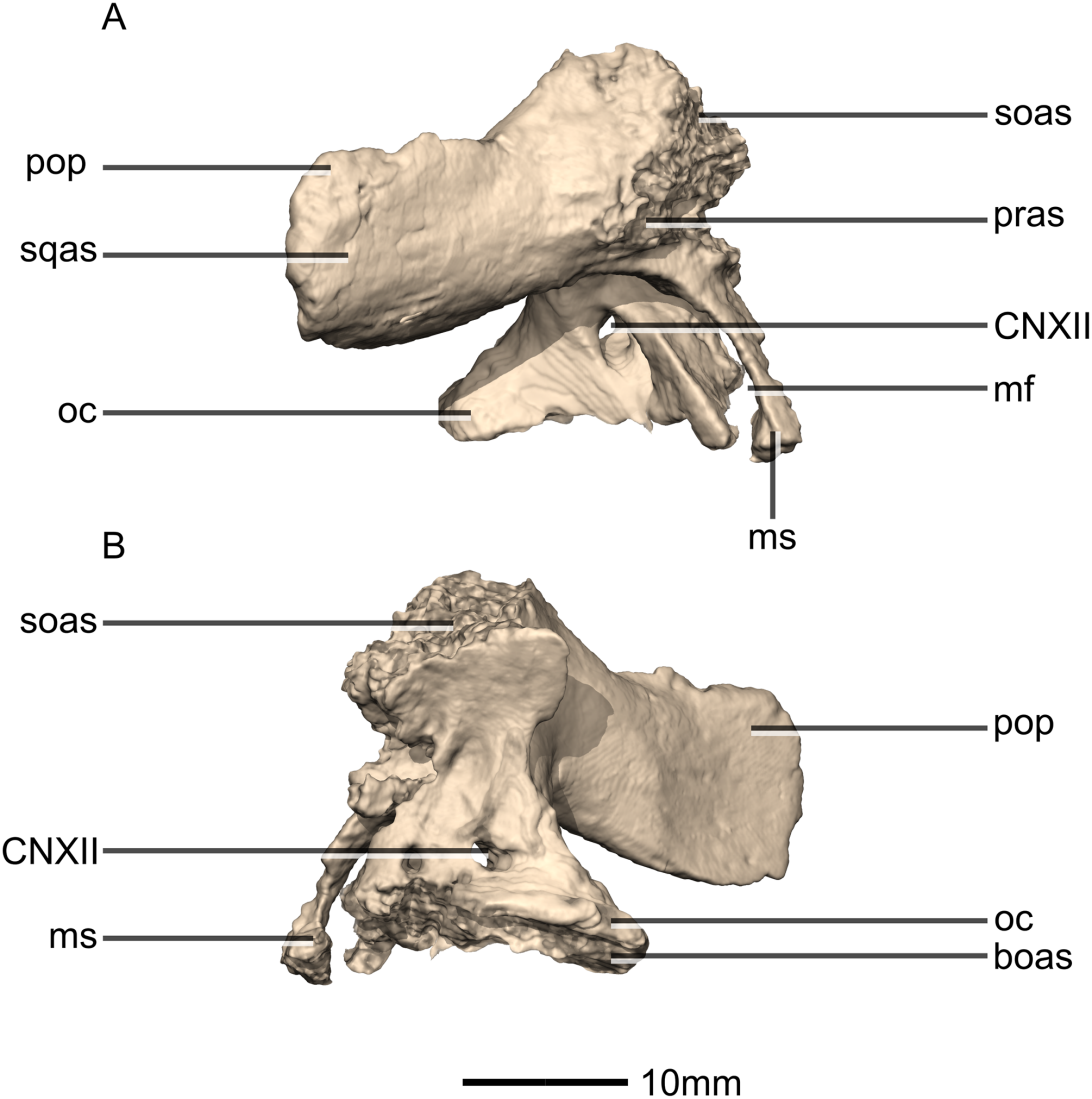
Reconstructed right exoccipital/opisthotic of BP/1/5241. (A) Right lateral view. (B) Right medial view. boas, basioccipital articular surface; CN, cranial nerve foramen; mf, metotic fissure; ms, metotic strut; oc, occipital condyle; pop, paroccipital process; pras, prootic articular surface; soas, supraoccipital articular surface; sqas, squamosal articular surface.

**Figure 50:**
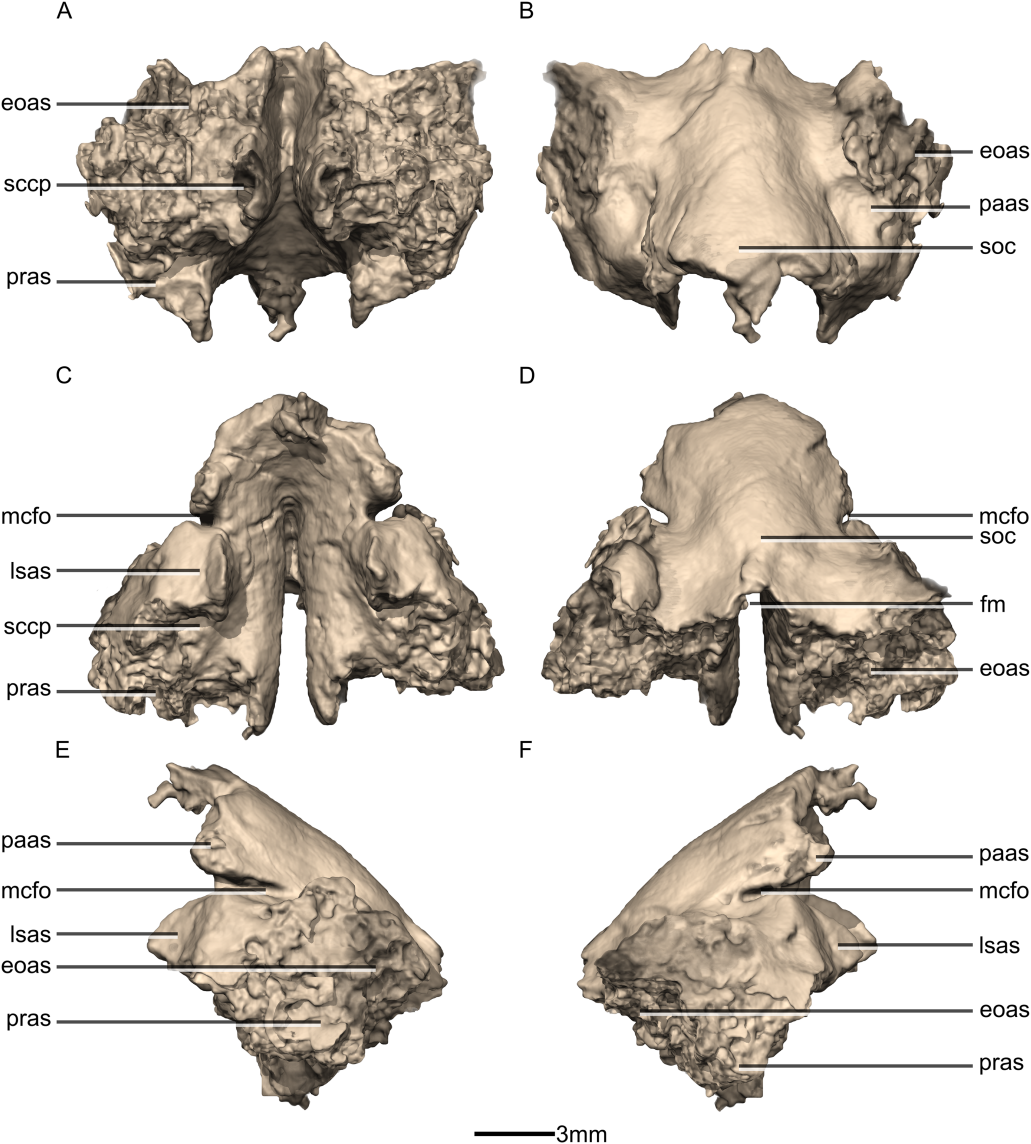
Reconstructed fused left and right supraoccipitals of BP/1/5241. (A) Ventral view. (B) Dorsal view. (C) Anterior view. (D) Posterior view. (E) Left lateral view. (F) Right lateral view. eoas, exoccipital articular surface; fm, foramen magnum; lasas, laterosphenoid articular surface; mcfo, mid cerebral vein foramen; paas, parietal articular surface; pras, prootic articular surface; sccp, semicircular canal passage; soc, supraoccipital crest.

**Figure 51:**
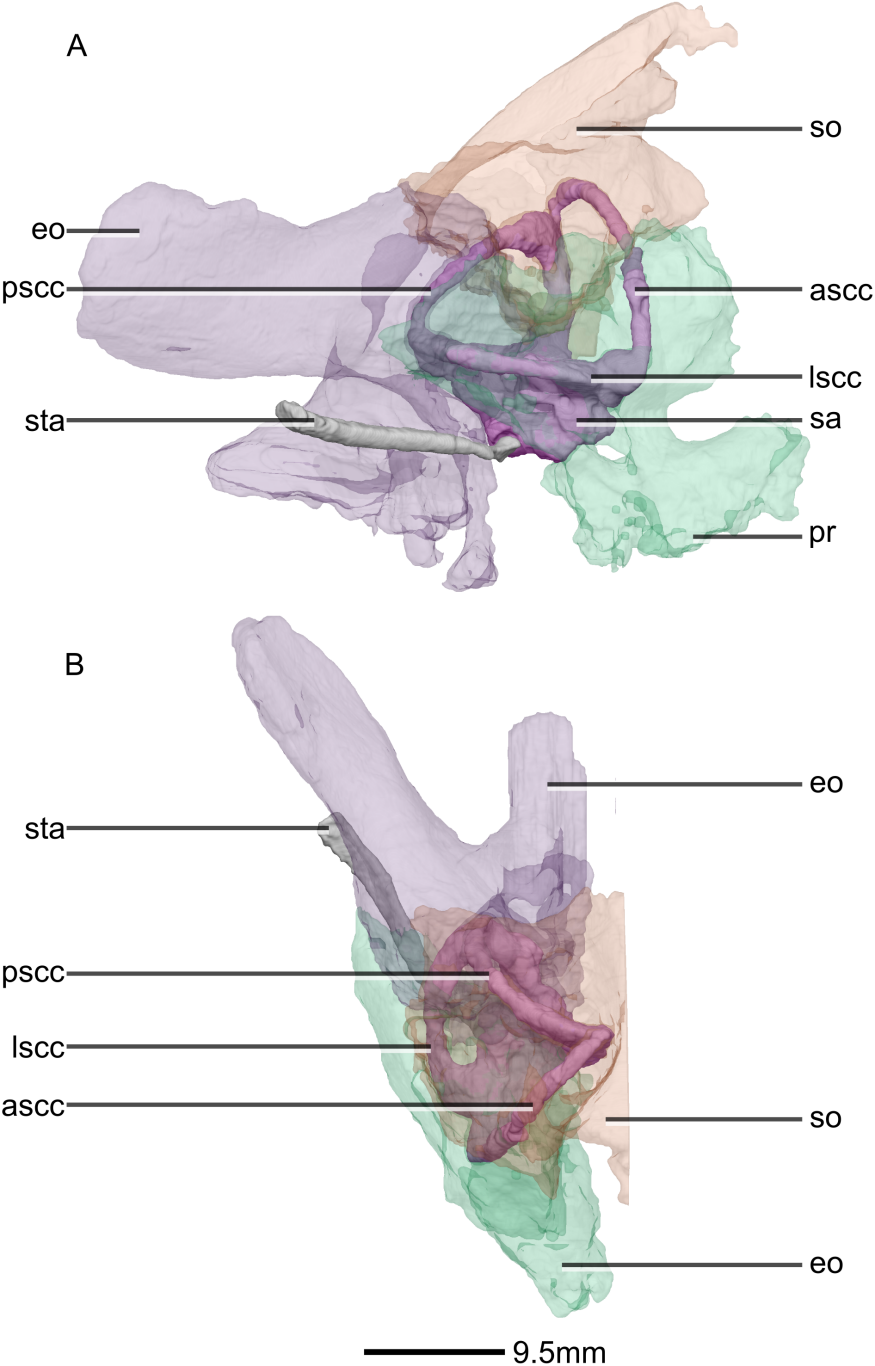
Reconstructed right semi-circular canals, stapes and semi-transparent encasing bones of BP/1/5241. (A) Right lateral view. (B) Dorsal view. ascc, anterior semicircular canal; eo, exoccipital; lscc, lateral semicircular canal; pr, prootic; pscc, posterior semicircular canal; sa, sacculus; so, supraoccipital; sta, stapes.

**Figure 52:**
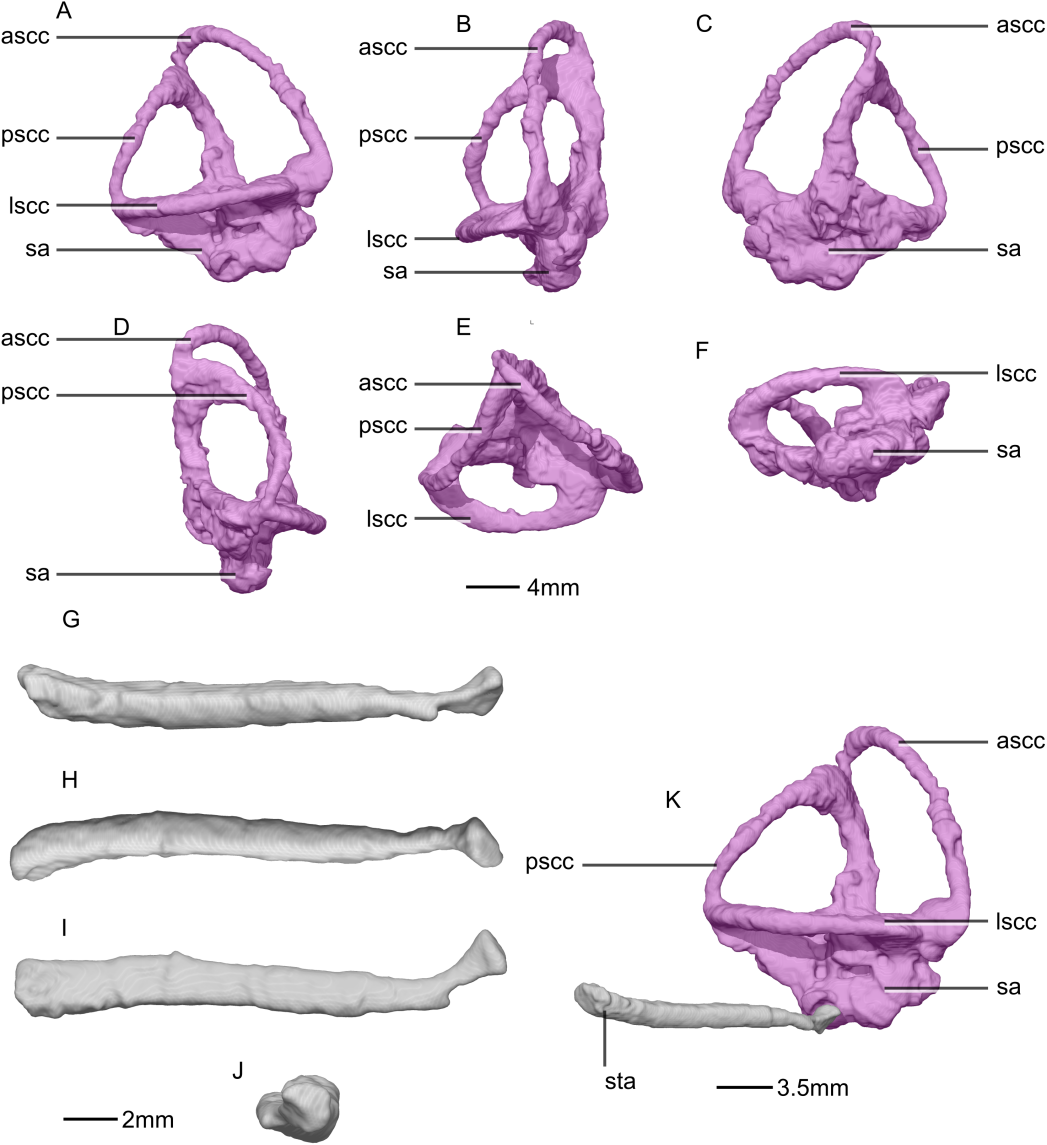
Reconstructed right semi-circular canals and stapes of BP/1/5241. (A) Right semicircular canal in right lateral view. (B) Right semicircular canal in anterior view. (C) Right semicircular canal in right medial view. (D) Right semicircular canal in posterior view. (E) Right semicircular canal in dorsal view. (F) Right semicircular canal in ventral view. (G) Right stapes in right lateral view. (H) Right stapes in medial view. (I) Right stapes in dorsal view. (J) Right stapedial footplate in proximal view. (K) Right semicircular canal and stapes in right lateral view showing the contact between the two. ascc, anterior semicircular canal; lscc, lateral semicircular canal; pscc, posterior semicircular canal; sa, sacculus; sta, stapes.

**Figure 53:**
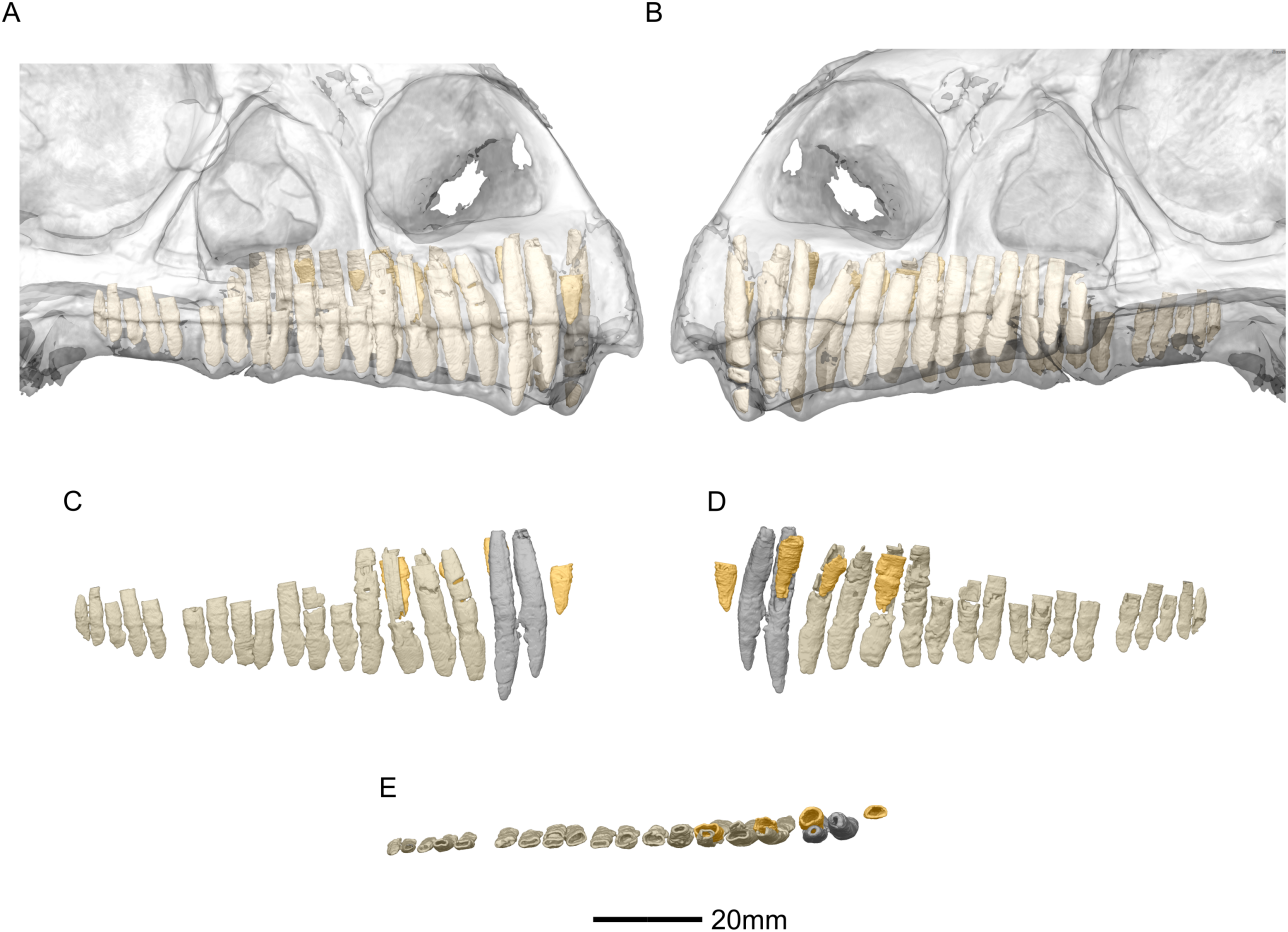
Reconstructed erupted and replacement teeth of BP/1/5241. (A) Right lateral view with semi-transparent skull to show extent of roots (erupted teeth in beige, replacement teeth in orange). (B) Left lateral view with semi-transparent skull (erupted teeth in beige, replacement teeth in orange). (C) Right lateral view with maxillary erupted teeth in beige, premaxillary erupted teeth in grey and replacement teeth in orange. (D) Right medial view with maxillary erupted teeth in beige, premaxillary erupted teeth in grey and replacement teeth in orange. (E) Dorsal view of right side with maxillary erupted teeth in beige, premaxillary erupted teeth in grey and replacement teeth in orange.

SYSTEMATIC PALEONTOLOGY
Dinosauria Owen, 1842
Saurischia Seeley, 1887
Sauropodomorpha Huene, 1932
Massospondylidae Huene, 1914 sensu Yates, 2003b
*Massospondylus carinatus* Owen, 1854

**Holotype:** The syntypes of *M. carinatus* comprised five damaged vertebrae from an outcrop on the Farm Beauchef Abbey, in the Free State Province of South Africa (Owen, 1854; Yates & Barrett, 2010). These were donated to the Hunterian Museum of the Royal College of Surgeons in London and were described by Sir Richard Owen in 1854. The original syntypes were destroyed during a German bombing during World War II (Cooper, 1981; Yates & Barrett, 2010). Plaster casts of at least some of Owen’s specimens remain.

**Neotype:** In 2010, Yates and Barrett formalized the suggestion made by Sues and designated BP/1/4934 as a neotype specimen for *M. carinatus*. The specimen, collected in the Clocolan District of the Free State Province in South Africa, is the largest known *M. carinatus* specimen and comprises a well-preserved articulated skeleton including a skull. The specimen has been illustrated and published by several authors (Gow, Kitching & Raath, 1990; Sues et al., 2004; Yates & Barrett, 2010).

**Referred specimen in this study:** BP/1/5241 (Fig. 1) comprises a partially complete articulated postcranial skeleton and the second largest *M. carinatus* skull. The mandible is not preserved. The specimen was discovered in 1984 in the Barkly East region, Eastern Cape, South Africa. The skull has been illustrated and published several times since 1990 (Gow, Kitching & Raath, 1990; Sues et al., 2004).

**Revised cranial diagnosis:** The following unique combination of features is autapomorphic for *M. carinatus* based on our research and is present in both specimens (BP/1/5241 and BP/1/4934): basipterygoid processes that are separated by an angle smaller than 60° (also present in *Coloradisaurus* and *Mamenchisaurus*). Additionally, the following autapomorphy is present in BP/1/5241 but not confirmable in BP/1/4934: a jugal process of the ectopterygoid that is strongly curved (also present in *Leyesaurus* and *Pantydraco*).

#### Overview and skull openings

The major skull openings comprise the orbit, external naris, antorbital fenestra, infratemporal fenestra and supratemporal fenestra (Figs. 2 and 3). The circular orbit is formed by the postorbital posteriorly, the frontal dorsally, the prefrontal and lacrimal anteriorly and the jugal ventrally. The external naris has a semicircular posterior margin, a horizontal ventral margin and a linear, anteroventrally sloping anterior margin. It is bordered by the premaxilla anteriorly and anteroventrally, the maxilla posteriorly and posteroventrally and the nasal posterodorsally. The triangular antorbital fenestra is bordered anteriorly and ventrally by the maxilla and posteriorly by the lacrimal. The hourglass-shaped infratemporal fenestra is formed by the postorbital dorsally and anterodorsally, the jugal anteroventrally, the quadratojugal posteroventrally and the squamosal posterodorsally. Finally, the supratemporal fenestra is bordered by the parietal medially, posteromedially and anteromedially, the postorbital anterolaterally and the squamosal posterolaterally.

#### Premaxilla

The fused premaxillae form the anterior end of the snout as well as the anteroventral margin of the external naris (Figs. 4 and 5). CT scans show that the internal structure of the bone is solid. The premaxilla contacts the maxilla posteriorly, the nasal dorsally and the vomer medially. It possesses two rami that arise from the body of the premaxilla: the posterolateral maxillary ramus and the anterodorsal nasal ramus. In ventral view, the fused premaxillae have a triangular outline, with the apex of the triangle pointing anteriorly. Together, they form an acute angle in dorsal/ventral views, possibly differing from the condition in *L. huenei* (which is heavily reconstructed) where the outline of the anterior end of the snout is more rounded. The ventral margin of the premaxilla of *M. carinatus* slopes anteroventrally at a low angle in lateral view, so that the anteriormost tip of the rostrum is slightly more ventrally positioned than the rest of the rostrum. In *P. erlenbergiensis*, the anteroventral corner of the snout is rounded in lateral view whereas in *M. carinatus*, it forms a more acute angle.

The maxillary ramus forms the ventral margin of the external naris and extends posterolaterally from the dorsal margin of the premaxilla as a long, distally tapering structure which is proportionally anteroposteriorly shorter and mediolaterally wider than in *P. erlenbergiensis*. The ramus is triangular in lateral and dorsal views and all of its surfaces are smooth and flat. Its ventral margin forms a 90° angle with the posterior margin of the premaxillary body. The axial length of the maxillary ramus is subequal to that of the ventral margin of the alveolar portion of the premaxillary body. The left and right rami are separated by a V-shaped notch that opens posteriorly. The premaxilla–maxilla contact is ‘L’-shaped in lateral view, with the maxillary ramus of the premaxilla overlapping the premaxillary ramus (subnarial ramus) of the maxilla and the posterior margin of the premaxillary body forming a vertical butt-joint with the anterior margin of the maxilla. Above the level of the alveoli, the premaxilla extends medially, forming the anterior part of the palate. In posterior view, these medial processes, along with the medial surface of the premaxilla and the ventral surface of the maxillary ramus, form a medially opening cup-like structure. This is where the anterior process of the premaxillary ramus of the maxilla articulates.

The nasal ramus of the premaxilla extends posterodorsally from the anterodorsal margin of the premaxilla and is oriented more vertically than that of *P. erlenbergiensis*. It extends posteriorly to the same level as the maxillary ramus. It forms the anterior margin of the external naris. This process is thin and slightly mediolaterally expanded at its distal end, where it contacts the nasal. The ramus is also anteroventrally expanded at its base where it joins the premaxillary body. The surfaces of this ramus are smooth.

Three evenly spaced neurovascular foramina are present on the anterolateral surface of the premaxillary body, and are arranged in an anteriorly convex arc, ventral to the nasal ramus (Fig. 5: fo1, fo2 and fo3). They are more visible on the right premaxilla and they follow the contour of the anteriormost margin of the premaxilla. These foramina differ from those of other basal sauropodomorphs: *P. erlenbergiensis* bears two foramina—one is slit shaped, slopes anteroventrally, and is situated anteroventrally to the anteroventral corner of the narial fossa and the second is smaller and circular, situated dorsally to the first tooth; whereas *S. aurifontanalis* has a small neurovascular foramen present dorsal to the first premaxillary tooth, on the lateral surface of the premaxilla at the same height as the maxillary neurovascular foramina. The medial surface of the alveolar region of the premaxillary body in *M. carinatus* is formed by one continuous sheet of bone whereas in *P. erlenbergiensis* and *L. huenei*, the alveoli are separated by individual interdental plates along the medial surface.

#### Maxilla

Portions of the lateral surface of the maxilla are abraded, leaving only a thin layer of cortical bone (Figs. 6 and 7). CT scans show that the internal structure of the bone is solid. The maxilla contacts the premaxilla anteriorly, the lacrimal and nasal dorsally, the jugal and lacrimal posteriorly and the palate and vomer medially. The maxilla is a triradiate bone and comprises three rami: the premaxillary ramus, the lacrimal ramus and the jugal ramus. The maxilla is at its dorsoventral highest anteriorly, and the dorsal and ventral margins remain parallel for most of their lengths and taper out posteriorly, at the distal end of the jugal ramus. The maxilla forms 75% of the overall anteroposterior length of the tooth row. It forms the posteroventral and posterior margins of the naris, as well as the ventral and anterior margins of the antorbital fenestra. The lateral surface of the maxilla bears neurovascular foramina which open posterolaterally. These are arranged in a linear manner and are situated halfway up the dorsoventral height of the entire lateral surface of the maxilla. The maxillae do not contact each other medially.

The premaxillary ramus of the maxilla is the shortest of the three. It is oriented parallel to the main axis of the skull and is approximately 30% of the entire maxillary length. All the surfaces of this ramus are smooth, apart from the dorsal surface that is rugose. The ventral surface is convex. The anterodorsal portion of the ramus is dorsally overlapped by the maxillary ramus of the premaxilla. The anterior margin of the maxilla is vertical and abuts the vertical posterior surface of the premaxillary body. The contact between the premaxilla and maxilla is therefore an inverted L-shape in lateral view. The surface of this contact is flat and smooth. The premaxillary ramus also bears an anteriorly oriented medial process (Figs. 6 and 7: p1) that extends from the dorsomedial surface of the anterior end of the premaxillary ramus. This process has convex surfaces and tapers distally. All the surfaces are smooth except for the dorsal one that is uneven. This dorsal surface is overlapped by the ventral surface of the maxillary ramus of the premaxilla. The ventral surface of this anteromedial process of the maxilla contacts the dorsal surface of the medial palatal processes of the premaxilla. These anterior processes are therefore slotted in the maxilla. In *P. erlenbergiensis*, the anteromedial process of the maxilla extends medially from the anteromedial surface of the maxilla and forms a medially extending shelf of bone. The anteromedial process of the maxilla in *M. carinatus* extends anteriorly from the anterodorsal margin of the maxilla. The medial surface of this process does not extend further medially than the medial surface of the rest of the maxilla. The medial surface of the dorsal portion of the premaxillary ramus of the maxilla (dorsal to the alveoli) contacts the vomers.

The lacrimal ramus of the maxilla extends dorsally from a point approximately one third of the anteroposterior length of the maxilla, as in *L. huenei*, and *P. erlenbergiensis*. Its base forms an 85° angle relative to the ventral margin of the maxilla, and the dorsal half becomes more posterodorsally oriented at a 60° angle. This is unlike the lacrimal rami of *L. huenei* and *P. erlenbergiensis* which are straight and form approximately 55° angles with the ventral margin of the maxilla. This ramus forms the posterior margin of the naris and thus separates the external naris from the antorbital fenestra. The base of the lacrimal ramus is triangular in lateral view, but it is much less anteroposteriorly expanded in *M. carinatus* than in of *L. huenei* and *P. erlenbergiensis*. The antorbital fossa is more deeply excavated with proportionally more bone forming the medial wall of the fossa in these taxa than in *M. carinatus*. The lacrimal ramus possesses a strong ridge (Fig. 7: ri1) of bone that runs along the midline of its entire lateral surface in *M. carinatus*. This ridge expands anteroposteriorly as it extends ventrally, becoming less developed laterally, ultimately grading smoothly into the alveolar region of the lateral surface of the maxilla. The distal end of the lacrimal ramus tapers and forms a tongue-like joint with the forked anterior ends of the lateral processes of the lacrimal. It is also medially overlapped by the long tapering maxillary ramus of the lacrimal. The ventral portion of the nasal laterally overlaps the dorsal portion of the lacrimal ramus of the maxilla. The lacrimal ramus bears a long ovoid-shaped fossa (Fig. 7: f1) on its ventromedial surface. This fossa covers the ventral half of the medial surface of the lacrimal ramus. It is anteroposteriorly short at its dorsal margin and expands anteroposteriorly as it extends ventrally, becoming less developed.

The jugal ramus is slender and elongate relative to those of *L. huenei* and *P. erlenbergiensis*. It extends posteriorly from the junction between the first two rami and is oriented in the same plane as the main axis of the skull. This ramus represents 60% of the length of the entire maxilla. The lateral and medial surfaces are slightly convex and smooth. The dorsal and ventral surfaces are poorly preserved due to the thinness of the bone and the presence of alveoli. In *P. erlenbergiensis* and *L. huenei*, the alveoli are separated by individual interdental plates along the medial surface. This is not the case in *M. carinatus* where the medial surface of the jugal ramus is one continuous sheet of bone. This ramus possesses alveoli along its entire length. The posterior half of the ramus tapers and comes to an end at the midpoint of the jugal. This posterior half ventrally underlaps the oblique contact between the jugal and the lacrimal. The maxilla therefore contacts the anteroventral corner of the lacrimal as well as the anterior portion of the ventral surface of the jugal.

The contact between the maxilla and the palatine is not preserved although there is an almost contact between the medial surface of the maxilla and the palatine, ventral to the maxilla–jugal contact.

Neurovascular foramina are present on the lateral surfaces of the jugal and premaxillary rami of the maxilla, offset dorsally from the alveolar margin by approximately 3 mm. The first foramen is situated between the second and third maxillary teeth. These are not evenly spaced but are linearly arranged and similarly sized, except for the posteriormost one, which is larger. The foramina open posteriorly.

#### Nasal

Both nasals are preserved but they are missing the medial edges where they contact along the midline of the dorsal surface of the rostrum (Figs. 8 and 9). Internally, CT scans reveal that the nasal is a solid sheet of bone. A short section of preserved nasal contact immediately posterior to the premaxillary contact shows that at least the anterior ends of the nasals were unfused. This is difficult to confirm on the neotype skull due to slight deformation in that area. The nasal contacts the premaxilla anteriorly, the frontal posteriorly and the lacrimal and maxilla ventromedially. The nasal is triangular in lateral view with a subhorizontal dorsal margin. The nasals comprise a premaxillary ramus, a maxillary ramus, and a frontal ramus. The most salient external feature of the nasal is a prominent depression posterior to the naris, along the dorsal margin of the nasal. This feature is shared with *L. huenei*, where it is more pronounced. The remaining portions of the dorsal and lateral surfaces of the nasal are convex and smooth. Likewise, the ventral and medial surfaces are concave and lack any distinguishing features.

The anteriorly extending premaxillary ramus forms the entire dorsal and posterodorsal margins of the external naris. It is formed by a dorsoventrally thin sheet of bone that tapers as it extends anteriorly. This ramus is subhorizontally oriented, whereas in *L. huenei* and *P. erlenbergiensis* the rami extend in a more anteroventral direction, making the ventral margin more concave. The premaxillary ramus of the nasal in *P. erlenbergiensis* is also proportionally anteroposteriorly longer than that of *M. carinatus.* The anteromedial surface of the premaxillary ramus contacts the posterolateral margins of the distal end of the nasal ramus of the premaxilla in a lap joint that extends anteriorly to the approximate midpoint of the nasal ramus of the premaxilla. Damage to the medial margin of the premaxillary ramus makes it unclear whether the left and right nasals met anteriorly to overlap the anterodorsal surface of the premaxillary nasal ramus.

The maxillary ramus is an anteroposteriorly long, triangular sheet of bone that tapers sharply as it extends ventrally. It forms the dorsal half of the posterior margin and the entire posterodorsal margin of the external naris. The maxillary ramus of *P. erlenbergiensis* has an anteroposteriorly wide base that tapers abruptly and forms an anteroposteriorly compressed and dorsoventrally high and rectangular distal half end. In *M. carinatus*, a shallow postnarial fossa (Fig. 9: f1) is present on the anterolateral surface of the maxillary ramus where it joins the premaxillary ramus. This fossa extends from the external naris and may be associated with the soft-tissue anatomy of the narial region. The maxillary ramus laterally overlaps the anterior end of the maxillary ramus of the lacrimal in a dorsoventrally long lap joint. Immediately dorsal to this lap joint, on the posterior margin, a posteriorly opening notch (Fig. 9: no1) is present on the right side of the skull. This feature cannot be confirmed on the left side, due to breakage, and may be due to poor preservation. A low, anteroventrally sloping ridge of bone (Fig. 9: ri1) along the medial surface marks the dorsal margin of this contact. The left maxillary ramus of *M. carinatus* is triangular and bears a posteriorly extending flange of bone that is not present in other taxa. This feature could be due to incomplete preservation of that portion of the bone.

The frontal ramus is a mediolaterally narrow, triangular sheet of bone that tapers as it extends posteriorly. It is similar in size and shape to the premaxillary ramus, and it dorsally overlaps the nasal ramus of the frontals, forming an anteroposteriorly extensive lap joint.

#### Prefrontal

Both prefrontals are complete, well-preserved and undistorted (Figs. 10 and 11). The prefrontal forms the anterodorsal corner of the orbit. CT scans show that the internal structure is composed primarily of trabecular bone. The prefrontal contacts the lacrimal anteroventrally, the nasal anterodorsally, and the frontal posteriorly. It comprises a body, a frontal ramus and a lacrimal ramus. Unlike the condition in *P. erlenbergiensis* and *S. aurifontanalis*, there is no bony connective sheet on the lateral surface connecting the lacrimal and frontal rami.

The frontal ramus extends posterodorsally from the posterior surface of the body as a distally rounded, dorsoventrally flattened, tab-like structure. This is unlike the condition in the massospondylid *S. aurifontanalis*, where this ramus is proportionally longer and more acuminate. The frontal ramus of *M. carinatus* bears two frontal articular surfaces: a laterally positioned, pronounced oval facet that opens posteriorly on the dorsal margin of the orbital rim, and a medially positioned groove along the anterior end of the medial surface. All the surfaces of this ramus are smooth.

The lacrimal ramus of the prefrontal begins at the anterodorsal margin of the orbit and forms a shallowly concave articulation with the posterior surface of the lacrimal angle. It extends ventrally from the anteroventral surface of the body as a tapering, splint-like structure. This process extends along the medial surface of the dorsal half of the ventral ramus of the lacrimal, to which it is closely appressed. The articular surface of the prefrontal along this suture is flat and rugose whereas the remainder of the surfaces are flat and smooth. The morphology of the lacrimal ramus of the prefrontal is markedly different between *M. carinatus* and *L. huenei*. That of *L. huenei* only extends along a short distance onto the posteromedial surface of the lacrimal (Barrett, Upchurch & Wang, 2005) whereas the lacrimal ramus of *M. carinatus* and *P. erlenbergiensis* are long and splint-like, extending more than halfway down the dorsoventral height of the posterior surface of the lacrimal.

The prefrontal body arcs anterodorsally as it extends over the anterodorsal corner of the orbit. The dorsal surface of the prefrontal body is mediolaterally flat. It extends medially as a sheet of bone that meets the nasal in a posteromedially–anterolaterally oriented suture dorsal to the nasal ramus of the frontal. The ventral surface of the prefrontal body forms the dorsomedial wall of the orbit and is mediolaterally flat and smooth. The lateral surface of the prefrontal body forms a ridge-like structure demarcating the orbital margin. This structure is continuous with the lateral margin of the frontal, forming the orbit margin. The medial surface of the prefrontal body is concave and rugose, forming an anteroposteriorly oriented trough-like feature that would have formed the lateral margins of the olfactory bulbs.

#### Lacrimal

Both lacrimals are complete, well-preserved and undistorted (Figs. 12 and 13). The lacrimal separates the antorbital fenestra from the orbit. CT scans show that the internal structure of the bone is solid. It contacts the nasal and maxilla anterodorsally, the prefrontal posterodorsally and the jugal ventrally. It comprises an anteroventrally directed maxillary ramus and a posteroventrally oriented jugal ramus. The two rami meet at approximately 90° to each other to form a sharp lacrimal angle which differs from most other nonsauropodan sauropodomorphs, such as *L. huenei*, *S. aurifontanalis*, and *P. erlenbergiensis* where the dorsal surface of the lacrimal is concave and the lacrimal angle is rounded. The lacrimal of *M. carinatus* is proportionally taller, thinner, and has a proportionally much shorter maxillary ramus that that of *P. erlenbergiensis*.

The main portion of the maxillary ramus is anteroposteriorly short and has a subtriangular cross-section, with rounded vertices. Its lateral surface is poorly exposed on the lateral side of the skull, forming only a small, triangular shelf overhanging the posterodorsal corner of the antorbital fenestra. The dorsal surface of this shelf is smooth, flat, and inclined dorsomedially and posteriorly. The medial surface of the maxillary ramus is flat and oriented vertically. Three distinct processes project anteroventrally from the maxillary ramus: (1) an anteroposteriorly elongate ventromedial process that forms an extended lap joint along its lateral surface with the lacrimal ramus of the maxilla (Figs. 12 and 13: p1); (2) a relatively short dorsomedial process that is overlapped by the lateral margin of the nasal; and (3) a similarly sized lateral third process that extends a short distance anteriorly from the shelf overhanging the antorbital fossa. Together the dorsomedial and lateral processes form an anteriorly opening, V-shaped notch that articulates with the dorsalmost tip of the lacrimal ramus of the maxilla (Figs. 12 and 13: p2 and p3). The condition of other sauropodomorph taxa is poorly known, but in *P. erlenbergiensis* the maxillary ramus lacks a dorsomedial process but does possess a dorsolateral process that forms a U-shaped notch with the longer ventromedial process in dorsal view. The dorsolateral and medial surfaces of these rami are smooth and flat whereas the ventral surfaces are smooth and concave.

The jugal ramus is an anteroposteriorly thin, dorsoventrally tall, pillar-like structure. It is inclined anterodorsally at approximately a 50° angle to horizontal. The jugal ramus expands anteroposteriorly as it extends ventrally, unlike the condition in *S. aurifontanalis* where the ventral end of the jugal is not significantly anteroposteriorly expanded. The expanded ventral end has a rugose surface where it contacts the anterior end of the jugal. This contact is posterodorsally inclined. The anteroventral corner and a thin strip along the medialmost ventral margin of the jugal ramus contact the posterior end of the jugal ramus of the maxilla in simple butt joints.

The lateral surface of the ventral distal end of the jugal ramus is a very mediolaterally narrow, dorsoventrally tall sheet of bone with a smooth, convex surface. It is emarginated distally by an anteroposteriorly long excavation for the antorbital fossa on the anterior side, which forms a tall triangular lamina of bone medially that grades into the shaft of the lacrimal (Fig. 13: f1). The emarginated area within the fossa is similarly developed in *L. huenei*, but in *P. erlenbergiensis* this region is hypertrophied. In *M.* carinatus, this fossa is continuous with a deep, dorsoventrally oriented, mediolaterally narrow groove (Fig. 12: gr1) that excavates the entire anterior surface of the jugal ramus. The dorsal end of this groove is pierced by the nasolacrimal duct, which forms a tall foramen in the floor of the groove. The lateral and medial margins of the anterior groove grade into the ventral surface of the lateral and ventromedial processes of the maxilla ramus.

The ventral end of the medial surface bears a tall, triangular fossa on its anterior half (Fig. 13: f2). A thin lip of bone marks the posterior boundary of this fossa where it meets the posterior half of the medial surface. This posterior half of the medial surface is shallowly concave. A narrow, dorsoventrally oriented groove is developed along the dorsal end of the medial surface. The medial sheet of the posterior surface borders this groove posteriorly. It is continuous dorsally with a pneumatic pocket formed in the lacrimal angle. The lacrimal angle is slightly inflated and heavily pneumatized. The posterodorsal surface of the lacrimal angle forms a slightly convex facet that articulates with a corresponding concave region on the anteroventral corner of the prefrontal. A deep, medially opening pneumatic pocket communicates anteriorly with a large, circular foramen between the bases of the processes of the maxillary ramus as well as posteriorly with the dorsal terminus of the tunnel-like posterior lacrimal fossa.

The ventral half of the posterior surface of the jugal ramus is shallowly convex. The medial margin of the dorsal half is expanded to form an anteroposteriorly compressed sheet of bone, which twists as it extends dorsally, so that its posterior surface faces posterolaterally. At its dorsal end, this sheet of bone grades into the lacrimal angle but remains as a distinct ridge. Lateral to this sheet of bone, a deep groove-like fossa opens posteriorly, and within this fossa is the posterior opening of the foramen for the nasolacrimal duct. The fossa extends dorsally above this foramen and into the lacrimal angle as a tunnel-like feature, ultimately communicating with a pneumatic pocket on the medial surface of the lacrimal angle.

#### Postorbital

Both postorbitals are complete, well-preserved and undistorted. The postorbital forms the posterior margin of the orbit and separates the supratemporal fenestra from the infratemporal fenestra (Figs. 14 and 15). CT scans show that the internal structure is composed primarily of trabecular bone. It contacts the jugal posteroventrally, the frontal anterodorsally and the squamosal posteromedially. It is a triradiate bone that comprises three rami: a jugal ramus, a frontal ramus and a squamosal ramus. The postorbital of *M. carinatus* is a gracile bone, similar to that of *P. erlenbergiensis* and *S. aurifontanalis*. The postorbital of *L. huenei* is proportionally more robust (with a dorsoventrally higher squamosal ramus, anteroposteriorly longer frontal and squamosal rami, and a mediolaterally and dorsoventrally more robust orbital rim).

The medially compressed jugal ramus is dorsoventrally oriented and its distal end tapers and forks into a lateral and a medial process (Fig. 15: p1 and p2 respectively). The posterior surface bears a low ridge (Fig. 14: ri1) that extends from the base of the squamosal ramus and grades into the posterior surface of the distal end of the lateral process (Figs. 14 and 15: p1) of the jugal ramus. Lateral to this ridge, dorsal to the junction of the medial and lateral processes, is a small ovoid facet where the postorbital ramus of the jugal articulates with the postorbital. The lateral surface of the jugal ramus is slightly convex. The medial surface is smooth and flat and slopes ventrolaterally. The jugal ramus of *S. aurifontanalis* has a slight mediolateral expansion at its distal end and is slightly laterally oriented in anterior view.

The dorsoventrally compressed frontal ramus widens mediolaterally as it extends dorsally, forming a dorsoventrally flattened tab-like structure. The frontal ramus overlaps the postorbital ramus of the frontal. The ventral surface of the frontal ramus is slightly concave and rugose, allowing for the convex dorsal surface of the frontal’s postorbital ramus to articulate with it. The dorsal surface is convex and smooth. Together, the frontal and jugal rami of the postorbital form the entire posterior margin of the orbit. The anterior margin of the postorbital is more concave in *L. huenei* than that of *M. carinatus* with the frontal and jugal rami being more anteriorly oriented (and not anterodorsally and anteroventrally respectively as in *M. carinatus*). This is best seen in lateral view. In *S. aurifontanalis*, the angle between the frontal ramus anterior margin and the jugal ramus anterior margin is sharp rather than circular and smooth, giving the postorbital a square anterior margin rather than a semicircular one as in *M. carinatus*. Medially, the frontal ramus of *M. carinatus* bears a triangular flange of bone that has smooth and flat dorsal and ventral surfaces. The articulation with the parietal and frontal differs between basal sauropodomorphs. The postorbitals of *L. huenei* and *S. aurifontanalis* possess a notch between their frontal rami and their medial flanges, forming a forked distal end with two processes separated by a U-shaped, semicircular notch. The posterior process is connected to a web of bone that extends posteromedially from the medial surface of the frontal ramus to the medial surface of the base of the squamosal ramus junction. It is not a medially extending flange of bone as in *M. carinatus*. The anterior process of the frontal ramus of *S. aurifontanalis* and *L. huenei* contact the frontal and the posterior one contacts the parietal, excluding the frontal from the supratemporal fenestra margin.

The squamosal ramus projects posteriorly from the junction of the frontal and jugal rami at the midheight of the postorbital. This ramus is the shortest of the three rami and forms the anterodorsal and dorsal margins of the infratemporal fenestra and the lateral margin of the supratemporal fenestra. This ramus tapers distally, where it contacts the squamosal by fitting into the fork of the postorbital ramus of the squamosal, overlapping it laterally. The medial surface is flat and smooth. It is ventrolaterally sloped in posterior view. The lateral surface is smooth and vertically flat. The angle between the frontal ramus and the squamosal ramus is near 180° and therefore flat in *S. aurifontanalis*, unlike *M. carinatus*.

#### Squamosal

Both squamosals are complete, well-preserved and undistorted. The squamosal is a tetraradiate bone and it forms the posterodorsal corner of the skull (Figs. 16 and 17). CT scans show that the internal structure of the bone is solid. It contacts the exoccipital posteromedially, the quadrate ventrally, the parietal dorsomedially, the postorbital anteriorly, and the supraoccipital ventromedially. The dorsal surface of the squamosal body forms a saddle-shaped structure. Four rami extend from the squamosal body: the exoccipital ramus, the quadrate ramus, the postorbital ramus and the supraoccipital ramus.

The exoccipital ramus is triangular in dorsal view. It extends posteroventrally from the squamosal body and forms a mediolaterally compressed tabular structure. This tabular portion slopes ventromedially at 45° to the dorsoventral axis of the skull. At the proximomedial corner of the ramus, a small lip of bone overhangs the medial surface, forming a shallow, smooth concavity. This concavity extends distally and becomes shallower across the entire medial surface of the ramus, forming a concavo-convex articulation with the paroccipital process. The lateral surface of the exoccipital ramus slopes ventromedially and is slightly convex and smooth.

The quadrate ramus is the longest of the four rami. It is straight, extends anteroventrally, and its dorsoventral length is approximately one-third that of the skull height. The quadrate ramus is proportionally dorsoventrally higher in *M. carinatus* and *P. erlenbergiensis*, being more than four times its anteroposterior length at its base, unlike *L. huenei*, where it is less than four times. In *P. erlenbergiensis*, this ramus is anterolaterally oriented and curves back posteriorly at its distal end, giving it a concave posterior margin and convex anterior margin in lateral view. In *M.* carinatus, the anterior margin of this ramus forms the dorsal half of the posterior margin of the infratemporal fenestra in lateral view. It is composed of a lateral sheet of bone and a medial sheet of bone that meet each other anteriorly at an acute angle to form a deep, posteriorly opening groove (Fig. 16: gr1) in which the anterior margin of the quadratojugal ramus of the quadrate articulates. The anterior surface of this ramus, where the sheets meet each other, faces anterodorsally as a convex surface. As this groove extends dorsally, it is continuous with the quadrate cotyle, which is located on the ventral surface of the squamosal body at the junction of the quadrate and exoccipital rami. The cotyle is a deep fossa with an ovoid outline in ventral view. The long axis of the fossa is oriented anteromedially, mirroring the shape of the quadrate head. In lateral view, the lateral sheet of the quadrate ramus tapers as it extends ventrally, forming a tall triangular splint shape, with the apex of the triangle pointing anteroventrally. The posterior corner of this triangle grades into the base of the exoccipital ramus, forming a shelf-like lateral border to the quadrate cotyle. Along the articulation with the quadratojugal ramus of the quadrate, the lateral surface of the medial sheet faces almost completely laterally, but at the proximal end near the contact with the quadrate head, the medial sheet changes its orientation such that the lateral surface faces more posteriorly and the medial surface faces more anteriorly. This change in orientation forms a medially directed bulge in the medial border of the quadrate cotyle. All the surfaces of this ramus are smooth.

The postorbital ramus projects anteriorly from the lateral surface of the squamosal and forks into dorsal and ventral processes as it extends anteriorly. The dorsal and ventral processes (Figs. 16 and 17: p1 and p2 respectively) are subsymmetrical and form elongated triangles in lateral view with the apex of the triangles pointing anteriorly. The medial surfaces of both processes are flat and smooth and face directly medially. The dorsal margin of the dorsal process of the postorbital ramus forms the posterior corner of the supratemporal fenestra. The lateral surface is convex and smooth. The lateral surface of the ventral ramus is flat but bears an anteroposteriorly oriented ridge of bone that is present along its ventrolateral margin (Fig. 17: ri1). This ridge extends posteriorly to grade into the lateral sheet of the quadrate ramus at its contact with the exoccipital ramus. This ridge overhangs the proximal end of the lateral sheet of the quadrate ramus, forming a ventrally facing groove between these two elements. A deep triangular fossa extends anteriorly from the junction of these two processes and articulates with the squamosal ramus of the postorbital. The postorbital ramus of the squamosal in *P. erlenbergiensis* is not forked, contrary to that of *M. carinatus*, but has a concave lateral surface and convex medial surface, giving it a U-shaped cross section. It is proportionally longer than that of *M. carinatus* and its anterior distal margin extends further anteriorly than that of the supraoccipital ramus.

The parietal ramus projects anteriorly from the medial surface of the squamosal and contacts both the parietal and the supraoccipital. This ramus is mediolaterally compressed and forms a tab-like structure that is rectangular in medial view. This ramus in *P. erlenbergiensis* is proportionally anteroposteriorly longer and more dorsoventrally compressed with a tapering distal end. In *M.* carinatus, the ventral half of the medial surface of this ramus forms a small contact with the lateral surface of the supraoccipital. This contact evidently was poorly sutured in BP/1/5241, because there is slight separation between these elements on both the left and right sides of the skull. The dorsal half of the medial surface of this ramus contacts the posterior portion of the lateral surface of the parietal wings. This contact surface is broadly triangular in medial view, and much more extensive than the contact with the supraoccipital. The medial surface of this ramus is flat and rugose whereas the lateral surface is slightly convex and smooth.

#### Jugal

Both jugals are complete, well-preserved and undistorted. The jugal is shaped like a sideways ‘Y’ in lateral view, with the opening of the Y facing posteriorly (Figs. 18 and 19). CT scans show that the internal structure is composed primarily of trabecular bone in the lacrimal ramus, whereas the rest of the bone structure is solid. The jugal contacts the maxilla anteroventrally, the lacrimal anterodorsally, the postorbital posterodorsally and the quadratojugal posteroventrally. The jugal possesses a main body and two rami: the postorbital ramus and the quadratojugal ramus. The postorbital ramus of the jugal, together with the main body of the jugal, form the ventral and posteroventral margins of the orbit. The jugal proportions differ between *M. carinatus*, *P. erlenbergiensis* and *L. huenei*. In *M. carinatus* and *P. erlenbergiensis*, the jugal is more gracile with a ratio of the minimum dorsoventral height of the jugal below the orbit to the distance between the anterior end of the jugal and the anteroventral corner of the infratemporal fenestra being less than in *L. huenei*.

The postorbital ramus projects posterodorsally from the main body of the jugal. This is the mediolaterally thickest of the two rami and it forms a long, overlapping contact with the posteromedial surface of the jugal ramus of the postorbital. The lateral surface of this ramus is convex and smooth and the medial surface is flat and smooth. In *P. erlenbergiensis*, the dorsomedial surface of the postorbital ramus of the jugal bears a deep, elongated fossa, representing the postorbital articular facet, on the posterior half of the ramus. This fossa is at its deepest posteriorly and is posteriorly and ventrally bordered by scarp-like ridges of bone.

In *M. carinatus*, the quadratojugal ramus of the jugal extends posteriorly from the jugal main body. It is a mediolaterally thin and dorsoventrally short strut of bone that forms an overlapping contact with the lateral surface of the jugal ramus of the quadratojugal. It has smooth and flat medial and lateral surfaces. In *P. erlenbergiensis*, the posteromedial surface of the quadratojugal ramus of the jugal bears a deep, elongated fossa, representing the quadratojugal articular facet, on the posterior half of the ramus. This fossa is at its deepest anteriorly and is fully bordered by scarp-like ridges of bone. In *M.* carinatus, the quadratojugal ramus of the jugal, along with the postorbital ramus, form the anteroventral and ventral margins of the infratemporal fenestra. These two rami meet at a 40° angle.

The main body of the jugal gradually tapers anteriorly to form a mediolaterally compressed process that is anteroposteriorly oriented. In *L. huenei*, this process is proportionally dorsoventrally higher and anterodorsally inclined. In *M. carinatus*, the jugal process of the lacrimal contacts the distal end of the dorsal margin of the anterior process of the jugal. This contact is a linear, posterodorsally inclined contact. It forms a 45° angle with the anteroposterior axis of the skull. The jugal ramus of the lacrimal also contacts the dorsal portion of the medial surface of the anterior process of the jugal. This articular surface is flat and rugose. The contact with the maxilla extends along the ventral margin of the main body of the jugal. The contact is oriented horizontally in lateral view, but it rises abruptly as it extends anteriorly, ultimately forming a 38° angle with regard to the horizontal axis of the skull. The main body of the jugal is medially and laterally overlapped by the medial and lateral portions of the maxilla, which form the posterior alveolar region. The maxillary articular surface on the jugal is smooth and indistinguishable from the rest of the surface on which it occurs.

The lateral surface of the jugal body is flat and smooth. The jugal bears a large ovoid fossa on the posterior portion of its medial surface (Fig. 19: f1). The long axis of the fossa is anteroposteriorly oriented and it originates at the posterior margin of the jugal, where the postorbital and quadratojugal rami meet. The fossa is deepest at its anterior end. Its dorsal margin is formed by a ridge of bone (Fig. 19: ri1) that extends along the dorsal margin from the anterior half of the medial surface of the postorbital process until the posterior half of the medial surface of the jugal body where it grades into the medial surface of the main body. In *P. erlenbergiensis*, this fossa is dorsally and ventrally bordered by scarp-like ridges of bone.

#### Quadratojugal

Both quadratojugals are preserved and undistorted. The left quadratojugal is complete whereas the right quadratojugal is missing some bone. The quadratojugal is mediolaterally thin, with an anteriorly opening, ‘V’-shape in lateral view. CT scans show that the internal structure of the bone is solid. It contacts the jugal anterolaterally, the squamosal dorsally and the quadrate posterolaterally (Figs. 20 and 21). It comprises a squamosal ramus and a jugal ramus. These rami meet at the quadratojugal angle at approximately a 75° angle to each other. In *L. huenei* the jugal and squamosal rami of the quadratojugal are separated by a 45° angle and in *P. erlenbergiensis* they are subparallel. A short posterior process extends posteriorly in *M. carinatus* from the quadratojugal angle, forming a tab that curls posteromedially to wrap around the ventral half of the lateral margin of the quadrate.

The jugal ramus projects anterodorsally and forms an overlapping contact with the quadratojugal ramus of the jugal. The posterior end of this contact occurs anterior to the middle of the ventral margin of the infratemporal fenestra in *M.* carinatus, whereas in *L. huenei*, it extends up to the midline of the ventral margin. In *M. carinatus* this contact is 17mm in length (approximately half of the jugal ramus’ length) and it terminates slightly posteroventrally to the anteroventral corner of the infratemporal fenestra. There is a slight gap in this specimen between the jugal ramus of the quadratojugal and the quadratojugal ramus of the jugal. There is therefore no pronounced articulation facet on either of the bones. The dorsal margin of the ramus in *L. huenei* is convex whereas it is linear in the other taxa. The lateral and medial surfaces of this ramus are flat and smooth.

The squamosal ramus extends anterodorsally and contacts the quadrate along the ventral portion of the medial surface of the quadratojugal ramus of the quadrate. It also forms a butt joint contact with the quadrate ramus of the squamosal. This contact occurs at midheight of the posterior margin of the infratemporal fenestra. The jugal process is 30% longer than the squamosal ramus. The posterior margin of the ramus is straight and the anterior margin is convex. In *P. erlenbergiensis* the squamosal ramus extends anteriorly and has a concave dorsal margin and a convex ventral margin. The lateral and medial surfaces of this ramus are flat and smooth.

The posterior process forms the apex of the quadratojugal, where the two rami join. The medial surface of this process contacts the lateral surface of the ventral portion of the quadrate shaft (just above the condyles). The medial surface of this process is rugose and slightly concave. The lateral surface is smooth and bears no salient features. In *P. erlenbergiensis*, the posterior process is proportionally anteroposteriorly longer.

#### Quadrate

Both quadrates are complete, well-preserved and undistorted. The quadrate forms the posterolateral margin of the skull and would articulate with the mandible although the latter is not present in BP/1/5241 (Figs. 22 and 23). CT scans show that the internal structure of the bone is solid, except for the condyles which are composed of trabecular bone. It contacts the squamosal anterodorsally, the quadratojugal anterolaterally, and the pterygoid medially. The quadrate comprises a quadrate head, a main shaft, a lateral quadratojugal ramus and a medial pterygoid ramus. The quadrate foramen is formed from a lenticular separation between the ventral portion of the anterior margin of the quadratojugal ramus and the center of the posterior margin of the quadrate ramus of the quadratojugal.

In posterior view, the ventral third of the medial margin of the quadrate shaft is dorsoventrally oriented whereas the dorsal two thirds are dorsolaterally oriented, forming an angle of approximately 135°. This margin is medially concave on its dorsal two-thirds. In posterior view, the medial margin of the quadrate shaft in *P. erlenbergiensis* and *L. huenei* is sinuous with a convex ventromedial margin and a concave dorsomedial margin. The posterior surface of the quadrate shaft is dominated by a hemi-cylindrical ridge (hereafter: ‘quadrate ridge’), that extends along the medial margin from the quadrate head to the medial quadrate condyle. A shallow, dorsoventrally elongate fossa is present on the ventral half of the posterior surface, lateral to the quadrate ridge (Figs. 22 and 23: f1). The bone surface on the floor of this fossa is rugose. This fossa tapers medially and laterally and deepens dorsally. The anterior surface of the shaft is concave, and the degree of concavity varies from broad and shallow immediately dorsal to the condyles to a deeply incised groove between the quadratojugal and pterygoid rami (Fig.22: gr2). The medial surface of the quadrate shaft is formed entirely by the quadrate ridge, and bears no foramina or other features. In lateral view, a second ridge is located anterior to the posterior fossa and extends dorsally from the lateral margin of the lateral condyle and grades into the ventral base of the quadratojugal ramus. The ventrolateral half of this ridge is overlapped by the posterior process of the quadratojugal. In *P. erlenbergiensis*, the articular surface for the posterior process is formed by a deep dorsoventrally oriented groove that extends along the ventral half of the lateral surface of the quadrate shaft. This groove is at its deepest dorsally and is bounded by scarp-like ridges.

The quadrate head articulates within a pronounced cotyle on the ventral surface of the squamosal. It is mediolaterally compressed and reniform in dorsal view, with the medial margin being concave and the lateral margin convex. The long axis of the quadrate head is angled anteromedially in dorsal view. Its articular surface is shallowly convex. The outlines of the medial and lateral condyles in ventral view are both semicircular.

The condyles are separated by a deep, anteromedially oriented intercondylar groove (Fig. 22: gr1). The sub-ovoid medial condyle is anteroposteriorly longer than the subtriangular lateral condyle but both condyles are sub-equal in mediolateral width. The posteromedial margin of the medial condyle extends further ventrally than the lateral condyle. The lateral and medial condyles extend to the same ventral level in *P. erlenbergiensis*. The condyles of *S. aurifontanalis* are very smooth and poorly defined. In *L. huenei* the ventral margin of the quadrate extends ventral to the level of the maxillary tooth row, leading in a ventral offset for the craniomandibular articulation, as per Barrett, Upchurch & Wang (2005). This could be exaggerated by deformation. In anterior view, a pronounced lip of bone marks the junction between the articular surface of the condyles and the anterior surface of the quadrate shaft in *M. carinatus*. This lip is oriented mediolaterally, but is sinuous and the medial half is dorsally concave, exposing the articular surface of the medial condyle in anterior view.

The pterygoid ramus of the quadrate extends anteriorly as a mediolaterally thin sheet of bone from the dorsal two-thirds of the medial margin of the quadrate shaft, forming a 90° angle with the latter. The pterygoid ramus forms an extensive articulation with the quadrate ramus of the pterygoid along its entire anterior margin. In lateral view, the anterior margin of the ramus forms a dorsoventrally elongated ‘S’-shape. The ventral margin of the ramus is separated by a semicircular notch from the quadrate body, and the margin of this notch is demarcated by a small ridge of bone on the pterygoid ramus. In lateral view the dorsal end of the anterior margin of the pterygoid ramus grades gradually into the quadrate head. The ventral portion of the pterygoid ramus flares slightly laterally as it extends anteriorly, so that the medial surface of the ventral half is visible in anterior view. The medial surface is concave, and this concavity extends as a broad, shallow fossa bordered posteriorly by the quadrate shaft. This fossa is continuous with a similar fossa present on the medial surface of the quadrate ramus of the pterygoid. The lateral surface is slightly convex.

The quadratojugal ramus extends anteriorly from the dorsal half of the lateral margin of the quadrate shaft, forming a 90° angle with the latter. The pterygoid and quadratojugal rami are therefore parallel to one another. The angle between the pterygoid and quadratojugal rami in *L. huenei* is near 90°. The ramus is oval shaped in lateral view, with a shallowly convex anterior margin. Almost the entirety of the anterior margin of the quadratojugal ramus articulates within a slot on the posterior surface of the quadrate ramus of the squamosal. The ventral margin and a small portion of the anteroventral corner of the medial surface of the ramus form a lap joint articulation with the quadrate ramus of the quadratojugal. The lateral surface of the ramus is smooth and slightly convex and the medial surface is flat and smooth. The lateral surface of the quadratojugal ramus of *S. aurifontanalis* is concave.

### Palate

The palate comprises the vomers, palatines and pterygoids (Fig. 24).

#### Pterygoid

Both pterygoids are preserved and although they are undistorted, they are also incomplete. The pterygoid is a complex bone that forms the posterior part of the palate (Figs. 25 and 26). CT scans show that the internal structure of the bone is solid. It contacts the quadrate posterolaterally, the basisphenoid ventromedially, the palate dorsally and the vomers anteriorly. It can be subdivided into three sections: the quadrate ramus, a central region and the palatal ramus.

The quadrate ramus is a large lamina of bone that extends posterolaterally from the main central body of the pterygoid. It is connected to the latter by a constricted, neck-like structure that projects from the sub-terminal region of the posteroventral margin of the pterygoid body. The quadrate ramus of *P. erlenbergiensis* is proportionally lower and is separated from the pterygoid main body by an anteroposteriorly shorter neck. In *M. carinatus*, this lamina is posteriorly subdivided into three processes (Figs. 25 and 26: p1, p2, p3). The dorsalmost process projects dorsally from the anterior margin of the lamina, forming a continuation of the latter (Figs. 25 and 26: p1). This process is most certainly the epipterygoid, however no suture could be found. It is approximately the same height as the quadrate ramus main body. The second and third processes contact the pterygoid ramus of the quadrate. The anteriormost of the two processes (Figs. 25 and 26: p2) extends out from the dorsal margin of the quadrate ramus and forms a 45° angle with the vertical axis of the skull. It medially overlaps the pterygoid ramus of the quadrate and tapers at the base of the quadrate head. The third process (Figs. 25 and 26: p3) extends posteriorly from the ventral margin of the quadrate ramus. It is the smallest of the three processes and is tab like rather than tapering in morphology. In posterior view, these posterior processes extend ventromedially, forming a cup-like shape. The quadrate ramus is concave along its medial surface and convex along its lateral surface. Both surfaces are slightly rugose.

Contra Sues et al. (2004), the pterygoid does possess a ‘hook-like medial ramus around the basipterygoid process,’ as in *Plateosaurus* (Sues et al., 2004, p. 250). This ramus extends posteriorly from the posteromedial surface of the pterygoid body. When viewed in ventral view, this basisphenoid ramus, along with the ventral margin of the quadrate ramus, lie in the same plane and form a hook-like structure. This hook cups the basipterygoid process of the basisphenoid at its midheight. The tab-like basisphenoid ramus of the pterygoid extends slightly dorsomedially between the basipterygoid processes. This basisphenoid ramus has a smooth, slightly convex medial surface and a smooth flat lateral surface. In *S. aurifontanalis*, the medial process that hooks around the basipterygoid process is mediolaterally oriented and anteroposteriorly shorter than in *M. carinatus*.

The palatal ramus of the pterygoid is incomplete on both sides. However, it is possible to observe that its remains form a lamina of bone that project ventrolaterally from the posterior end of the central region of the pterygoid. On the right side of the skull, the palatine slightly contacts this ramus by overlapping it dorsally. The surfaces of this palatal ramus are smooth.

The central region of the pterygoid comprises a mediolaterally compressed lamina of bone that extends anteriorly and tapers where it contacts the pterygoid process of the vomer. The surfaces of this lamina are smooth. Its lateral surface is slightly concave. The medial surfaces of the left and right laminae are subparallel to one another and almost contact each other. Together they form a medial crest that forms a 70° angle with the vertical axis of the skull. The main body of the pterygoid of *S. aurifontanalis* extends farther medially than that of *M. carinatus*. In *L. huenei*, the pterygoid possesses a laterally extending flange of bone that projects from the main body anterior to the quadrate ramus and almost contacts the medial surface of the jugal. Anterior to this process is the anterolaterally projecting ectopterygoid ramus of the pterygoid. *M. carinatus* does not bear the lateral rectangular processes of the pterygoid, however this could be a preservation issue. The pterygoid of *P. erlenbergiensis* also possesses an extra process that articulates with the ectopterygoid. These features have no obvious homologues on the skull of *M. carinatus* where the pterygoid lateral margins are level with those of the basipterygoid processes.

#### Ectopterygoid

Both ectopterygoids are disarticulated from the rest of the palatal complex and only the right one is complete (Fig. 27). CT scans show that the internal structure of the bones is solid. The ectopterygoid is a hook-shaped bone that connects the palate to the lateral bones of the skull. It comprises an anteroposteriorly compressed, curved jugal ramus and a dorsoventrally compressed, anteroposteriorly expanded pterygoid ramus. The entire cortical surface of the ectopterygoid is smooth.

The pterygoid ramus forms the proximal base of the ectopterygoid. It has a slightly convex ventral surface and a concave dorsal surface. The posterior portion of the expanded pterygoid ramus is anteroposteriorly longer than the anterior portion. The medial margin of this ramus is crenulated and corresponds to where the ectopterygoid would articulate with the lateral margin of the pterygoid in life position.

The jugal ramus is formed by a strip of bone that is dorsoventrally compressed at its proximal end and twists as it extends distally to have a mediolaterally compressed distal end. This ramus tapers to a point. It has a convex anterior margin and a concave posterior margin, giving it its hook shape. The articular surface for the jugal is on the lateral surface of the distal end of the ramus but is not clearly distinguishable from the rest of the surface. Compared to that of *M. carinatus*, the ectopterygoids of both *S. aurifontanalis* and *L. huenei* have a shallower radius of curvature. In *P. erlenbergiensis*, the distal end of the jugal ramus of the ectopterygoid is autapomorphically expanded mediolaterally to form a T-shaped articulation with the jugal.

#### Palatine

Both palatines are preserved but incomplete. The palatine forms the middle component of the palate between the pterygoid and vomers, and is situated laterally, such that the two palatines do not meet on the midline (Figs. 28 and 29). CT scans show that the internal structure of the bone is solid. The palatine forms the posterior margin of the choana, and contacts the jugal dorsolaterally, the maxilla ventrolaterally, the pterygoid posteriorly, and the vomer anteriorly (although it is slightly separated in this specimen). The palatine comprises three main rami, the vomerine ramus, the pterygoid ramus, and the jugomaxillary ramus. The right palatine is missing the vomerine ramus. Breakage of the medial margin of the left palatine between the vomerine and pterygoid rami makes it impossible to tell if these rami were mesially connected by a sheet of bone.

The vomerine ramus extends anterodorsally at approximately a 105° angle to the vertical axis of the skull. The vomerine process is attached to the palatine body by a distinct mediolaterally compressed neck of bone. The distal end of the vomerine process is strongly mediolaterally compressed into a vertically oriented sheet of bone. This sheet appears to fork into a dorsal and ventral process as it extends distally (Fig. 29: p1 and p2 respectively); however, the bone is very thin in this region and may be below the resolving power of the CT scan. The lateral surface of the vomerine process bears two parallel ridges: the more anterior ridge begins at the anterior margin of the ventral process, continues ventrally along the anterior margin of the neck, and grades into the lateral surface at the base of the latter; the second ridge extends from halfway up the posterior margin of the dorsal process and ventrally down the lateral surface of this process to grade into the neck of the dorsolaterally extending process of the jugomaxillary ramus. Posterior to the second ridge, the vomerine ramus bears a shallow groove (Fig. 29: gr1). The lateral and medial surfaces of the vomerine ramus are smooth. The medial surface of the vomerine process bears a ridge along its ventral margin (Fig. 29: ri1), which begins at the ventral process of the distal end and continues posteroventrally to grade into the neck. This is possibly for muscle attachment.

The jugomaxillary ramus is a complex structure. The base of this ramus is located at the junction of the vomerine and pterygoid rami. The ventral surface of the base bears a ventrally opening, anteroposteriorly oriented groove that forms a contact with the medial surface of the posterior end of the maxilla (Fig. 29: gr2). The medial margin of this groove is ventrally more extensive than the lateral margin and is formed by a mediolaterally thin sheet of bone, which projects ventrally. The lateral margin of this groove forms a low lip of bone, the central portion of which is ventrally more extensive than the anterior and posterior portions. The contact between the palatine and maxilla is more posteriorly positioned in *L. huenei* than in *M. carinatus*. In *L. huenei*, the palatine possesses a laterally extending process that is greatly anteroposteriorly expanded at its distal end and which contacts the ventral surface of the maxilla. In *M. carinatus*, a process extends dorsolaterally from the lateral surface of the base of the jugomaxillary ramus (Figs. 28 and 29: p3). This process expands distally into a bulbous structure that likely contacted the medial surface of the maxillary ramus of the jugal in life position, but as preserved these bones are separated on both sides of the skull. This process is a possible autapomorphy for *M. carinatus*, however this is difficult to confirm due to a poor sample of well-preserved sauropodomorph palates. All the surfaces of this jugomaxillary ramus are smooth.

The pterygoid ramus is a dorsoventrally compressed sheet of bone, whose mediolateral axis is oriented subhorizontally, although the exact angle cannot be determined because both palatines are not in life position. The contact with the pterygoid is poorly preserved, but would have likely been a simple overlapping contact of thin lamellar bone. The lateral surface of the pterygoid ramus is shallowly concave and the medial surface is slightly convex. Both of these surfaces are smooth.

#### Vomer

The fused vomer is a complex bone that forms the anterior portion of the palate (Figs. 30 and 31). Both vomers appear to be complete. The vomer is a mediolaterally compressed, thin sheet of bone that tapers both anteriorly and posteriorly. CT scans show that the internal structure of the bone is solid. It contacts the premaxilla anteriorly and the pterygoid posteriorly. The vomer comprises two main rami: the anterior premaxillary ramus and the posterior pterygoid ramus. It has an overall sinusoidal shape in lateral view with the dorsal margin of the premaxillary ramus being concave and the dorsal margin of the pterygoid ramus being convex. The medial surfaces of the opposing vomers are subparallel to each other. Posteriorly, the two vomers form a continuation of the anterior rami of the pterygoid, and are medially unfused until approximately one third of their length where they then become fused. The contact with the palatine is not preserved.

The pterygoid rami of the vomers are arced dorsally with a deeply concave ventral margin (maximum depth of 20 mm). Together, the anterior rami of the pterygoid and the posterior portion of the vomers form an arch that extends dorsally until the midheight of the skull. The posterior portion of the vomer is also the highest part of the sheet of bone. The lateral and medial surfaces of this portion are slightly rugose.

The anterior portion of the vomer has a shallowly concave dorsal margin, giving the vomers a slight S-shape in lateral view. There is no dorsal expansion of the vomers anteriorly, however there is a slight lateral flaring. The dorsal margin of this flaring is concave. The tapered anterior portion contacts the posterior margin of the medial expansion of the premaxilla. A posterodorsal process projects out from the anteriormost end of the vomer (Fig. 31: p1). This process is flat and extends posterodorsally until it contacts the ventral margin of vomer’s main body approximately halfway through its length. This forms a foramen when viewed laterally (Fig. 31: fo1). This portion of the vomer is smooth on all its surfaces.

### Neurocranium

The neurocranium comprises the frontals, parietals, basioccipital, basisphenoid, orbitosphenoids, laterosphenoids, prootics, exoccipitals and supraoccipital (Figs. 32 and 33).

#### Frontal

The fused frontals are complete, well-preserved and undistorted. They form a thick sheet of bone that roofs most of the posterior end of the skull and which forms the dorsal margins of the orbits (Figs. 34 and 35). CT scans show that the internal structure of the bones is solid. The frontal contacts the nasal anteriorly, the prefrontal anterolaterally, the postorbital posterolaterally and the parietal posteriorly. The frontal is comprised of a subtriangular main body and two rami: the prefrontal and the postorbital ramus. All the surfaces of the frontal are smooth, except for the postorbital articular surface, which is rugose.

The prefrontal ramus is a tab-like structure with a rounded distal margin. It extends anterolaterally from the anterior region of the lateral margin of the frontal, forming a posteromedially extending notch in dorsal view (Fig. 34: no1). The dorsal surface of the frontal bears an ovoid depression that extends posteromedially from the posterior margin of this notch and terminates just prior to the mid length of the frontal. This notch and depression are where the frontal ramus of the prefrontal dorsally overlap the frontal.

The postorbital ramus is approximately 60% longer than the prefrontal ramus, and it extends laterally from the posterior portion of the frontal. This ramus is triangular in dorsal view, lending a strong lateral flare to the posterior portion of the frontal. The posterior half of the postorbital ramus slopes posteroventrally. This surface is dorsally overlapped by the frontal ramus of the postorbital. Contra Sues et al. (2004), this contact is not interdigitating posteriorly but is clearly visible as a lap joint along its entire length. This contact results in the exclusion of the postorbital in the dorsal margin of the orbit. *M. carinatus* has a shallow postorbital articular surface. In *P. erlenbergiensis*, the frontal has a deep articular facet for the postorbital frontal ramus. In *P. erlenbergiensis*, this articular facet is formed by a U-shaped notch opening anterolaterally and the floor of this articular surface is formed by a small shelf of bone that extends laterally, below the level of the dorsal surface.

Medially adjacent to the postorbital articular surface is the frontal contribution to the anteromedial corner of the supratemporal fossa, which is weakly defined. In *P. erlenbergiensis*, *S. aurifontanalis* and in *L. huenei*, the supratemporal fossa is more deeply excavated on the posterolateral portion of the dorsal surface. The frontal does not contribute to the supratemporal fenestra margin in any of these taxa.

The prefrontal and postorbital rami are dorsally upturned and dorsally arced in lateral view, forming the dorsal margin of the orbit. This dorsal orientation of the lateral rami render the frontal transversely concave. The dorsal surface is deepest in the center of the frontal, in the center of the intertemporal region.

The main body of the frontal (the interorbital region) lies in the same plane as the main skull axis. An anteroposteriorly extending crest is present on the dorsal surface of the frontals along the presumed line of fusion between them. This crest extends slightly further anteriorly than the anterior margin of the frontal, forming a minor rectangular anterior projection that extends from the middle of the anterior margin of the frontal. The posterior margin of the frontal is linear in dorsal view. This entire margin contacts the anterior margin of the parietal in an interdigitating contact. The frontal is dorsally overlapped along its anterior margin by the nasal. This contact extends approximately 7 mm posteriorly. Only the lateral portions of this contact are present due to poor preservation of the medial regions of both the nasal and the frontal (Figs. 34 and 35: no2).

The frontal bears a deep hourglass-shaped depression that extends anteroposteriorly along its ventral surface. The anterior region of this depression is approximately 25% longer than the posterior region. The mid-section of the depression (the olfactory depression) is groove-like and its margins are subparallel. This is also where the depression is the shallowest. In *M. carinatus*, this mid-section of the depression is proportionally mediolaterally narrower than that of *E. minor*. In *M. carinatus*, this groove then opens into the posterior portion of the hourglass-shaped depression (the deeper cerebral cavity). This portion of the depression is continuous with the recess present on the ventral surface of the parietal (both bones participate equally to the cerebral depression). The posterior portion of the frontal depression bears a small subterminal fossa on either side of the midline. This is where the frontal ramus of the laterosphenoid articulates with the frontal. This hourglass-shaped depression is laterally bordered by an acute ridge of bone that originates in the prefrontal notch, it arcs medially and tapers in the lateral corner of the posterior margin of the frontal (Fig. 34: ri1). Lateral to this ridge of bone are the prefrontal and postorbital rami, which extend dorsally.

#### Parietal

The fused parietals are complete, well-preserved and undistorted. They form the posterodorsal roof of the skull, between the supratemporal fenestrae (Fig. 36). The midsagittal axis of the parietals is horizontal in lateral view. Each parietal contacts the frontal along its anterior surface, the laterosphenoid along its anteroventral surface, the supraoccipital along its posteroventral and posterior surfaces and the squamosal along its posterolateral surface. All the surfaces of the parietal are smooth except for the anterior half of the ventral surface, which is slightly irregular. The parietal comprises a main body and a squamosal ramus.

The squamosal ramus of the parietal forms a posterolaterally flaring posterior projection that is triangular in cross section with the apex oriented dorsolaterally. In *P. erlenbergiensis* this ramus tapers to a point. In *M. carinatus*, its anteroposterior length is equivalent to half of that of the entire parietal anteroposterior length and its slopes posteroventrally. In *L. huenei*, the squamosal ramus of the parietal is more laterally oriented. In *M. carinatus*, the posterior margins of these two projections together form an anteriorly pointing, ‘V’-shape in dorsal view whose anterior apex overlies the supraoccipital. An anteromedially extending bony ridge is present along the midline of the dorsal surfaces of each posterolateral projection. The left and right ridges meet at the midline of the skull where they taper into the sagittal crest. The exterior edge of the posterolateral process forms the posteromedial margin of the supratemporal fenestra. The ventral portion of lateral surface of this process bears a triangular fossa that extends from the posterior margin of the process. The floor of this fossa is rugose. This is where the medial surface of the parietal ramus of the squamosal overlaps the posteroventral surface of the squamosal ramus of the parietal. The medial surface of the ramus is slightly convex and smooth.

The parietals narrow substantially where they form the medial margins of the supratemporal fenestrae. Anteriorly, the parietal flares out into a laterally extending process with a rounded distal margin. This process does not extend as far laterally as the squamosal ramus. The anterior margin of the fused parietals is convex and corresponds with the concavity on the parietal contact of the frontal. In *E. minor*, the parietal appears to have a concave anterior margin and the frontal a convex posterior margin. In *M. carinatus*, this contact is a thin, irregular, well-sutured contact, oriented horizontally in anterior view and transversely in dorsal view. In *L. huenei*, this process is proportionally mediolaterally wider and anteroposteriorly shorter than in *M. carinatus*. This affects the shape of the long axis of the supratemporal fenestra, which appears to be transversely oriented in *L. huenei*, whereas in *M. carinatus* they are anteroposteriorly oriented. The dorsal surface of the parietal is smooth in *M. carinatus*. On the dorsal surface of the fused parietals, along the symphysis, a low ridge of bone is present, forming a midsagittal crest that extends along the entire anteroposterior length of the dorsal surface of the parietals. The midsagittal crest is more pronounced in *L. huenei* than in *P. erlenbergiensis* and *S. aurifontanalis.* The ventral surface of the parietal is slightly rugose. The laterosphenoid contact originates in the middle of the ventrolateral surface of the parietal and diverges anterolaterally outwards to follow the shape of the anterior parietal flaring. The supraoccipital contact is a mediolaterally wide suture which occurs along the posteroventral surface of the parietal and extends to the posterodorsal surface as the supraoccipital takes up the space between the two posterior parietal wings.

#### Basioccipital

The basioccipital is complete, well-preserved and undistorted. The basioccipital forms the posterior portion of the floor of the braincase (Figs. 37 and 38). The long axis of the basioccipital from the occipital condyle to the basal tubera runs along the midline of the skull and is oriented subhorizontally. The basioccipital contacts the exoccipitals on its dorsal surface and the basisphenoid on its anterior and anterolateral surfaces. The basioccipital comprises the occipital condyle posteriorly, the basal tubera anteriorly and the floor of the foramen magnum dorsally.

The condyle is demarcated by a lip-like extension of its articular surface that projects both laterally and ventrally. It is separated from the basal tubera by a distinct neck, set off from the condyle by a semicircular notch that bears emarginations on its lateral and ventral surfaces. The ventral margin of the basioccipital neck of *S. aurifontanalis* is linear (i.e. lacks the semicircular notch of *M. carinatus*), and it forms a right angle with the posterior surface of the basal tubera which extend ventrally. In *P. erlenbergiensis*, the ventral margin of the occipital condyle lies above the level of the distal tip of the cultriform process whereas in *M. carinatus*, it lies slightly below the level of the cultriform process. In *M. carinatus*, the condyle is not mediolaterally wider than the neck in ventral view. In *E. minor*, the condyle appears to be expanded and is mediolaterally wider than the neck in ventral view.

The ventrolaterally diverging basal tubera are formed equally by the basisphenoid and basioccipital, with the basioccipital forming the posterior half. The basioccipital contribution to the basal tubera is narrowly separated by a small ‘U’-shaped notch (Fig. 37: no1). Lateral to the notch, on the ventral side of the posterior surfaces of the basal tubera, there is a crescent-shaped ridge that is separated along the sagittal plane (Fig. 37: ri1). This separation is aligned with the medial separation of the basal tubera. The ridge is absent in *S. aurifontanalis*. In *E. minor*, the basioccipital contribution to the basal tubera comprises a ridge of bone that is rectangular in shape when viewed posteriorly and extends along the contacts between the basisphenoid and basioccipital, on the ventral surface of the latter. In *M. carinatus*, in lateral view, the basal tubera are oriented posteroventrally and follow the slight hook shape of the occipital condyle. The vertically oriented contact with the basisphenoid is a well-sutured butt joint. The surface of the contact on the basioccipital is shallowly convex and matches the posterior surface of the basisphenoid. This articular surface is very rugose.

The dorsal surface of the basioccipital is mostly occupied by the neural groove, which medially separates the left and right exoccipital articular surfaces. The exoccipital contact is a well-sutured butt joint. This articular surface is therefore highly irregular. In lateral view, the suture is horizontally oriented. It lies on the lateral margins of the dorsal surface of the basioccipital body and curves out anterolaterally on this surface. In posterior view, the contact is angled dorsomedially such that the lateral margin of the contact is lower than the medial margin. The occipital condyle contributions of the exoccipitals flare out ventrally to overlap with the basioccipital and form a continuation of the occipital condyle articular surface. The metotic strut of the exoccipitals also contact the basioccipital on the dorsolateral corner of its anterior surface.

#### Basisphenoid–parasphenoid

The basisphenoid is complete, well-preserved and undistorted. It is lying in life position along the ventral surface of the midline of the skull (Figs. 39 and 40). The long axis of the basisphenoid, which extends from the basal tubera to the tip of the cultriform process, is orientated subhorizontally, slightly sloping posteriorly. Internally, CT scans reveal that the basisphenoid is highly trabeculated, especially in the basipterygoid processes. There is also a pneumatic pocket linking the lateral foramina to the posterior one (Fig. 40). The basisphenoid contacts the basioccipital on its posterior surface, the prootic along its dorsal surface and the pterygoid at the ventral surface of its basipterygoid processes. It is formed equally by the parasphenoid and basisphenoid bodies which are fused and indistinguishable from each other. It comprises a basisphenoid body, basal tubera, basipterygoid processes and a cultriform process.

The basisphenoid/prootic contact is a well-sutured butt joint. It is primarily horizontally oriented although a shallow depression in the dorsal surface of the basisphenoid gives the contact a saddle shape. This articular surface is highly irregular.

The basal tubera, located on the posterior end of the ventral surface, are formed equally by the basisphenoid and basioccipital. The basal tubera are widely separated from each other along the midline by a deep, U-shaped notch. The basal tubera are developed as subtetrahedral protuberances with rounded apices pointing mostly ventrally and slightly posteriorly and laterally. The basal tubera of *P. erlenbergiensis* radiate into four separate ventral projections. These ventral projections are not visible in *M. carinatus*. In the latter, on the anteroventral surfaces of the basal tubera, a weak bony ridge extends from the ventral apex and grades into the lateral wall of the basisphenoid recess (Fig. 39: ri1). Their posterior surfaces corresponds to the articular surface for the basioccipital contribution of the basal tubera. This contact is a firmly sutured butt joint. This contact is vertically oriented in lateral view and mediolaterally oriented in ventral view. The basioccipital contact surface of the basisphenoid is shallowly concave and irregular. The lateral surfaces of the basal tubera are slightly convex and smooth.

The basipterygoid processes are situated on the ventral surface of the basisphenoid. These are medium-length finger-like projections, are ovoid in cross section, and project primarily ventrally but also slightly anteriorly and laterally. They diverge from one another at an angle of approximately 35°, not at the 100° angle noted by Barrett (2004), and are approximately twice the dorsoventral height of the basal tubera. The angle between the basipterygoid processes is closer to 60° in *P. erlenbergiensis* and 70° in *E. minor*. In *M. carinatus*, the distal ends of the basipterygoid processes are strongly convex and subhemispherical. Their anterior surface bears a ridge that extends anterodorsally from their ventralmost tip to grade into the cultriform process (Fig. 40: ri2). The posterior surfaces of the basipterygoid processes do not bear any salient features and are smooth. In lateral view, the basipterygoid processes are well separated from the basal tubera by an anteroposteriorly long U-shaped notch. In *P. erlenbergiensis*, the ventral margin of the basal tubera form a near right angle with the posterior margin of the basipterygoid processes in lateral view. This leads to the appearance of the ‘stepped’ braincase in *P. erlenbergiensis.* In *S. aurifontanalis* and *E. minor*, the basal tubera and basipterygoid processes are separated by a much wider angle, making the ventral margin of the basisphenoid more linear. In *M. carinatus*, the lateral surfaces of the processes are smooth, except for a rugose irregular patch on the ventral portion of the lateral surface that corresponds to the articular surface for the basisphenoid ramus of the pterygoid. The basipterygoid processes form a ball and socket joint with the pterygoid (respectively). The bases of the basipterygoid processes are connected medially by a low web of bone which forms the anterior wall of the basisphenoid recess (Figs. 39 and 40: ri3). The medial surfaces are smooth.

The cultriform process is an anteriorly tapering structure. It differs in relative length amongst early branching sauropodomorphs—the cultriform process of *M. carinatus* is 15% shorter than the combined anteroposterior length of the basioccipital and basisphenoid body whereas in *P. erlenbergiensis*, the cultriform process is 28% longer than the combined lengths of the basioccipital and basisphenoid. In *M. carinatus*, the cultriform process has a concave ventral margin and a convex dorsal margin, making it arced dorsally in lateral view. In *P.* erlenbergiensis, the dorsal margin of the cultriform process is horizontal and linear and the ventral margin is convex. The cross section of the cultriform process is V-shaped with the apex pointing ventrally. The dorsal surface of the cultriform process is composed of a groove that is proximally separated from the sella turcica by a dorsoventrally low wall of bone (Fig. 39: gr1). The proximal end of the ventral surface of the cultriform process bears a deep depression that becomes anteriorly shallower and gradually tapers (Fig. 39: f1). This fossa is proportionally deeper in *E. minor*.

In *M. carinatus*, the dorsal surface of the basisphenoid body has a deep circular fossa, the sella turcica (hypophyseal fossa), situated between the basisphenoid–prootic contact and the base of the cultriform process. The basisphenoid recess is positioned in the center of the ventral surface of the basisphenoid body. This recess is circular and deeply concave (deeper than in *P. erlenbergiensis* and *S. aurifontanalis*). It is formed laterally by a bony wall between the basipterygoid processes and the basal tubera and anteriorly by the bony ridge connecting the basipterygoid processes. Posteriorly, the basisphenoid recess is continued onto the ventral surface of the basioccipital as the notch separating the basioccipital contribution of the basal tubera (Fig. 33). This is different to *P. erlenbergiensis*, where a highly scarred mediolaterally oriented ridge of bone on the ventral surface separates the basioccipital basal tubera from the basisphenoid basal tubera. In *M. carinatus*, the lateral surfaces of the basisphenoid body bear deep and large elliptical fossae. The basisphenoid body has a hollow central chamber. This chamber communicates dorsally and anteriorly with the sella turcica, laterally with the depressions present on the lateral surfaces of the basisphenoid, and posteriorly with a foramen present on the right lateral side of the posterior surface (Fig. 40: fo1). Gow (1990) indicated that the carotids entered the sella turcica via a common foramen in *Plateosaurus*, and that this foramen was not present in juvenile *Massospondylus* but could appear with age. Our results confirm the presence of this common lateral foramen. It appears that the two carotid arteries enter the basisphenoid laterally via the foramina present on the lateral surfaces of the basisphenoid body, and then enter the sella turcica via a common foramen, although they are still mildly separated by a low septum.

Prieto-Márquez and Norell found that the dorsal surface of the anterior end of the basisphenoid body of *P. erlenbergiensis* extends dorsally into a bulb-like projection. We consider that this bulb-like projection was misidentified in *P. erlenbergiensis* and actually represents the ventral portion of the anterior end of the prootic. This therefore leads to changes in the position of the cranial nerve passages as reconstructed by Prieto-Márquez & Norell (2011). In *P. erlenbergiensis*, the basisphenoid is described as forming, from anterior to posterior, the ventral half of trigeminal foramen (V), facial foramen, fenestra ovalis, and the anterior half of fissure metotica. Comparison with the *M. carinatus* scans suggest that these foramina are more likely formed entirely by the prootic (see prootic description).

#### Orbitosphenoid

The fused orbitosphenoids are complete, well-preserved and undistorted. The orbitosphenoid forms the anterior wall of the braincase and is located anterior to the laterosphenoid (Figs. 42 and 43). CT scans reveal that the internal structure of the bone is hollow (Fig. 43). It contacts only the laterosphenoid posteriorly. The contact is divided into three small surfaces at the distal end of the posterior processes of the orbitosphenoid (Figs. 42 and 43: p1 and p2), which contact three anterior projections of the laterosphenoid. The orbitosphenoid bears two processes on its posterior surface and one on its anterior surface. All of the surfaces are smooth except for the articular surfaces for the laterosphenoid which are rugose.

The dorsalmost posterior process is the longest (Figs. 42 and 43: p1). Its length is equivalent to approximately half of that of the main body and it is directed posterolaterally in dorsal view, and posterodorsally in lateral view, forming a 90° angle with the long axis of the main body. These processes diverge from each other mediolaterally at a 90° angle in dorsal view. They form the anterior margin of the opening for the olfactory bulb (CNI) and the anteromedial processes of the laterosphenoid form the posterior margin.

The orbitosphenoid bears two ventrally oriented processes (Figs. 42 and 43: p2), which are approximately half the length of the total dorsoventral height of the ovale. These projections are directed posteromedially towards each other and almost meet along the midline of the skull. The anterior foramen formed by these projections is for the passage of the optic nerve (CNII). Although separated here, the posterior surface of the distal end of these processes would have probably contacted the anterior surface of the prootic rami of the laterosphenoids in life position.

The third contact with the laterosphenoid occurs halfway along the posterior surface of the orbitosphenoid. It is rugose and not clearly distinguishable from the rest of the surface.

In lateral view, the main body of the orbitosphenoid is ‘S’-shaped. Anteriorly, the orbitosphenoids meet at the midline and form a 91° angle between the two medial surfaces. The suture where the orbitosphenoids meet bears an anteriorly oriented projection (Figs. 42 and 43: p3). The three contacts between the orbitosphenoid and the laterosphenoid form two lateral foramina, with the orbitosphenoid contributing to the anterior margin and the laterosphenoid forming the posterior margin. The foramen formed by the dorsal posterior processes is the largest, with a diameter about three times the length of that of the foramen situated directly ventrally to it, formed by the middle laterosphenoid articular surface and the ventral posterior projections. The larger foramen is for the passage of cranial nerve IV whereas the one beneath it is for the passage of cranial nerve III.

#### Laterosphenoid

Both laterosphenoids are complete, well-preserved and undistorted (Figs. 45 and 46). The laterosphenoid forms the anterolateral walls of the braincase. CT scans show that the internal structure is composed primarily of trabecular bone. The laterosphenoid contacts the prootic posteriorly and ventrally, the supraoccipital posteriorly, the frontal anterodorsally, the parietal dorsally, the orbitosphenoid anteriorly and the postorbital laterally. It is a laterally compressed bone with several rami: a ventral prootic ramus, a posterior supraoccipital ramus, an anteromedial frontal ramus, an anterior orbitosphenoid ramus and a lateral postorbital ramus. All the rami are subequal in length, except for the orbitosphenoid ramus which is the shortest. The ventral surface of the laterosphenoid in *M. carinatus* does not contact the basisphenoid as in *P. erlenbergiensis* but it does contact the prootic. *E. minor* has a laterosphenoid that is more similar to that of *M. carinatus* than that of *P. erlenbergiensis*.

The prootic ramus is ventrally oriented and has a rectangular cross section. Its dorsoventral height is approximately one third of that of the entire laterosphenoid. The contact with the prootic on the ventral surface of the prootic process is a well-sutured butt joint. It is almost horizontal. The prootic also contacts the posteroventral surface of the posterior process. These two prootic contacts are medially separated by the notch present in the anterior surface of the prootic, which is where the laterosphenoid forms the anterior margin of the large trigeminal foramen (CNV).

The supraoccipital ramus tapers dorsomedially in lateral view. It has a convex posterior margin and a linear anterior margin. The lateral surface of this ramus is concave and smooth and the posteromedial surface contacts the anterodorsal surfaces of the supraoccipital. This is a small, loose horizontal contact, oriented transversely in ventral view. In *E. minor*, this supraoccipital ramus is posteriorly oriented and is proportionally anteroposteriorly longer and dorsoventrally lower than that of *M. carinatus*. In the former taxon, the supraoccipital ramus tapers to a point posteriorly and is therefore triangular in shape with the apex of the triangle pointing posteriorly.

The frontal ramus is a thin, splinter-like bone projection that is anterodorsally oriented in lateral view and anteromedially oriented in dorsal view. It tapers distally and has no salient features on its surfaces. The contact with the frontal is small and occurs on the distal end of the dorsal surface of the ramus. This ramus contact the posterior end of the ventromedial surface of the frontal. The anterior distal surface of this ramus also contacts the posterior surface of the dorsal posterior process of the laterosphenoid. In *P. erlenbergiensis*, this ramus only articulates with the frontal.

The postorbital ramus extends laterally and is rectangular in cross section. It has smooth surfaces, except for the distal surface which is rugose. There is a shallow groove on the ventral surface of this ramus. This groove is for the passage of the ophthalmic branch of the trigeminal nerve (CNV1). This groove is bound anteriorly and posteriorly by weakly developed bony ridges. The convex postorbital contact occurs on the distal end the ramus and is marked by a rugose surface. In *P. erlenbergiensis*, the postorbital process is proportionally anteroposteriorly much longer and curves anterolaterally.

The orbitosphenoid ramus is forked and comprises dorsal and ventral processes. These processes are of subequal length and anteroposteriorly short. They have rounded distal margins and smooth surfaces. The distal ends of these processes contact the posterior surface of the orbitosphenoid.

The laterosphenoid body of *M. carinatus* is more robust than that of *P. erlenbergiensis.* The anterior surface of the laterosphenoid is characterized by two distinct notches. The dorsalmost notch is bordered dorsally by the frontal process and ventrally by the orbitosphenoid process. This notch borders the posterior margin of the trochlear nerve (CNIV). The ventralmost notch is bordered dorsally by the orbitosphenoid process and ventrally by the prootic process. This notch forms the posterior margin of the occulomotor nerve (CNIII). In lateral view, the laterosphenoid is triangular with the apex pointing posterodorsally. The laterosphenoid medial surfaces are concave (to a greater degree than *P. erlenbergiensis*) where they form the walls of the cranial cavity. The dorsolateral surface of the laterosphenoid is concave and forms the anterior end of the medial margin of the supratemporal fenestra. The parietal contact is located on the dorsal surface of the laterosphenoid. It is horizontally oriented in lateral view and bends anterolaterally in dorsal view as it follows the anterior flaring of the parietal. In *P. erlenbergiensis*, the dorsolateral surface of the laterosphenoid is weakly convex whereas in *M. carinatus*, it is deeply concave.

#### Prootic

The prootics are complete, well-preserved, and lying in life position. The left and right prootic are separated along the midline contra Gow (1990), who stated that in adults the prootics were linked by a transverse bridge sutured to the dorsum sellae. The prootic is the central part of the lateral braincase wall (Fig. 47). CT scans show that the internal structure of the bone is composed primarily of trabecular bone. It contacts the basisphenoid on its ventral surface, the exoccipital on its posteromedial surface, the supraoccipital on its dorsal surface and the laterosphenoid on its anterior surface. The prootic is an irregularly shaped bone, with a larger posterodorsal process that is elliptical in lateral view and a smaller, foot-like anteroventral process.

These two processes are partially separated from each other by the foramen for the trigeminal nerve, which forms a deep posteroventrally oriented notch in the prootic. The anterior surfaces of the processes contact the laterosphenoid. The laterosphenoid contact is extensive, but is interrupted by the foramen for the trigeminal nerve (CNV). In lateral view this contact is sloped anteroventrally at about 45° relative to the horizontal axis of the skull. The contact forms a simple butt joint, with no interdigitation. CT scans reveal that the laterosphenoid contact shows no fusion or coossification.

The trigeminal foramen (CNV) lies at approximately mid height of the prootic in lateral view. The prootic forms all but the anterodorsal margin of the foramen, which is formed by the laterosphenoid. Two grooves extend from the trigeminal foramen onto the prootic (Fig. 47: gr1 and gr2). The topological similarities and phylogenetic congruence of several braincase bones and soft tissues in crocodyliform archosaurs suggests that these grooves represent the passage for the maxillary (CNV2) and mandibular (CNV3) branches of the trigeminal nerve (CNV) (Holliday & Witmer, 2009). The groove for CNV3 extends from the posterior margin of the trigeminal foramen (Fig. 47: gr2). It is oriented anteroposteriorly and runs along the lateral surface of the posterodorsal process of the prootic, and terminates at the base of the paroccipital process near the contact between the prootic and exoccipital. The groove for CNV2 is less defined (Fig. 47: gr1). It originates from the ventral margin of the trigeminal foramen and extends ventrally along the foot of the prootic, almost reaching the basisphenoid contact. The foramen for the facial nerve (CNVII) is located posterior and slightly ventral to that of the trigeminal nerve (CNV). It is an ovoid foramen with the long axis sloping anteroventrally at a 45° angle relative to the horizontal axis of the skull. The foramen for the facial nerve (CNVII) lies within a groove defined anteriorly by the crista prootica and posteriorly by a smaller, less defined crista (Fig. 47: c1). This groove slopes anteroventrally. In medial view, the passageway of the facial nerve can clearly be seen, extending ventral to the sacculus in the floor of the vestibular region.

The prootic body encloses the anterior and lateral semicircular canals of the inner ear. The anterior canal enters through the anterodorsal surface of the prootic and runs vertically through the bone. The canal expands before exiting the bone at approximately the mid-height of the posterior end of the medial surface of the prootic. As it expands, it joins the sacculus, although the exact delineation of the latter is unclear. The cavity for the anterior canal is connected to that of the facial nerve (CNVII). The lateral semicircular canal runs subhorizontally through the prootic, slightly sloping anteriorly relative to the horizontal plane of the skull. It branches off from the sacculus and the anterior canal within the larger posterodorsal process of the prootic and exits the bone at its posteromedial surface, where it joins the posterior semicircular canal.

The anteroventral process of the prootic in *M. carinatus* is foot-shaped with a linear ventral margin in lateral view. It only contacts the basisphenoid ventrally and posteriorly borders the foramen ovale. In *P. erlenbergiensis*, the corresponding anteroventral process of the prootic (misidentified as being basisphenoid as mentioned in the above description of the basisphenoid) is more bulb-like and contacts the basisphenoid both ventrally and posteriorly. This contact is semicircular in lateral view. *E. minor* has an anteroventral process that is more similar to that of *P. erlenbergiensis* than *M. carinatus*. In *M. carinatus*, the anteroventral process has a flat lateral surface.

The lateral surface of the posterodorsal process is convex in *M. carinatus* although a small shallow cavity is present ventral to the supraoccipital–prootic contact. In dorsal view, the element is oriented anteromedially. In anterior view, the posterodorsal process flares laterally such that the lateral extent is beyond that of the anteroventral process.

The rugose dorsal surface of the prootic contacts the supraoccipital and the exoccipital. The dorsalmost half of the posterodorsal surface of the prootic forms a complex suture with the supraoccipital. This contact excludes the prootic from contacting the parietal, Sues et al. (2004). In lateral view, this suture is inclined anterodorsally at 30° relative to the horizontal axis of the skull. In dorsal view the contact surface is roughly parallelogram shaped, with the anteriormost corner of the parallelogram located along the skull midline, and extending to the level of the anterior end of the sella turcica and posteriormost corner along the lateral side of the skull extending to the level of the base of the paroccipital processes. CT scans reveal that the contacts between the supraoccipital, the prootic and the exoccipital are not coossified close to the skull midline, but that they become progressively more fused as they approach the lateral side of the braincase where they are virtually indistinguishable. The area of these contacts laterally is replaced by trabecular bone. The ventral half of the posterodorsal surface of the prootic forms a complex suture with the anteroventral surface of the base of the paroccipital processes of the exoccipitals. This contact is angled at 15° relative to the vertical axis.

#### Opisthotic–exoccipital

The exoccipitals are complete, well-preserved, and lying in life position. The exoccipital is fused to the opisthotic, as is common in most adult dinosaurs (Figs. 48 and 49). They form the lateral margins of the foramen magnum and the dorsolateral corner of the occipital condyle. The axis of the paroccipital processes is horizontal in lateral view and directed slightly ventrally. CT scans show that the internal structure is composed primarily of trabecular bone. The exoccipital contacts the basioccipital posteromedially, the supraoccipital both dorsally and anterodorsally, the prootic anteriorly and the squamosal laterally. The exoccipital–opisthotic complex is comprised of the paroccipital processes, the contributions to the occipital condyle, the metotic strut, and the lateral margins of the foramen magnum. Internally, the exoccipital contains the passage for the posterior semicircular canal of the inner ear.

The paroccipital process is rectangular, wing-like, and flares slightly dorsoventrally at its distal end. Its long axis is mostly horizontal although it slightly bends dorsally as it extends distally, giving it a slightly convex ventral margin and concave dorsal margin. In *P. erlenbergiensis*, this process has a linear ventral margin and slightly convex dorsal margin. The paroccipital processes diverge dorsolaterally from one another at a 67° angle in *M. carinatus*, contrary to the 45° angle observed by Sues et al. (2004). Each process is also shallowly concave on its medial surface. This medial surface faces slightly dorsally as it extends distally. The paroccipital processes are proportionally anteroposteriorly longer in *P. erlenbergiensis* than in *M. carinatus*.

The ventral margin of the paroccipital process of the exoccipital curves down anteroventrally and tapers into a thin bony rod (the metotic strut). This separates the foramen ovale anteriorly from the metotic fissure posteriorly. The foramen ovale is a neurocranial foramen formed dorsally by the anterior surface of the ventral portion of the exoccipital, posteriorly by the metotic strut, ventrally by the anteriormost section of the dorsal surface of the basioccipital, anteroventrally by the basisphenoid and dorsally by the prootic. The foramen ovale is where the stapes meets the internal ear and is separated from the fenestra pseudorotunda by the crista interfenestralis. The latter is not preserved here and the foramen ovale is therefore not distinguishable from the fenestra pseudorotunda. The metotic fissure is formed anteriorly by the metotic strut, anteroposteriorly and posteriorly by the main body of the exoccipital and ventrally by the dorsal margin of the basioccipital. This fissure is for the passage of cranial nerves CNIX, CNX and CNXI.

The dorsoventral height of the exoccipital body is proportionally much greater in *M. carinatus* than in *P. erlenbergiensis*, and it extends farther ventrally, making the metotic strut dorsoventrally higher and more prominent. The anterior surface of the exoccipital body is rugose in *M. carinatus*. It contacts the prootic. The prootic contact is a complex suture situated on the anterior surface of the exoccipital. It is dorsolaterally oriented in anterior view (see prootic description). The dorsal surface of the exoccipital body is convex and rugose. Its contact with the supraoccipital is a well-sutured butt joint that is subvertically oriented in lateral view, inclined slightly posterodorsally, and posterolaterally oriented in dorsal view. The ventral projection of the exoccipital body forms the dorsolateral corners of the occipital condyle. This condylar projection flares out ventrally and is triangular in lateral view. In *S. aurifontanalis*, this projection slopes ventromedially (as is visible in posterior view). In *M. carinatus*, the posterior margin of the exoccipital and basioccipital contributions to the condyle are continuous and form one semicircular margin in lateral view. In *P. erlenbergiensis*, there is a step between the two with the exoccipital posterior margin being linear and vertical and the basioccipital condyle posterior margin being semicircular in lateral view. The lateral surface of this projection is smooth and bears an elliptical foramen for the passage of the hypoglossal nerve (CNXII). This foramen is oriented posterodorsally. This nerve has two passages from the braincase but exits via a single foramen, visible on the medial and lateral surfaces. In *P. erlenbergiensis*, the lateroventral wall of the exoccipital contains two foramina for the branches of the hypoglossal nerve (CNXII). The medial surface of the exoccipital is smooth and forms the dorsolateral margin of the foramen magnum. This surface is slightly concave. The foramen magnum is more circular and proportionally mediolaterally wider in *S. aurifontanalis* than that of *M. carinatus*. The ventral surface of the ventral projection of the exoccipital body is rugose and is the articular surface for the basioccipital. This contact forms a horizontal, well-sutured butt joint and it extends ventrolaterally to overlap the dorsolateral surface of the basioccipital. The basioccipital and exoccipital also make contact at the ventral surface of the crista interfenestralis of the exoccipital.

Internally, the exoccipital holds the posterior semicircular canal of the inner ear. The latter enters through the anterior surface of the exoccipital (the supraoccipital articular surface) and runs horizontally through the bone. It exits at the anteromedial surface of the exoccipital where it joins the lagena/sacculus.

#### Supraoccipital

The supraoccipital is complete, well-preserved, and lying in life position (Fig. 50). The sagittal axis of the bone is angled at approximately 60° from the axis of the skull. CT scans show that the internal structure is composed primarily of trabecular bone. It contacts the exoccipitals posteroventrolaterally, the prootic ventrolaterally, and the parietal along the dorsal portion of its anterior surface.

Overall, the supraoccipital is sloped posteroventrally and has a transverse section that is U-shaped. This bone broadens anterodorsally, and tapers posteroventrally where it forms the roof of the foramen magnum. The lateral walls of the supraoccipital broaden posteroventrally. The supraoccipital of *S. aurifontanalis* appears to be proportionally mediolaterally wider posteriorly than that of *M. carinatus*.

In dorsal view, the supraoccipital is triangular with the apex of the triangle pointing anteriorly. The posterior corners of the supraoccipitals extend laterally and contribute to the base of the paroccipital processes of the exoccipitals. The exoccipital contact is a well-sutured butt joint. The contact is transversely oriented although it is slightly angled posterodorsally. This contact is mesially divided by a small notch, which corresponds to the foramen magnum dorsal margin. The dorsal surface of the supraoccipital bears a prominent median ridge that begins on the posterior end of the supraoccipital and broadens out as it extends anteriorly. *S. aurifontanalis* does not bear a prominent midline ridge. The lateral surface of the supraoccipital in *M. carinatus* is sloped posteriorly and forms a continuation of the exoccipitals. Triangular tabs project anteriorly from the lateral surface and contact the posterodorsal surface of the laterosphenoid.

The anterior dorsal apex of the supraoccipital contacts the posterior surfaces of squamosal rami of the parietal. It is a well-sutured but thin contact, which is arced dorsally in anterior view, and V-shaped in dorsal view with the apex of the V pointing anteriorly. There is a gap between the anteriormost apex of the supraoccipital and the junction of the parietal squamosal rami which likely is homologous to the postparietal fenestra present in some derived sauropods (Knoll et al., 2012). The supraoccipital of *S. aurifontanalis* has a concave anterior margin, forming a larger more circular post parietal fenestra than in *M. carinatus*.

The ventral surface of the supraoccipital is rugose and corresponds to the articular surface for the prootic. This contact is a well-sutured butt joint, situated on the ventrolateral surfaces of the supraoccipital. The contact is oriented anterodorsally in lateral view and transversely in anterior view. In ventral view, it is triangular with the apex of the triangle pointing anteriorly and forms a continuation with the ventral prootic contact of the exoccipitals.

The medial surface of the supraoccipital forms a vertical narrow groove at its posterior end and broadens out to form an inverted ‘U’-shape as it extends anteriorly. This surface corresponds to the dorsal margin of the foramen magnum. The junction between the posterior and anterior semicircular canals is located in the interior of the supraoccipital. The posterior canal enters the supraoccipital at its exoccipital articular surface. The anterior canal extends from the prootic and enters the supraoccipital at its anteroventral surface (prootic articular surface). The two canals meet within the medial side of the bone, at midheight.

### Otic region

#### Stapes

Contra Sues et al. (2004), the stapes are visible in cross section in BP/1/5241 (Figs. 51 and 52). The left stapes is complete, well-preserved, and lying in life position, whereas the footplate of the right stapes is not preserved. The stapedial shafts are slender rods that are circular in cross section and they expand as they extend anteromedially to form the stapedial footplate. The shafts are relatively straight although there is a slight lateral arc towards the anterior end. The stapes are anteromedially oriented, parallel to the paroccipital process, and their long axes are subhorizontal, with a slight anteroventral slope. The surface of the stapedial footplate that contacts the sacculus is semicircular in shape, with a convex dorsal margin and a linear ventral margin. The foramen piercing the stapes as well as the short diagonal ridge that passes distally onto the shaft, as reported by Galton & Upchurch (2004) are not present on this specimen.

##### Vestibular canals

The lateral semicircular canal is the shortest of the three inner ear canals, whereas the anterior semicircular canal is the longest, as noted by Galton & Upchurch (2004) (Figs. 51 and 52). The cross sections of the anterior and lateral semicircular canals are ovoid, whereas that of the posterior canal is more circular. The posterior and anterior canals join dorsally, with the anterior canal being more dorsally elevated than the posterior canal, as is seen in most archosaurs (Knoll et al., 2012). As described above, the semicircular canals pass through the prootic, exoccipital and supraoccipital. The bony margins of the lagena and sacculus are not preserved in this specimen.

### Dentition

BP/1/5241 bears 21 alveoli on either side of the skull (four alveoli in the premaxilla and 17 in the maxilla) (Fig. 53). The right 12th tooth (from the anterior end) is missing as well as the distal left seven teeth (11–17). The teeth are imbricated with the distal side of a more anterior tooth labially overlapping the mesial side of the succeeding tooth. The teeth bear serrations on both the mesial and distal carinae. These serrations are restricted to the upper half of the crown and are coarse and angled dorsally at an angle of 45° to the margin of the tooth. In general, the teeth decrease in apicobasal height from the anterior end of the tooth row to the distal end. The fourth premaxillary tooth is apicobasally higher than all the other teeth. This can be seen on the left side of the skull. The roots of the teeth represent approximately 50% of the entire apicobasal height of the teeth.

Computed tomography scans reveal the presence of replacement premaxillary teeth for the second and fourth right premaxillary teeth as well as the second, third and fourth left premaxillary teeth. In the maxilla, replacement teeth are present on the 1st, 3rd and 15th right tooth positions and on the 2nd, 3rd, 4th, 5th, 7th and 11th left tooth positions. The replacement teeth are only about 30–50% of the apicobasal length of the descended teeth (when looking at both the crown and root) and are positioned lingually to the descended teeth. The resolution of the scans is insufficient to determine if there are any serrations on the replacement teeth, but the crown morphology strongly resembles that of the apical ends of the descended teeth.

### Phylogenetic analysis

Twenty-two character scores for *M. carinatus* were changed from the original data matrix (Yates et al., 2010). These changes were due either to: missing scores; ambiguous character definitions; or to uncertainty of source specimen referred to for scoring in the original matrix. Three cranial characters required extensive revision: Character 30 in Yates (2010) matrix (Antorbital fossa: Posteroventral extent of medial wall of antorbital fossa either reaching the anterior tip of the jugal or terminating anterior to the anterior tip of the jugal) and 58 in Yates (2010) matrix (Frontal: Frontal contribution to the supratemporal fenestra). Many of the taxa we examined personally (including for *L. huenei*, *P. erlenbergiensis* and *S. aurifontanalis*) have scoring errors for these characters, which we attribute to confusion between the terms ‘fossa’ and ‘fenestra.’ We chose to amend the character 30 coding to accurately use fossa and added a character (char. 32 in our revised character matrix) to describe the posteroventral extent of antorbital fenestra. We chose to amend codings for character 58 to accurately use ‘fenestra,’ forcing us to change scores for many taxa for these characters. We also added a character (char. 63 in our revised character matrix) that describes frontal contribution to the supratemporal fenestra. Therefore, *M. carinatus’* scores are changed from 1 to 0 for character 30 (the antorbital fossa does reach the anterior tip of the jugal as it is present on the anteroventral corner of the lacrimal, however the antorbital fenestra does not) and from 0 to 1 for character 58 (the frontal contributes to the supratemporal fossa but not fenestra). Finally, closer inspection of the character 81 in the original matrix (‘shape of floor of the braincase’) revealed that multiple anatomical features with potentially independent homology statements were present. To assess variation in the braincase floor morphology, we divided this character into several separate characters to ensure that all the potential morphological combinations possible for the floor of the braincase were assessed individually (chars. 97–100 and 104 in our revised character matrix).

**Figure 54:**
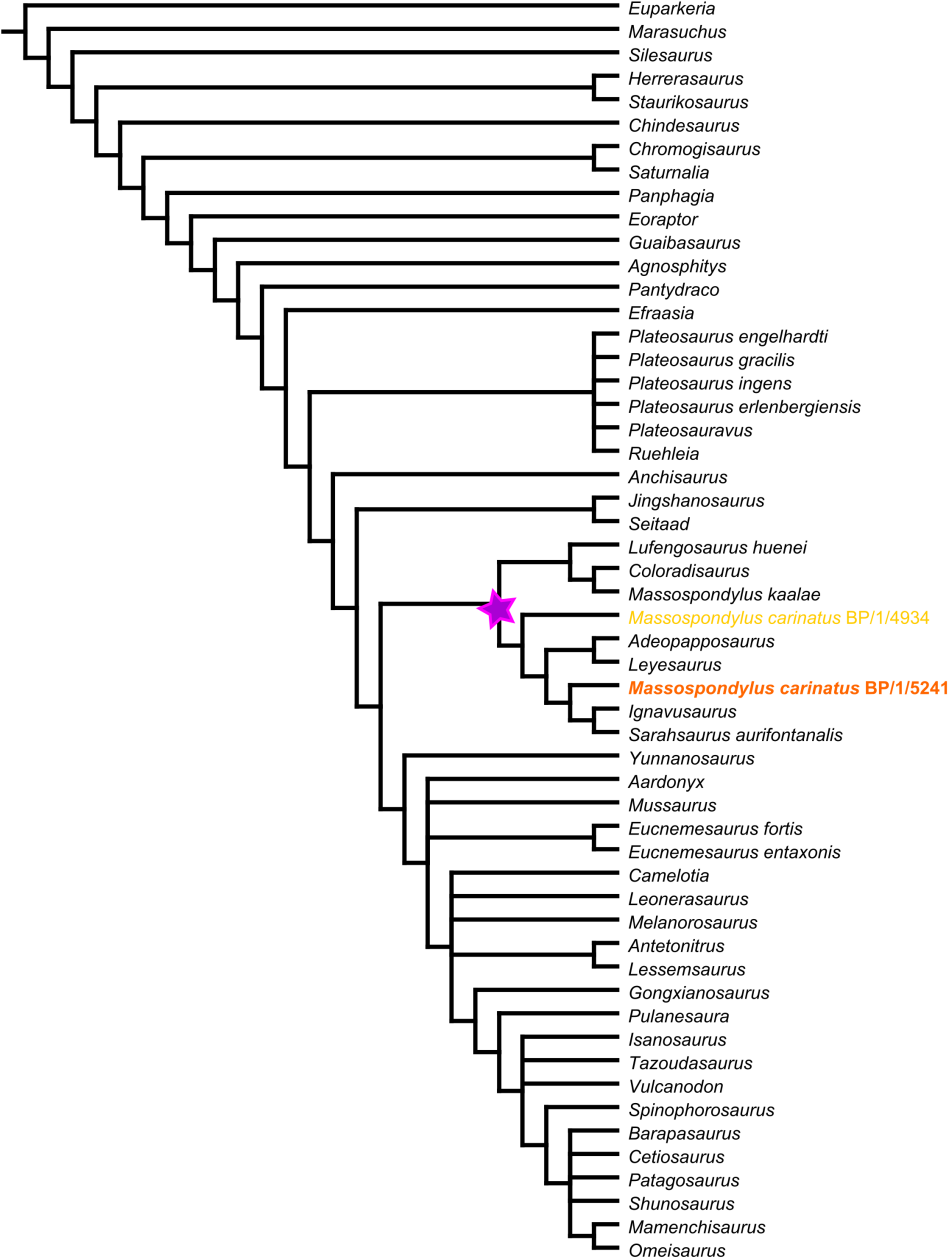
Strict consensus tree of 85 most parsimonious trees (MPTs) of length 1,264, a retention index of 0.636 and a consistency index of 0.346. The purple star indicates Massospondylidae clade.

The ‘New Technology’ search yielded 56 trees of length 1,264. The ‘Traditional Search’ then produced 85 most parsimonious trees (MPTs) of length 1,264, a retention index of 0.636 and a consistency index of 0.346 (Supplemental Information 3). The strict consensus tree of this phylogenetic analysis is presented (Fig. 54). All trees support a monophyletic Massospondylidae comprising *S. aurifontanalis*, *Ignavusaurus rachelis*, *Leyesaurus marayensis*, *Adeopapposaurus mognai*, *M. carinatus* (BP/1/5241 and BP/1/4934), *Coloradisaurus brevis*, *Massospondylus kaalae* and *L. huenei*. In total, this clade is supported by four cranial synapomorphies and three postcranial synapomorphies. Cranial features supporting this clade are: a depression behind the naris along the dorsal margin of the snout (char. 22, state 1); a pterygoid ramus that occupies less than 70% of the dorsoventral height of the quadrate (char. 76, state 0; except in *Leyesaurus*); the anteroposterior axis of the dorsal surface of the supraoccipital strongly sloping so that the dorsal tip lies level with the basipterygoid processes in lateral view (char. 83, state 1; except in *Adeopapposaurus*) and a ventral margin of the dentary that is ventrally curved in lateral view (char. 119, state 1; except in *Ignavusaurus* and *Sarahsaurus*).

A unique combination of character states sets BP/1/5241 apart from other terminals in our analysis. These include two cranial characters and six postcranial characters. These cranial characters are: basipterygoid processes that are separated by an angle smaller than 60° (char. 90, state 0), and a jugal process of the ectopterygoid that is strongly curved (char. 105, state 1).

In all the MPTs and the strict consensus tree, the two *M. carinatus* specimens coded in this analysis are polyphyletically positioned within a clade containing *S. aurifontanalis*, *I. rachelis*, *L. marayensis*, and *A. mognai*. In this topology, BP/1/5241 is sister taxon to the most derived monophyletic clade containing *I. rachelis* and *S. aurifontanalis*, and BP/1/4934 is the earliest branching member of the group.

## Discussion

### New information on *M. carinatus*

The use of CT scans allows us to reveal new information regarding the anatomy and morphology of the *M. carinatus* cranium, especially with regards to the braincase and palate. Of special note are the presence of an ossified orbitosphenoid. These orbitosphenoids have a hollow internal structure (Fig. 44). New information on the carotid foramina revealed that the two carotid arteries enter the basisphenoid laterally then enter the sella turcica via a common foramen (separated by a low septum). The digital reconstructions also allowed for a clear visualization of the laterosphenoids which are poorly known in basal sauropodomorphs. These differ greatly to *Plateosaurus* laterosphenoids. A clear visualization and description of the morphologies of the vestibular system including the inner and middle ears were possible. A previously unknown structure of the palate was identified. This bulbous process that articulates with the jugal could be a possible autapomorphy for *M.* carinatus (presently, this is difficult to observe in other basal sauropodomorphs). Finally, the replacement teeth of *M. carinatus* could be clearly viewed and no specific pattern in the distribution of the replacement teeth was found.

Digital reconstructions of the scans enable the shape of the floor of the braincase to be explored in greater detail. This is an important systematic character in basal sauropodomorphs and it differentiates (among other things) *Plateosaurus* and *M. kaalae* from *M. carinatus*. Three states are currently hypothesized for a single character (char. 81 in the original matrix) describing braincase floor shape: straight, with ventral margin of the occipital condyle, ventralmost extent of the basal tubera, proximal base of the basipterygoid process and anterior tip of the cultriform all aligned at same level (state 0 as in *Thecodontosaurus*); bent, with proximal base of the basipterygoid process and anterior tip of cultriform process below the level of the ventral margin of the occipital condyle and ventralmost extent of basal tubera (state 1 as in *Plateosaurus*) and bent with the ventral most extent of basal tubera below the level of the ventral margin of the basioccipital condyle and the anterior tip of the cultriform process at a level dorsal to it (state 2 as in *Anchisaurus*) (Yates, 2010). In previous analyses (Yates et al., 2010) *M. carinatus* was scored as state 0. However, our scans reveal that in the nearly undistorted BP/1/5241 specimen, the anterior tip of the cultriform process and ventral margin of the occipital condyle are dorsal to the ventral margin of the basal tubera and proximal base of the pterygoid processes.

This morphology is difficult to homologize with any of the states. Furthermore, this morphology varies ontogenetically. When compared to the basisphenoid of the juvenile specimen BP/1/5231 [illustrated by Gow (1990)], clear differences are apparent with regards to the morphology of BP/1/5241. The BP/1/5231 basisphenoid has a much wider angle between its basal tubera and basipterygoid processes and the two structures reach the same ventral level. It also has an anteriorly oriented cultriform process. In BP/1/5241, the basal tubera are positioned more dorsally and the basipterygoid processes are more ventrally oriented and extend further ventrally than the basal tubera. The cultriform process extends anterodorsally. Thus, we consider that ontogeny likely affects the architecture of the floor of the braincase. This character should therefore be treated with caution as currently circumscribed, especially when ontogenetic stages of the exemplars are unknown, and future efforts need to refine the homology statements contained within it.

Our investigation also allows us to correct several minor misinterpretations found in previous literature. Sues et al. (2004) described the prootic as contacting the parietal dorsally and Gow (1990) found the opposing prootics to be connected medially. Our results show otherwise: the prootics in *M. carinatus* are medially separated, and they do not contact the parietal dorsally but rather contact the supraoccipital. In lateral view on the specimen, the supraoccipital is only marginally visible, and it is possible that the prootic/supraoccipital contact was previously misidentified because of this. Our interpretation of the prootic/supraoccipital contact is congruent with the findings of Gow (1990), and this feature is shared with *P. erlenbergiensis* within non-sauropodan Sauropodomorpha. The entrance of the internal carotids is single, as found by Gow (1990), but a low septum separates the two carotid entrances at the posterior end of the sella turcica. It is possible that this septum was more well-developed in life, either by a thin sheet of bone that is not preserved or by cartilaginous tissue that did not preserve.

### Comparisons to other *M.* species

Taxonomic work on *Massospondylus* by Barrett (2004, 2009) identified a second valid species within the genus, *M. kaalae*. Several braincase features were used to support this species-level distinction, including: the *M. kaalae* specimen has a ‘stepped’ braincase with the cultriform process and basipterygoid processes lying ventral to the basioccipital condyle; the basipterygoid processes are separated by a 60° angle (Barrett, 2004), later redefined as a 40° angle (Barrett, 2009), and are distally unexpanded relative to the shaft; and finally, in ventral view, the depression between the basipterygoid processes (the basisphenoid recess) is deep and well-defined. *M. carinatus* was described as having a 100° angle between its basipterygoid processes, mediolateral expansion of the distal end of its basipterygoid processes, and a shallow basisphenoid recess (Barrett, 2004, 2009). Comparisons were made in the paper to *M. carinatus*, but were not accompanied by specific specimen numbers.

Substantial variation exists across different ontogenetic stages and different specimens of *carinatus*, making it important to reassess the validity of *M. kaalae* (Barrett, 2004, 2009). Many of the distinguishing characters of *M. carinatus* put forward in Barrett’s (2004, 2009) comparisons differ from the morphology observed in BP/1/5241. *M. carinatus* has non-expanded basipterygoid processes separated by a 35° angle, and the basisphenoid recess is deep and well-defined. Although there is a difference in the alignment of the braincase (chars. 97–100 and 104 in the revised matrix) between *M. kaalae* and *M. carinatus*, the phylogenetic significance of this difference is difficult to determine due to the ontogenetic variability of these characters. Ontogenetically variable characters can be misleading when different-aged exemplars are used in an analysis. It is therefore important to assess the ontogenetic age of the specimens before these differential features can be accepted. This could be improved by running a phylogenetic analysis using specimens for which ages have been determined (e.g. by using histology) as individual taxonomic units.

### Phylogenetic systematics

We modified the original matrix by adding 27 new cranial characters and deleting five pre-existing characters to reflect new information gained from this research and to determine relationships in non-sauropodan Sauropodomorpha. Eight of the new characters are a result of reductive coding (sensu Strong & Lipscomb, 1999) of multistate characters that included the state ‘absent.’ It is important to note that most of the new cranial characters added to the matrix were specifically braincase characters.

The phylogenetic analysis yielded a well-supported Massospondylidae clade across all trees. This clade includes *S. aurifontanalis*, an outcome which has not often been hypothesized (Pol & Powell, 2007; Rowe, Sues & Reisz, 2010; Upchurch, Barrett & Galton, 2007). This is however interesting since *S. aurifontanalis* was previously referred to *Massospondylus* sp. from the North American Kayenta Formation and then reassigned to *Sarahsaurus* in 2010 (Attridge, Crompton & Jenkins, 1985; Rowe, Sues & Reisz, 2010). It also means that Massospondylidae was a biostratigraphically successful clade as it has members present on three major contemporary continents (*S. aurifontanalis* in North America; *L. marayensis*, *A. mognai* and *C. brevis* in South America; *M. carinatus, M. kaalae*, and *I. rachelis* in southern Africa, and *L. huenei* in Asia).

Our results yielded two monophyletic groups within Massospondylidae. The first comprises *S. aurifontanalis*, *I. rachelis*, *L. marayensis*, *A. mognai* and *M. carinatus*; and the second comprises *C. brevis*, *M. kaalae* and *L. huenei*. There are several taxonomic issues worth mentioning. Firstly, *M. carinatus* and *M. kaalae* do not form a monophyletic clade within Massospondylidae. This could be due to the incompleteness of the *M. kaalae* specimen (i.e. incomplete skull and no postcranial material) which could be affecting the results of the analysis. Secondly, the inclusion of two exemplars for *M. carinatus* (BP/1/5241 and 4934) did not return a consistent sister-taxon relationship for these specimens, and the presumed younger specimen (BP/1/5241) is hypothesized as being more derived than the larger specimen (BP/1/4934). The two specimens differ in age and preservation. BP/1/5241 is 14% smaller in size than BP/1/4934. It is not known what the exact growth stage difference is between the two specimens or how this affects character scores for phylogenetic analyses. Furthermore, the postcranial material for BP/1/4934 as well as the presence of the lower jaw make this specimen more complete than BP/1/5241. This could affect the results of the phylogenetic analysis. More stringent tests need to be done that would include a larger sample of individual specimens of different growth stages in order to understand how ontogeny affects character scoring and phylogenetic hypotheses. These issues present an ideal opportunity for further taxonomic revision within the group which will be presented elsewhere.

The revision of braincase characters in basal sauropodomorphs is important to better represent possible homology statements. However, due to the lack of descriptive detail in the basal sauropodomorph braincase literature, the additional new braincase characters could not be scored for many of the other taxa. It is also important to note the uncertainty regarding the ontogenetic stages of the specimens used as well as how this affects character scores. With future work on sauropodomorph braincases, such as the recent work by Bronzati & Rauhut (2017), several new autapomorphies for *M. carinatus* should become apparent.

## Conclusion

The use of CT scanning and 3D visualization graphics allows for a better understanding of the internal and external morphological structures of the braincase as well as information about the soft tissues such as the vestibular canals. *M. carinatus* can be tentatively diagnosed cranially by basipterygoid processes that are separated by an angle smaller than 60° and a jugal process of the ectopterygoid that is strongly curved. A revision of cranial characters provides a basis for more comparative work on the braincase of sauropodomorphs in general. Results also show a well-supported Massospondylidae clade. Further phylogenetic analyses using individual specimens of known ontogenetic stages as operational taxonomic units would provide better resolution for the Massospondylidae clade as well as a better understanding of which sets of character states set *M. carinatus* aside from other taxa.

## Supplemental Information

10.7717/peerj.4224/supp-1 Supplemental Information 1 Revised and illustrated cranial characters used in this study. Postcranial characters were not revised and are originally from Yates (2007). They are therefore not listed here (see Supplemental file, S3.nexus).

10.7717/peerj.4224/supp-2 Supplemental Information 2 3D model of BP/1/5241.

10.7717/peerj.4224/supp-3 Supplemental Information 3 Character matrix used in analysis. Cranial characters were revised (see Supplemental file, S1.pdf). Postcranial characters were not revised and are originally from Yates (2007).

## Additional Information and Declarations

### Data Availability

The following information was supplied regarding data availability: The raw data have been provided as Supplemental Dataset Files and at MorphoSource: http://www.morphosource.org/Detail/ProjectDetail/Show/project_id/426.
