# Supplementary material for: A revised cranial description of Massospondylus carinatus Owen (Dinosauria: Sauropodomorpha) based on computed tomographic scans and a review of cranial characters for basal Sauropodomorpha
Source: PeerJ. 2018 Jan 12;6:e4224. doi: 10.7717/peerj.4224 (PMC5768178; doi:10.7717/peerj.4224)
Supplement: Supplemental Information 1 — Postcranial characters were not revised and are originally from Yates (2007). They are therefore not listed here (see Supplemental file, S3.nexus). [file peerj-06-4224-s001.pdf]

The character matrix used as a basis for this study is that of Yates et al (2010) which is modified from the earlier matrix used by Yates (2007). This matrix includes characters acquired and/or modified from 19 other sources (Barrett et al. 2005; Benton et al. 2000; Galton 1990; Galton & Bakker 1985; Galton & Upchurch 2004; Gauthier 1986; Holtz 1994; Langer 2004; Leal et al. 2004; Rauhut 2003; Sereno 1999; Sereno et al. 1996; Sereno et al. 1993; Upchurch 1995; Upchurch 1998; Wilson 2002; Wilson & Sereno 1998; Yates 2003a; Yates 2003b). This original matrix comprises 353 characters, of which 120 regard craniodental homologies.

We modified this matrix by adding 27 new cranial characters and deleting five pre-existing characters to reflect new information gleaned from this research and to determine relationships in non-sauropodan Sauropodomorpha. The characters deleted were either ambiguous or did not make any useful homology statements within basal Sauropodomorpha. Eight of the new characters are a result of reductive coding (sensu Strong & Lipscomb 1999) of multistate characters that included the state "absent". The revised cranial characters are presented below. Where new characters are presented, or where characters have been revised heavily, we present discussions and images to assist in assessing the homology statements.

All pictures not taken by the author are referenced below figures. For specimen numbers and collection information on pictures taken by author, please refer to table 1 in paper. For unrevised postcranial characters used in analyses please refer to S2 Nexus character matrix.

1. Entire skull: Ratio of maximum anteroposterior length of skull to proximodistal length of femur (Gauthier 1986; from Yates 2007 ch. 1)

0 greater than 0.6

1 less than 0.6

2. Premaxilla: ventrolateral margin of alveolar region extends further ventrally than ventromedial margin in anterior/posterior view (Upchurch 1995; from Yates 2007 ch. 2)

0 absent

1 present

3. Entire skull: Dorsoventral height of skull at posterior margin of the external naris (Langer 2004; from Yates 2007 ch. 3)

0 more than 0.6 the height of the skull at the middle of the orbit

1 less than 0.6 the height of the skull at the middle of the orbit

4. Premaxilla: Foramen on the lateral surface of the premaxillary body (Yates 2007 ch. 4)

0 absent

1 present

5. Premaxilla: Morphology of the distal end of the nasal ramus (dorsal ramus) of the premaxilla (Sereno 1999; from Yates 2007 ch. 5)

0 tapered

1 mediolaterally expanded

6. Premaxilla: Lateral surface of the premaxilla (Upchurch 1995; from Yates 2007 ch. 6)

0 convex

1 with an inflection at the base of the nasal ramus (dorsal process)

7. Premaxilla: Size and position of the maxillary ramus (posterolateral process) of the premaxilla (Yates 2007 ch. 7)

0 large and lateral to the anteromedial process of the premaxillary ramus of the maxilla

1 small and medial to the premaxillary ramus of the maxilla

8. Premaxilla: Contact between the maxillary ramus of the premaxilla and the premaxillary ramus of the nasal (New character)

0 absent, separated by maxilla

1 present

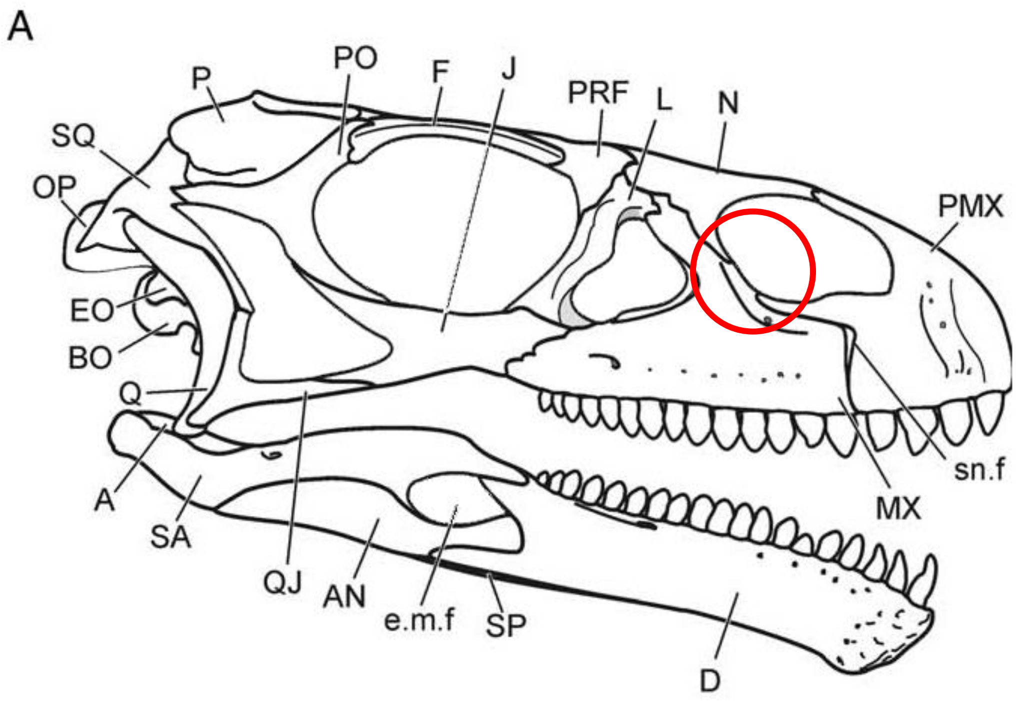

Adeopapposaurus (0) (Image from: Martinez & Alcober 2009)

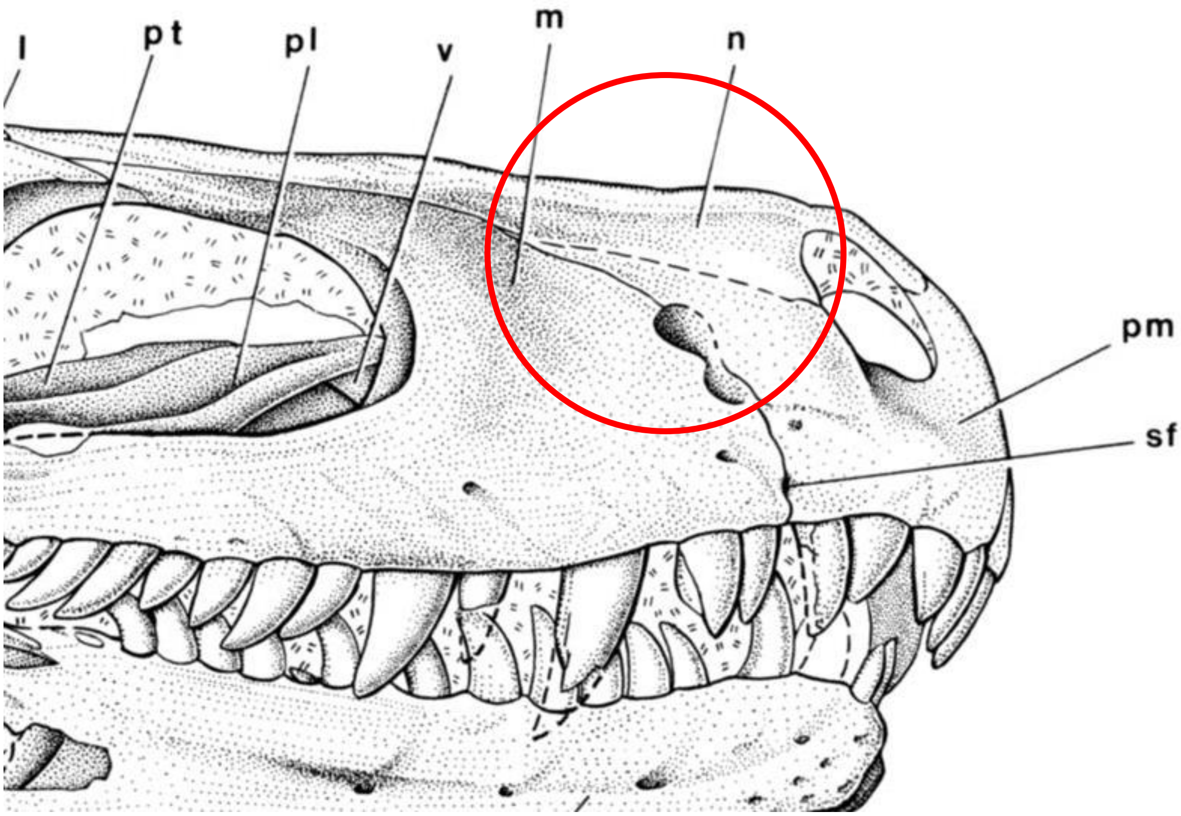

Herrerasaurus (1) (Image from: Sereno & Novas 1993; copyright © Society of Vertebrate Paleontology, www.vertpaleo.org, reprinted by permission of Taylor & Francis Ltd, http://www.tandfonline.com on behalf of Society of Vertebrate Paleontology)

9. Premaxilla: Relationship between the maxillary ramus of the premaxilla and the premaxillary ramus of the nasal (Modified from Gauthier 1986. Ordered, from Yates 2007 ch. 8)

  0 broad sutured

  1 point contact

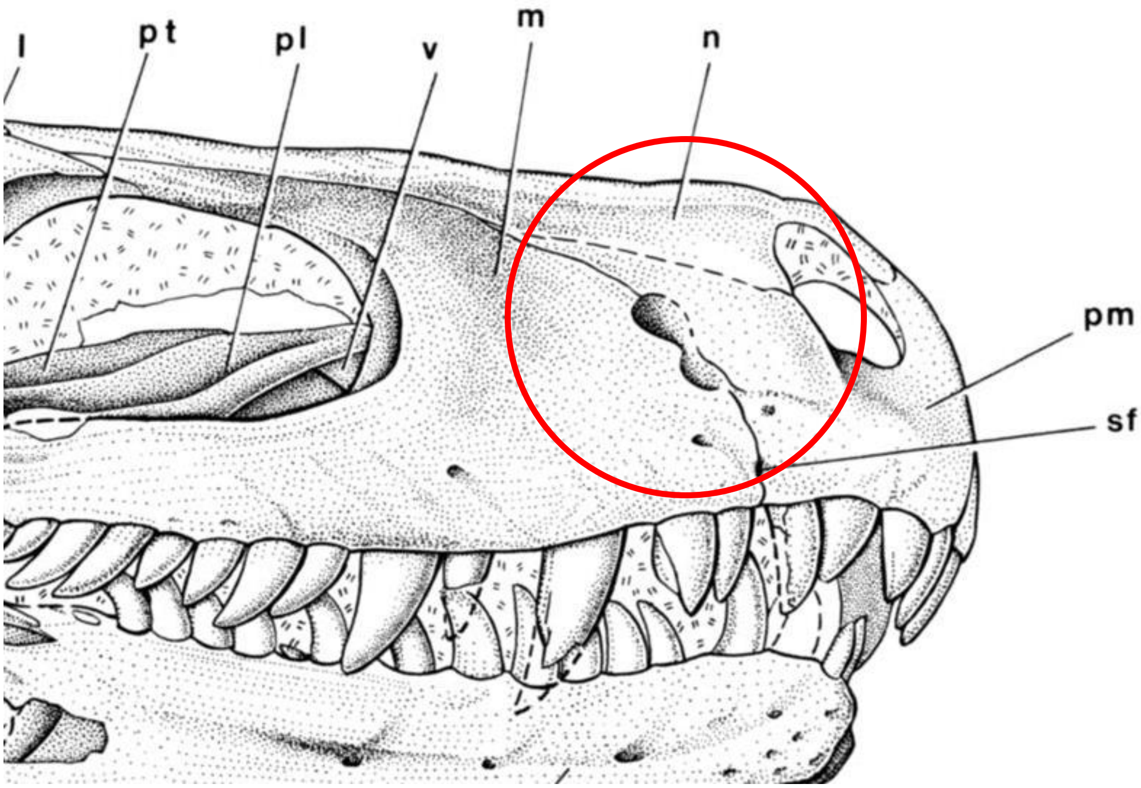

Herrerasaurus (0) (Image from: Sereno & Novas 1993; copyright © Society of Vertebrate Paleontology, www.vertpaleo.org, reprinted by permission of Taylor & Francis Ltd, http://www.tandfonline.com on behalf of Society of Vertebrate Paleontology)

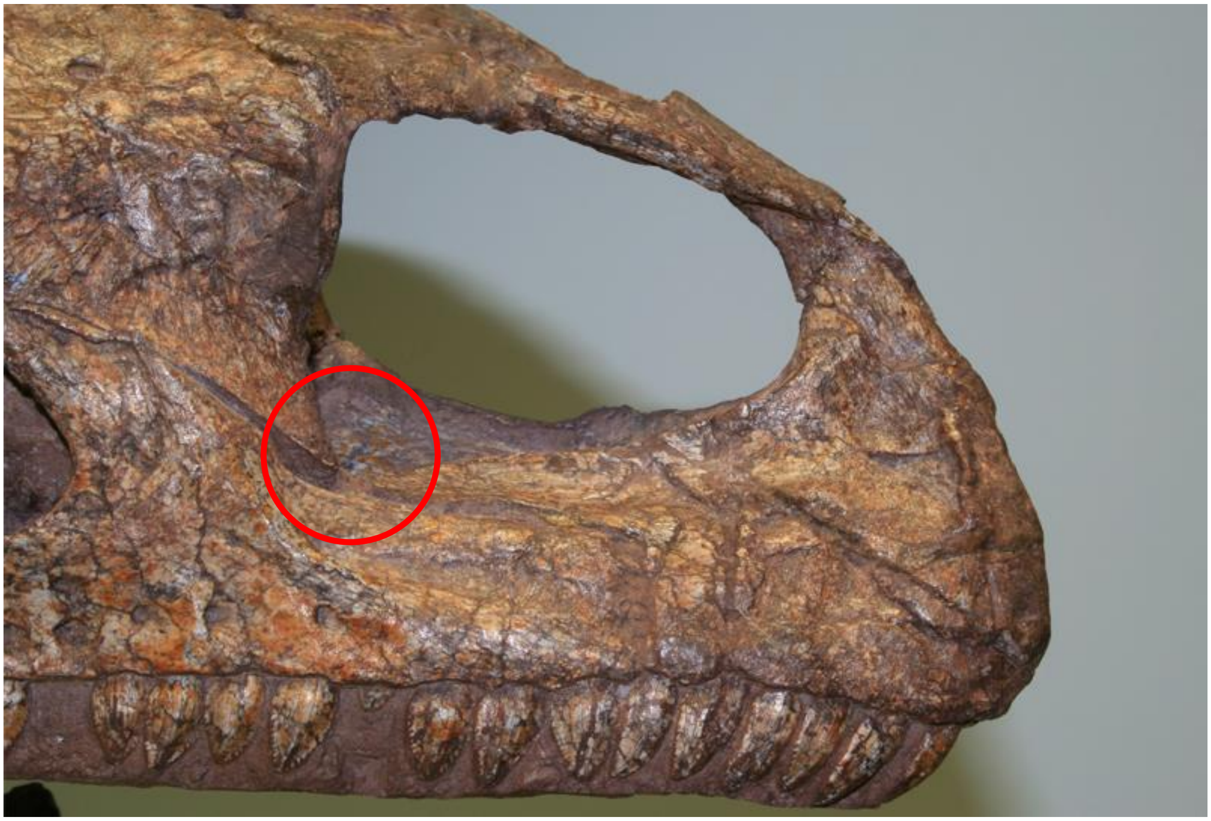

Plateosaurus engelhardti (1) (Image by: Kimberley Chapelle)

10. Premaxilla: Posteromedial process of the premaxilla (Rauhut 2003; from Yates 2007 ch. 9)
    0 absent
    1 present

11. Maxilla: Shape of the anteromedial process of the premaxillary ramus of the maxilla (Yates 2007 ch. 10)
    0 narrow, elongated and projecting anterior to lateral premaxilla-maxilla suture
    1 short, broad and level with lateral premaxilla-maxilla suture

12. External narial fossa: Development of external narial fossa (Yates 2007 ch. 11)
    0 absent to weak
    1 well developed with sharp posterior and anteroventral rims

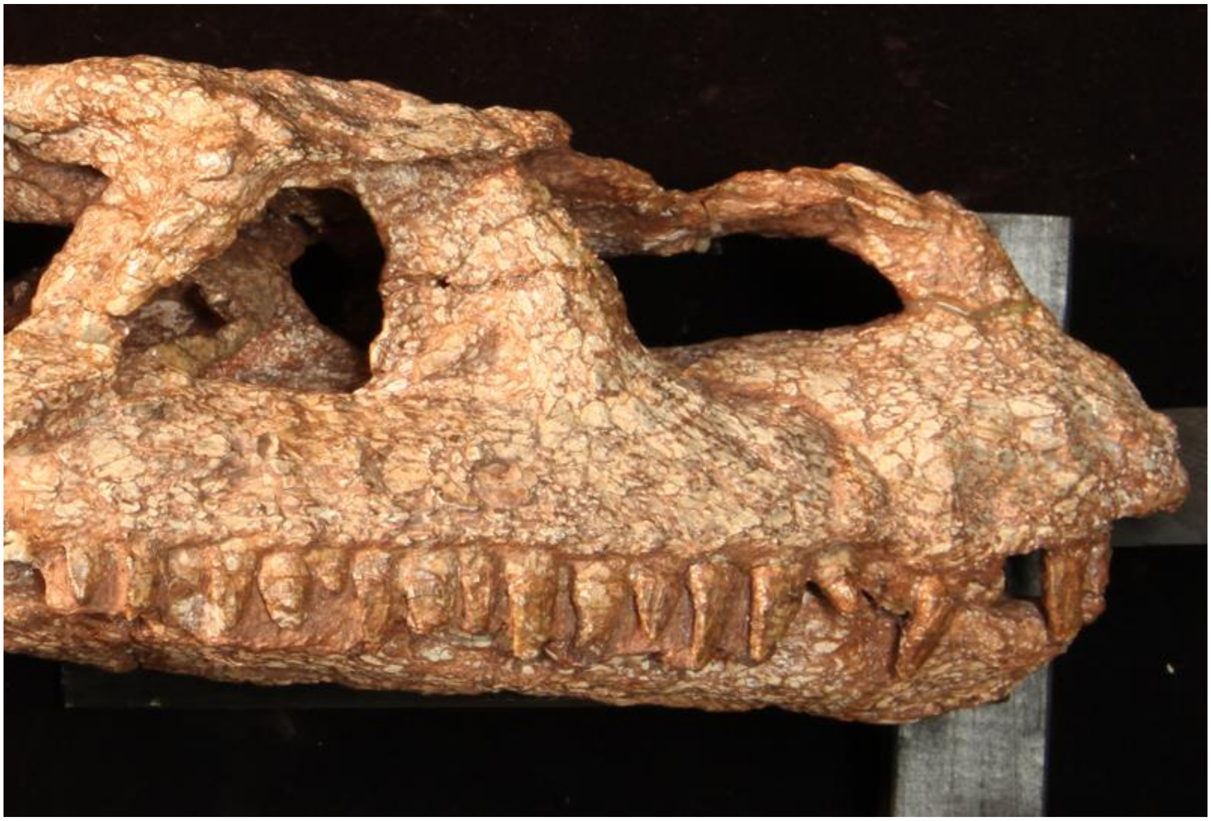

Melanorosaurus (0) (Image by: Kimberley Chapelle)

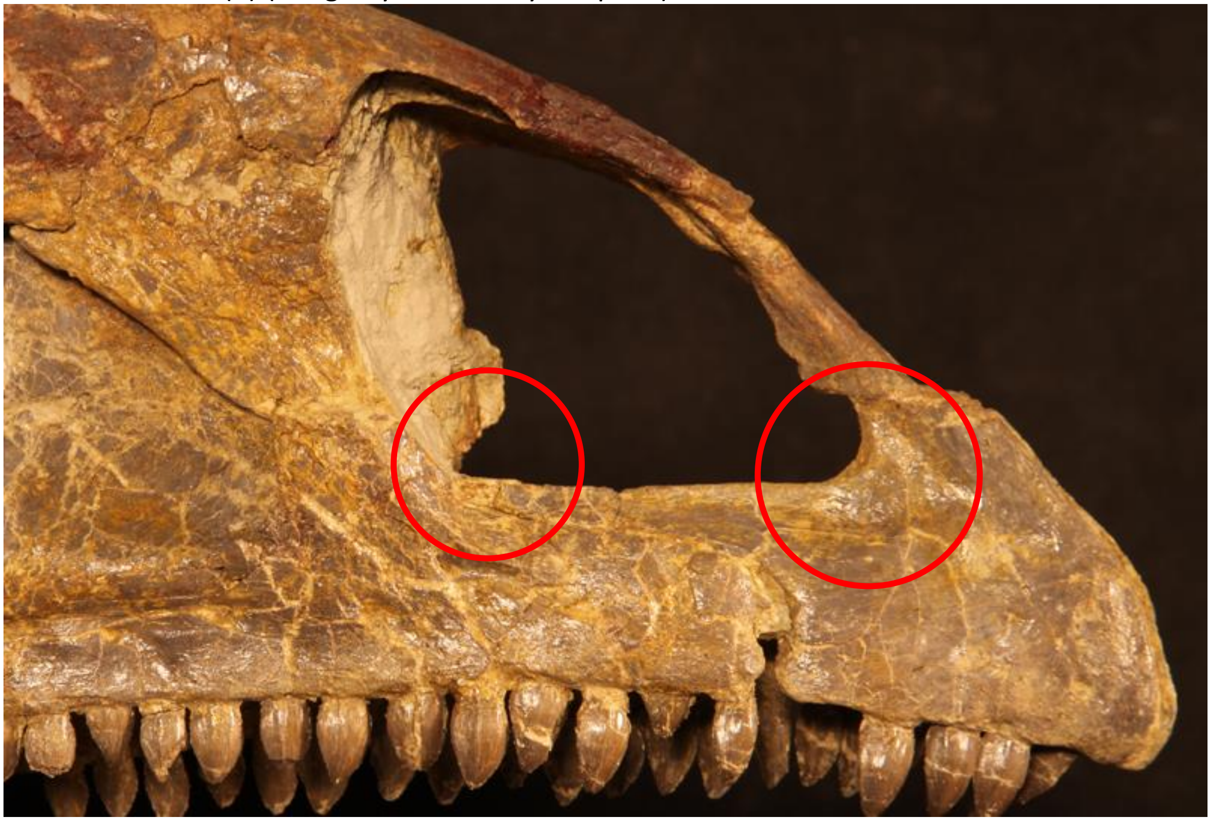

P.

Plateosaurus longiceps (1) (Image by: Kimberley Chapelle)

13. Maxilla: Maxillary contribution to the posterior and posteroventral margins of the narial fossa (Yates 2007 ch. 15)

0 absent

1 present

14. Maxilla: Development of narial fossa on the premaxillary ramus of the maxilla (anterior ramus) (modified from Upchurch 1995; Yates 2007 ch. 12)

0 weak and orientated laterally to dorsolaterally

1 well developed and forming a horizontal shelf

15. Subnarial foramen: presence (New character)

0 absent

1 present

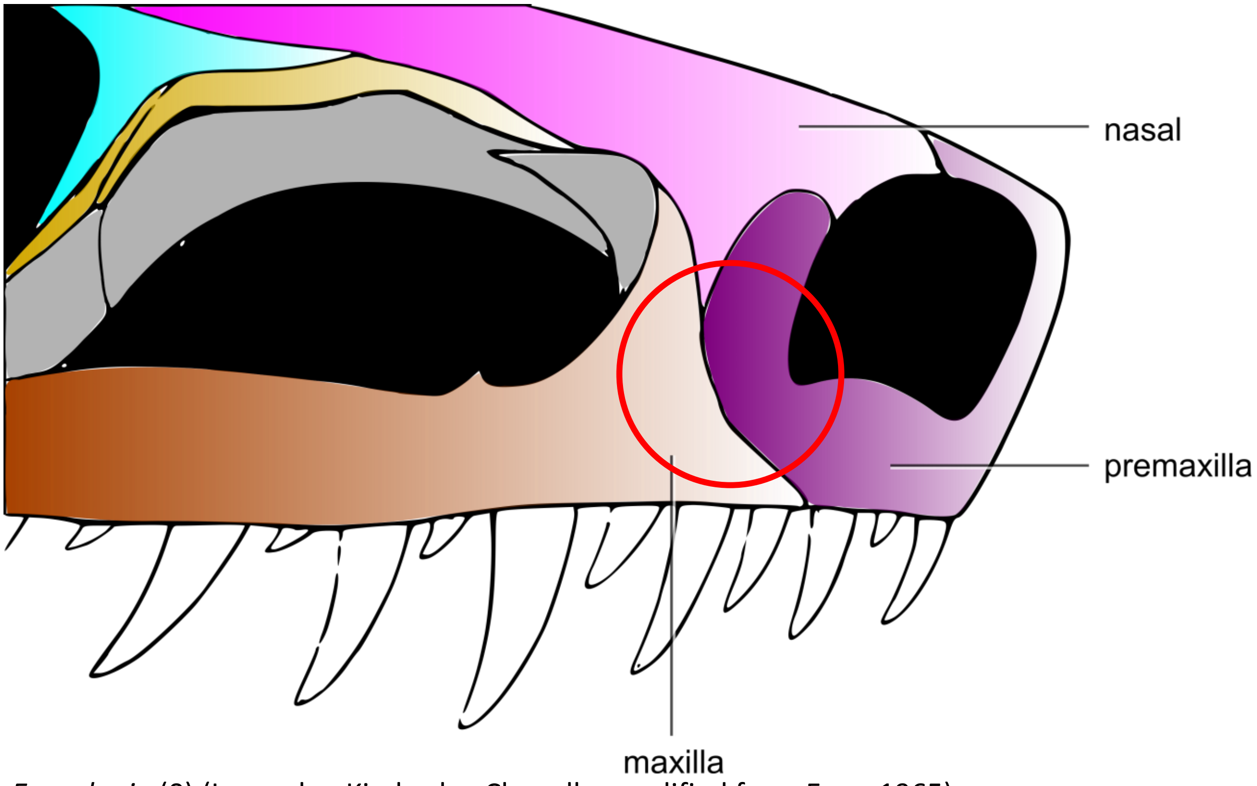

Euparkeria (0) (Image by: Kimberley Chapelle, modified from Ewer 1965)

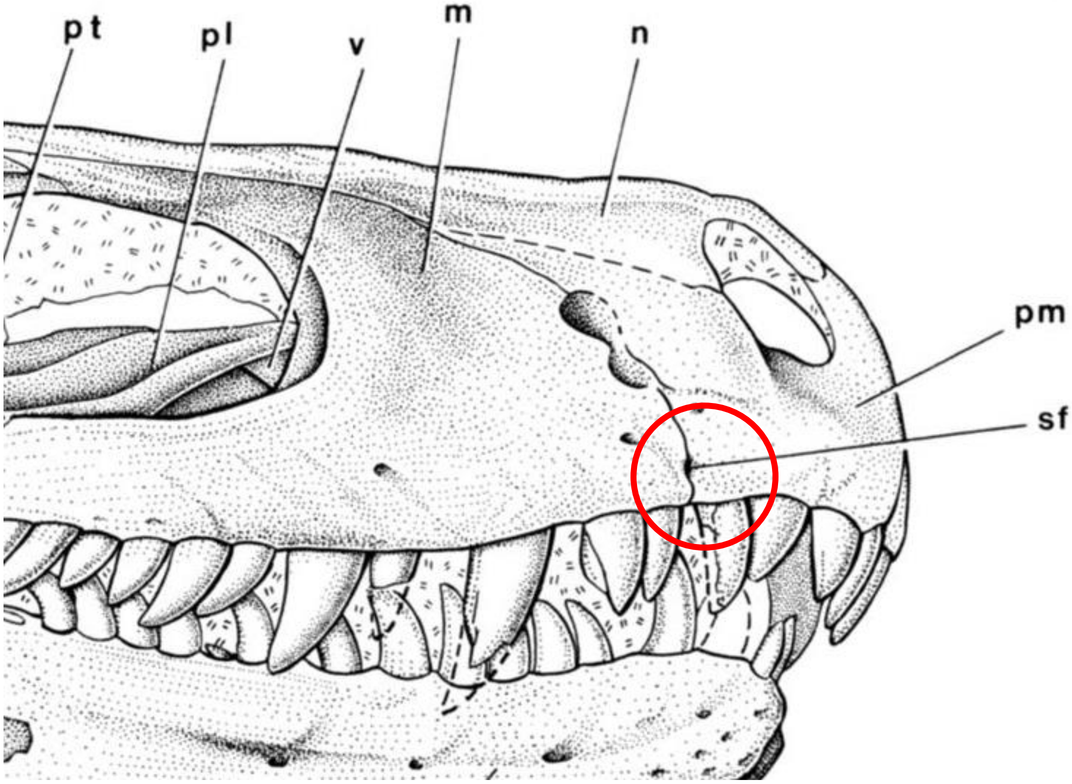

Herrerasaurus (1) (Image from: Sereno & Novas 1993; copyright © Society of Vertebrate Paleontology, www.vertpaleo.org, reprinted by permission of Taylor & Francis Ltd, http://www.tandfonline.com on behalf of Society of Vertebrate Paleontology)

16. Subnarial foramen: Size and position of subnarial foramen (modified from Sereno et al.1993. Ordered, Yates 2007 ch 13)

0 small (no larger than adjacent maxillary neurovascular foramina) and positioned outside of narial fossa

1 large and on the rim, or inside, the narial fossa

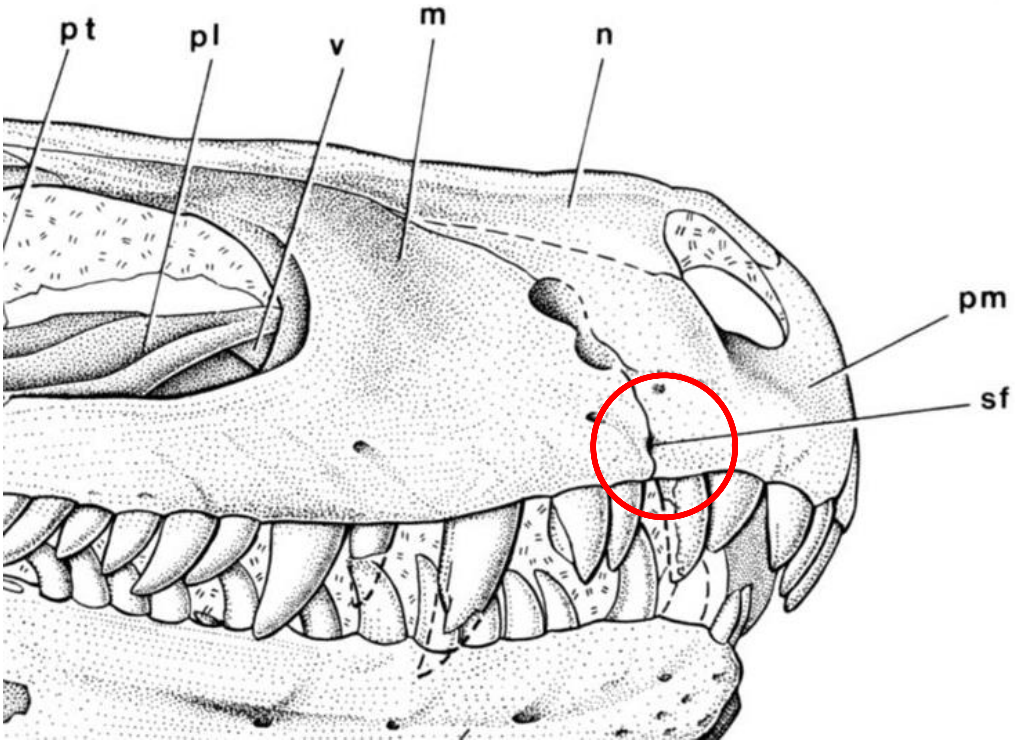

Herrerasaurus (0) (Image from: Sereno & Novas 1993; copyright © Society of Vertebrate Paleontology, www.vertpaleo.org, reprinted by permission of Taylor & Francis Ltd, http://www.tandfonline.com on behalf of Society of Vertebrate Paleontology)

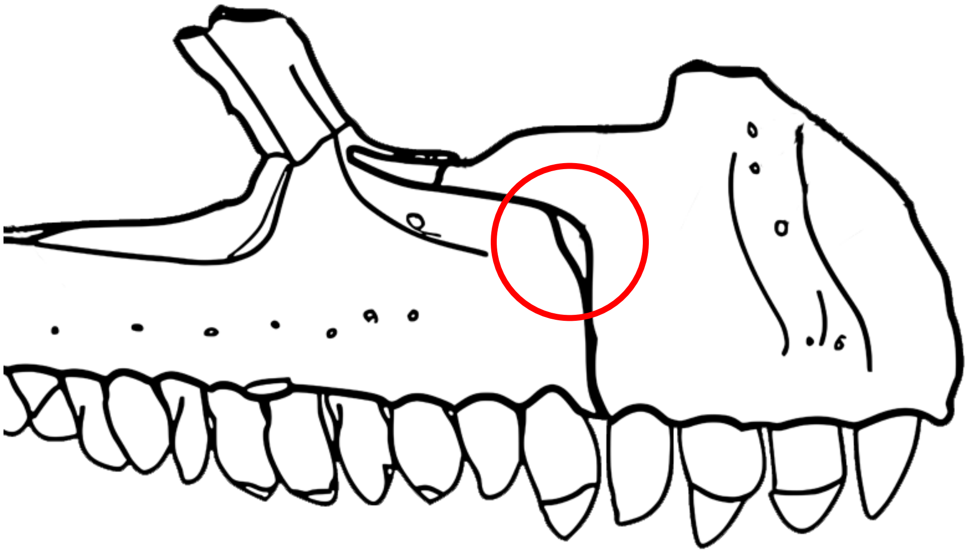

Adeopapposaurus (1) (Image from: Martinez & Alcober 2009)

17. Subnarial foramen: Shape of subnarial foramen (Yates 2007 ch. 14)
    0 rounded
    1 slot-shaped

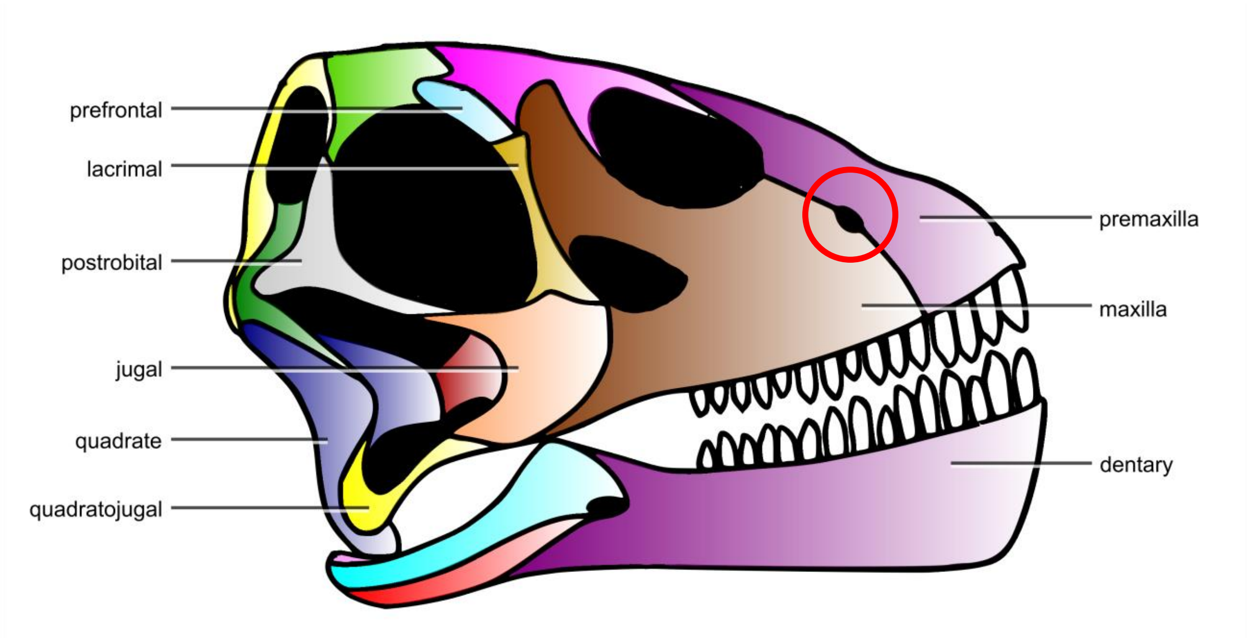

Omeisaurus (0) (Image by: Kimberley Chapelle, modified from He et al. 1988)

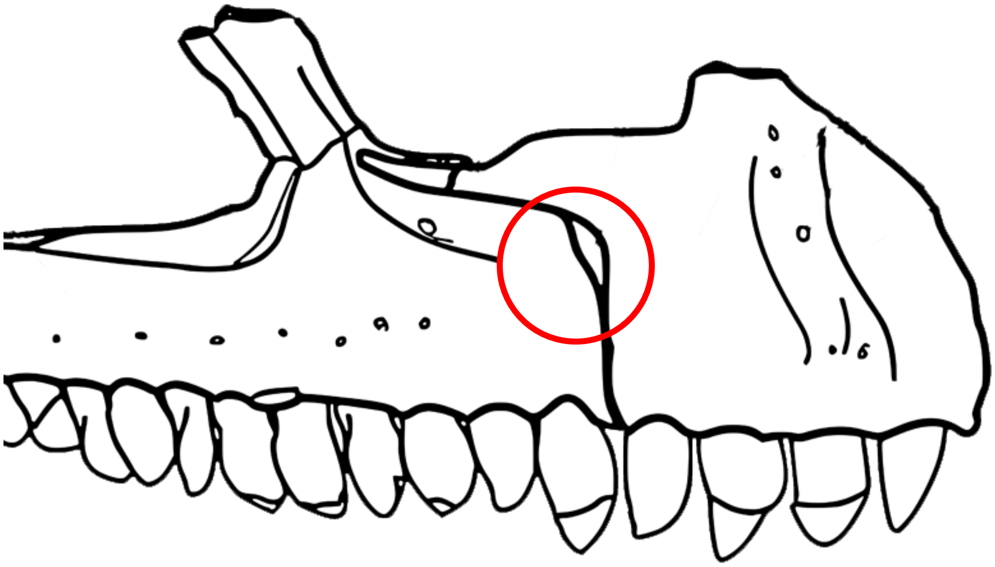

Adeopapposaurus (1) (Image from: Martinez & Alcober 2009)

18. External naris: ratio of anteroposterior diameter of external naris to anteroposterior diameter of orbit, both at midheight (Wilson and Sereno 1998; Yates 2007 ch. 16)

    0 less than 0.5 of the orbital diameter
    1 greater than 0.5 of the orbital diameter

19. External naris: Shape of the external naris (in adults) (Galton and Upchurch 2004; Yates 2007 ch. 17)

    0 rounded
    1 subtriangular with an acute posteroventral corner

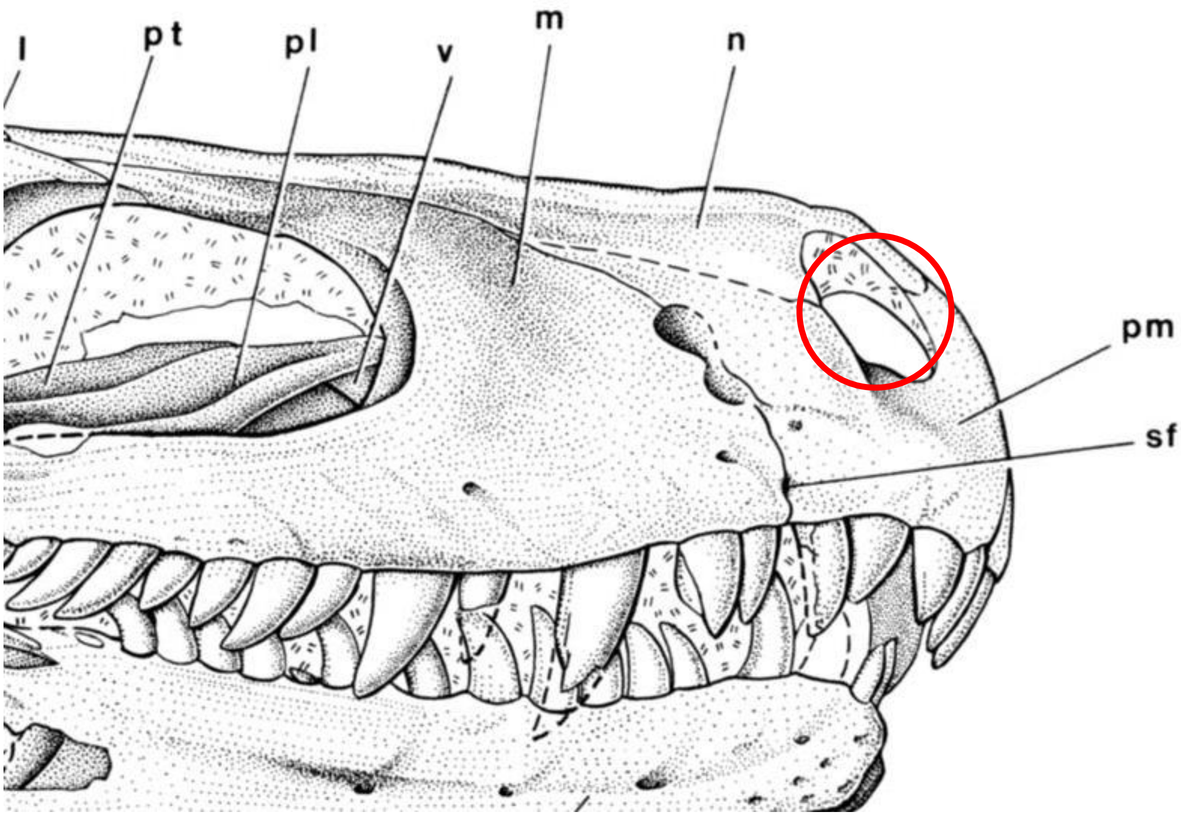

Herrerasaurus (0) (Image from: Sereno & Novas 1993; copyright © Society of Vertebrate Paleontology, www.vertpaleo.org, reprinted by permission of Taylor & Francis Ltd, http://www.tandfonline.com on behalf of Society of Vertebrate Paleontology)

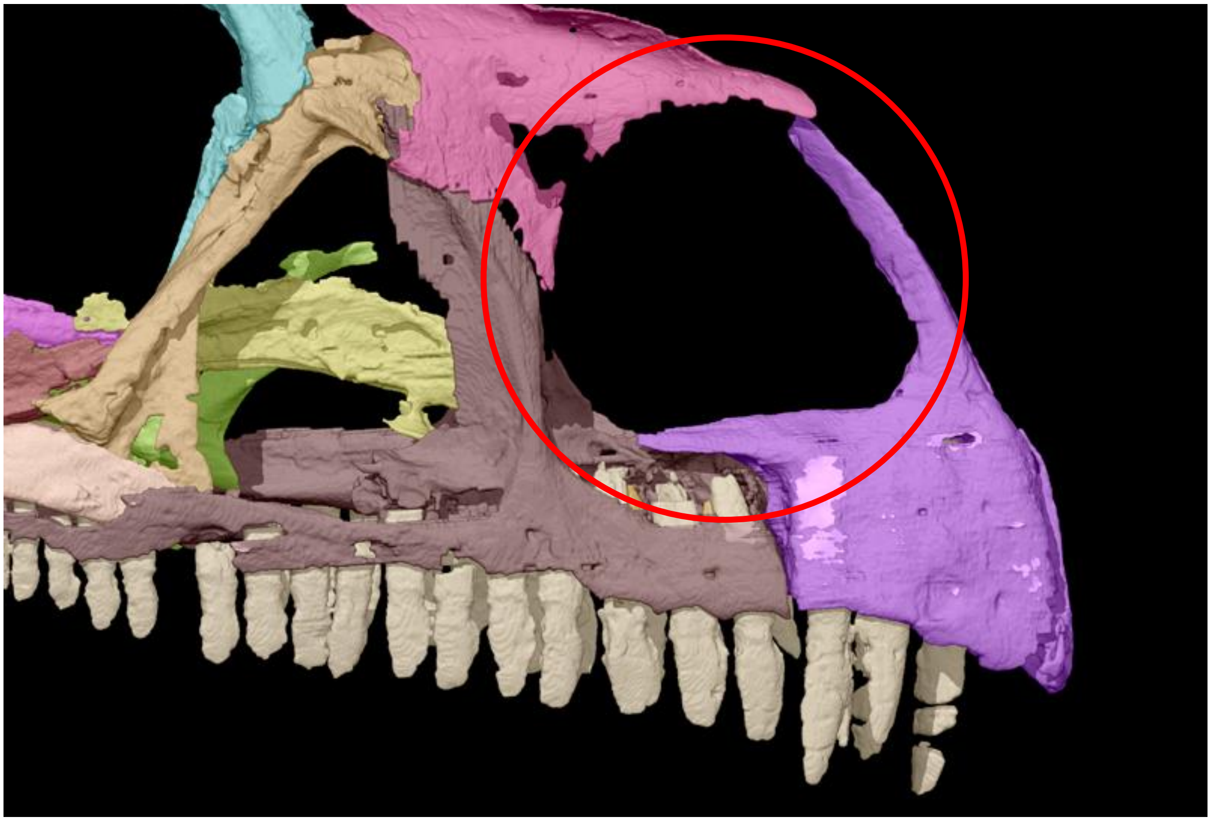

Massospondylus (1) (Image by: Kimberley Chapelle)

20. External naris: Level of the anteroventral corner of the external naris (anterior margin) (Rauhut 2003; Yates 2007)

0 anterior to the midlength of the premaxillary body

1 posterior to the midlength of the premaxillary body

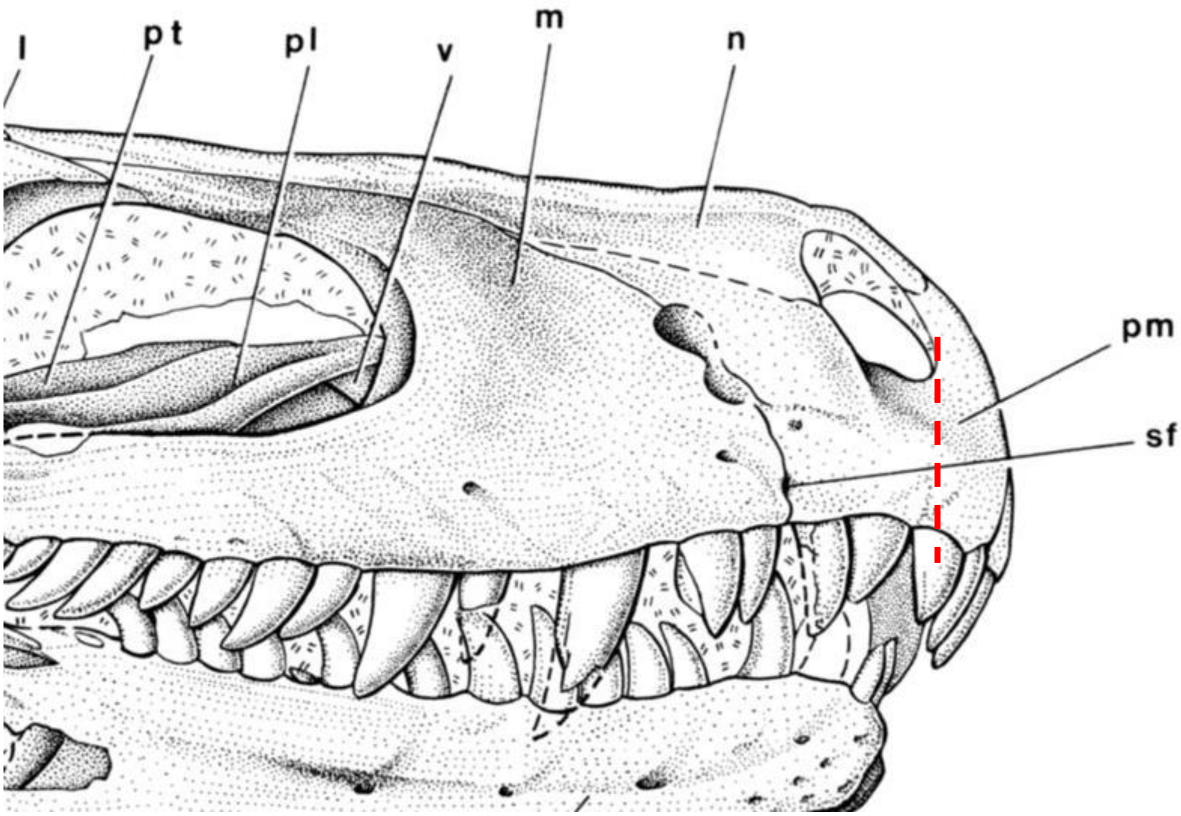

Herrerasaurus (0) (Image from: Sereno & Novas 1993; copyright © Society of Vertebrate Paleontology, www.vertpaleo.org, reprinted by permission of Taylor & Francis Ltd, http://www.tandfonline.com on behalf of Society of Vertebrate Paleontology)

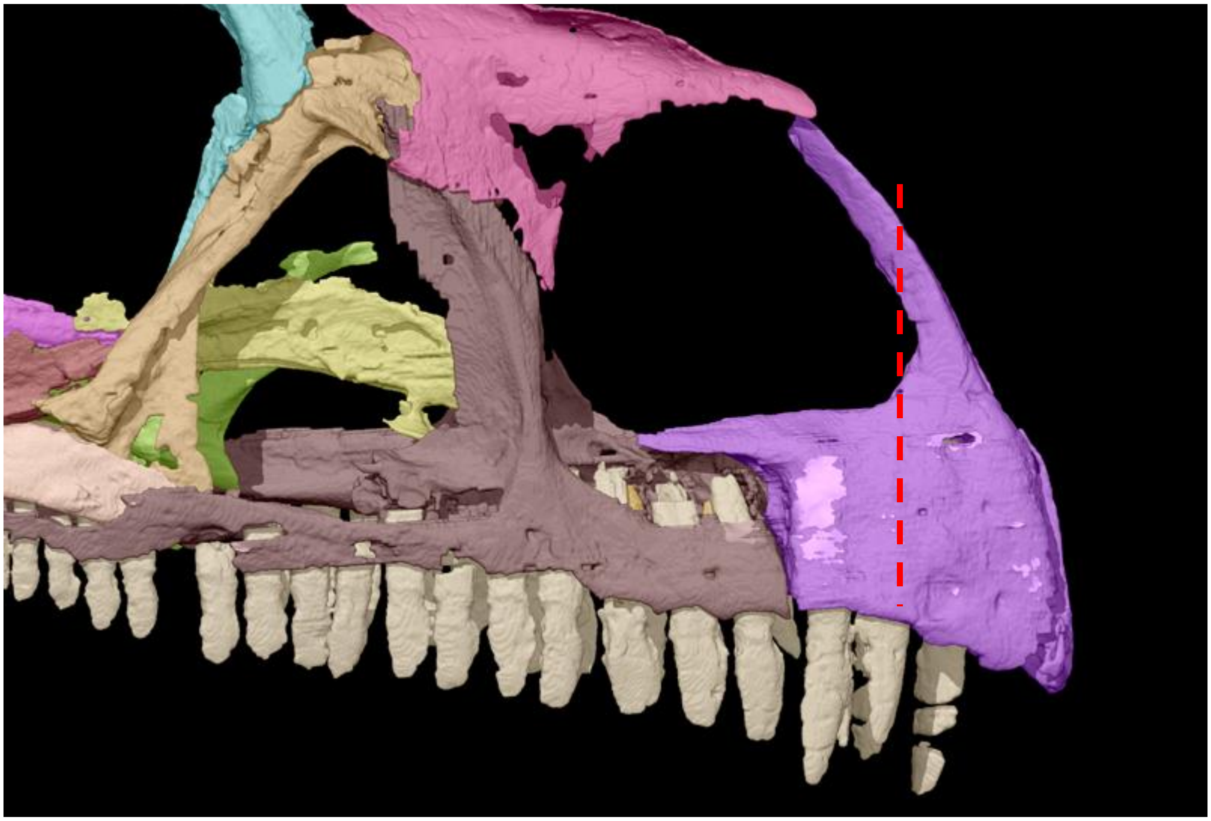

Massospondylus (1) (Image by: Kimberley Chapelle)

21. External naris: Level of the posteroventral corner of external naris (posterior margin) (modified from Wilson and Sereno 1998. Ordered, Yates 2007)

0 anterior to, or level with the premaxilla-maxilla suture

1 posterior to the first maxillary alveolus

2 posterior to the midlength of the maxillary tooth row and the anterior margin of the antorbital fenestra

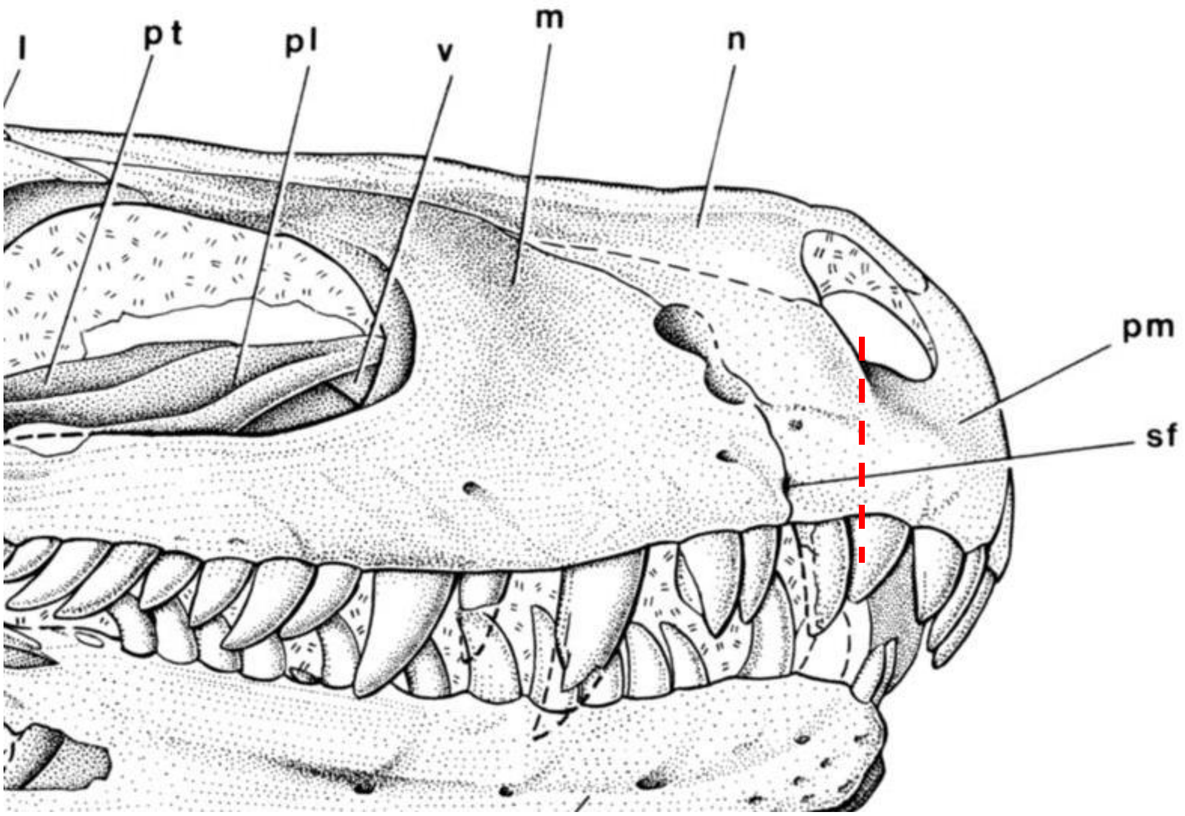

Herrerasaurus (0) (Image from: Sereno & Novas 1993; copyright © Society of Vertebrate Paleontology, www.vertpaleo.org, reprinted by permission of Taylor & Francis Ltd, http://www.tandfonline.com on behalf of Society of Vertebrate Paleontology)

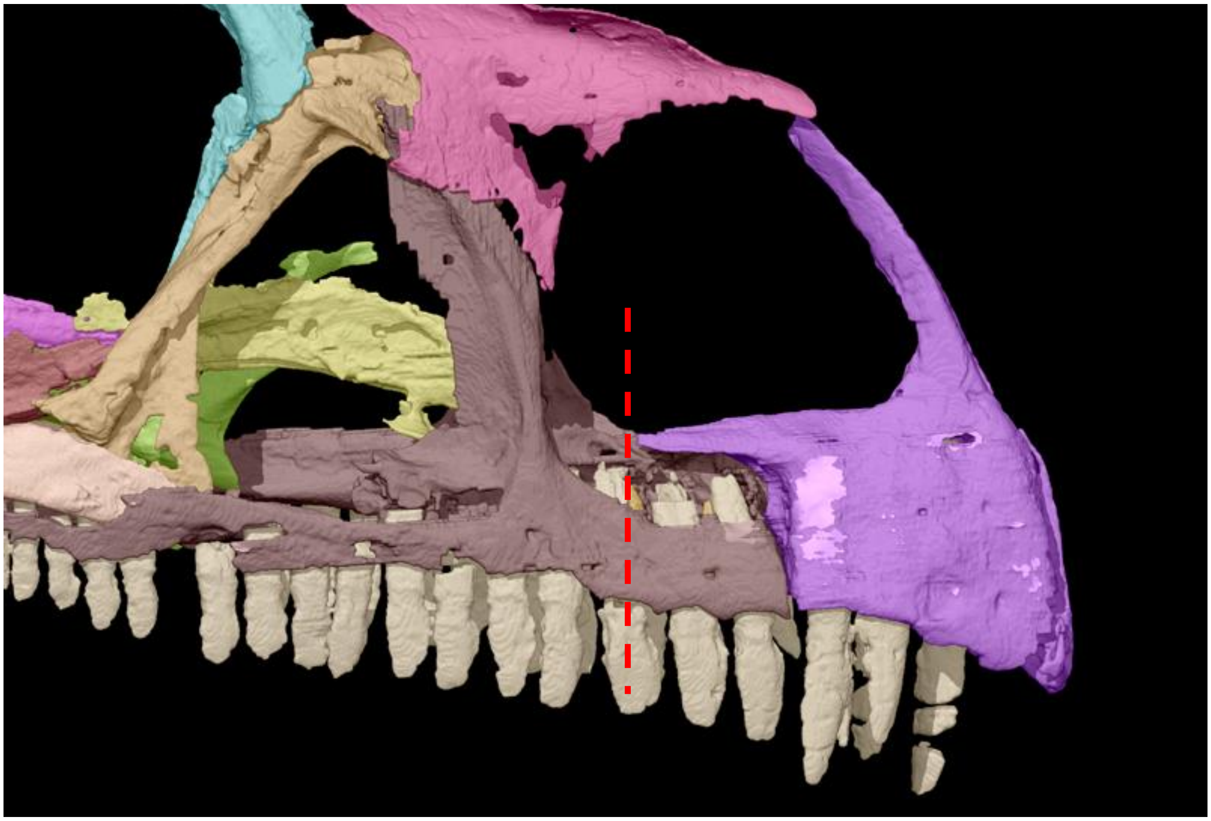

Massospondylus (1) (Image by: Kimberley Chapelle)

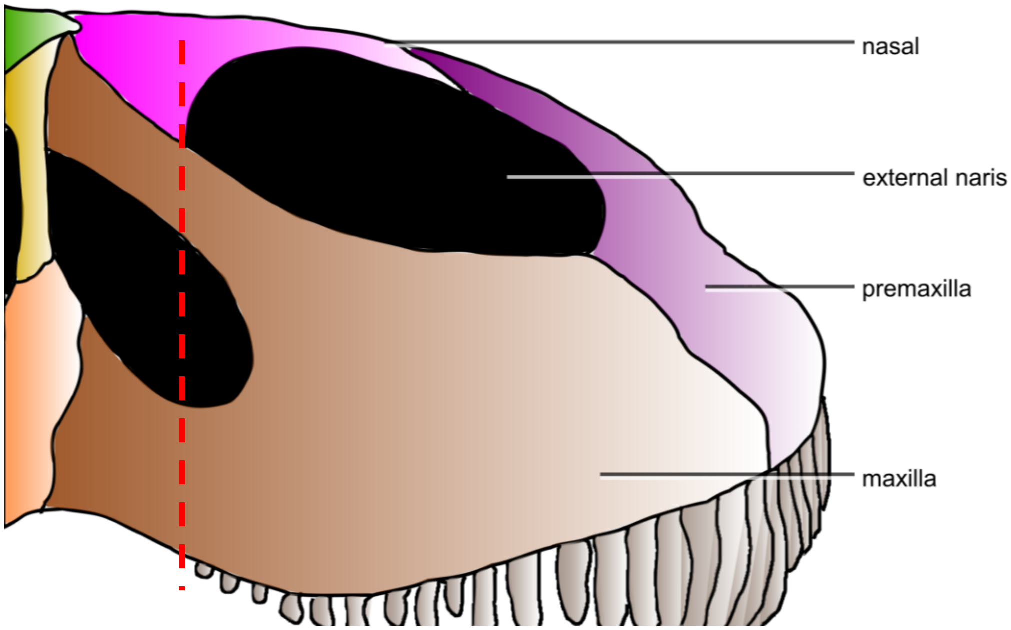

Shunosaurus (2) (Image by: Kimberley Chapelle, modified from Zheng 1996)

22. Snout: Dorsal margin of the snout in lateral view (Yates 2007 ch. 20)

   0 straight to gently convex

   1 with a depression behind the naris

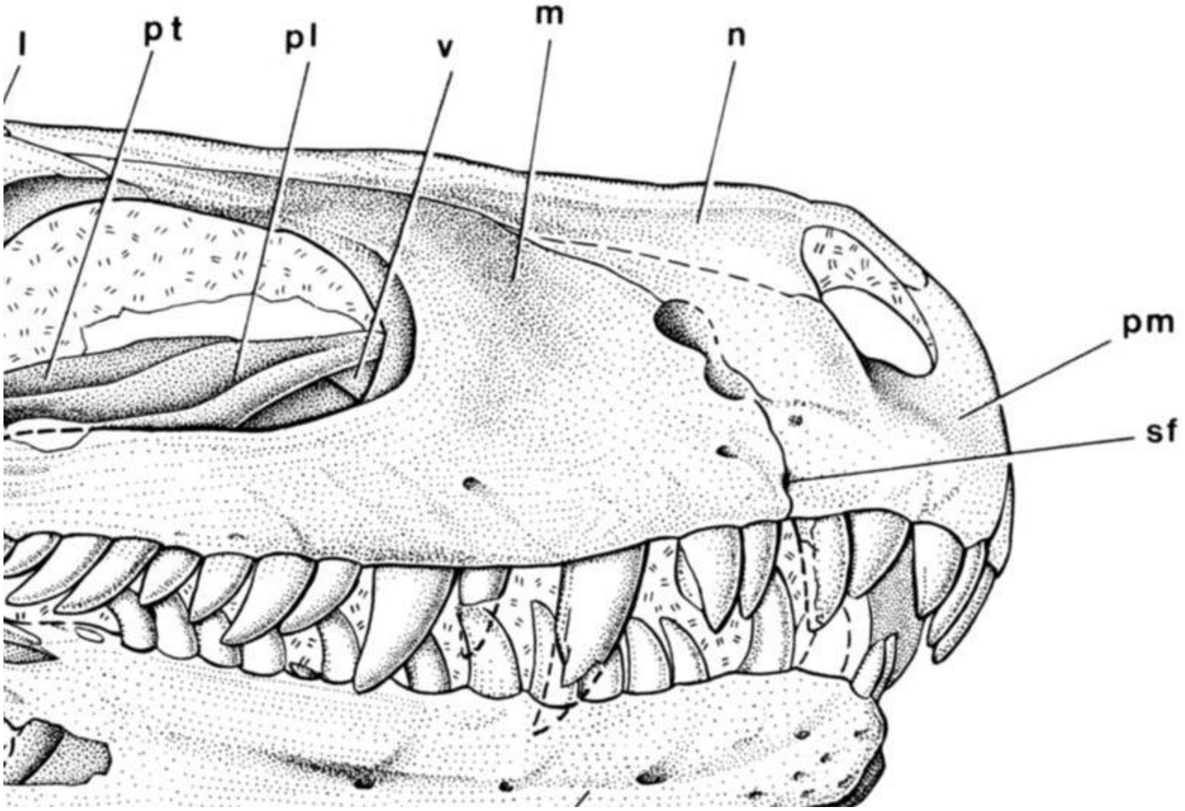

Herrerasaurus (0) (Image from: Sereno & Novas 1993; copyright © Society of Vertebrate Paleontology, www.vertpaleo.org, reprinted by permission of Taylor & Francis Ltd, http://www.tandfonline.com on behalf of Society of Vertebrate Paleontology)

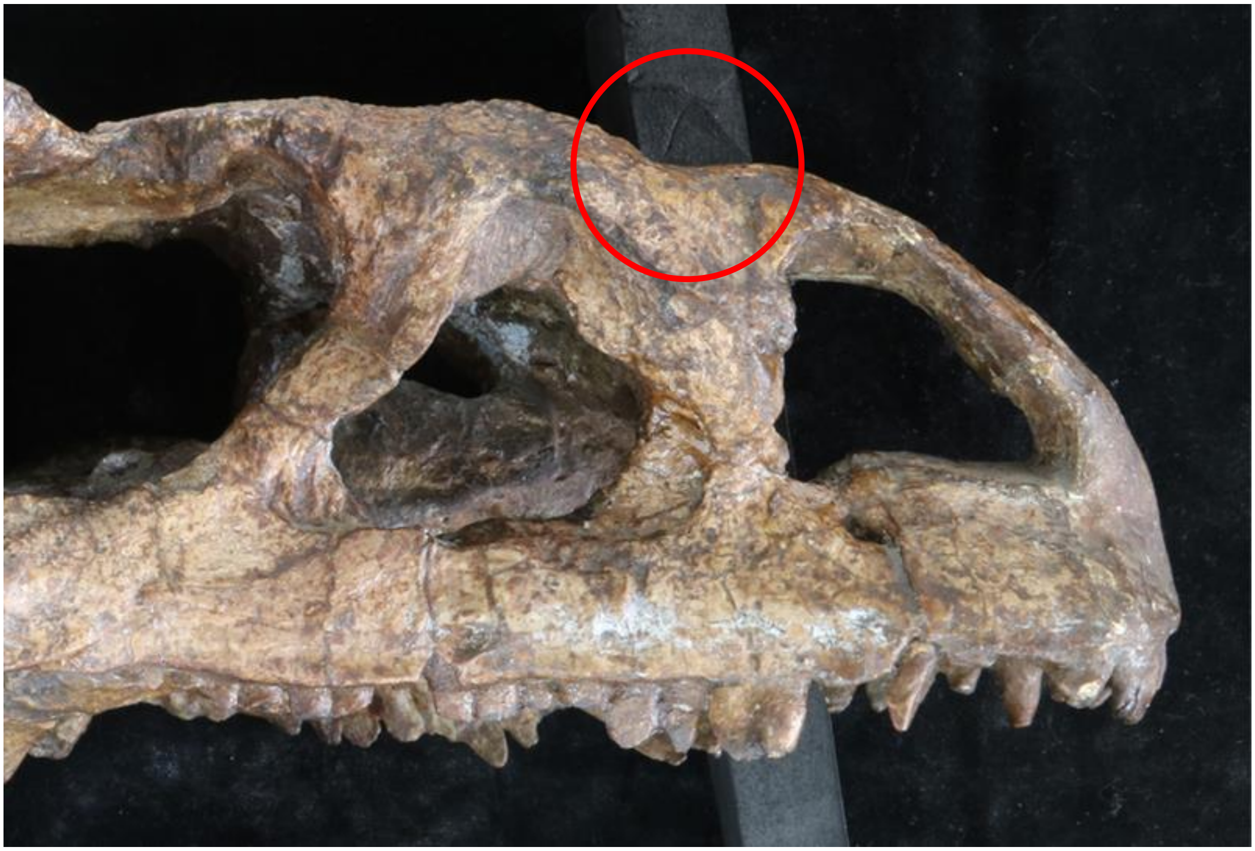

Lufengosaurus (1) (Image by: Kimberley Chapelle)

23. Nasal: Mediolateral width of maxillary ramus (anteroventral process) of nasal at its base (modified from Sereno 1999; Yates 2007 ch. 22)

 0 less than the width of the premaxillary ramus (anterodorsal process) at its base
 1 greater than the width of the premaxillary ramus (anterodorsal process) at its base

24. Nasal: contribution to margin of antorbital fossa (New character)

 0 absent
 1 present

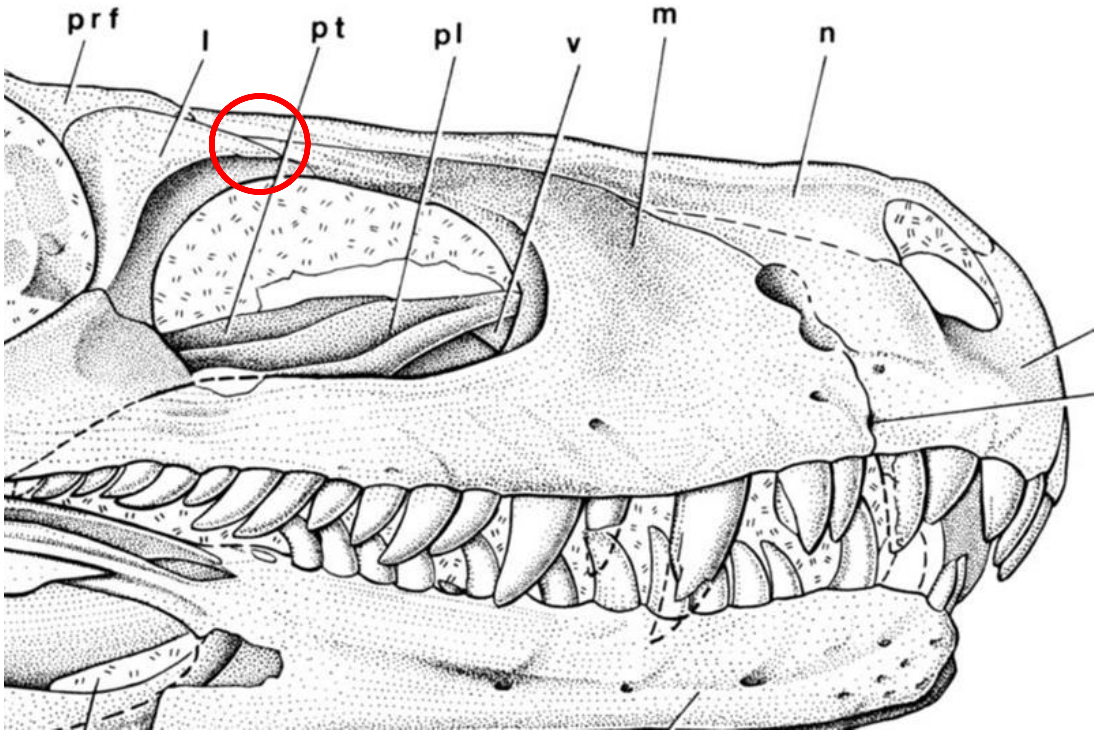

Herrerasaurus (0) (Image from: Sereno & Novas 1993; copyright © Society of Vertebrate Paleontology, www.vertpaleo.org, reprinted by permission of Taylor & Francis Ltd, http://www.tandfonline.com on behalf of Society of Vertebrate Paleontology)

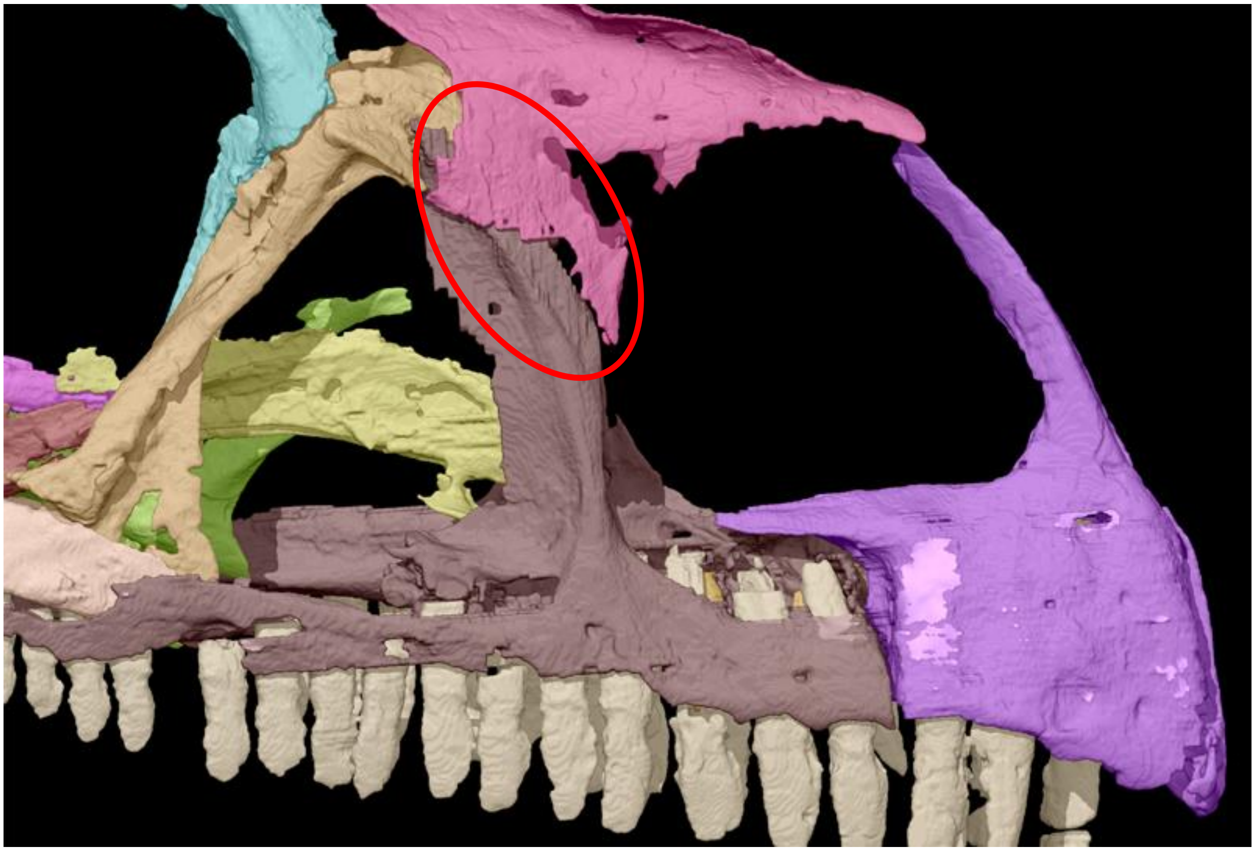

Massospondylus (1) (Image by: Kimberley Chapelle)

25. Nasal: relationship with dorsal margin of the antorbital fossa (modified from Sereno 1999; Yates 2007 ch. 23)

0 lateral margin overhangs the antorbital fossa and forms its dorsal margin

1 overhang extensive, obscuring the dorsal lacrimal/maxilla contact in lateral view

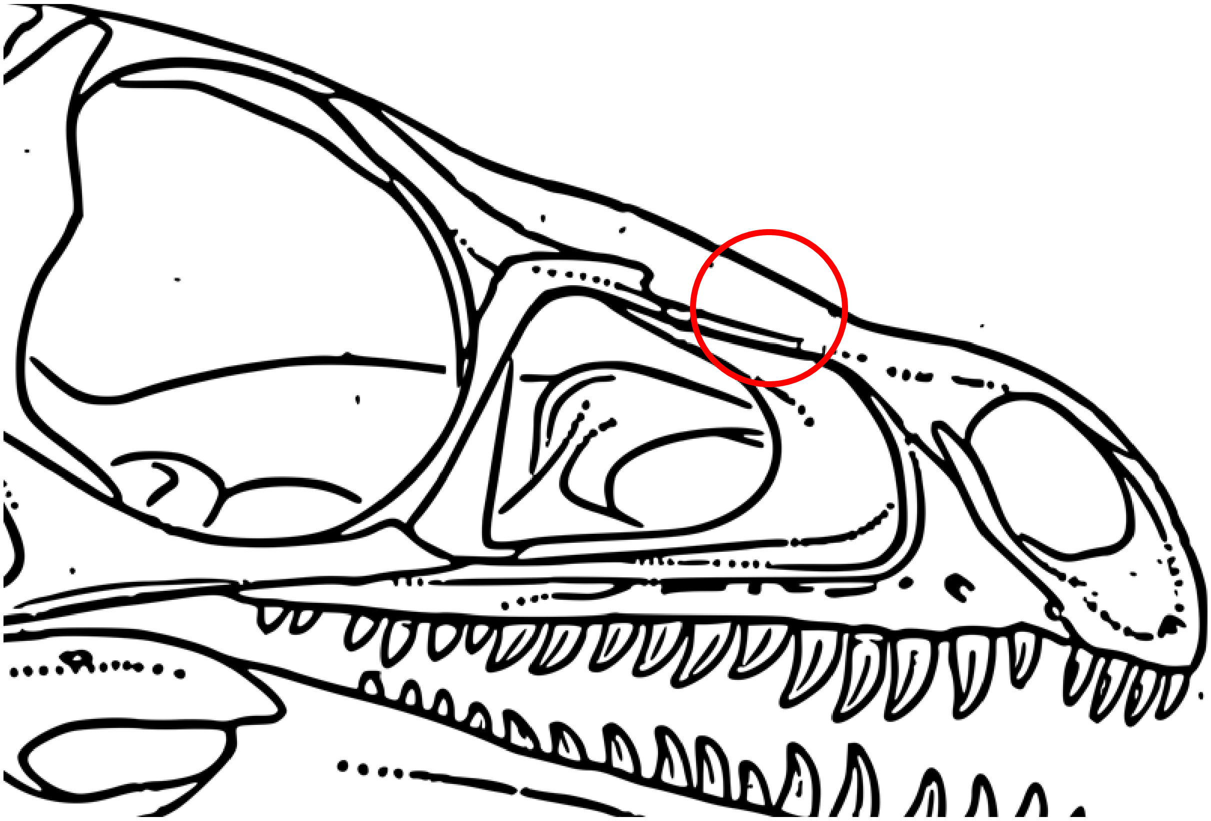

Eoraptor (0) (Image from: Sereno et al. 2012; copyright © Society of Vertebrate Paleontology, www.vertpaleo.org, reprinted by permission of Taylor & Francis Ltd, http://www.tandfonline.com on behalf of Society of Vertebrate Paleontology)

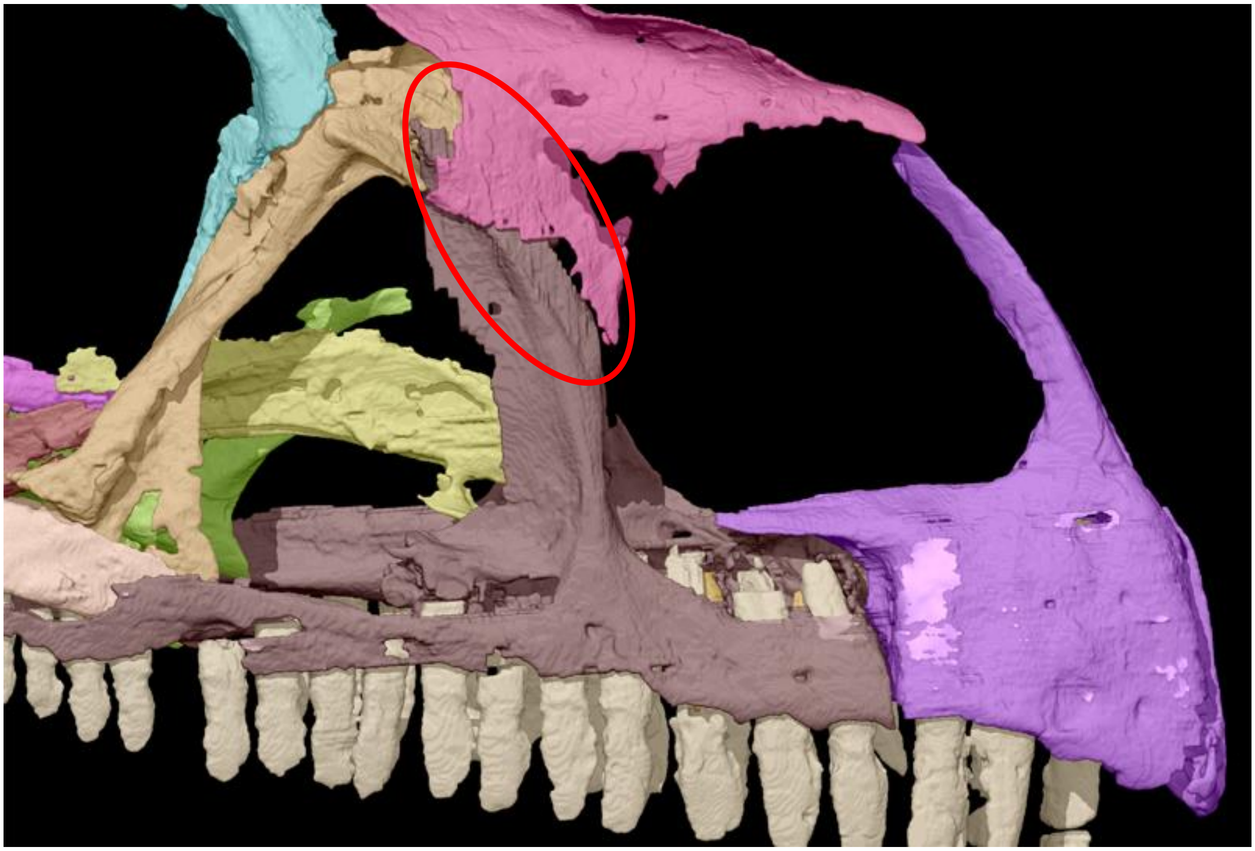
Massospondylus (1) (Image by: Kimberley Chapelle)

26. Nasal: Acuminate frontal ramus (posterolateral process) of the nasal overlapping the lacrimal (Sereno 1999; Yates 2007 ch. 24)

    0 absent
    1 present

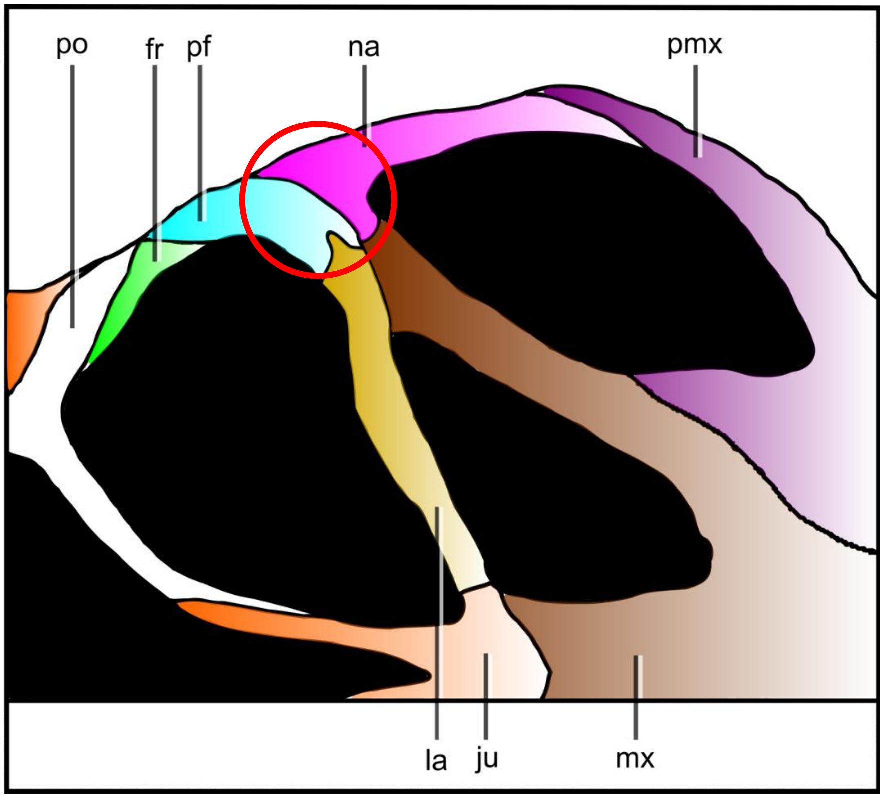

Mamenchisaurus (0) (Image by: Kimberley Chapelle, modified from Ouyang & Ye 2001). Abbreviations: fr: frontal; ju: jugal; la: lacrimal; mx: maxilla; na: nasal; pf: prefrontal; pmx : premaxilla ; po: postorbital

27. Maxilla: Anterior margin of the maxilla in lateral view (Sereno et al. 1996; Yates 2007 ch. 25)
    0 slopes continuously towards the anterior tip
    1 with a strong inflection at the base of the ascending ramus, creating an anterior ramus with parallel dorsal and ventral margins

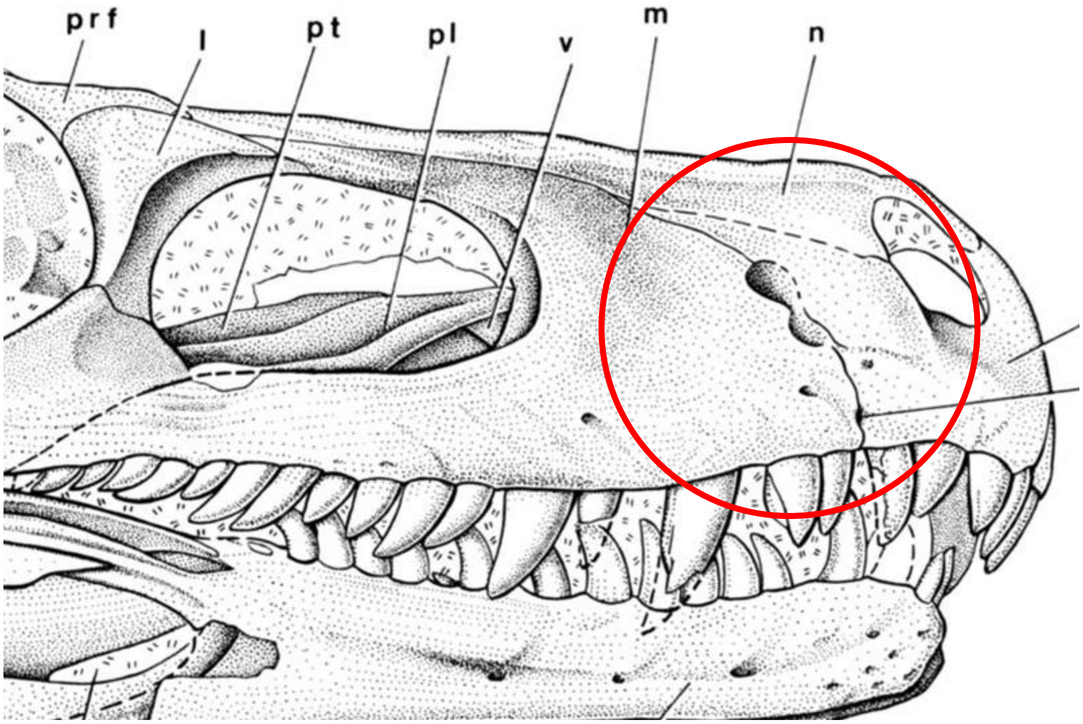

Herrerasaurus (0) (Image from: Sereno & Novas 1993; copyright © Society of Vertebrate Paleontology, www.vertpaleo.org, reprinted by permission of Taylor & Francis Ltd, http://www.tandfonline.com on behalf of Society of Vertebrate Paleontology)

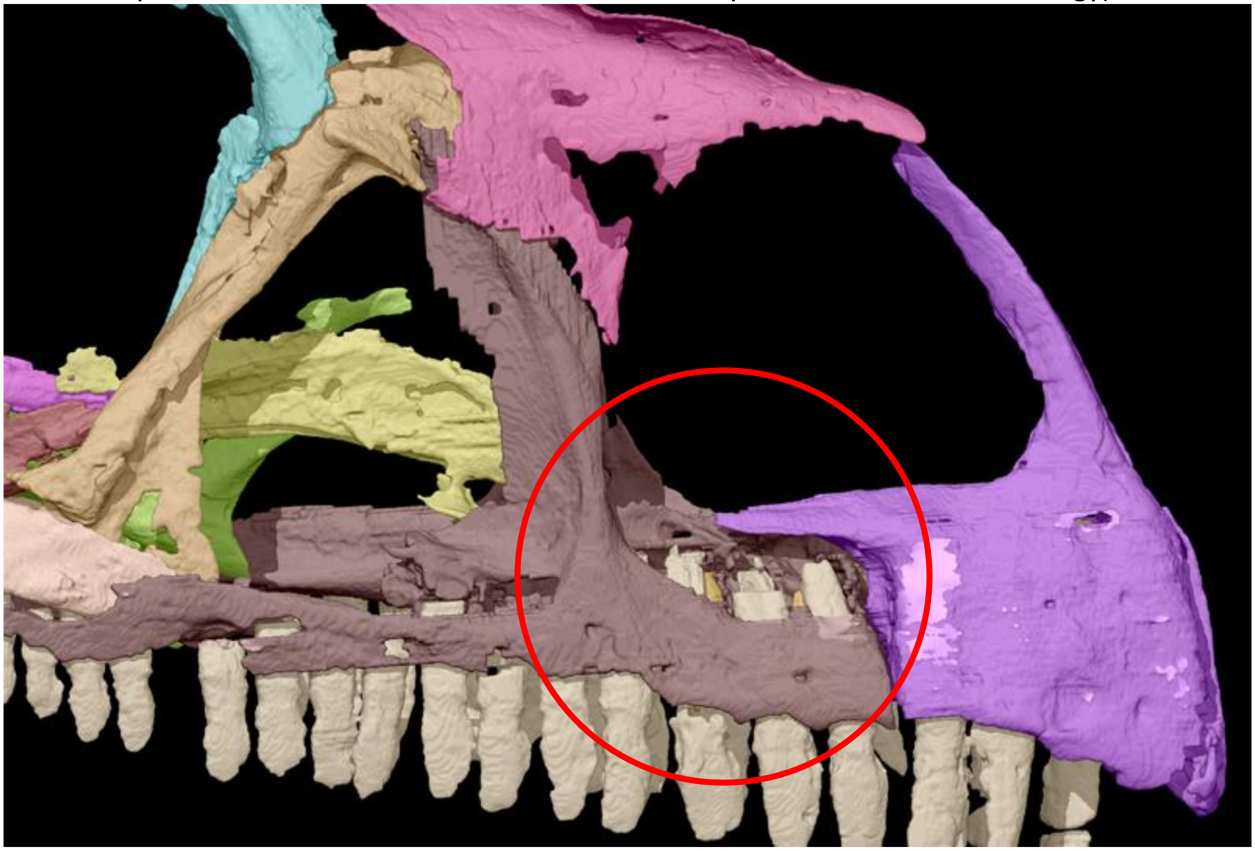

Massospondylus (1) (Image by: Kimberley Chapelle)

28. Maxilla: Length of premaxillary ramus of the maxilla (anterior ramus) (Sereno et al. 1996; Yates 2007 ch. 26)

0 less than its dorsoventral depth

1 greater than its dorsoventral depth

29. Maxilla: Shape of the jugal ramus of the maxilla (main body) in lateral view (Yates 2007 ch. 27)

0 tapering posteriorly

1 dorsal and ventral margins parallel for most of their length

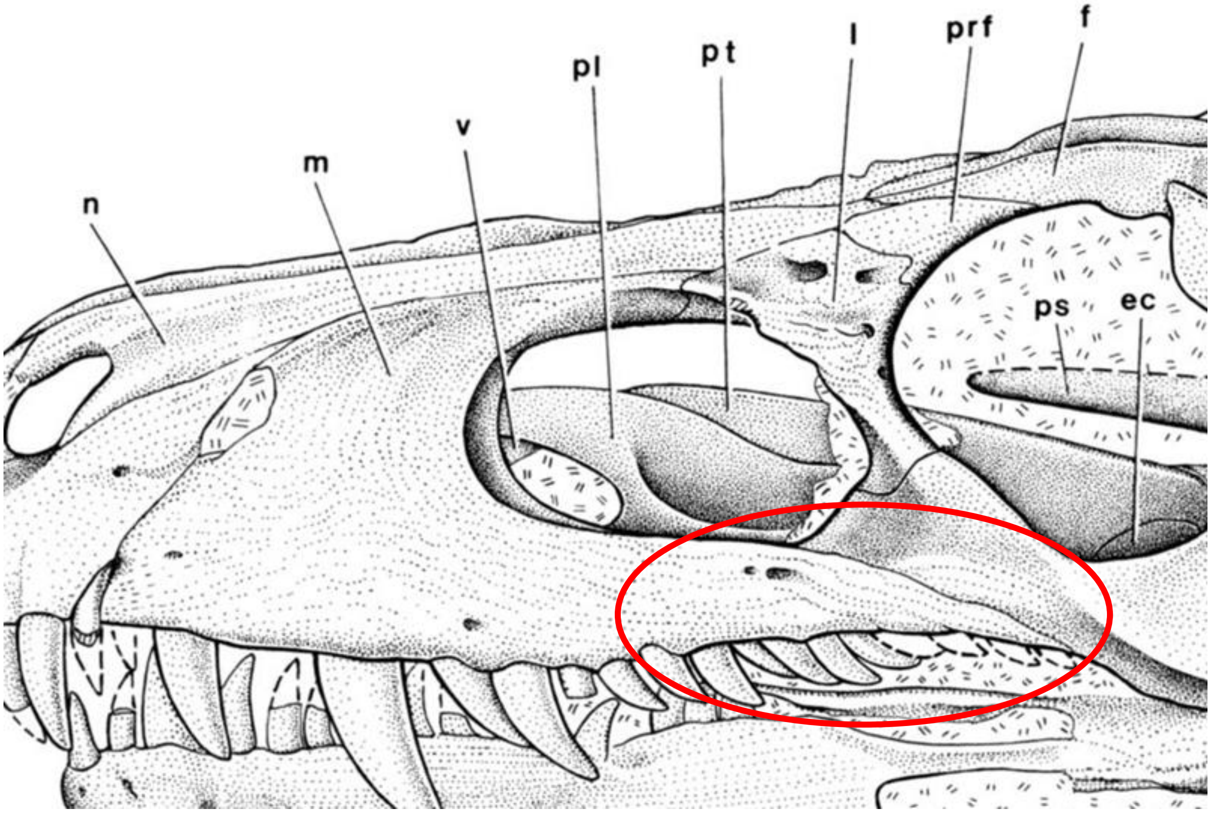

Herrerasaurus (0) (Image from: Sereno & Novas 1993; copyright © Society of Vertebrate Paleontology, www.vertpaleo.org, reprinted by permission of Taylor & Francis Ltd, http://www.tandfonline.com on behalf of Society of Vertebrate Paleontology)

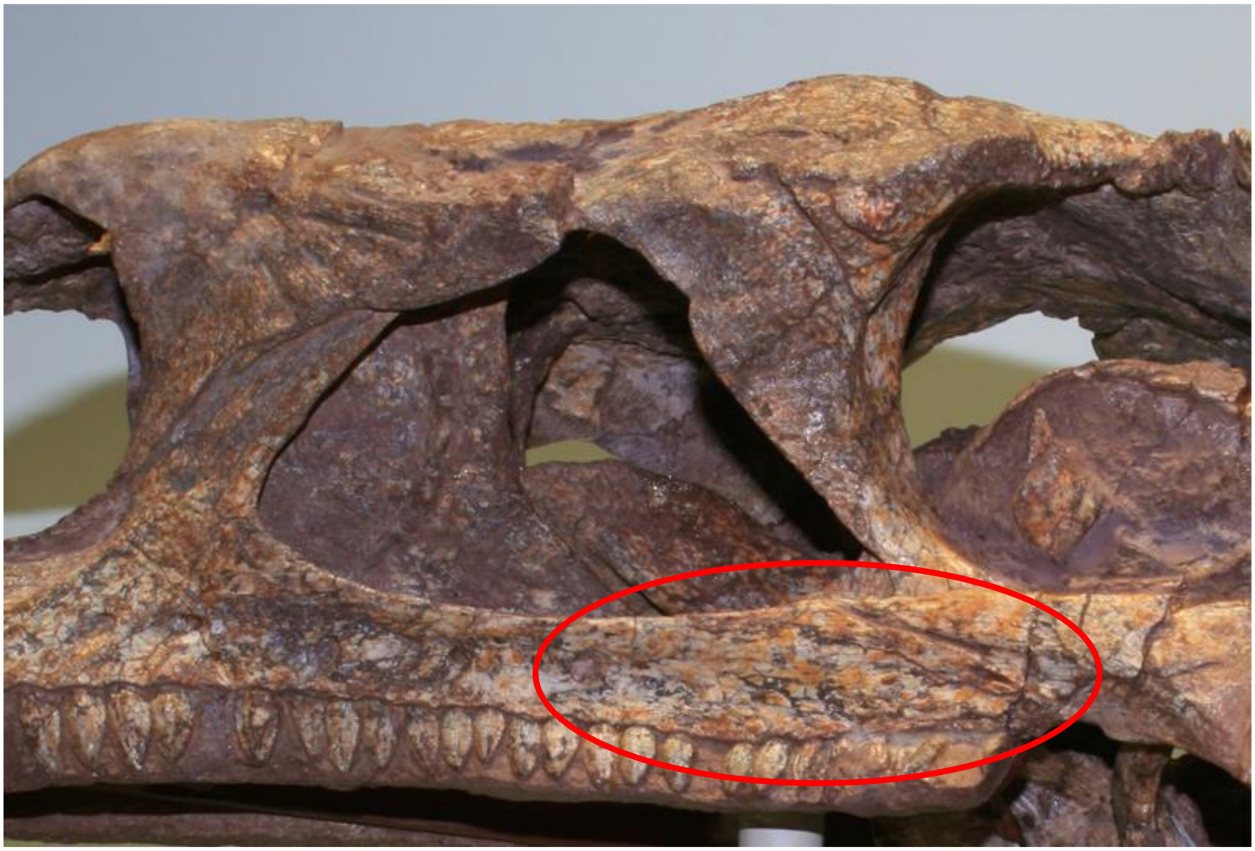

Plateosaurus engelhardti (1) (Image by: Kimberley Chapelle)

30. Antorbital fossa: Anteroposterior length of the antorbital fossa (Yates 2003; Yates 2007 ch. 29)
    - 0 greater than that of the orbit
    - 1 less than that of the orbit

31. Antorbital fossa: Posteroventral extent of medial wall of antorbital fossa (modified from Galton and Upchurch 2004; Yates 2007 ch. 30)
    - 0 reaching the anterior tip of jugal
    - 1 terminating anterior to the anterior tip of jugal

32. Antorbital fenestra: Posteroventral extent of antorbital fenestra (Originally from Holtz 1994, modified from Yates 2007 ch. 50)
    - 0 reaching the anterior tip of jugal
    - 1 terminating anterior to the anterior tip of jugal

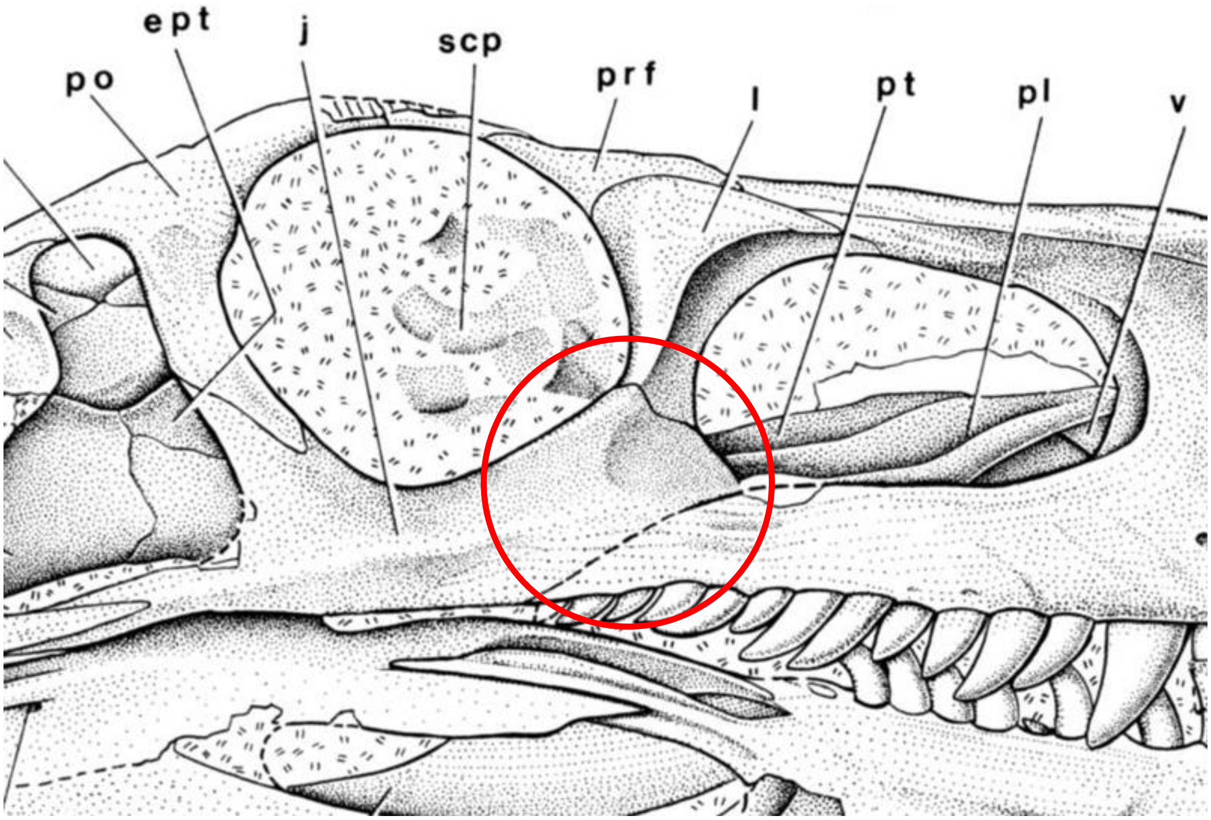

Herrerasaurus (0) (Image from: Sereno & Novas 1993; copyright © Society of Vertebrate Paleontology, www.vertpaleo.org, reprinted by permission of Taylor & Francis Ltd, http://www.tandfonline.com on behalf of Society of Vertebrate Paleontology)

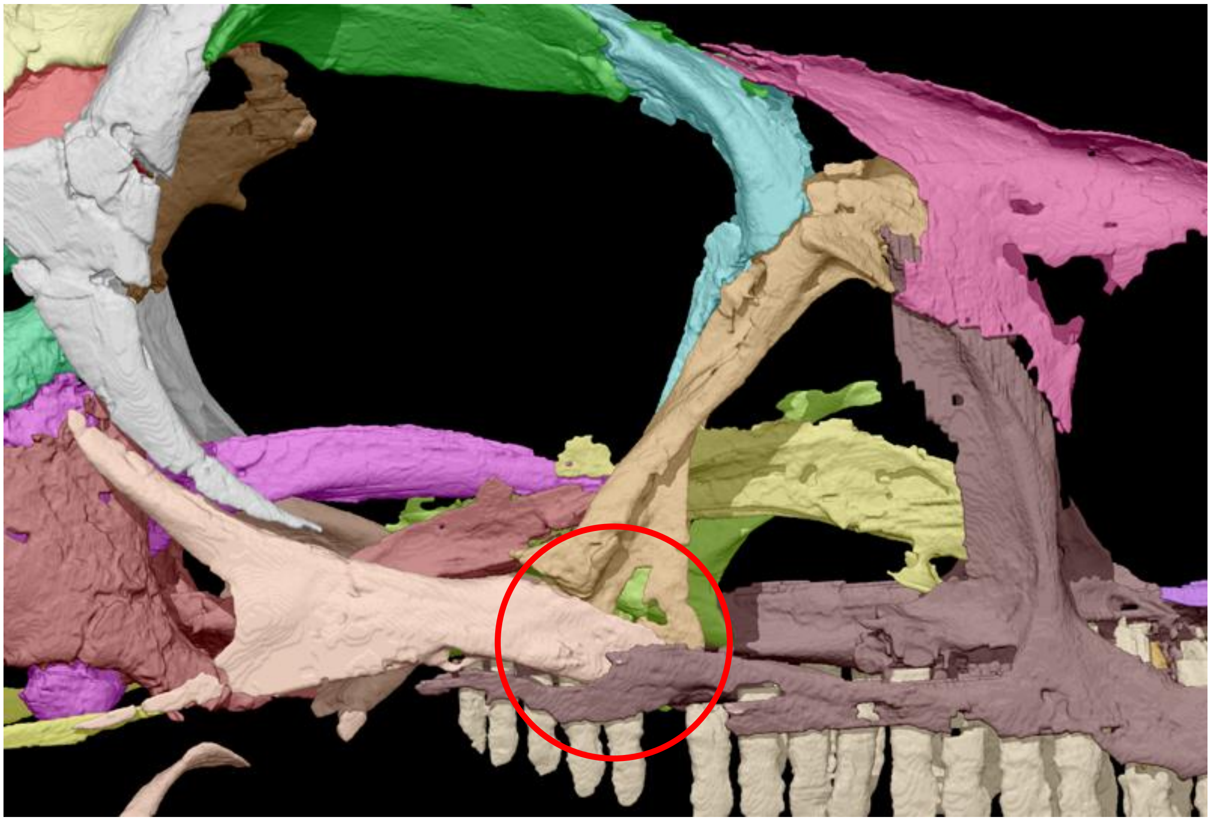

Massospondylus (1) (Image by: Kimberley Chapelle)

33. Maxilla: Development of the antorbital fossa on the lacrimal ramus (ascending ramus) of the maxilla (Yates 2007 ch. 31)

0 deeply impressed and delimited by a sharp, scarp-like rim

1 weakly impressed and delimited by a rounded rim or a change in slope

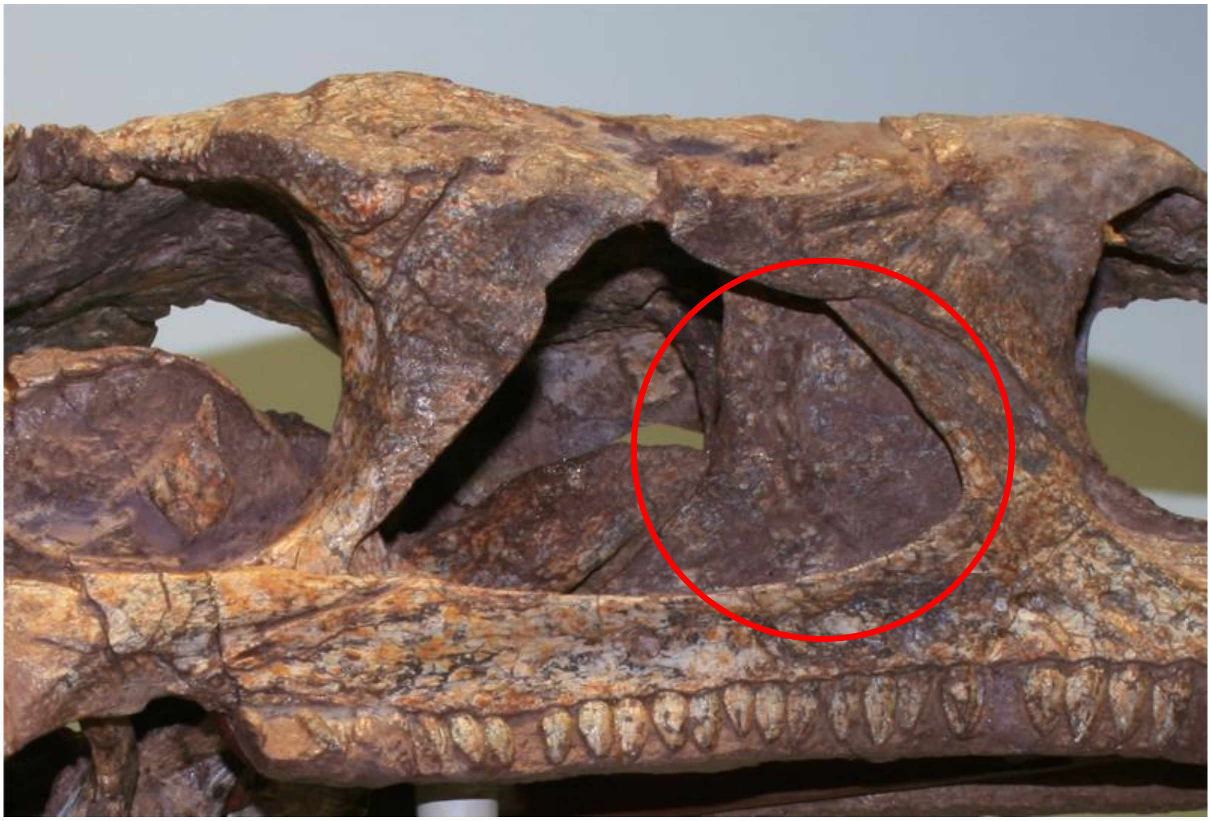

Plateosaurus engelhardti (0) (Image by: Kimberley Chapelle)

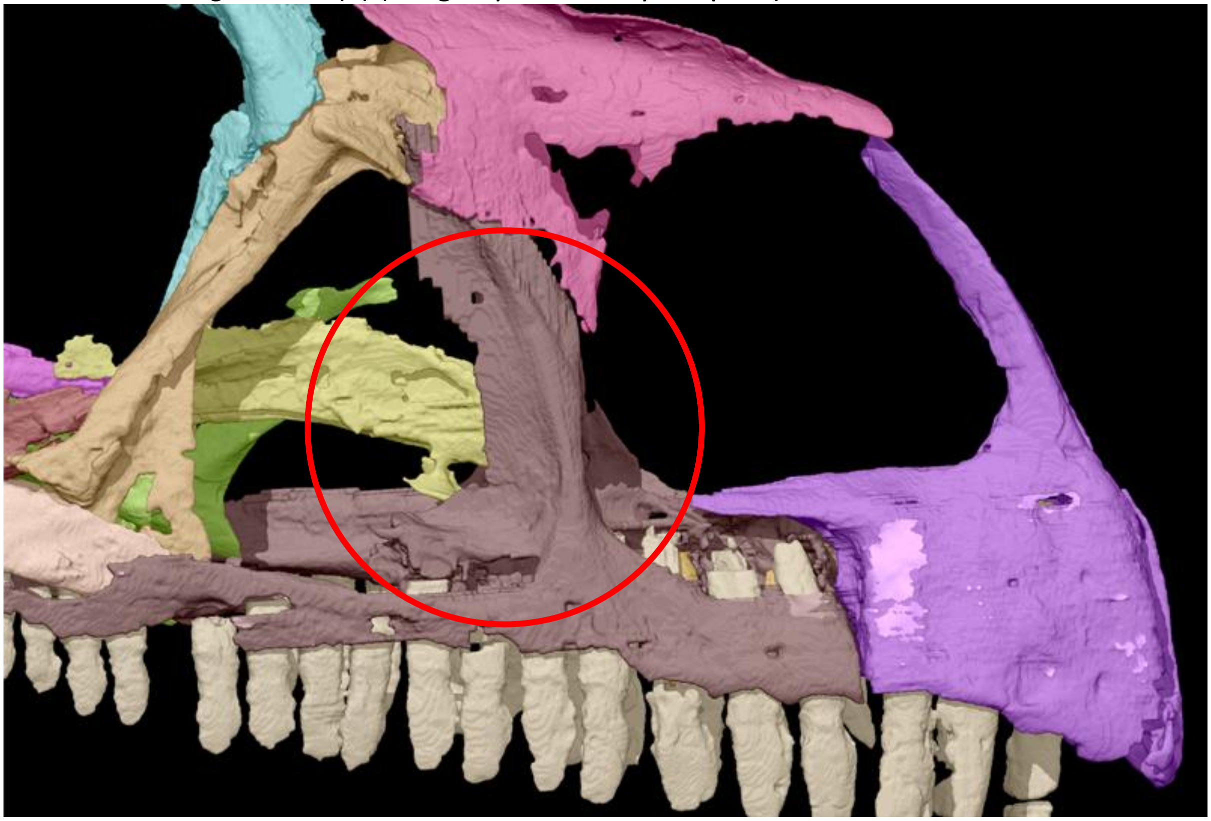

Massospondylus (1) (Image by: Kimberley Chapelle)

34. Antorbital fossa (New character)
  0 absent
  1 present

35. Antorbital fossa: Shape of the antorbital fossa (modified from Galton 1985a; Yates 2007 ch. 32.)
  0 crescentic with a strongly concave posterior margin that is roughly parallel to the anterior margin of the antorbital fossa
  1 subtriangular with a straight to gently concave posterior margin

36. Neurovascular foramina: Size of the neurovascular foramen at the posterior end of (Yates 2003b; Yates 2007 ch. 33)
  0 not larger than the others
  1 distinctly larger than the others in the row

37. Neurovascular foramina: Opening direction of the neurovascular foramen at the posterior end of the lateral maxillary row (modified from Sereno 1999; Yates 2007 ch. 34)
  0 posteriorly
  1 anteriorly, ventrally or laterally

38. Neurovascular foramina: Arrangement of lateral maxillary neurovascular foramina (modified from Sereno 1999; Yates 2007 ch. 35)
  0 linear
  1 irregular

39. Maxilla: Longitudinal ridge on the posterior end of the lateral surface of the maxilla (Barrett et al. 2005; Yates 2007 ch. 36)
  0 absent
  1 present

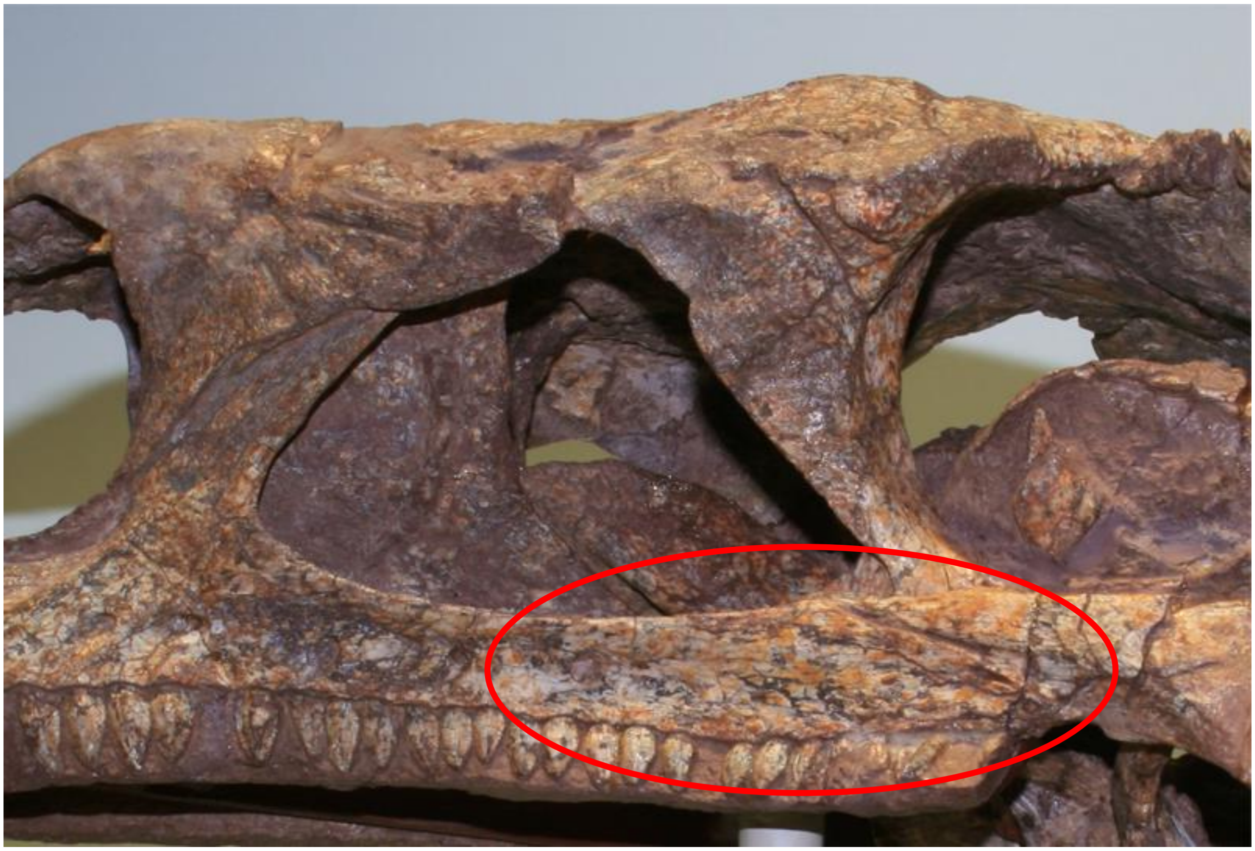
Plateosaurus engelhardti (0) (Image by: Kimberley Chapelle)

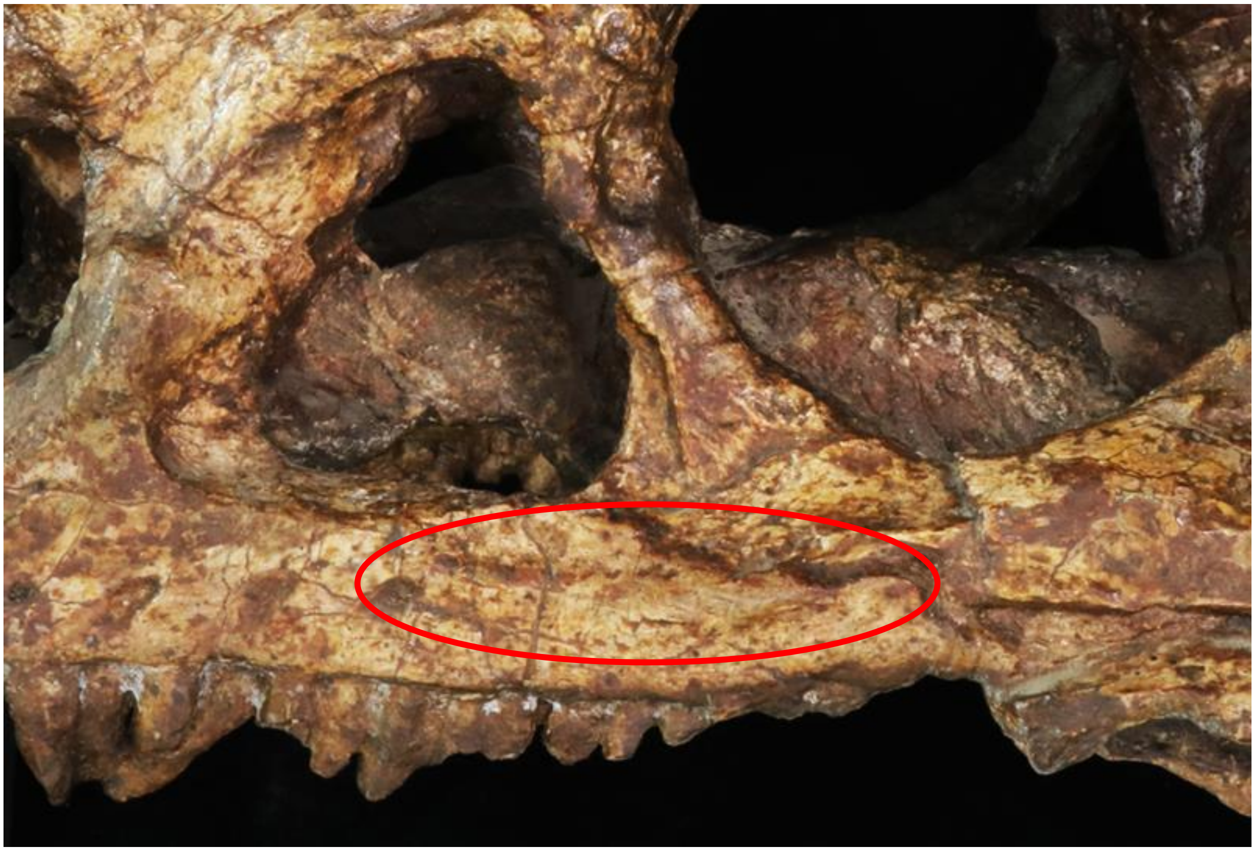
Lufengosaurus (1) (Image by: Kimberley Chapelle)

40. Lacrimal: Exposure of lacrimal on dorsal surface of the skull (Gauthier 1986; Yates 2007 ch. 37)

    0 present
    1 absent

41. Lacrimal: Shape of lacrimal in lateral view (Rauhut 2003; Yates 2007 ch. 38)

    0 dorsoventrally short and blockshaped
    1 dorsoventrally elongate and shaped like an inverted L

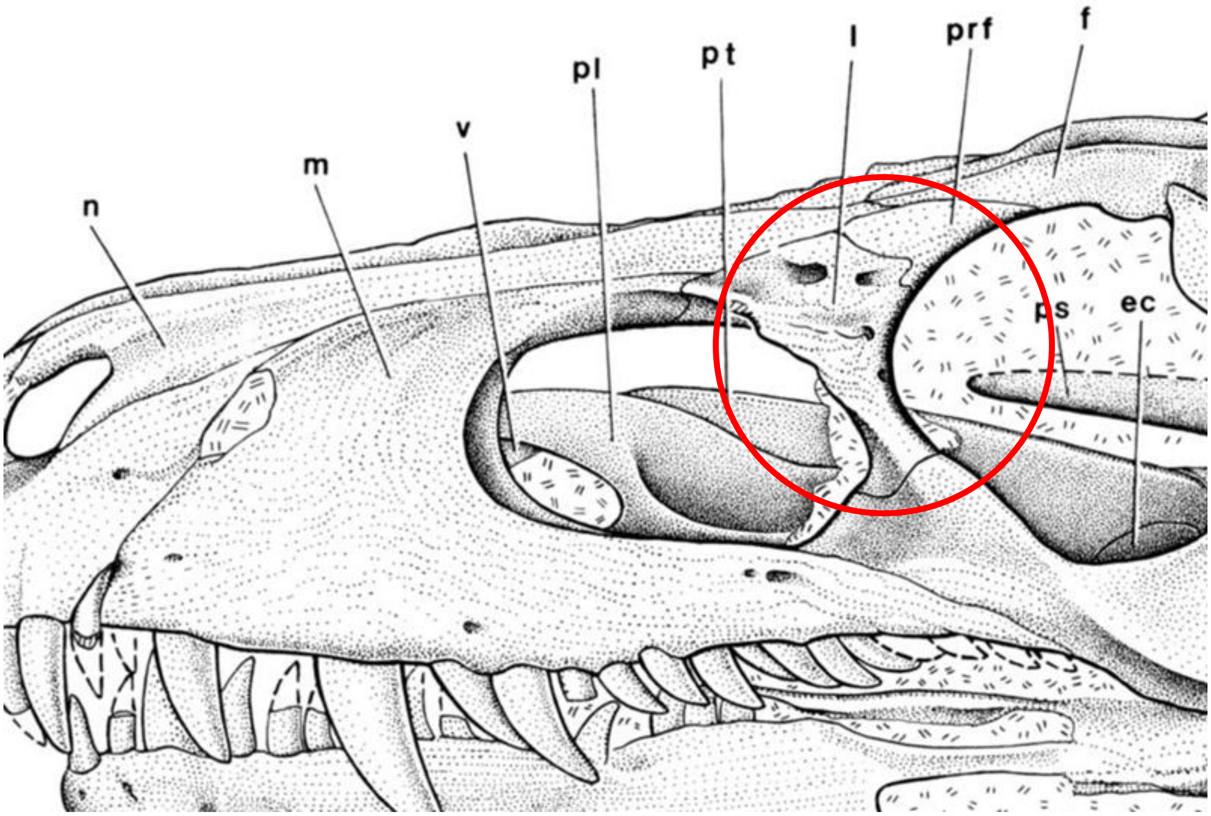

Herrerasaurus (0) (Image from: Sereno & Novas 1993; copyright © Society of Vertebrate Paleontology, www.vertpaleo.org, reprinted by permission of Taylor & Francis Ltd, http://www.tandfonline.com on behalf of Society of Vertebrate Paleontology)

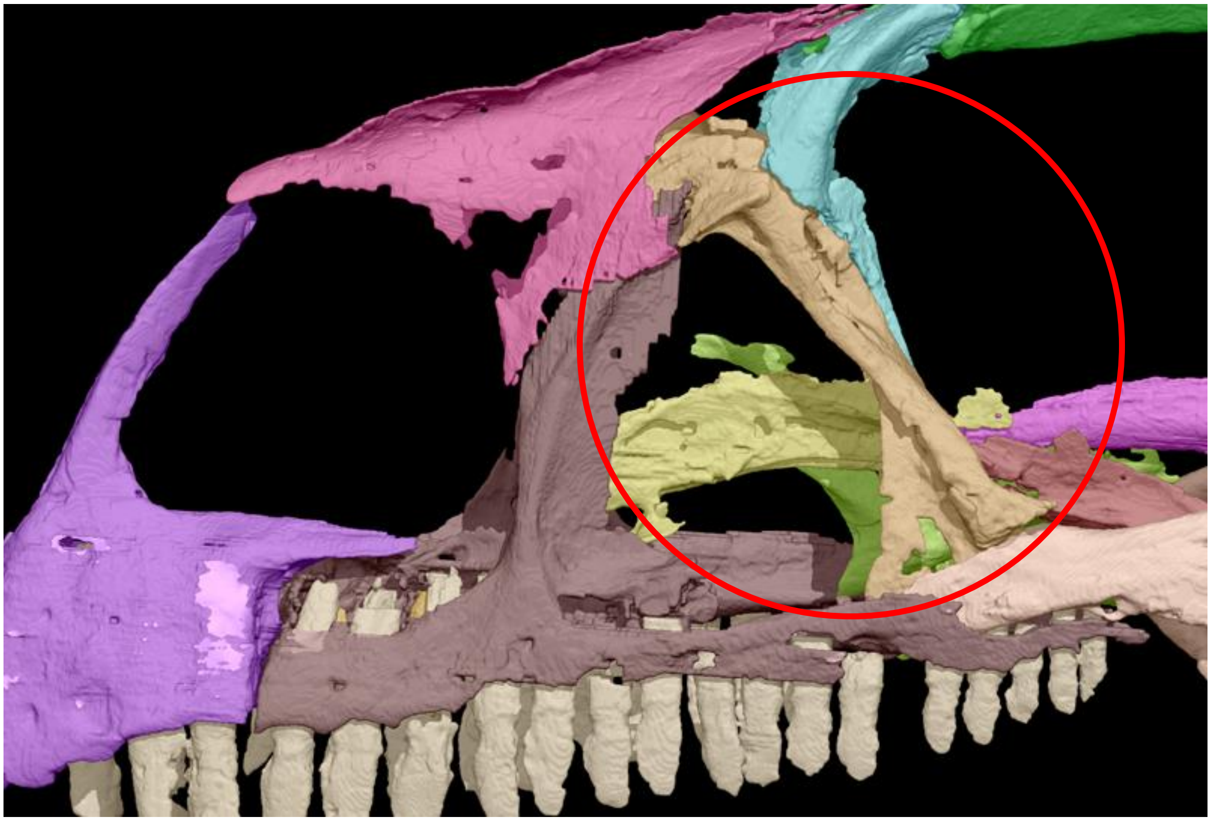

Massospondylus (1) (Image by: Kimberley Chapelle)

42. Orientation of the lacrimal orbital margin (Yates 2007 ch. 39)
    0 strongly sloping anterodorsally
    1 erect and close to vertical

43. Lacrimal: Anterior ramus of the lacrimal (New character)
    0 absent
    1 present

44. Lacrimal: Length of the anterior ramus of the lacrimal (modified from Galton 1990. Ordered, Yates 2007 ch. 40)
    0 greater than half the length of the ventral ramus
    1 less than half the length of the ventral ramus

45. Lacrimal: Sheet of bone present at the lateral boundary of the junction between anterior and ventral rami of lacrimal (Yates 2007 ch. 41)
    0 absent and antorbital fossa laterally exposed
    1 present, obscuring posterodorsal corner of antorbital fossa

46. Lacrimal: Extension of the antorbital fossa onto the ventral end of the lacrimal (modified from Wilson and Sereno 1998; Yates 2007 ch. 42)
    0 present

1 absent

47. Prefrontal: Lacrimal ramus of prefrontal extending down the posteromedial side of the lacrimal (Wilson and Sereno 1998; Yates 2007 ch. 44)

0 present

1 absent

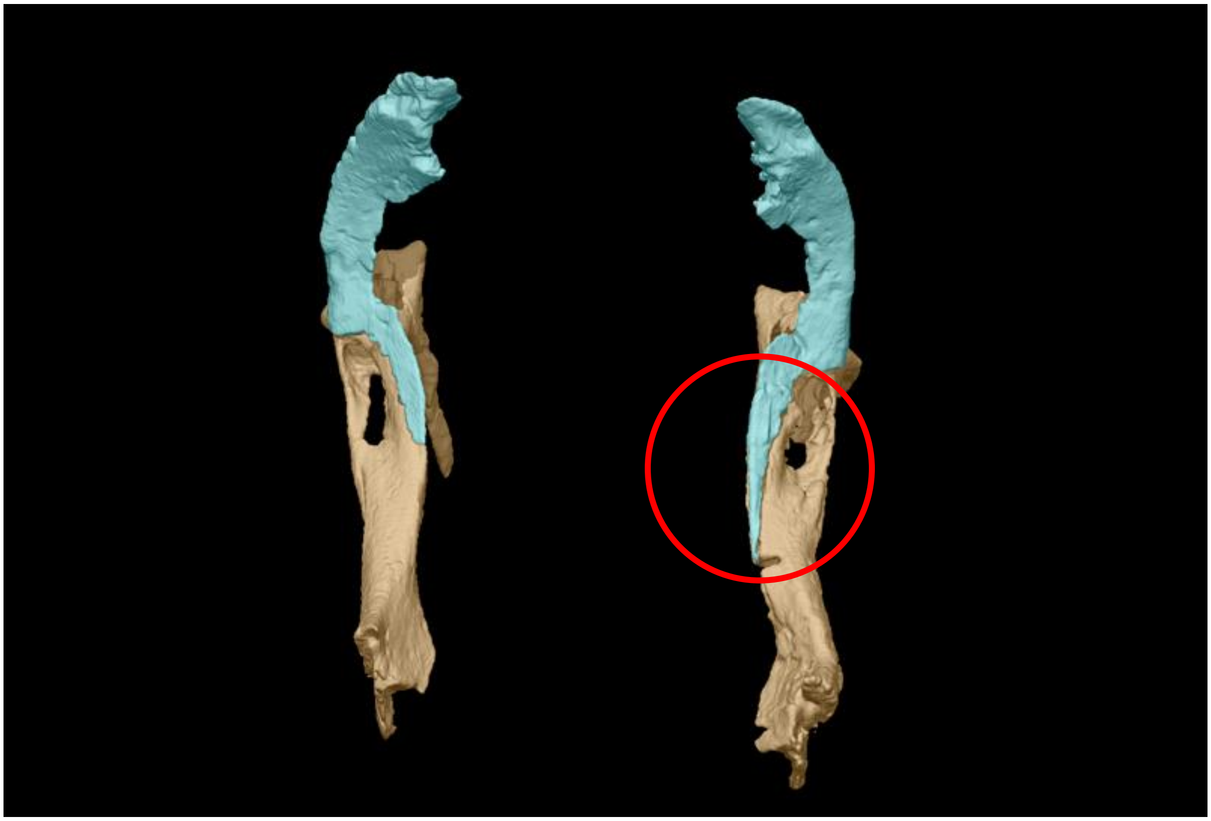

Massospondylus (0) (Image by: Kimberley Chapelle)

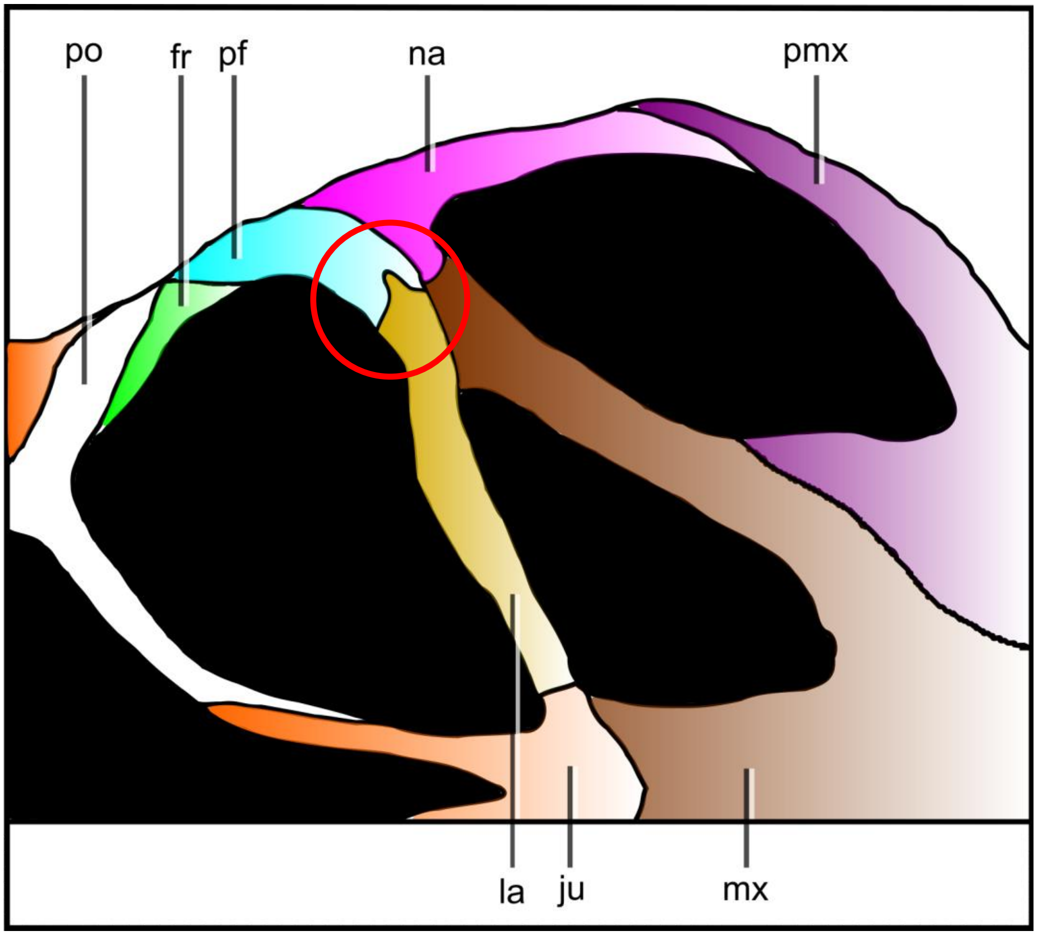

Mamenchisaurus (1) (Image by: Kimberley Chapelle; modified from Ouyang & Ye 2001). Abbreviations: fr: frontal; ju: jugal; la: lacrimal; mx: maxilla; na: nasal; pf: prefrontal; pmx : premaxilla ; po: postorbital

48. Prefrontal: Dorsoventral height of the lacrimal ramus (ventral process) of the prefrontal (New character). In some taxa, such as *Lufengosaurus,* the lacrimal ramus of the prefrontal only extends along a short distance of the dorsoventral height of the lacrimal. In other taxa, such as *Massospondylus*, the dorsoventral height of this process is more than 0.5 times that of the jugal ramus of the lacrimal.

0 more than 0.5 times that of the jugal ramus (ventral ramus) of lacrimal

1 less than 0.5 times that of the jugal ramus (ventral ramus) of the lacrimal

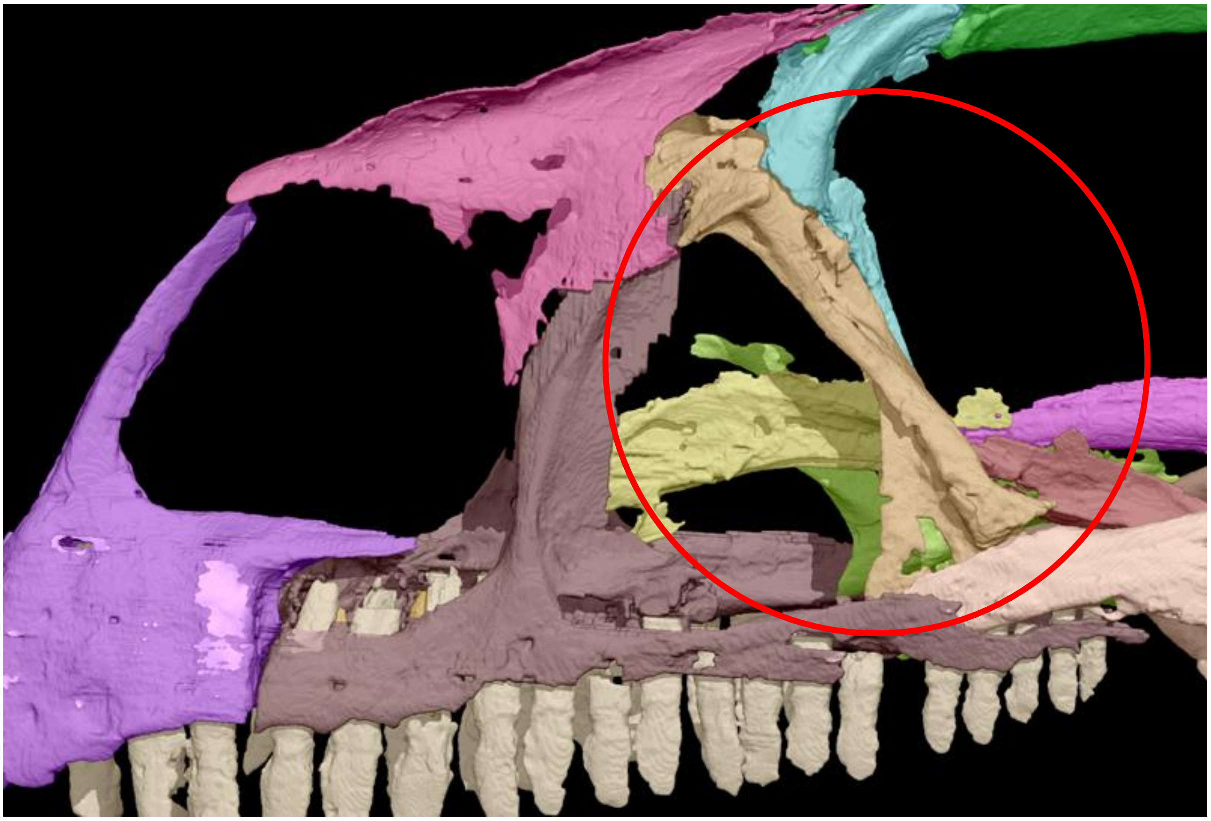

Massospondylus (0) (Image by: Kimberley Chapelle)

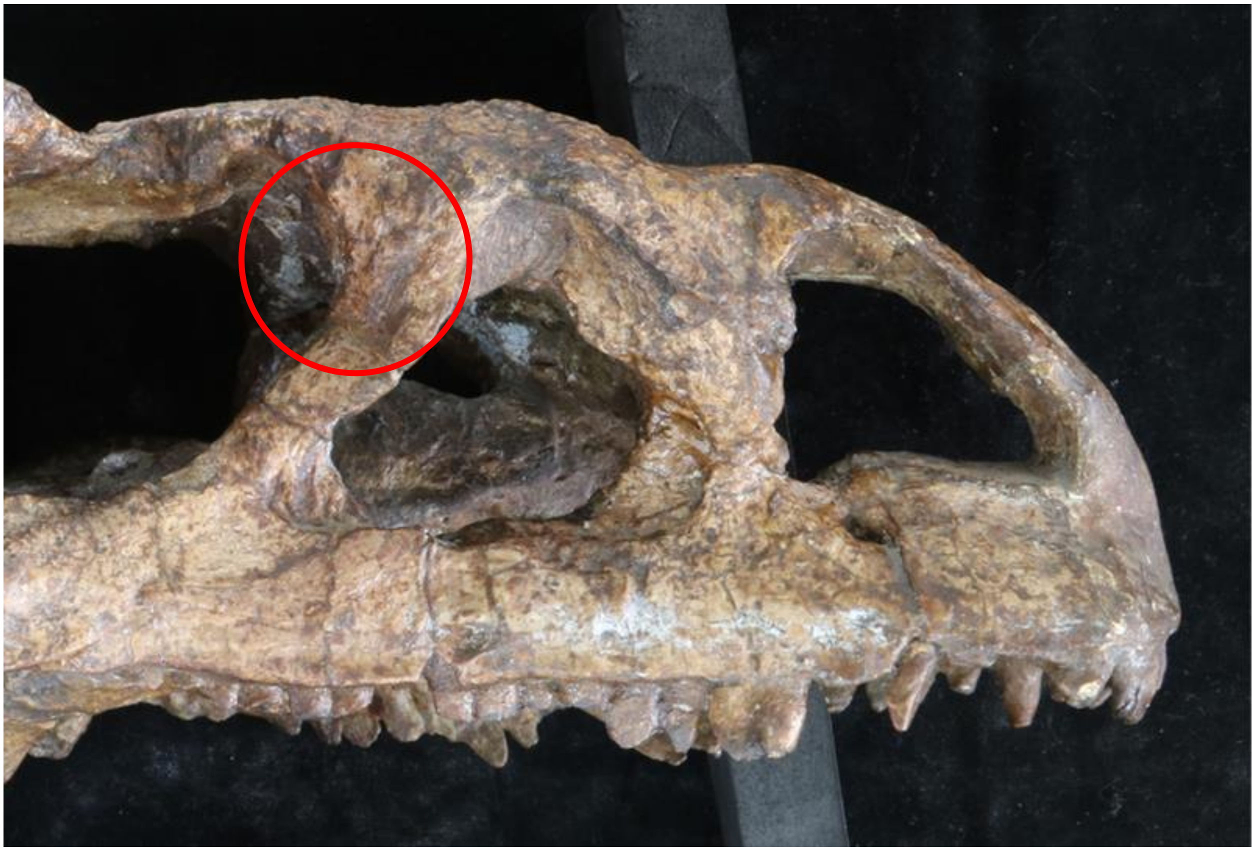

Lufengosaurus (1) (Image by: Kimberley Chapelle)

49. Prefrontal: Maximum transverse width of the prefrontal (modified from Galton 1990; Yates 2007 ch. 45)
    0 less than 0.25 of the skull width at that level
    1 more than 0.25 of the skull width at that level

50. Orbit: Shape in lateral view (Wilson and Sereno 1998; Yates 2007 ch. 46)
    0 subcircular
    1 ventrally constricted making the orbit subtriangular

51. Frontal: Slender anterior process of the frontal intruding between the prefrontal and the nasal (modified from Sereno 1999; Yates 2007 ch. 47)
    0 absent
    1 present

52. Jugal: Jugal-lacrimal relationship (Sereno et al. 1993; Yates 2007 ch. 48)
    0 lachrymal overlapping lateral surface of jugal or abutting it dorsally
    1 jugal overlapping lachrymal laterally

53. Jugal: Shape of the suborbital region of the jugal (Yates 2007 ch. 49)

0 an anteroposteriorly elongate bar
1 an anteroposteriorly shortened plate

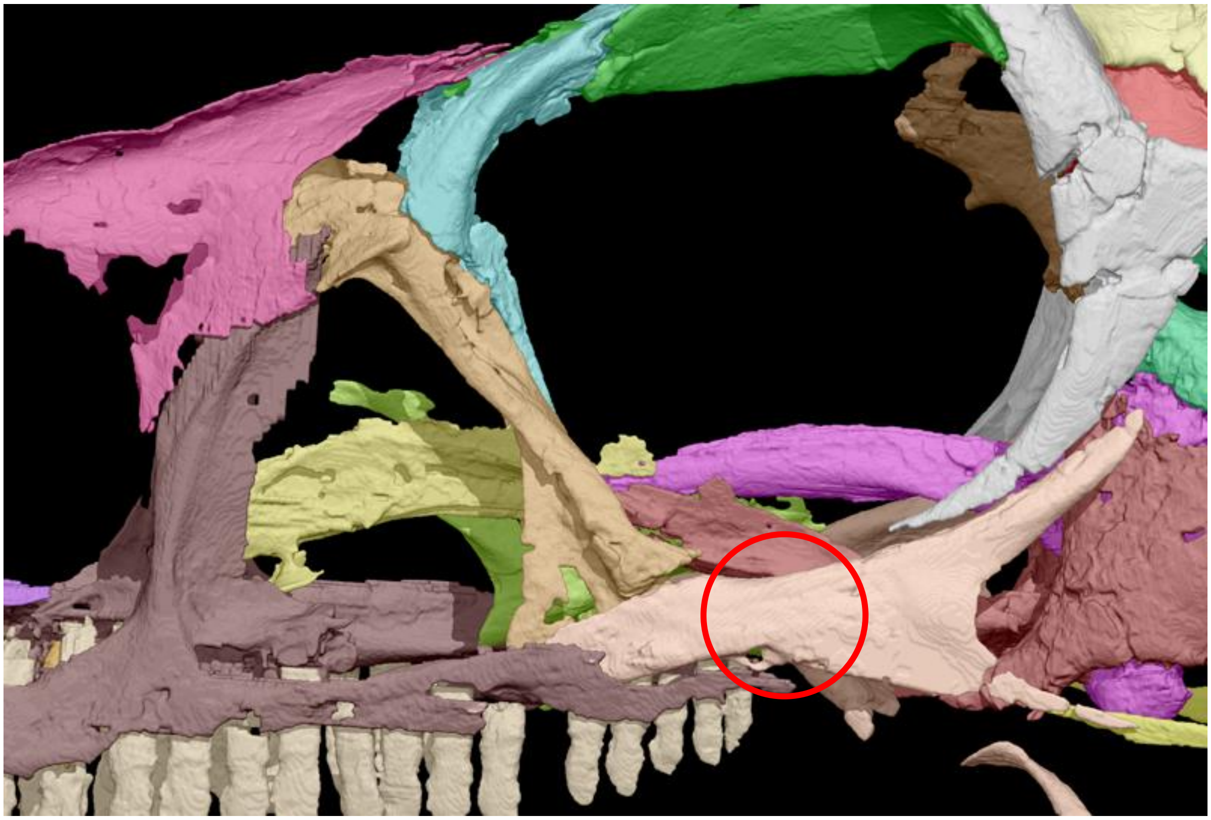

Massospondylus (0) (Image by: Kimberley Chapelle)

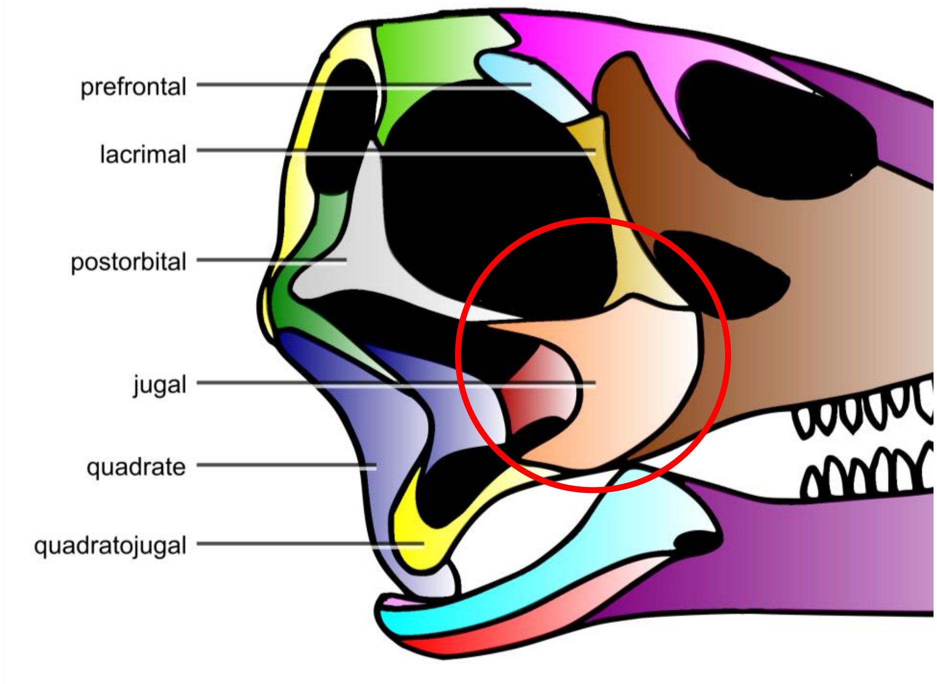

Omeisaurus (1) (Image by: Kimberley Chapelle, modified from He et al. 1988)

54. Jugal: Dorsally extending process of the anterior jugal (modified from Rauhut 2003; Yates 2007 ch. 51)

0 present

1 absent

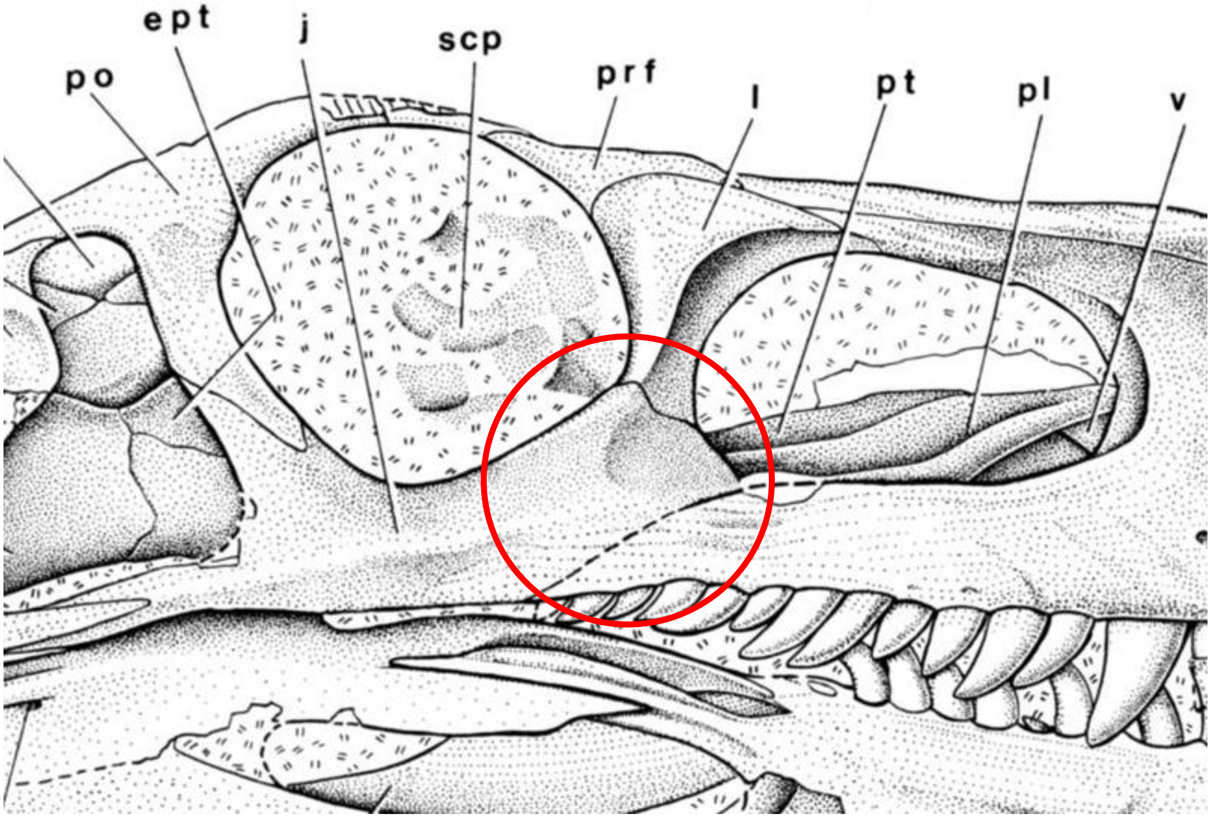

Herrerasaurus (0) (Image from: Sereno & Novas 1993; copyright © Society of Vertebrate Paleontology, www.vertpaleo.org, reprinted by permission of Taylor & Francis Ltd, http://www.tandfonline.com on behalf of Society of Vertebrate Paleontology)

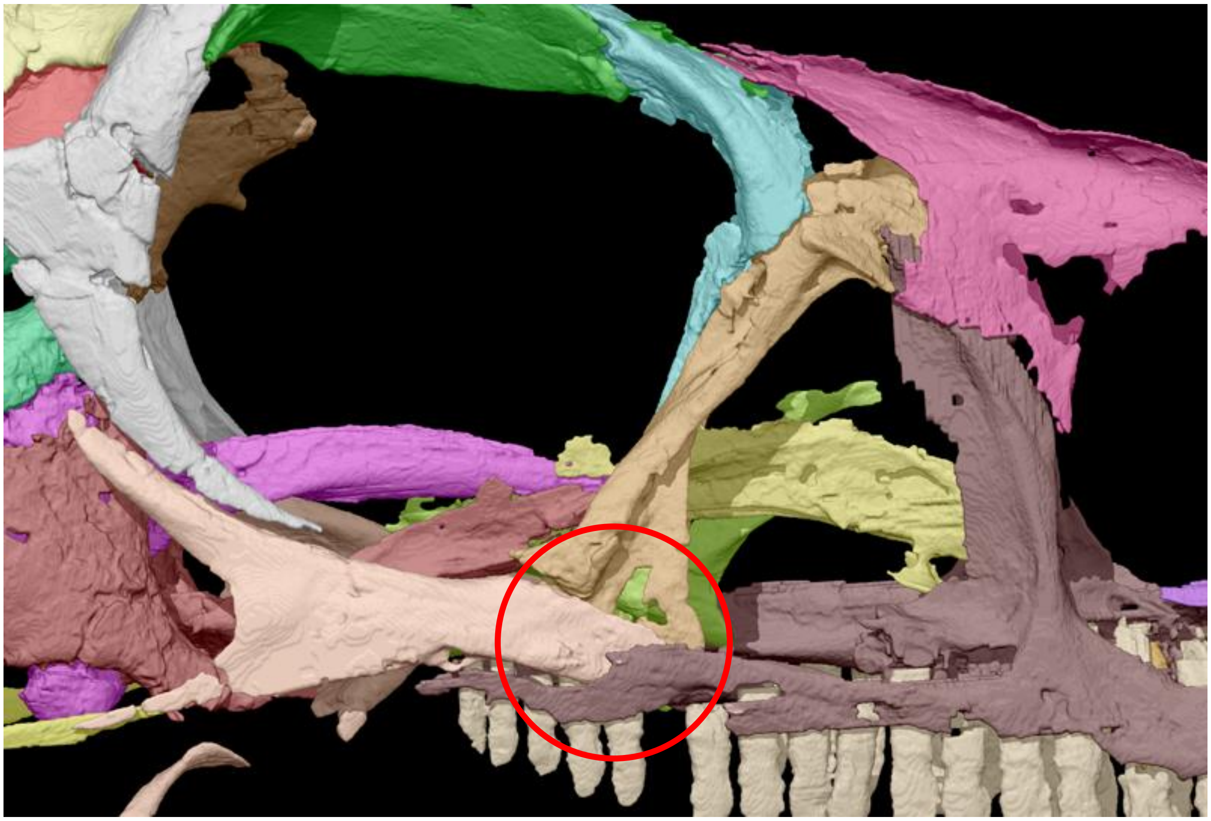

Massospondylus (1) (Image by: Kimberley Chapelle)

55. Jugal: Ratio of the minimum dorsoventral height of the jugal below the orbit to the distance between the anterior end of the jugal and the anteroventral corner of the infratemporal fenestra (modified from Galton 1985a; Yates 2007 ch. 52)

0 less than 0.2
1 greater than 0.2

56. Postorbital: Mediolateral width of the jugal ramus(ventral ramus) of the postorbital (Wilson and Sereno 1998; Yates 2007 ch. 53)

0 less than its anteroposterior width at midshaft
1 greater than its anteroposterior width at midshaft

57. Postorbital: Shape of the dorsal margin in lateral view (Yates 2007 - ch. 54)

0 straight to gently curved
1 with a distinct embayment between the frontal and squamosal rami (anterior and posterior dorsal processes)

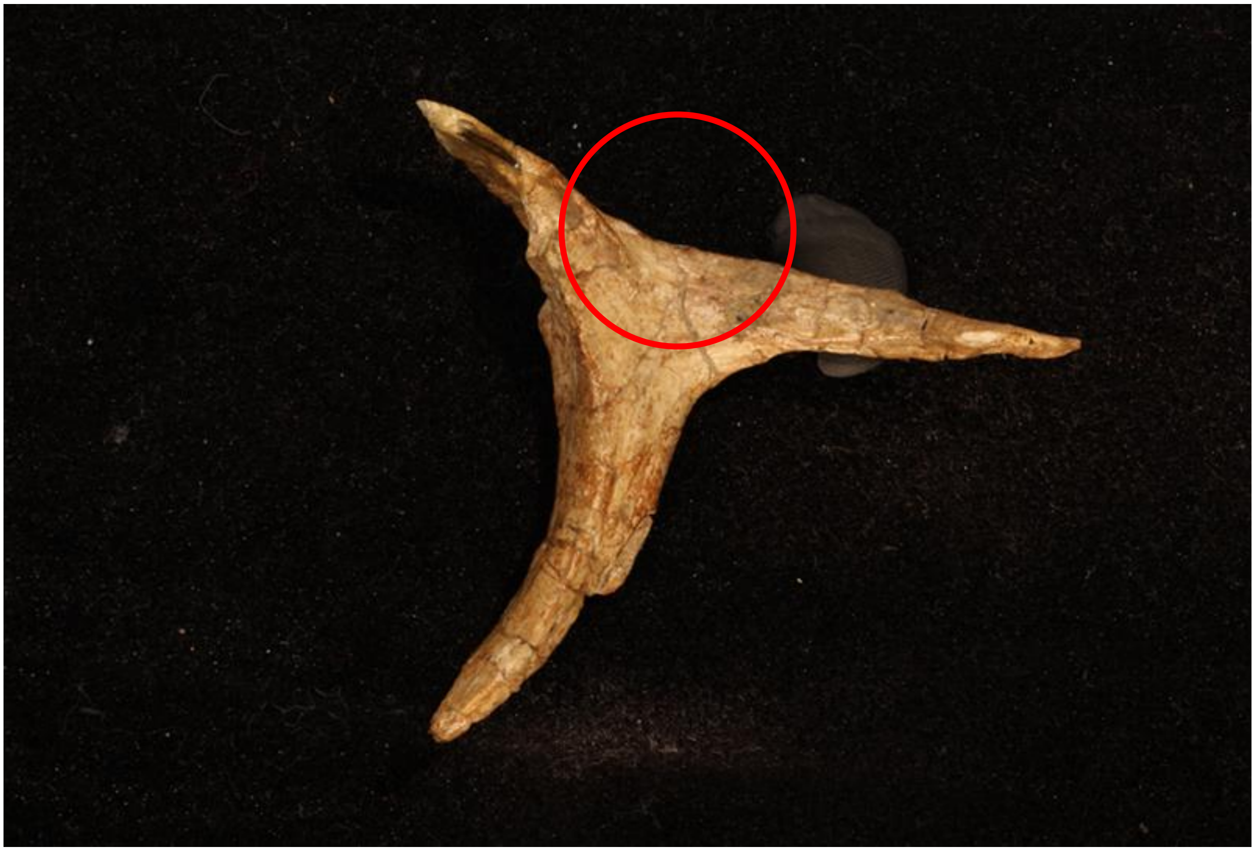

Plateosaurus erlenbergiensis (0) (Image by: Kimberley Chapelle)

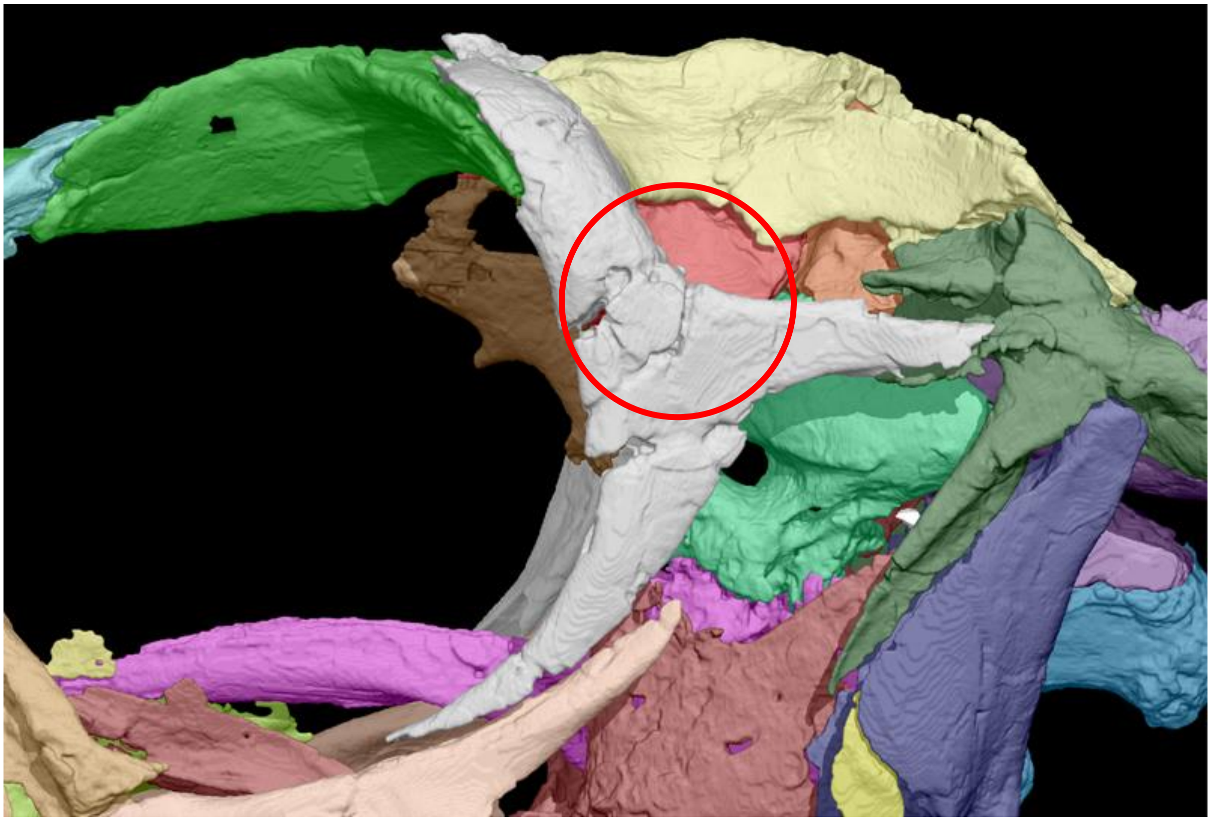
Massospondylus (1) (Image by: Kimberley Chapelle)

58. Postorbital: Distal end of frontal ramus, distinct concave notch between parietal and frontal facets (New character). In some taxa, such as *Sarahsaurus*, the distal end of the frontal ramus of the postorbital is forked into a posterior and anterior process. The posterior process contacts the parietal and the anterior process contacts the frontal. This is not the case in *Massospondylus*.

0 absent

1 present

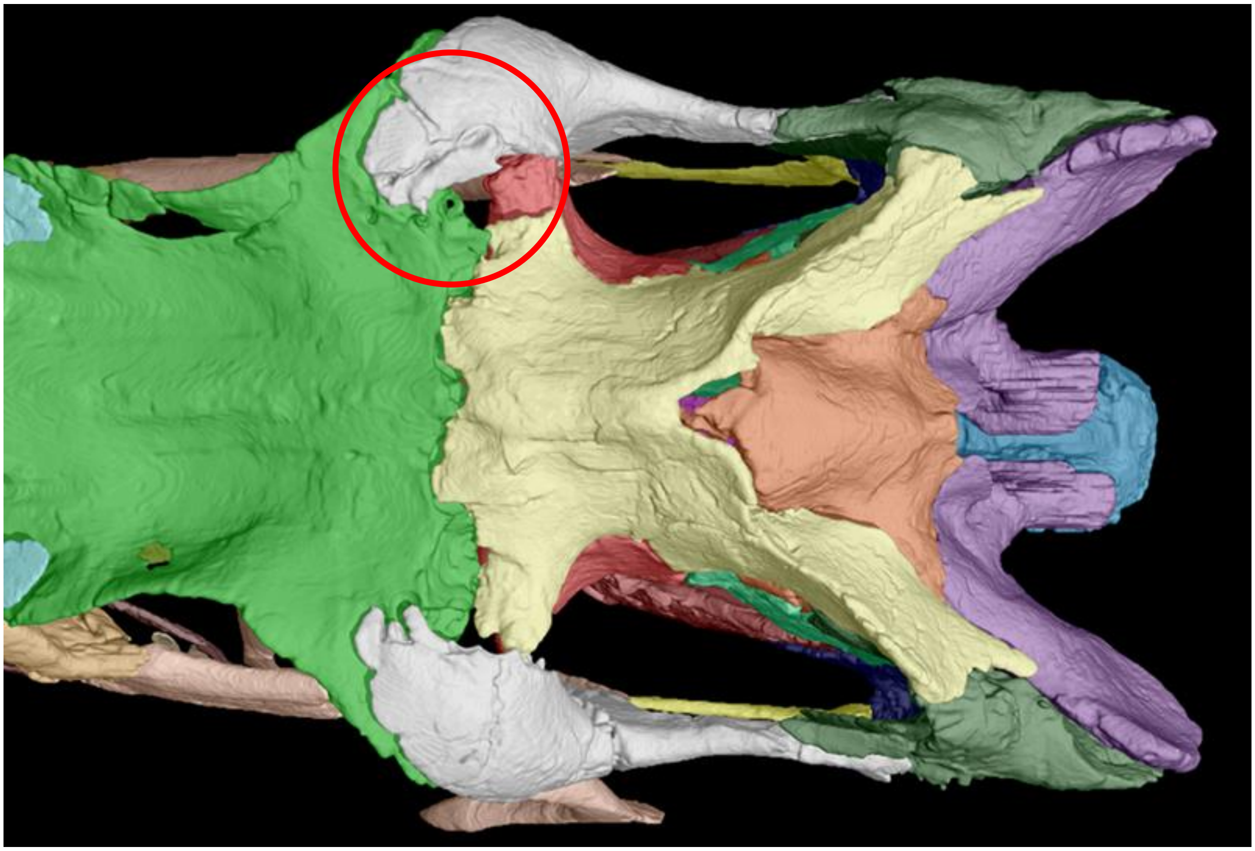

Massospondylus (0) (Image by: Kimberley Chapelle)

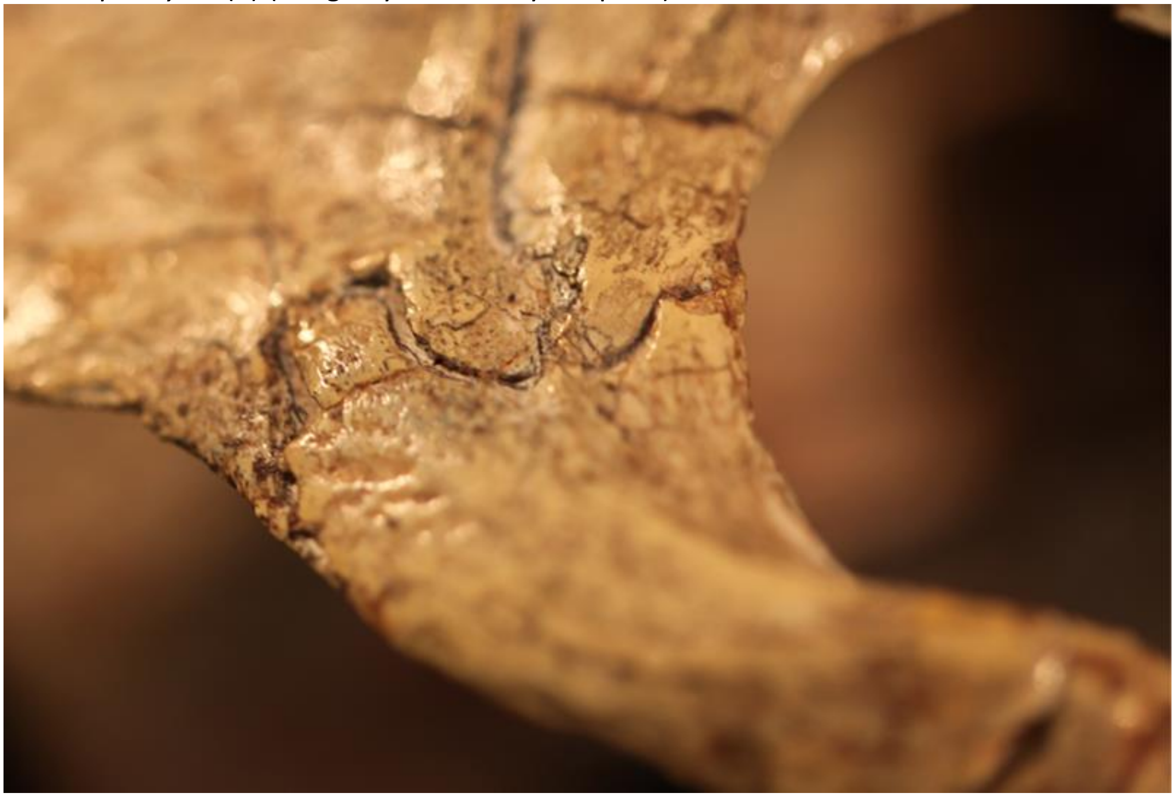

Sarahsaurus (1) (Image by: Kimberley Chapelle)

59. Postorbital rim: Relative height (Yates 2007 ch. 55)
0 flush with the squamosal ramus of the postorbital
1 raised so that it projects laterally to the squamosal ramus

60. Postfrontal bone (Sereno et al. 1993; Yates 2007 ch. 56)
0 present
1 absent

61. Infratemporal fenestra: Position of the anterior margin (modified from Upchurch 1995. Ordered, Yates 2007 ch. 57)
0 behind the orbit
1 extends under the rear half of the orbit
2 extends as far forward as the midlength of the orbit

62. Frontal: Frontal contribution to the supratemporal fenestra (modified from Gauthier 1986; Yates 2007 ch. 58) 63
0 present
1 absent

63. Frontal: Presence of anterior portion of supratemporal fossa on posterior end of dorsal surface of frontal. (New character)
In some taxa, such as *Sarahsaurus*, the presence of the supratemporal fossa on the posterodorsal surface of the frontal is only weakly excavated whereas in other taxa, e.g., *Plateosaurus engelhardti,* this fossa is deeply excavated and forms a sharp, scarp-like ridge along its anterior margin.
0 weak
1 deeply excavated, forming a scarp- like margin

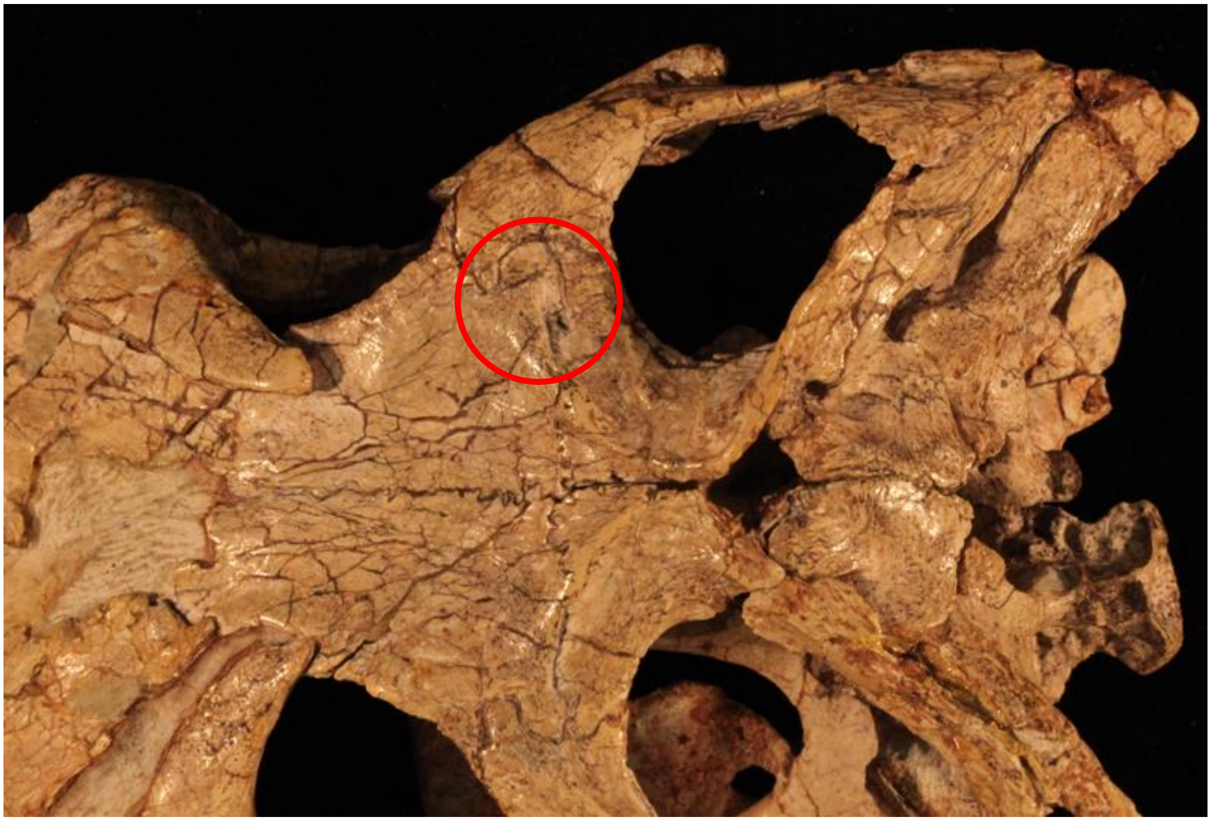
Sarahsaurus (0) (Image by: Kimberley Chapelle)
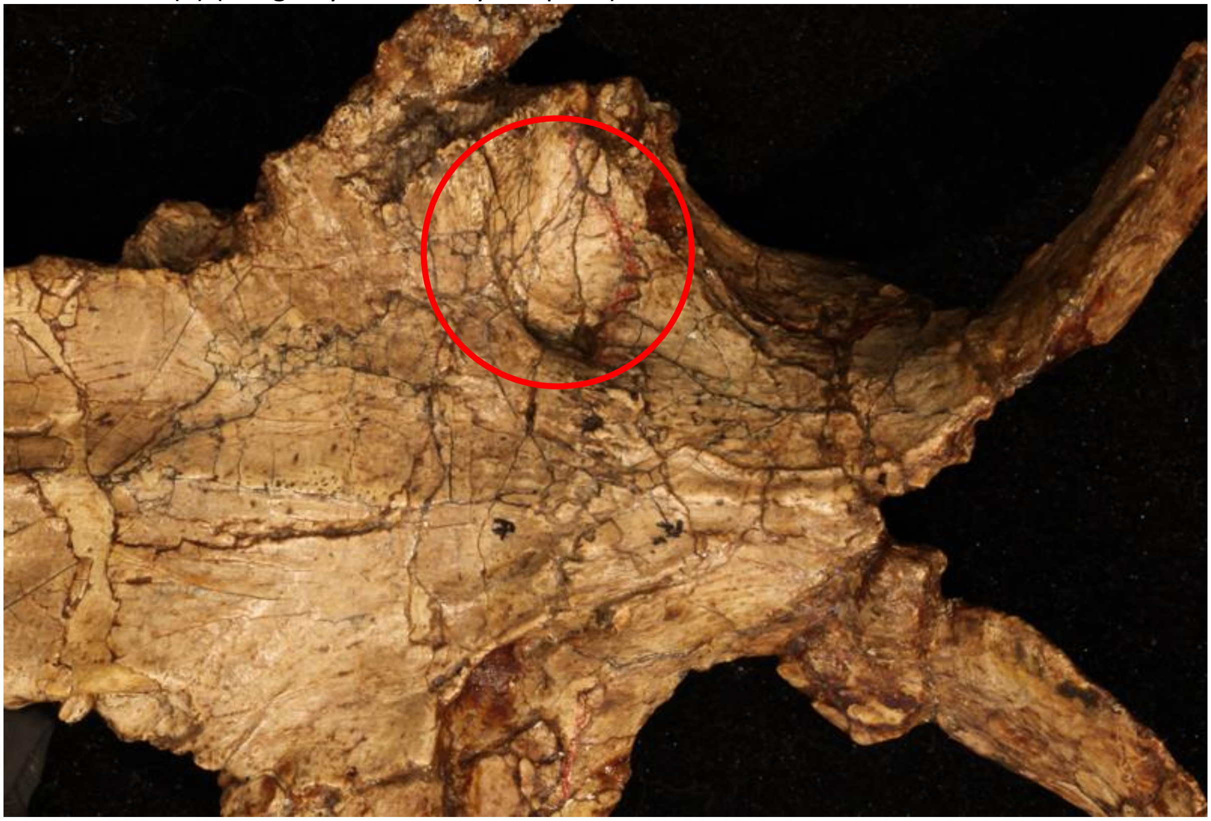
Plateosaurus engelhardti (1) (Image by: Kimberley Chapelle)

64. Supratemporal fenestra: Orientation of the long axis (Wilson and Sereno 1998; Yates 2007 ch. 59)

0 longitudinal

1 transverse

65. Squamosal: Dorsoventral height of the quadratojugal ramus relative to the anteroposterior length at its base (Sereno 1999; Yates 2007 ch. 61)

0 less than four times its width

1 greater than four times its width

66. Squamosal: Proportion of infratemporal fenestra bordered by squamosal (quadratojugal ramus) (Yates 2007 ch. 62)

0 more than 0.5 of the depth of the infratemporal fenestra

1 less than 0.5 of the depth of the infratemporal fenestra

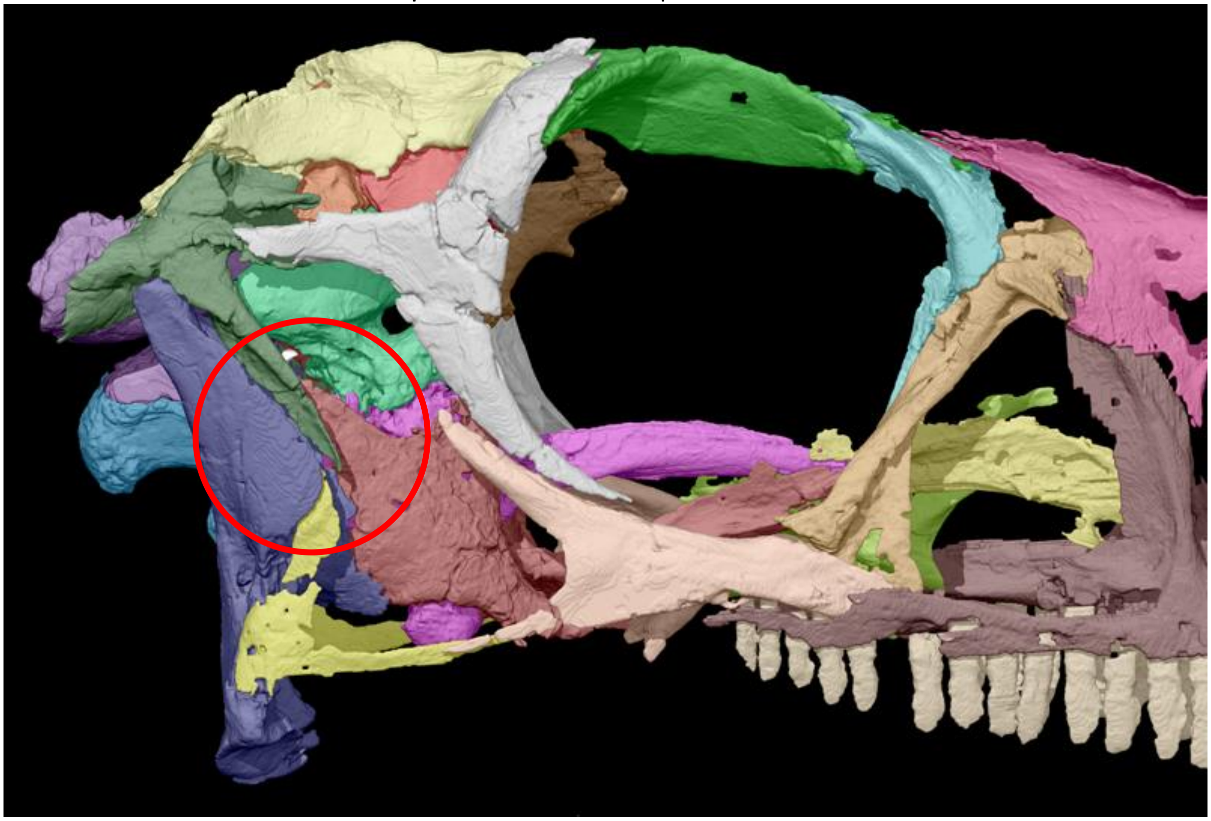

Massospondylus (0) (Image by: Kimberley Chapelle)

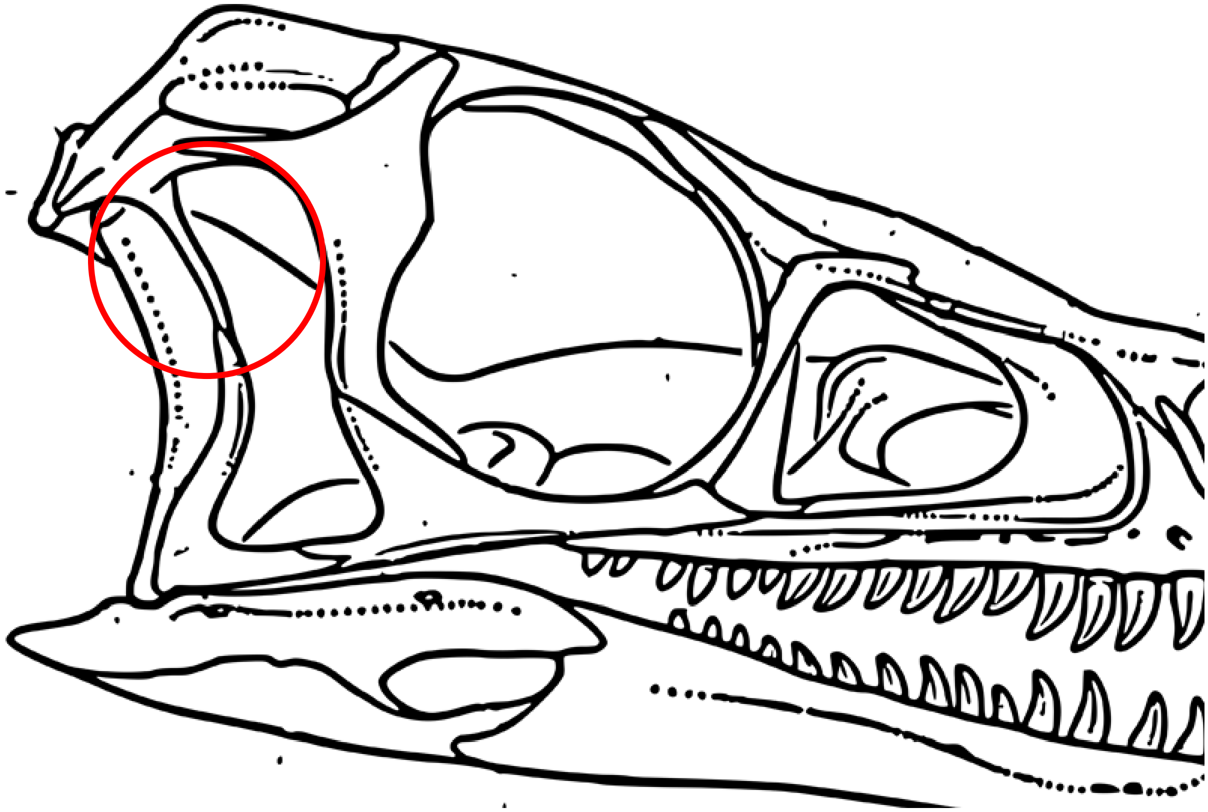

Eoraptor (1) (Image from: Sereno et al. 2012; copyright © Society of Vertebrate Paleontology, www.vertpaleo.org, reprinted by permission of Taylor & Francis Ltd, http://www.tandfonline.com on behalf of Society of Vertebrate Paleontology)

67. Squamosal: Squamosal-quadratojugal contact (Gauthier 1986; Yates 2007 ch. 63)
   0 present
   1 absent

68. Squamosal: Nature of squamosal-quadratojugal contact (New character). In some taxa, such as *Massospondylus carinatus*, the squamosal-quadratojugal contact is a point contact or a dorsoventrally oriented, narrow contact between the lateral surface of the distal end of the quadratojugal ramus of the squamosal and the medial surface of the distal end of the squamosal ramus of the quadratojugal. In other taxa, such as *Mamenchisaurus* and neosauropods, this contact is anteroposteriorly oriented and occurs between the ventral surface of the distal end of the quadratojugal ramus of the squamosal and the dorsal surface of the distal end of the squamosal ramus of the quadratojugal.
   0 point/narrow contact, dorsoventrally oriented
   1 broad contact, anteroposteriorly oriented

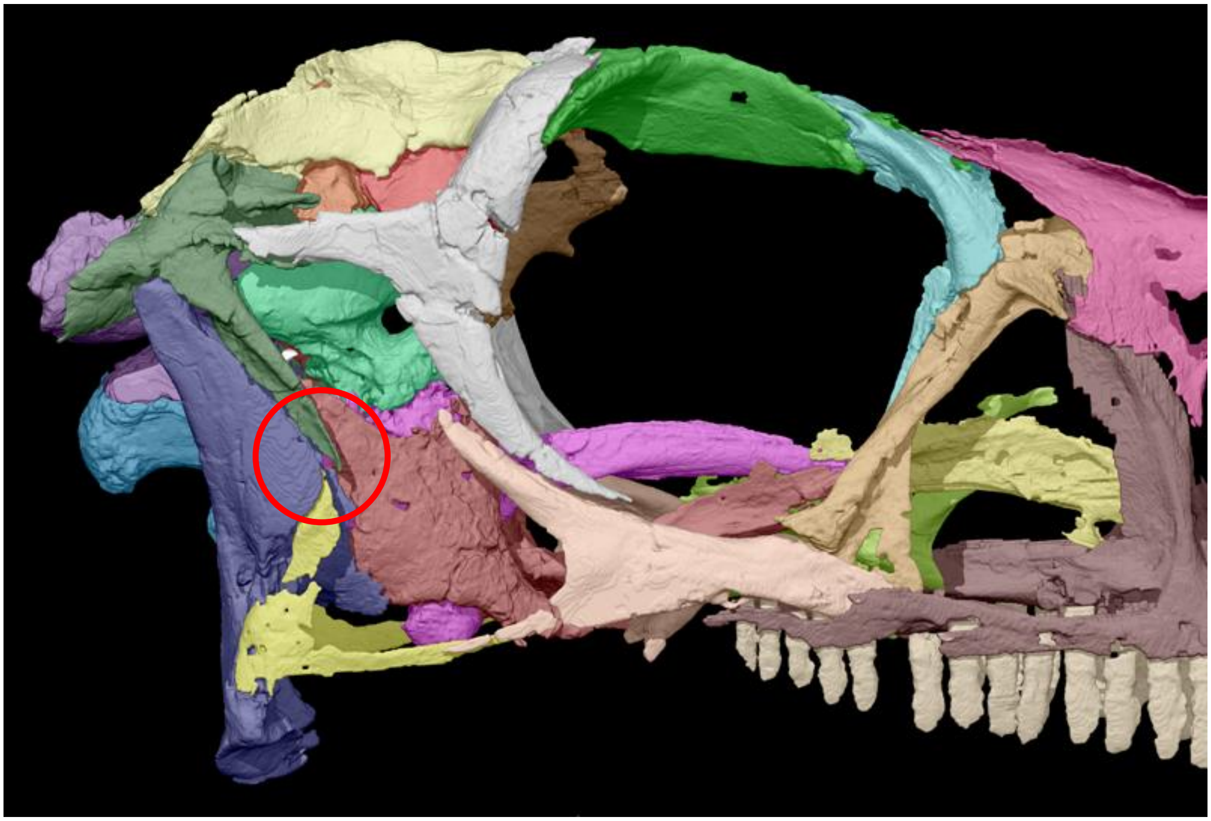

Massospondylus (0) (Image by: Kimberley Chapelle)

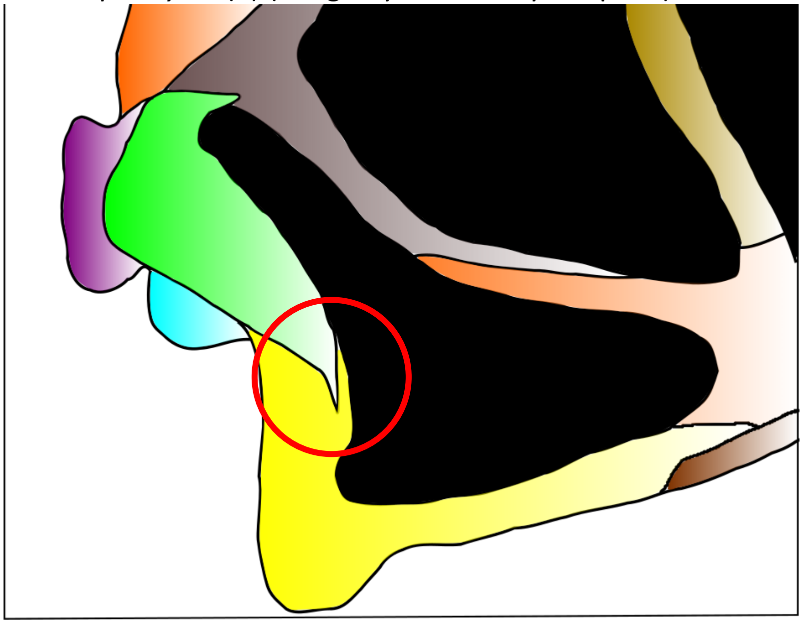

Mamenchisaurus (1) (Image by: Kimberley Chapelle, modified from Ouyang & Ye 2001)

69. Quadratojugal: Angle of divergence between jugal and squamosal rami of quadratojugal in lateral view (Yates 2007 ch. 64)

0 90 degrees or more

1 near parallel (much less than 90 degrees)

70. Quadratojugal: Length of jugal ramus (entire ventral margin, to quadratojugal angle) (Wilson and Sereno 1998; Yates 2007 ch. 65)

0 no longer than the squamosal ramus

1 longer than the squamosal ramus

71. Quadratojugal: Shape of the anterior end of the jugal ramus (Wilson and Sereno 1998; Yates 2007 ch. 66)

0 tapered

1 dorsoventrally expanded

72. Quadratojugal: Relationship of quadratojugal to jugal (Yates 2007 ch. 67. Unordered.)

0 jugal overlaps the lateral surface of the quadratojugal

1 quadratojugal overlaps the lateral surface of the jugal

2 quadratojugal sutures along the ventrolateral margin of the jugal

73. Quadrate foramen: Position of the quadrate foramen (modified from Rauhut 2003. Unordered, Yates 2007 ch. 68)

0 on the quadrate-quadratojugal suture

1 deeply incised into, and partly encircled by, the quadrate

2 on the quadrate-squamosal suture, just below the quadrate head

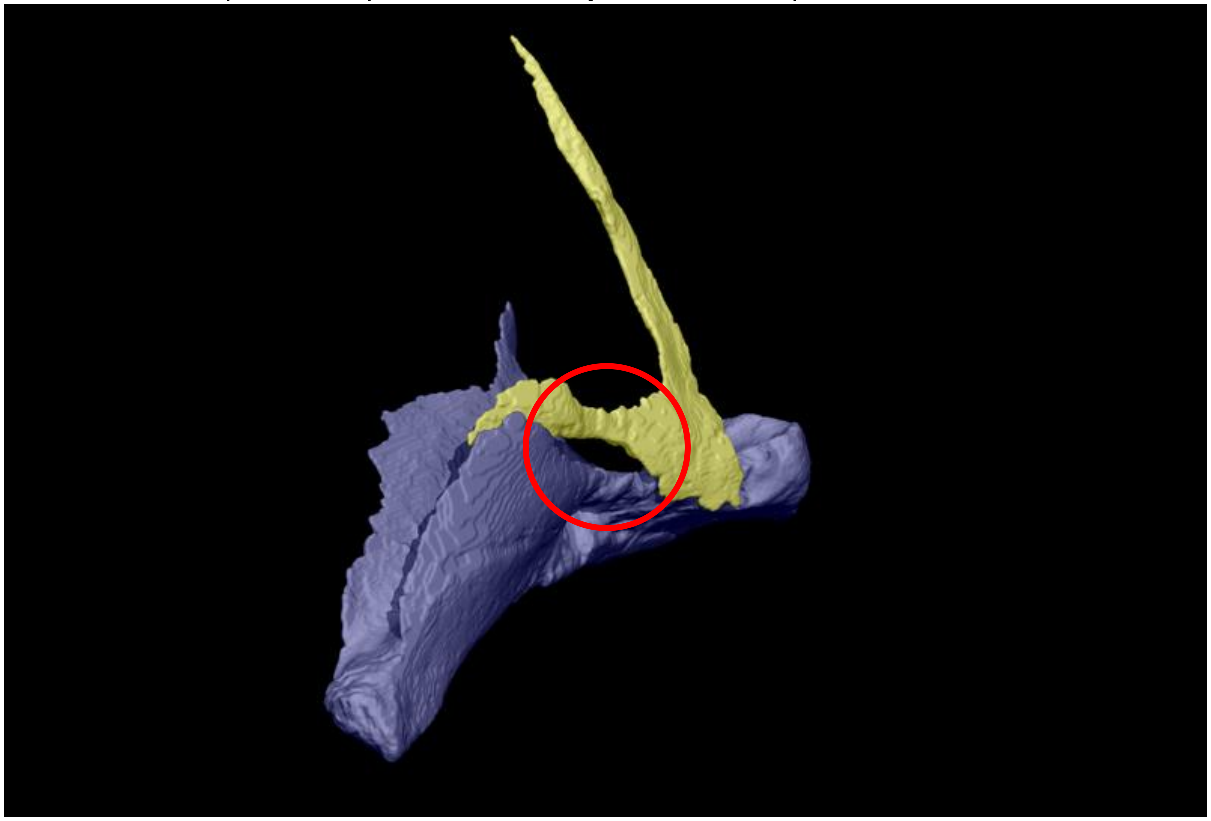

Massospondylus (0) (Image by: Kimberley Chapelle)

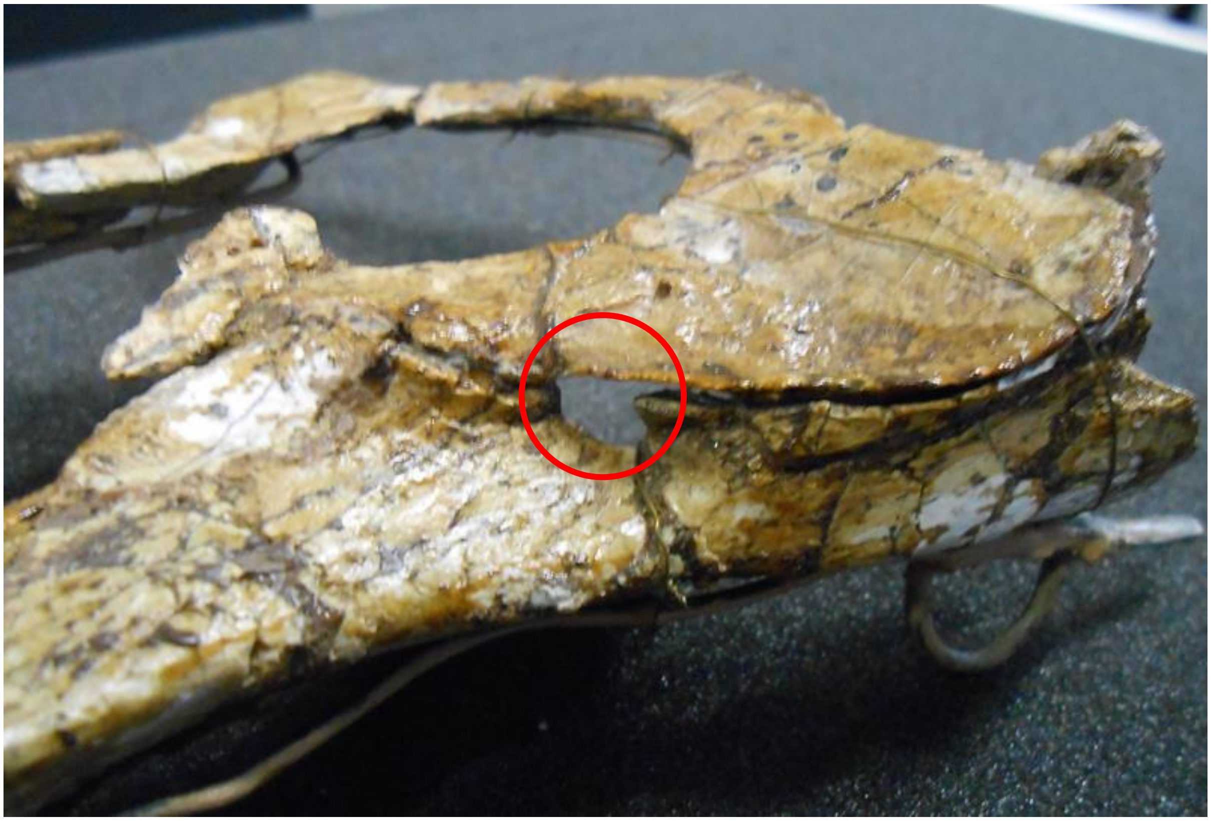
Plateosaurus engelhardti (1) (Image by: Kimberley Chapelle)

74. Shape of posterolateral margin of quadrate (Wilson and Sereno 1998. Ordered, Yates 2007 ch. 69)

0 sloping anterolaterally from posteromedial ridge
1 everted posteriorly creating a posteriorly facing fossa
2 posterior fossa deeply excavated, invading quadrate body

75. Quadrate: Exposure of the lateral surface of the quadrate head in lateral view in specimens with articulated skull bones (Sereno et al. 1993; Yates 2007 ch. 70)

0 absent, covered by lateral sheet of the squamosal
1 present

76. Quadrate: Proportion of the dorsoventral height of the quadrate that is occupied by the pterygoid wing (Yates 2003b; Yates 2007 ch. 71)

0 less than 70 per cent
1 greater than 70 per cent

77. Quadrate: Angle between quadratojugal and pterygoid rami (Ordered, New character). In some taxa such as *Massospondylus carinatus*, the angle between the medial surface of the quadratojugal wing and the lateral surface of the pterygoid wing forms an acute angle, less than or equal to 90 degrees. In other taxa, such as *Efraasia*, this angle is obtuse.

0 between 0 and 30 degrees, near parallel
1 between 30 degrees and 90 degrees

2 greater than 90 degrees

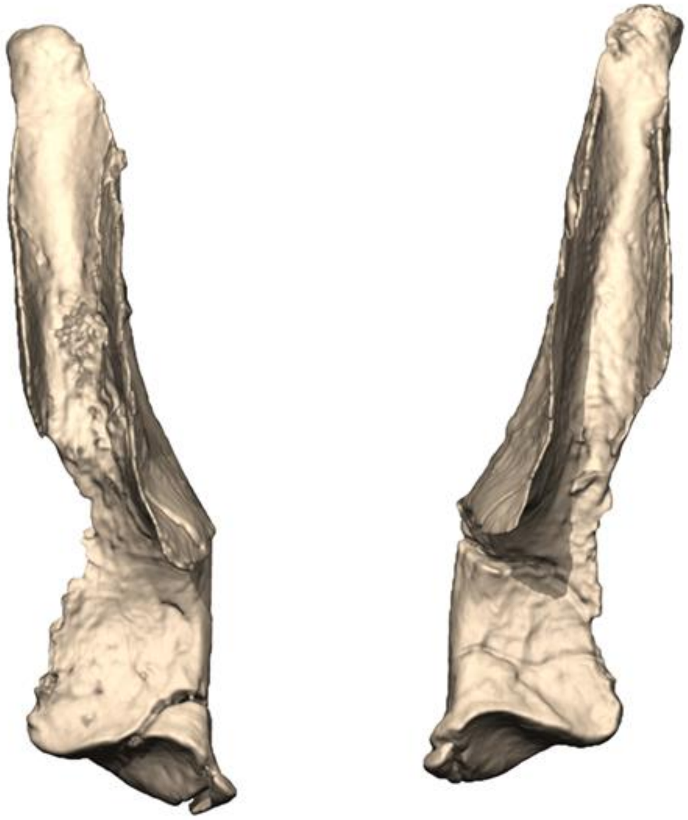

Massospondylus (0) (Image by: Kimberley Chapelle)

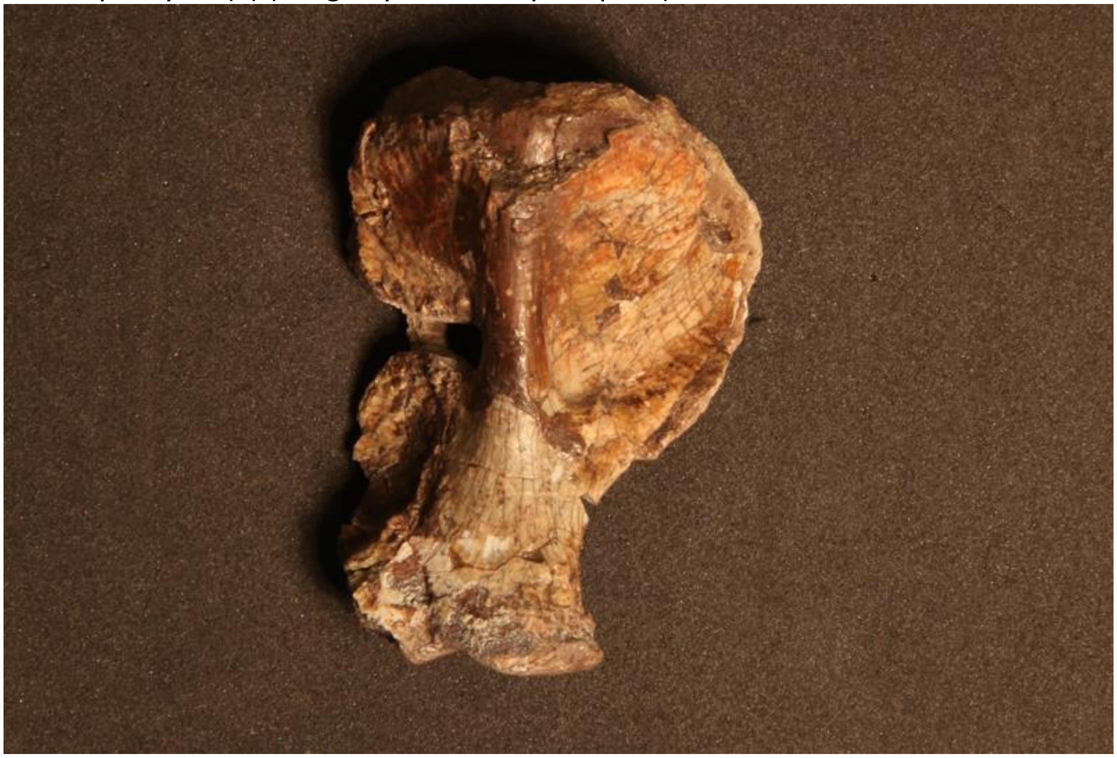

Efraasia (2) (Image by: Kimberley Chapelle)

78. Quadrate: Ventral extent of condyles (New character). In some taxa, such as *Massospondylus*, the medial condyle of the quadrate extends farther ventrally than the lateral condyle. In other taxa, such as *lateosaurus longiceps*, the opposite is true.

0 both condyles extend to the same ventral level
1 medial condyle extends farther ventrally
2 lateral condyle extends farther ventrally

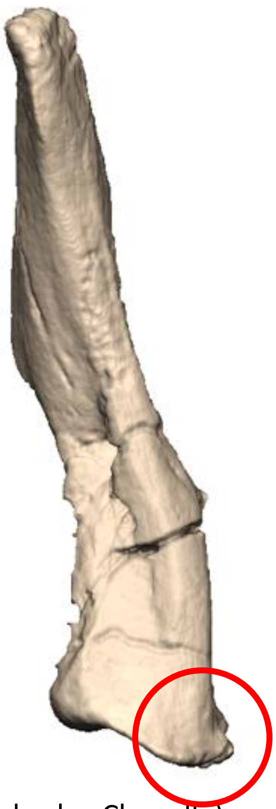

Massospondylus (1) (Image by: Kimberley Chapelle)

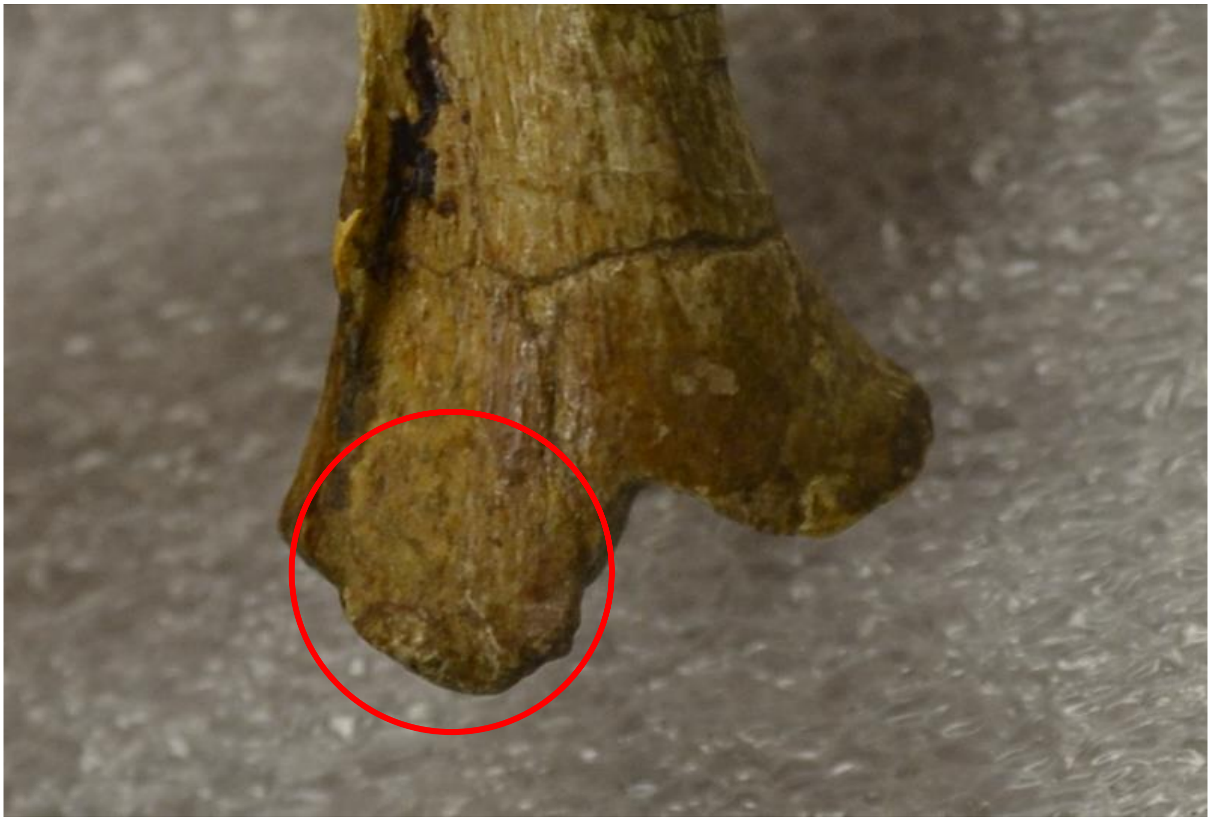

Plateosaurus longiceps (2) (Image by: Kimberley Chapelle)

79. Parietal: Dorsoventral height of the posterior wing of the parietal (Wilson and Sereno 1998; Yates 2007 ch. 72)
    0 less than 1.5 times the depth of the foramen magnum
    1 more than 1.5 times the depth of the foramen magnum

80. Supraoccipital Position of foramina for mid-cerebral vein on occiput (modified from Yates 2003b; Yates 2007 ch. 73)
    0 between supraoccipital and parietal
    1 on the supraoccipital

81. Postparietal fenestra: an opening along the skull midline between the anterior side of the dorsal end of the supraoccipital and the posterior surface of the parietals (Yates 2007 ch. 74)
    0 absent
    1 present

82. Supraoccipital: Shape of the supraoccipital in posterior view (Yates 2003c; Yates 2007 ch. 75)
    0 diamond-shaped, at least as high as wide
    1 semilunate and wider than high

83. Supraoccipital: Orientation of the anteroposterior axis of the dorsal surface of the supraoccipital in lateral view (Galton and Upchurch 2004; Yates 2007 ch. 76)

0 erect to gently sloping

1 strongly sloping forward so that the dorsal tip lies level with the basipterygoid processes

84. Exoccipital: Orientation of the long axis of the paroccipital processes in posterior view (Rauhut 2003; Yates 2007 ch. 77)

0 dorsolateral to horizontal

1 ventrolateral

85. Exoccipital: Orientation of the long axis of the paroccipital processes in dorsal view (Wilson 2002; Yates 2007 ch. 78)

0 posterolateral forming a V-shaped occiput

1 lateral forming a flat occiput

86. Post-temporal fenestra: Size of the post-temporal fenestra (Rauhut 2003; Yates 2007 ch. 80)

0 large fenestra

1 a small hole that is much less than half the depth of the paroccipital process

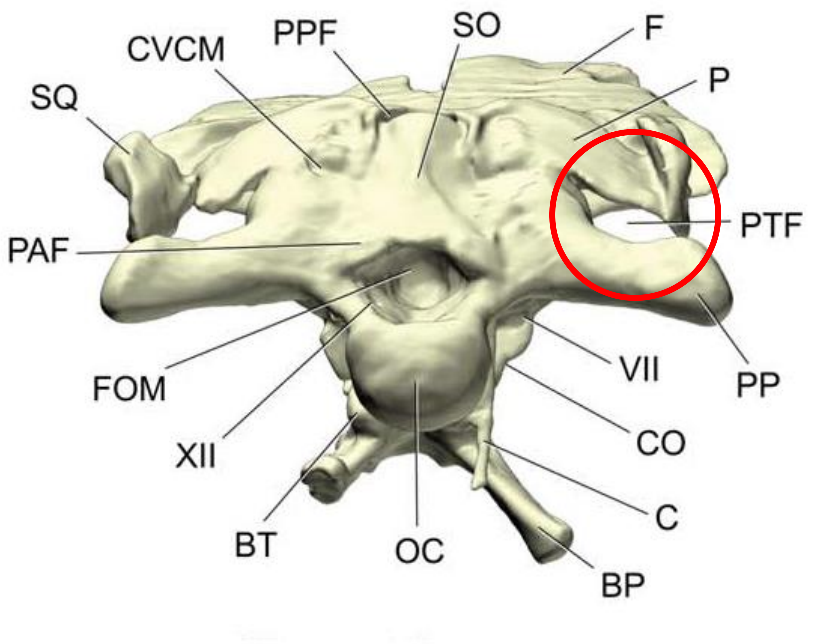

Spinophorosaurus (0) (Image from: Knoll et al. 2012)

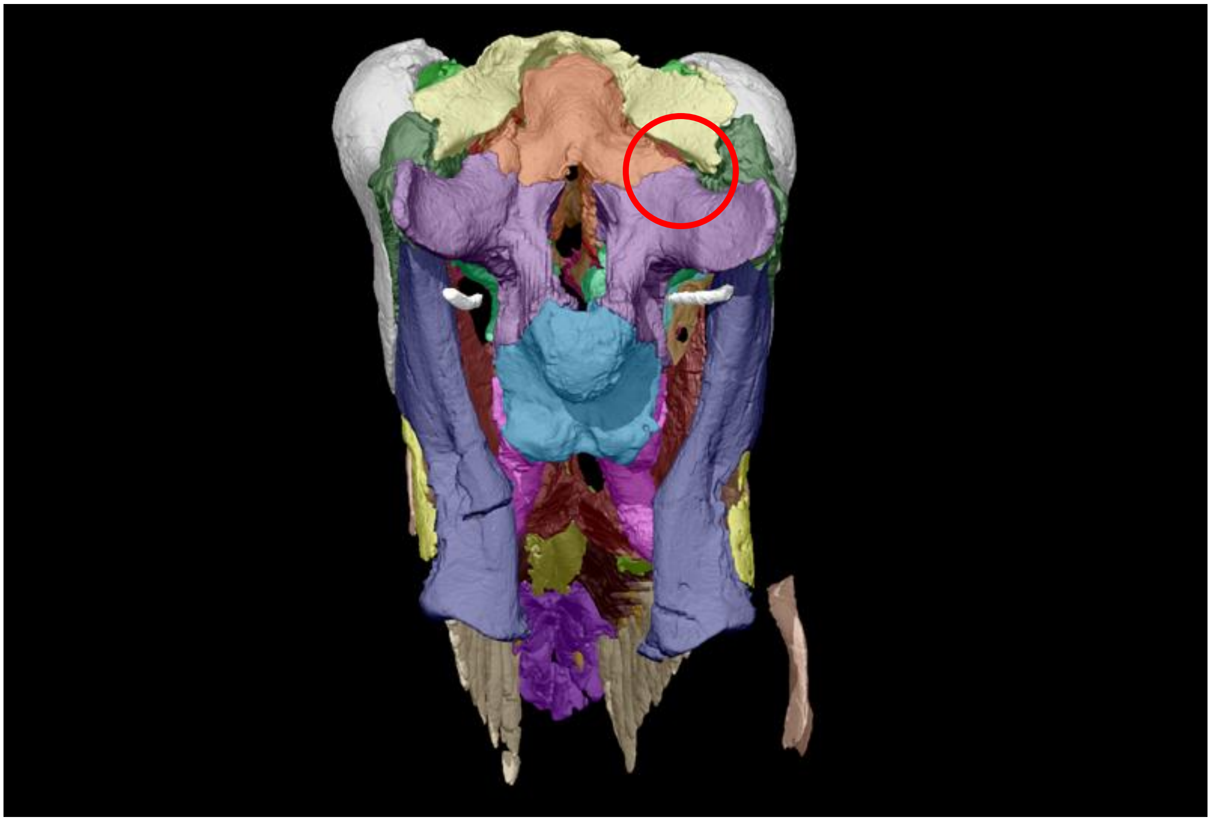
Massospondylus (1) (Image by: Kimberley Chapelle)

87. Prootic: Shape of ventral portion (New character). In some taxa, such as *Massospondylus*, the anteroventral portion of the prootic is rectangular and foot-shaped whereas in other taxa such as *Plateosaurus erlenbergiensis* and *Adeopapposaurus*, this portion is bulbous.

0 rectangular, anteroposteriorly longer than dorsoventrally high

1 bulbous, almost as anteroposteriorly long as dorsoventrally high

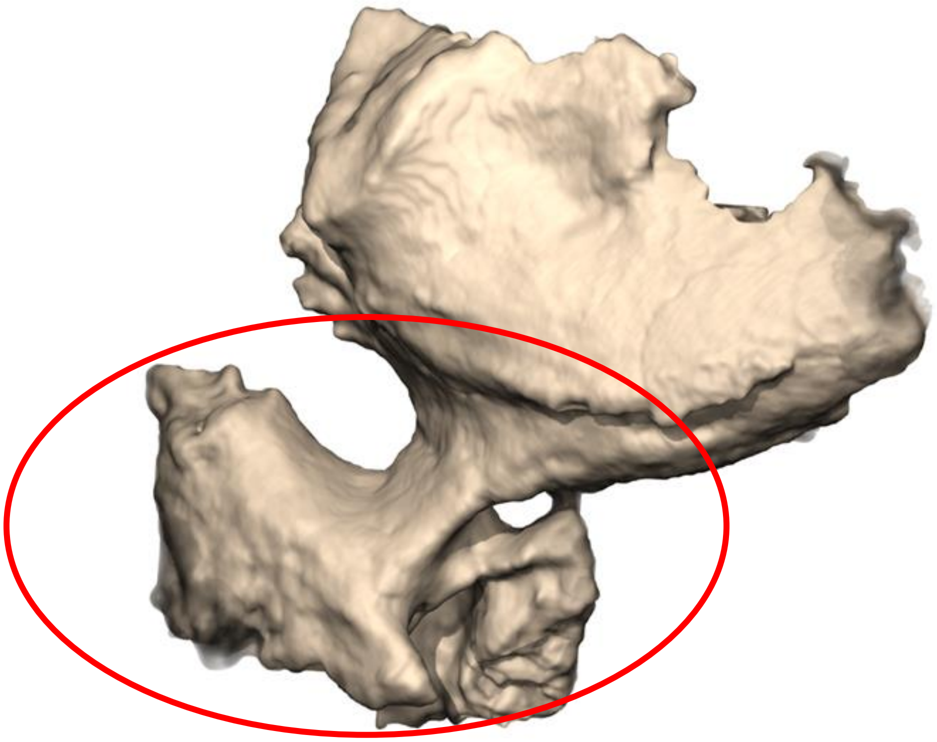

Massospondylus (0) (Image by: Kimberley Chapelle)

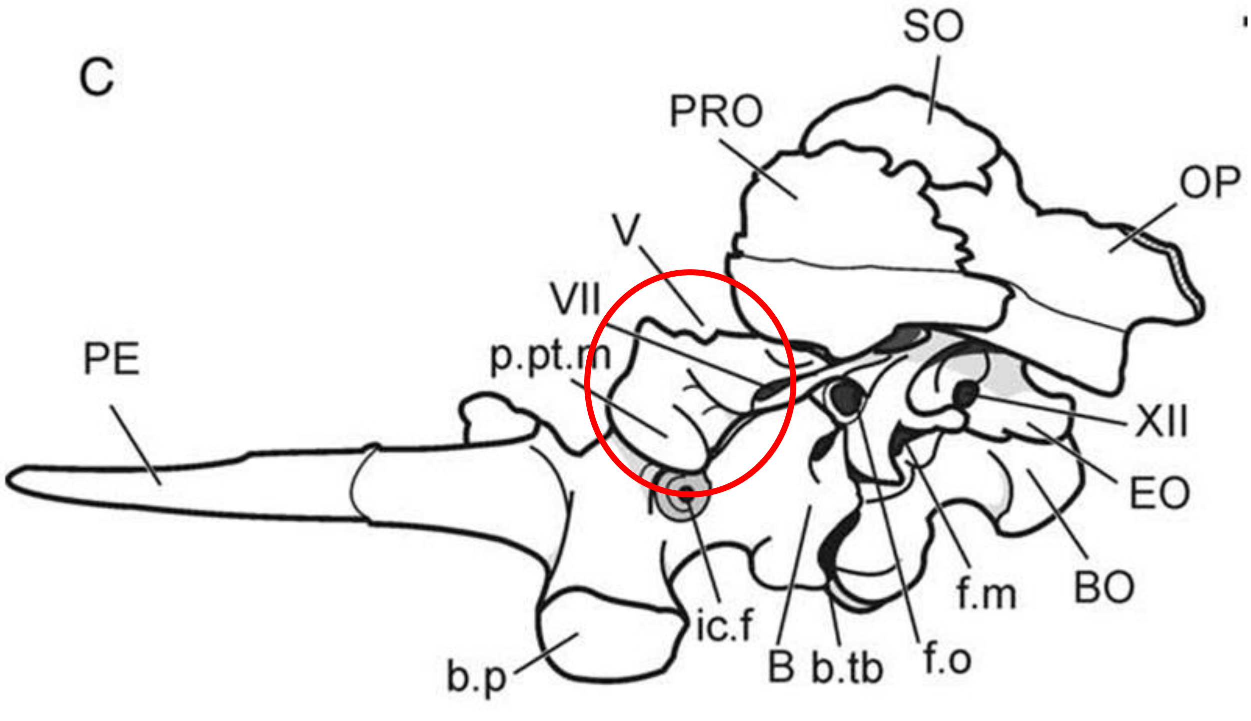

Adeopapposaurus (1) (Image from: Martinez & Alcober 2009)

88. Basal tubera: Shape in ventral view (Yates 2007 c h. 82)

0 basisphenoidal component anterior to basioccipital component

1 basisphenoidal component lateral to the basioccipital component

89. Basisphenoid: Length of the basipterygoid processes (from the top of the parasphenoid to the distal tip of the process) (Benton et al. 2000; Yates 2007 ch. 83)

0 less than the height of the braincase (from the top of the parasphenoid to the top of the supraoccipital)

1 greater than the height of the braincase (from the top of the parasphenoid to the top of the supraoccipital)

90. Basisphenoid: Angle separating the long axes of the basiperygoid processes in anterior view (New character). The angle separating the basipterygoid processes varies between taxa such as *Massospodylus* and *Sarahsaurus*.

0 60 degrees or less

1 More than 60 degrees

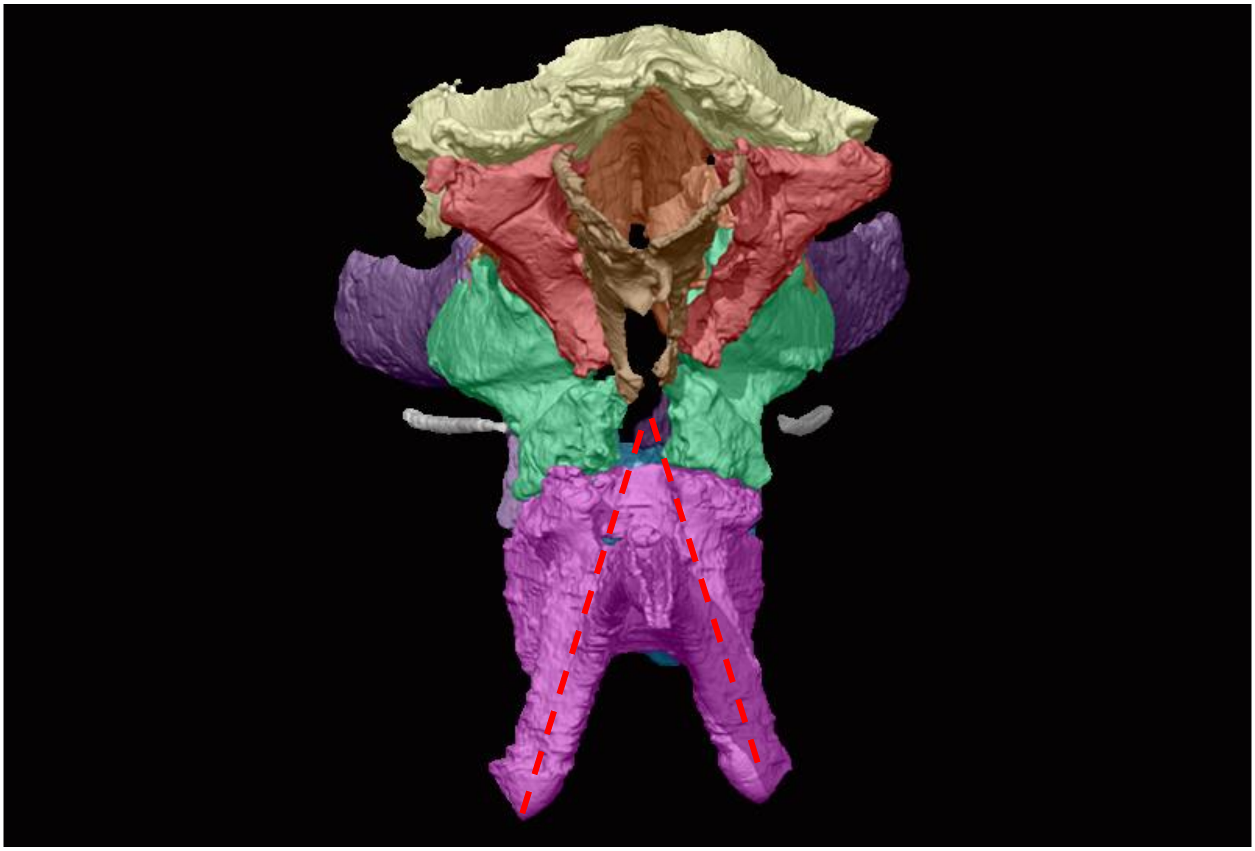

Massospondylus (0) (Image by: Kimberley Chapelle)

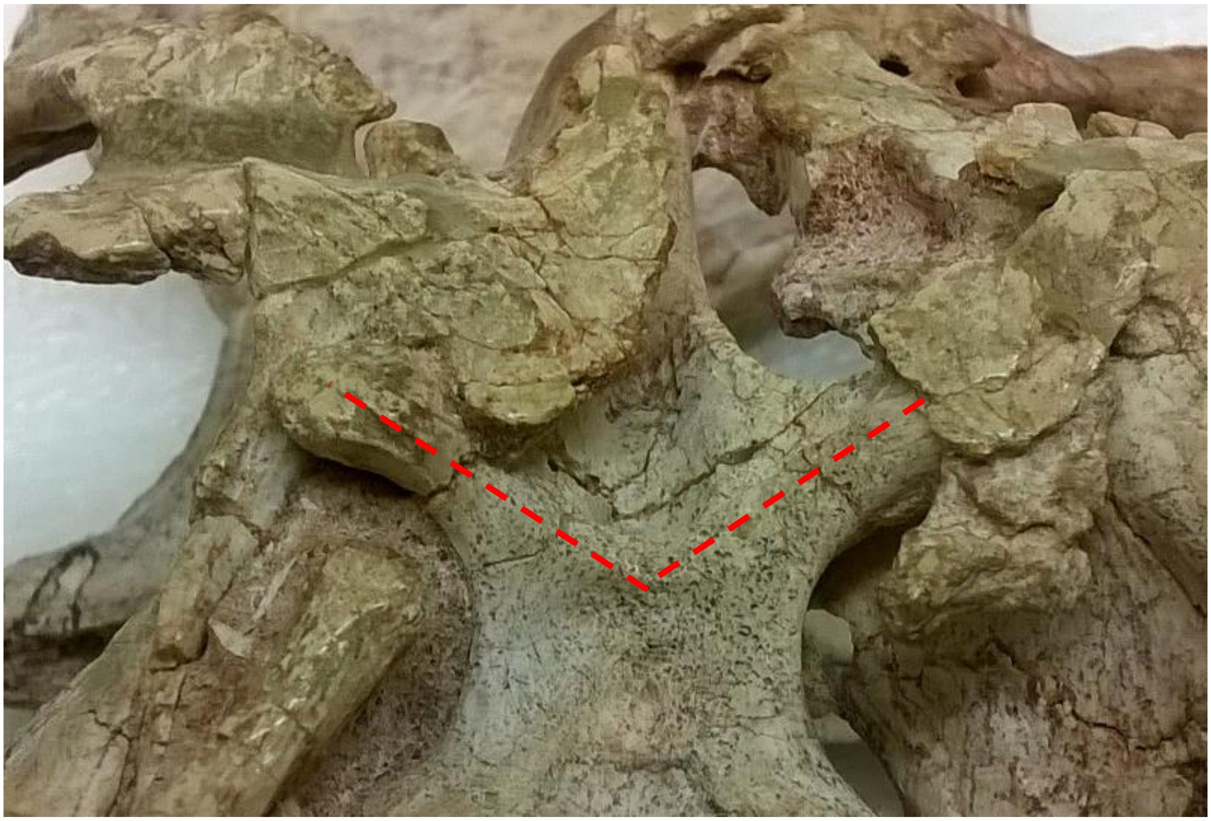
Sarahsaurus (1) (Image by: Kimberley Chapelle)

91. Basisphenoid: Ridge formed along the junction of the parabasisphenoid and the basioccipital, between the basal tubera in ventral view (New character)

0 absent with the basal tubera being separated by a deep posteriorly opening U-shaped fossa

1 Present

92. Basisphenoid: Ridge formed along the junction of the parabasisphenoid and the basioccipital, between the basal tubera in ventral view (Yates 2007 ch. 84. Unordered.)

0 present with a smooth anterior face

1 present with a median fossa on the anterior face

93. Basisphenoid: Dorsoventrally high septum spanning the interbasipterygoid space (Galton 1990; Yates 2007 ch. 85)

0 absent

1 present

94. Basisphenoid: Basisphenoid recess (New character). fossa on ventral surface, posterior to cultriform process base. In some taxa, such as *Massospondylus carinatus,* the ventral surface of the basisphenoid bears a fossa at the base of the cultriform frocess, anterior to the basipterygoid processes. This fossa tapers out as it extends anteriorly. In neosauropods, this feature is absent.

0 absent to weak
1 present and deeply excavated

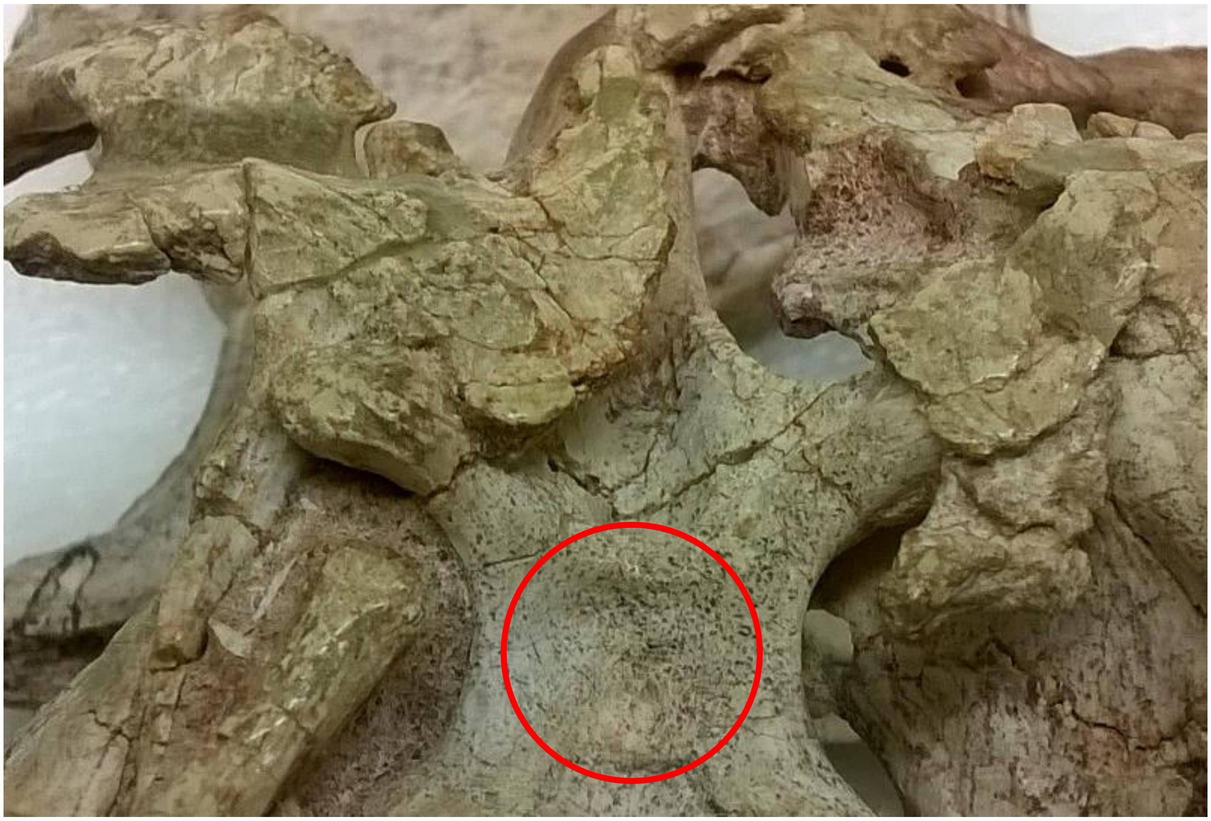

Sarahsaurus (0) (Image by: Kimberley Chapelle)

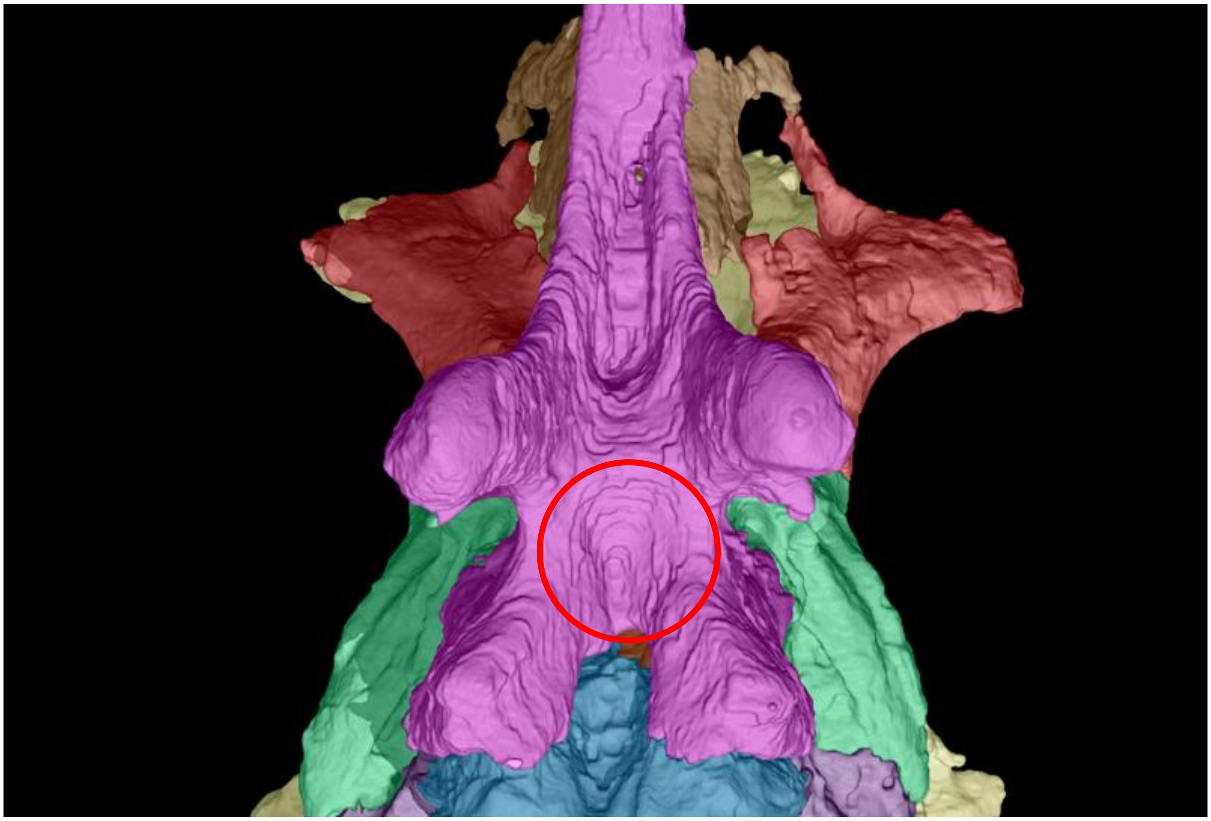

Massospondylus (1) (Image by: Kimberley Chapelle)

95. Basisphenoid: Dorsoventral height of the cultriform process (Yates 2003b; Yates 2007 ch. 86)

0 much less than the transverse width

1 about equal to the transverse width

Closer inspection of character 81 in the Yates 2007 matrix (“shape of floor of the braincase”) reveals that multiple anatomical features with potentially independent homology statements were present. To assess variation in the braincase floor morphology, we divided this character into several separate characters to ensure that all of the potential morphological combinations possible for the floor of the braincase were assessed individually.

96. Basisphenoid: orientation of basipterygoid processes long axes in lateral view (New character).

0 extend anteroventrally

1 extend ventrally or near ventrally

2 extend posteroventrally

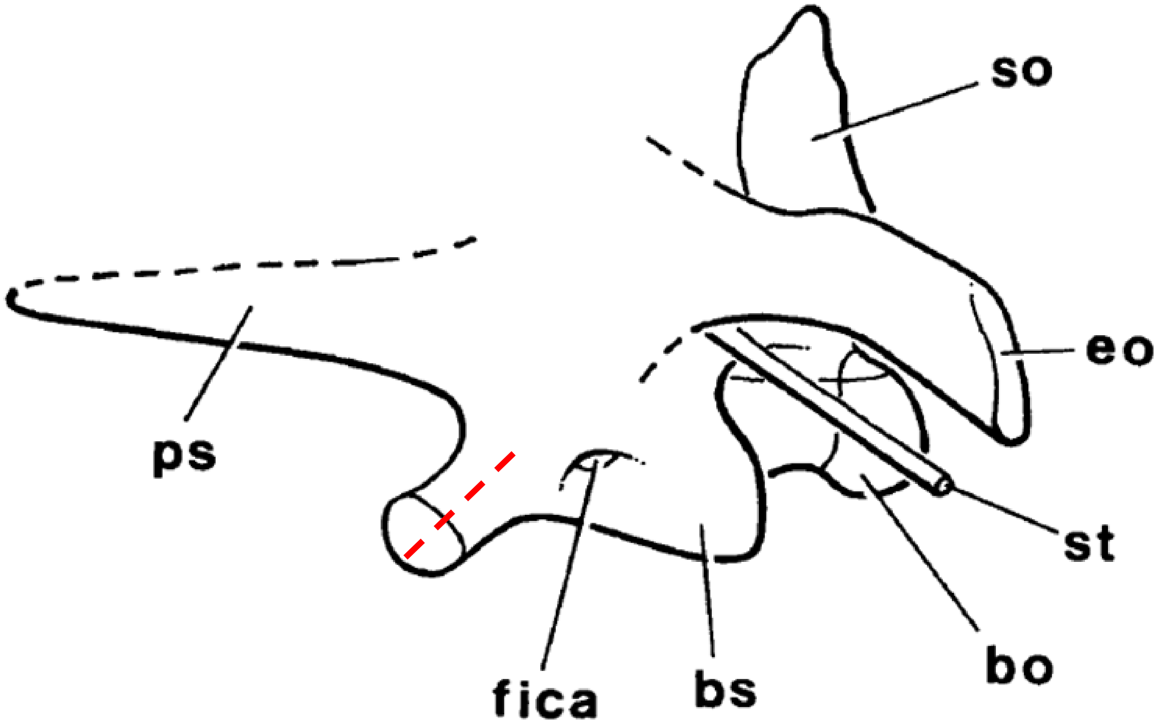

Herrerasaurus (0) (Image from: Sereno & Novas 1993; copyright © Society of Vertebrate Paleontology, www.vertpaleo.org, reprinted by permission of Taylor & Francis Ltd, http://www.tandfonline.com on behalf of Society of Vertebrate Paleontology)

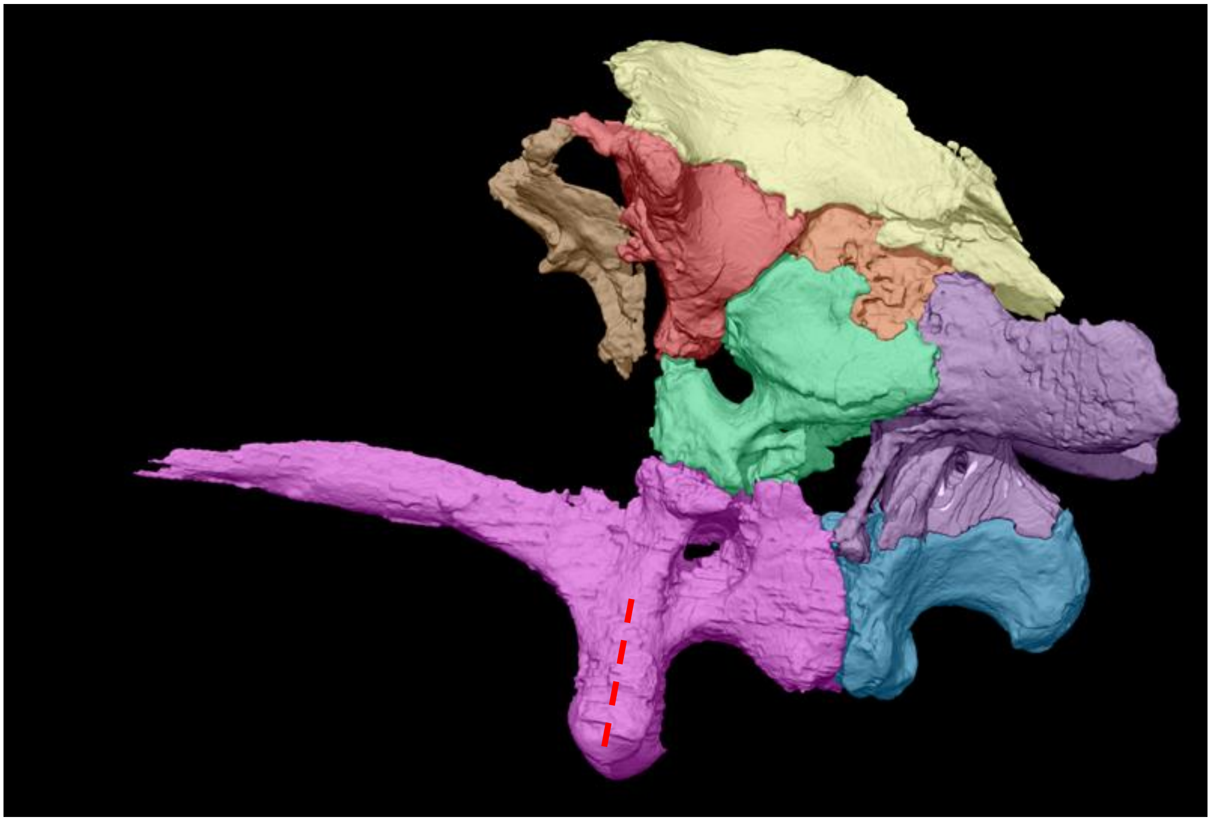

Massospondylus (1) (Image by: Kimberley Chapelle)

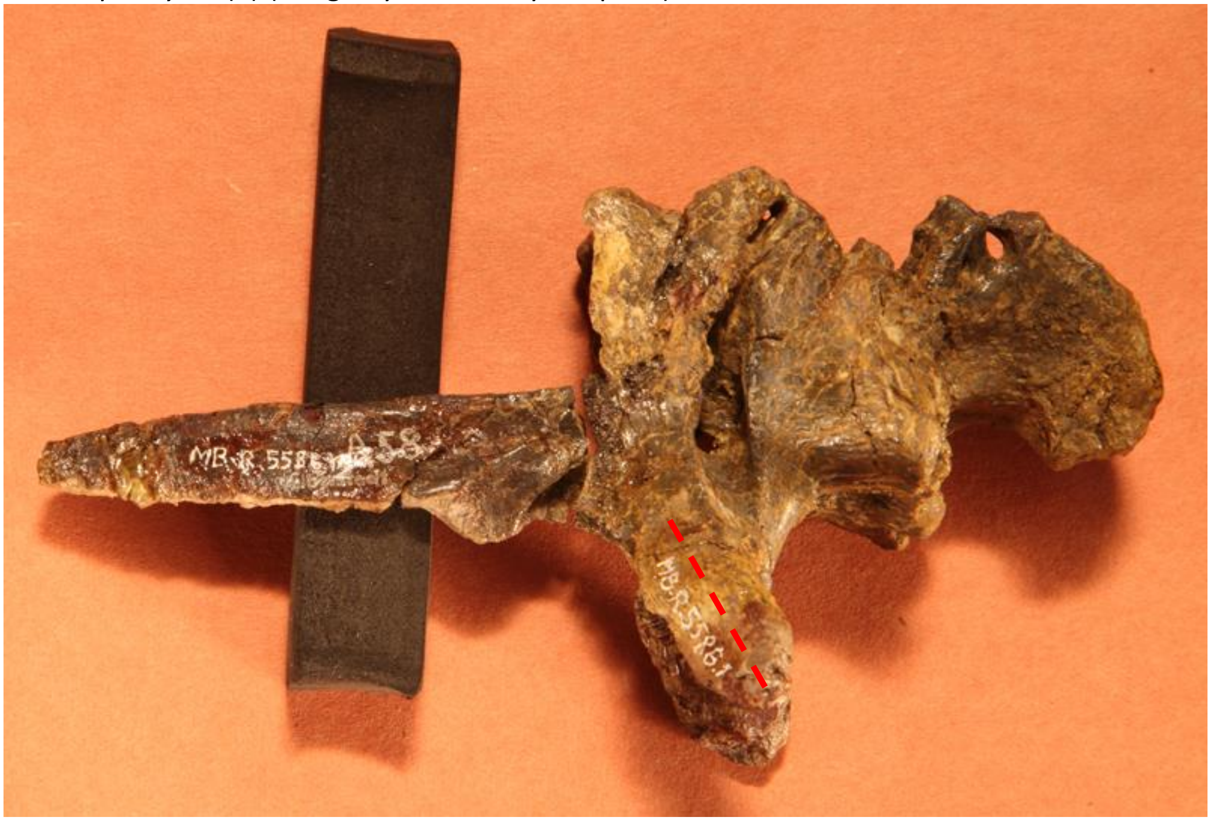

Plateosaurus longiceps (2) (Image by: Kimberley Chapelle)

97. Basisphenoid: Basal tubera ventral margin (New character)

0 basal tubera ventral margins extends as far ventrally as basipterygoid processes ventral margins

1 basipterygoid processes ventral margins extend further ventrally than that of basal tubera

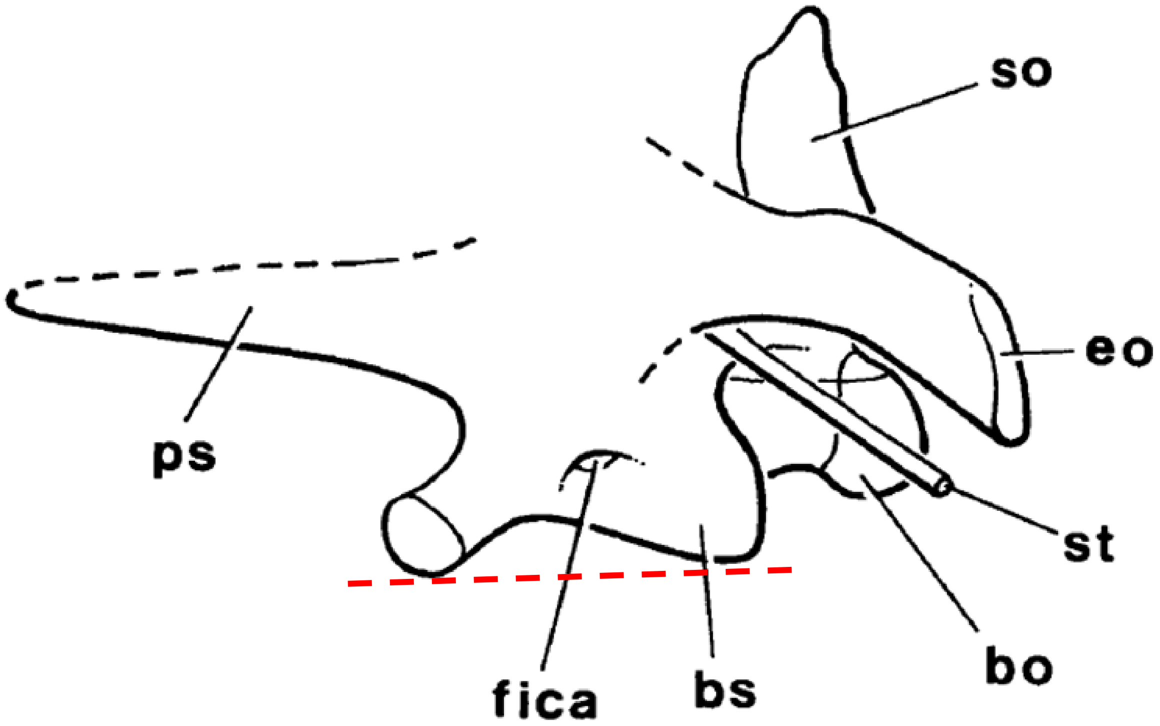

Herrerasaurus (0) (Image from: Sereno & Novas 1993; copyright © Society of Vertebrate Paleontology, www.vertpaleo.org, reprinted by permission of Taylor & Francis Ltd, http://www.tandfonline.com on behalf of Society of Vertebrate Paleontology)

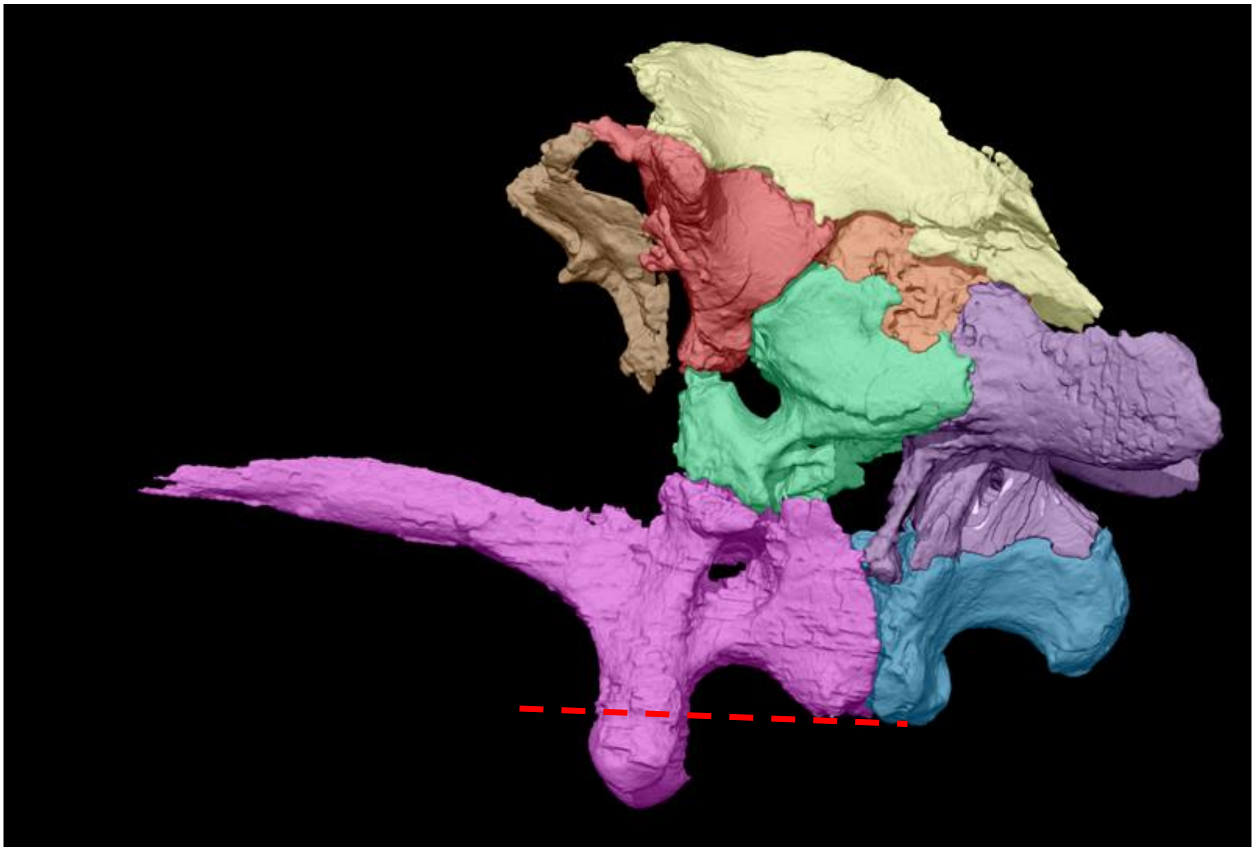

Massospondylus (1) (Image by: Kimberley Chapelle)

98. Basisphenoid: Basal tubera ventral margin (New character)

0 Level or dorsal to proximal base of basipterygoid processes

1 Ventral to proximal base of basipterygoid processes

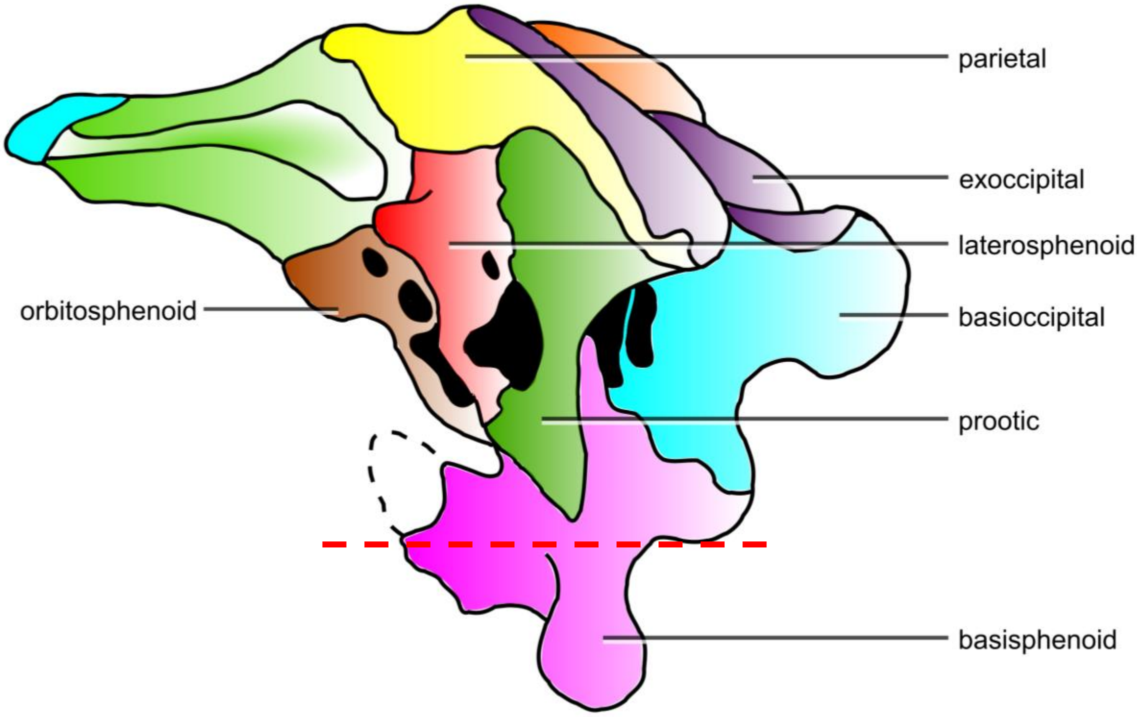

Shunosaurus (0) (Image by: Kimberley Chapelle, modified from Zheng 1996)

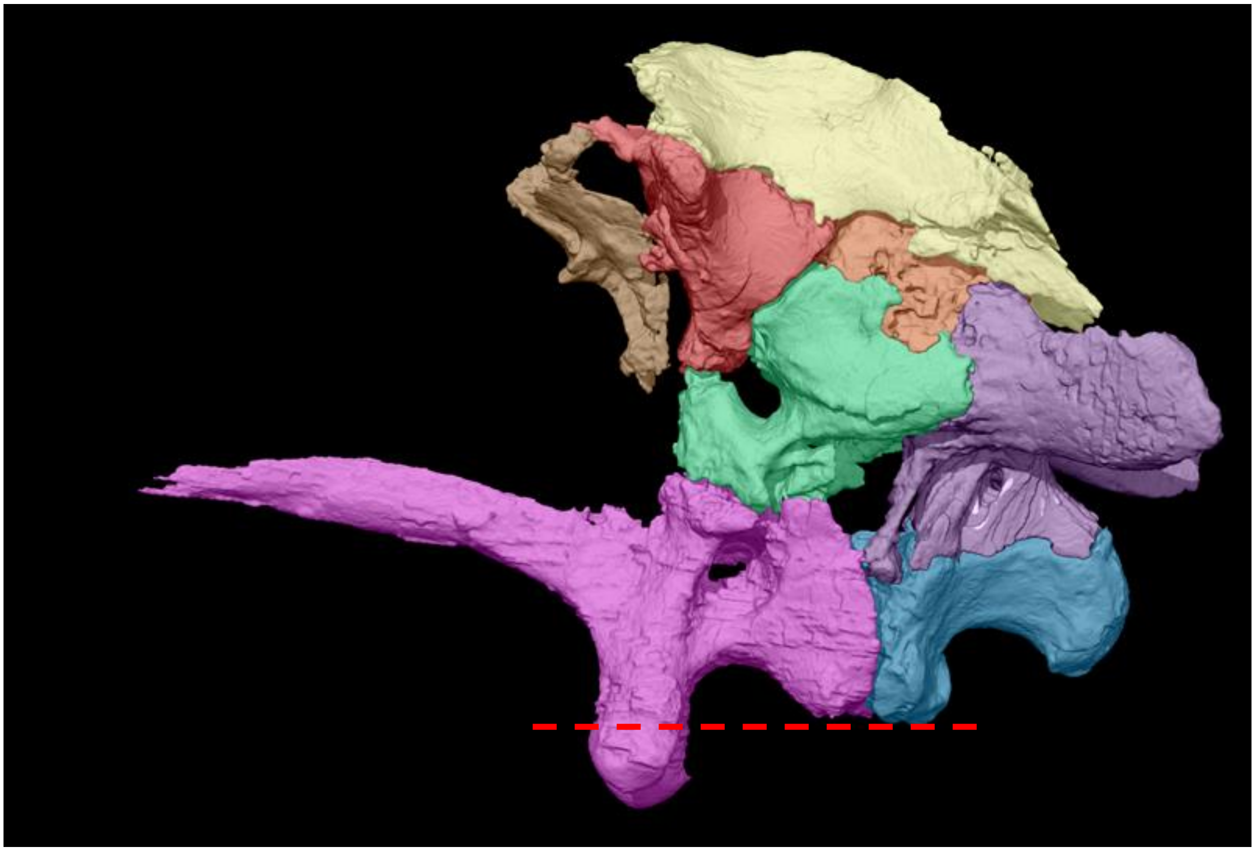

Massospondylus (1) (Image by: Kimberley Chapelle)

99. Basisphenoid: Orientation of long axis of cultriform process (New character)

0 anteroposteriorly oriented, horizontal

1 slopes posteroventrally

2 slopes anteroventrally

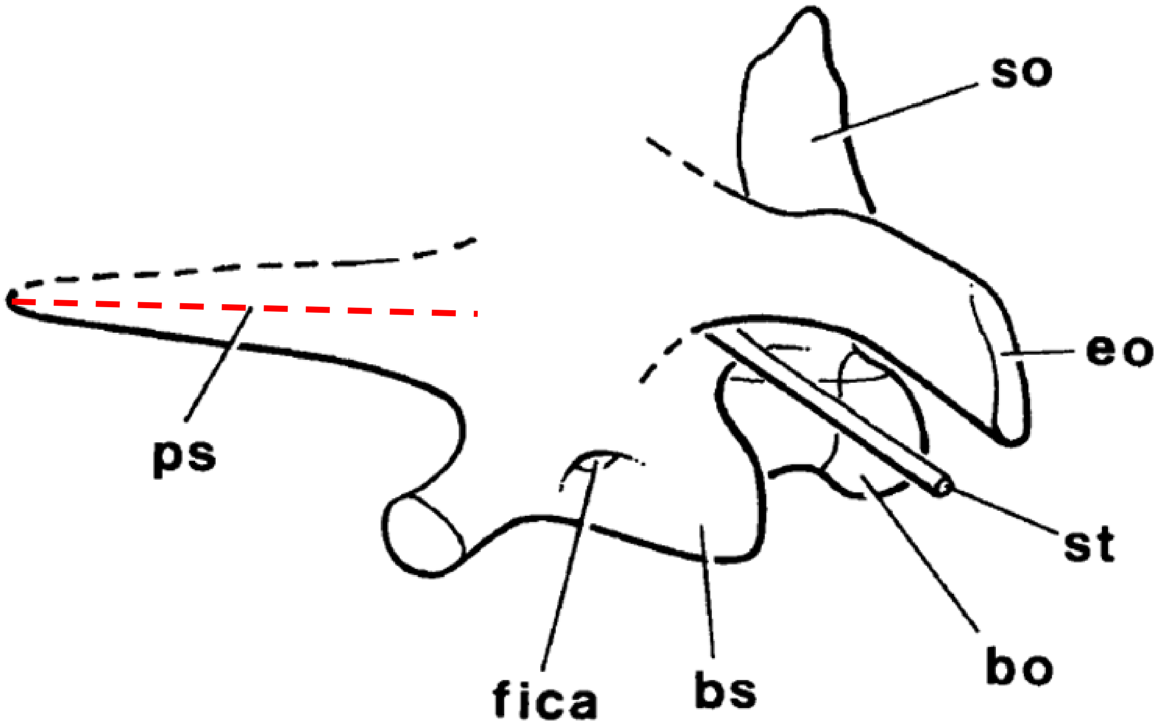

Herrerasaurus (0) (Image from: Sereno & Novas 1993; copyright © Society of Vertebrate Paleontology, www.vertpaleo.org, reprinted by permission of Taylor & Francis Ltd, http://www.tandfonline.com on behalf of Society of Vertebrate Paleontology)

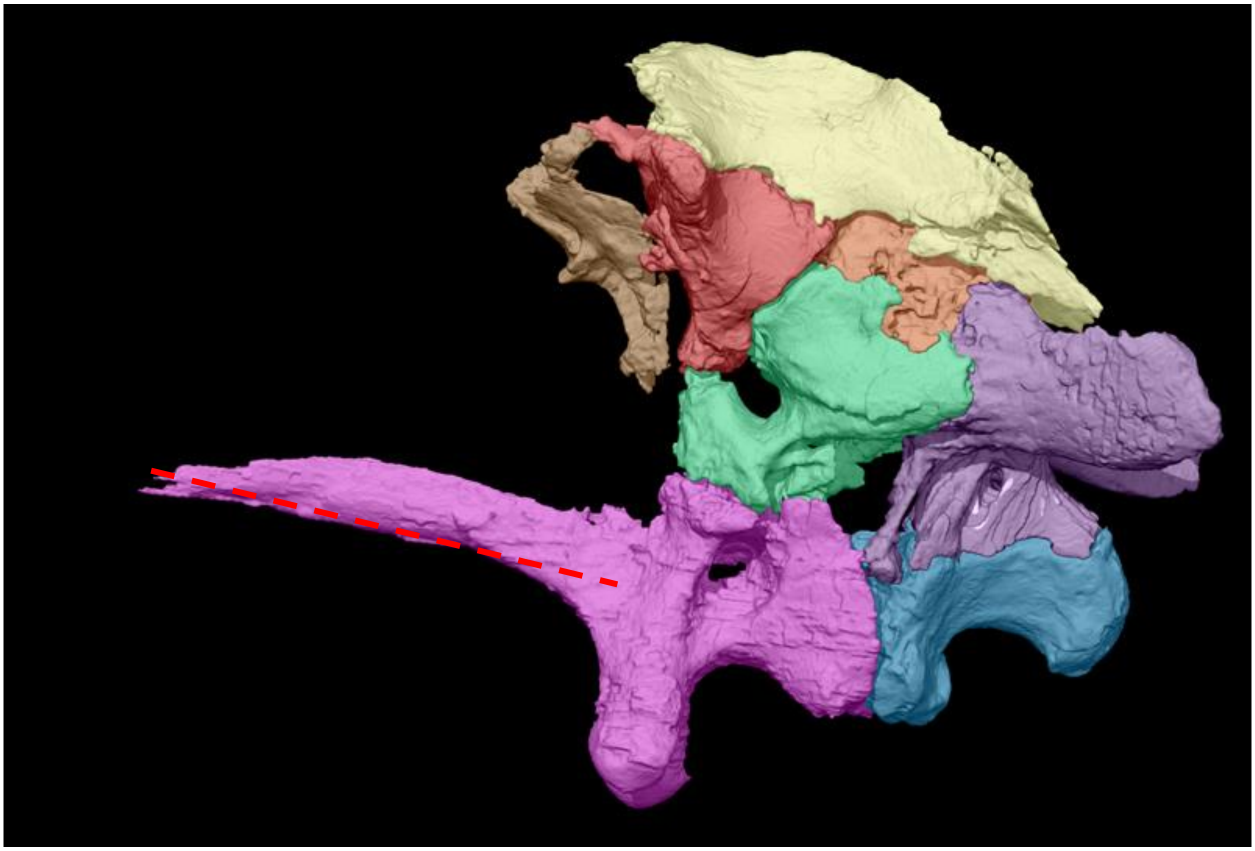

Massospondylus (1) (Image by: Kimberley Chapelle)

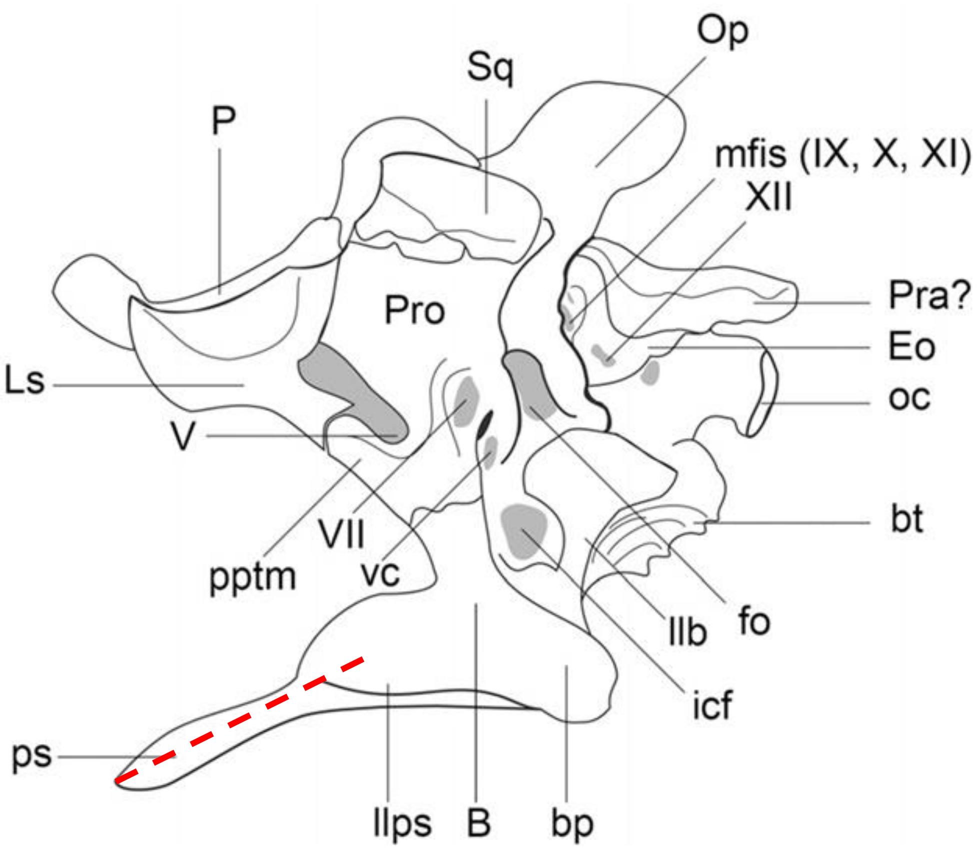

Coloradisaurus (2) (Image from: Apaldetti et al. 2014; copyright © Society of Vertebrate Paleontology, www.vertpaleo.org, reprinted by permission of Taylor & Francis Ltd, http://www.tandfonline.com on behalf of Society of Vertebrate Paleontology)

100. Basisphenoid: Orientation of main body (axis passing through middle of posterior margin of basal tubera and junction between base of basipterygoid process and cultriform process) in lateral view (New character, Chapelle 2017, ch 102)

0 anteroposteriorly oriented, horizontal

1 slopes anteroventrally

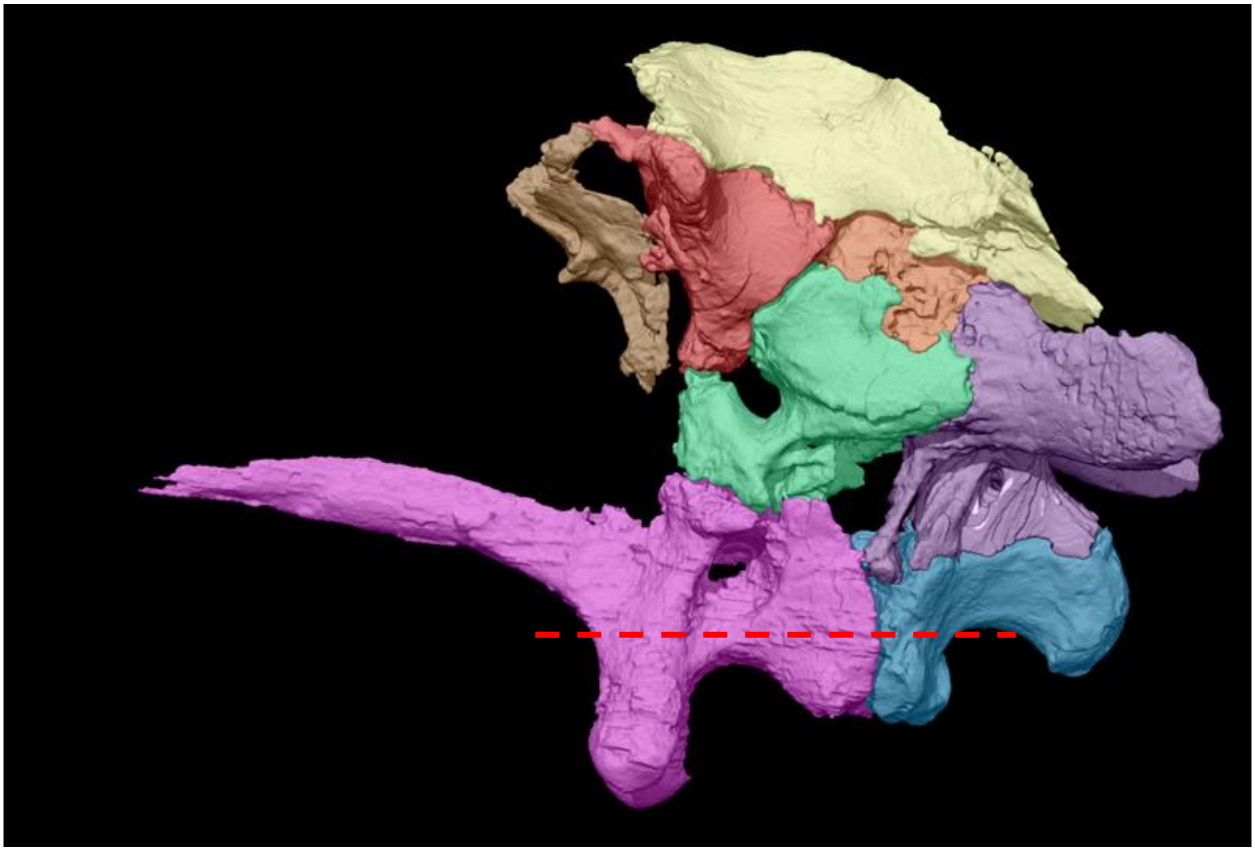

Massospondylus (0) (Image by: Kimberley Chapelle)

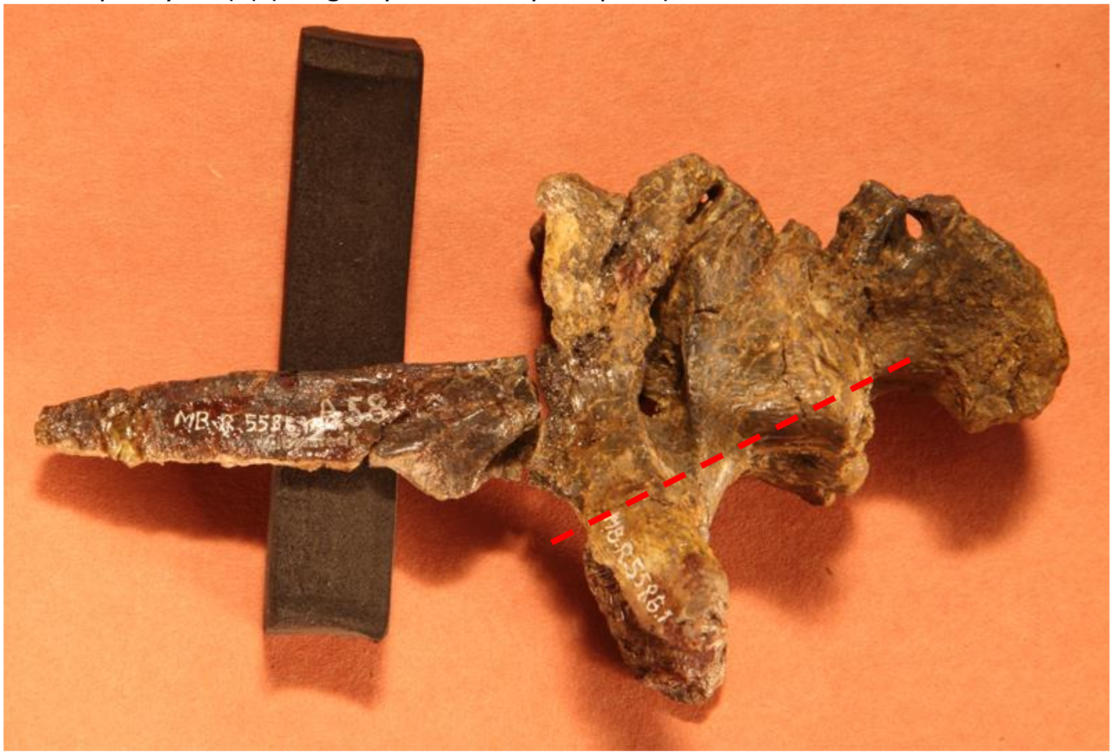

Plateosaurus longiceps (1) (Image by: Kimberley Chapelle)

101. Laterosphenoid: Length of postorbital ramus (New character). The length of the postorbital ramus of the laterosphenoid with respect to that of the supraoccipital ramus of the laterosphenoid varies between taxa like *Massospondylus* and *Plateosaurus erlenbergiensis*.

0 short (subequal to supraoccipital ramus)

1 long (more than 10% longer than supraoccipital ramus)

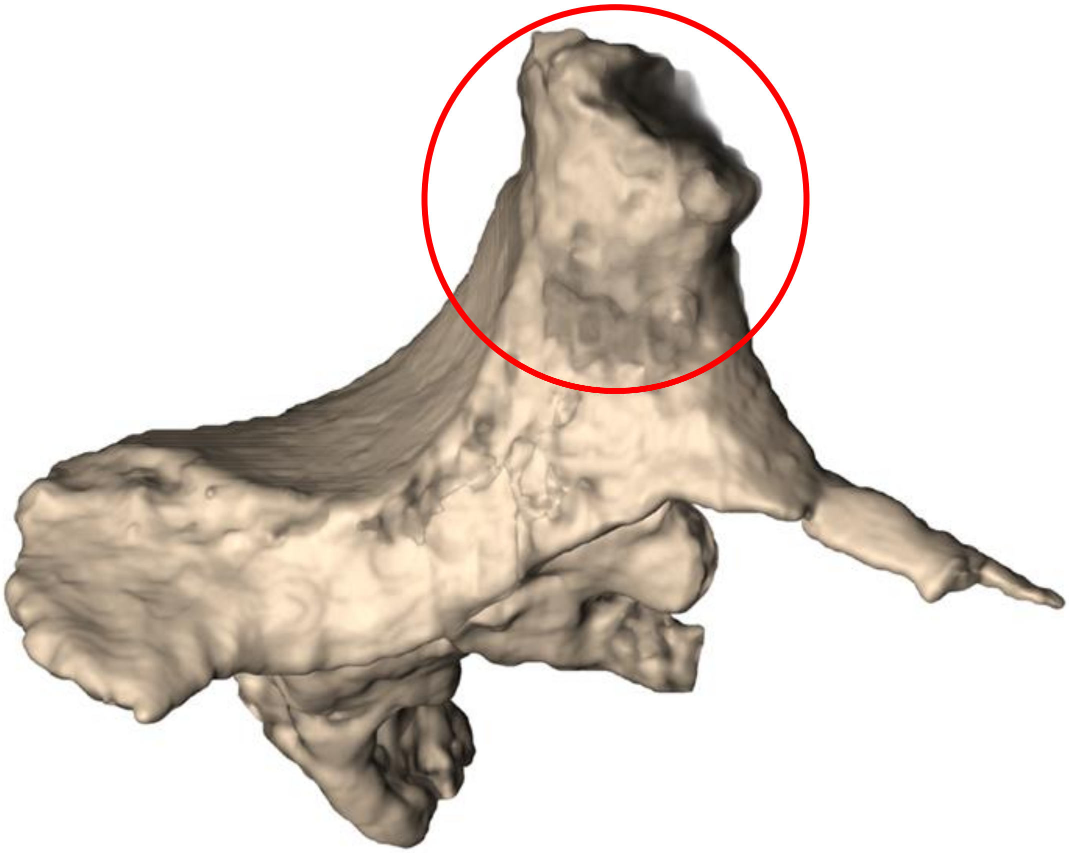

Massospondylus (0) (Image by: Kimberley Chapelle)

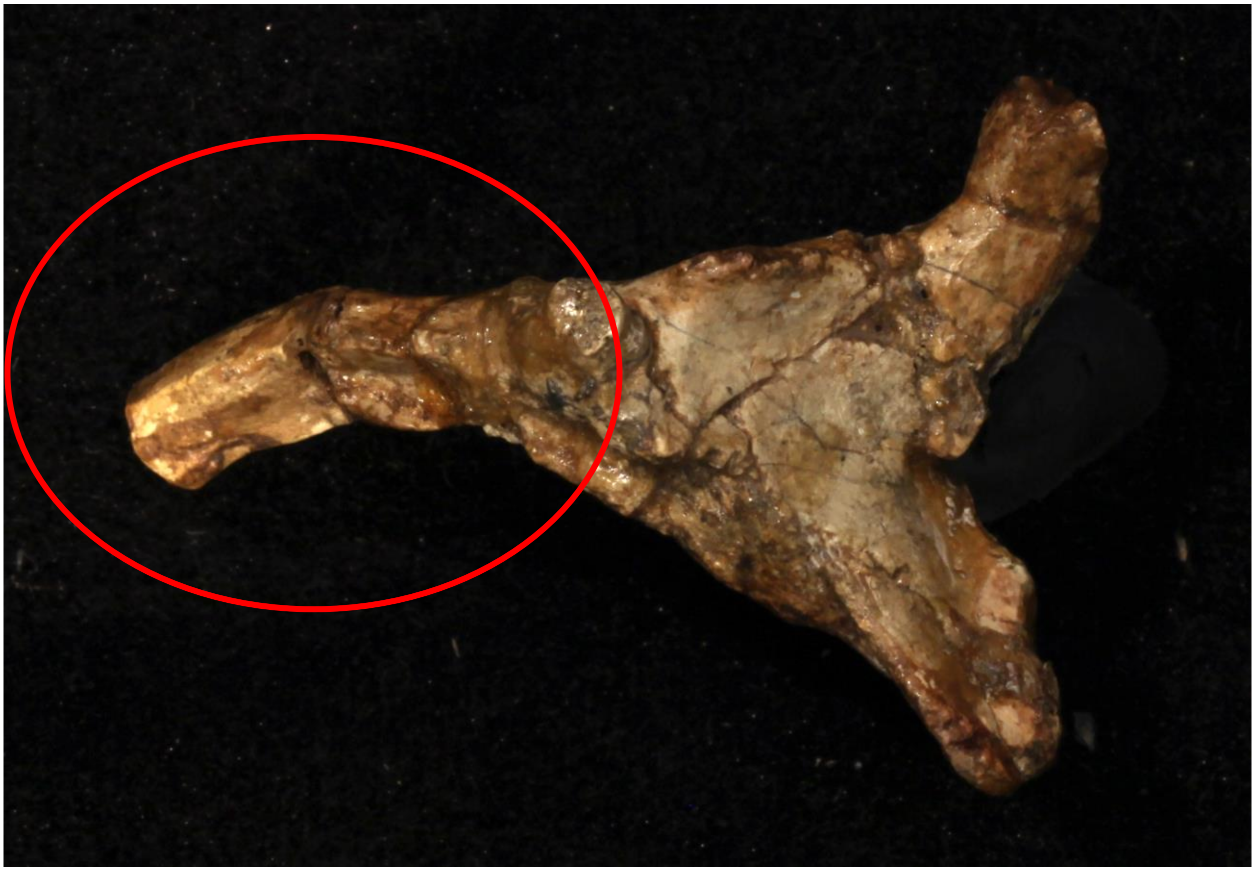

Plateosaurus erlenbergiensis (1)

102. Laterosphenoid: Orientation of postorbital ramus (New character). In some taxa, such as *Massospondylus*, the postorbital ramus of the laterosphenoid extends laterally whereas in *Plateosaurus erlenbergiensis*, it extends anterodorsally.

0 extends laterally
1 extends anterodorsally

103. Laterosphenoid: orientation of frontal ramus (New character). In some taxa, such as *Plateosaurus erlenbergiensis*, the frontal ramus of the laterosphenoid extends medially whereas in *Massospondylus*, it extends anteromedially.

0 extends medially
1 extends anteromedially

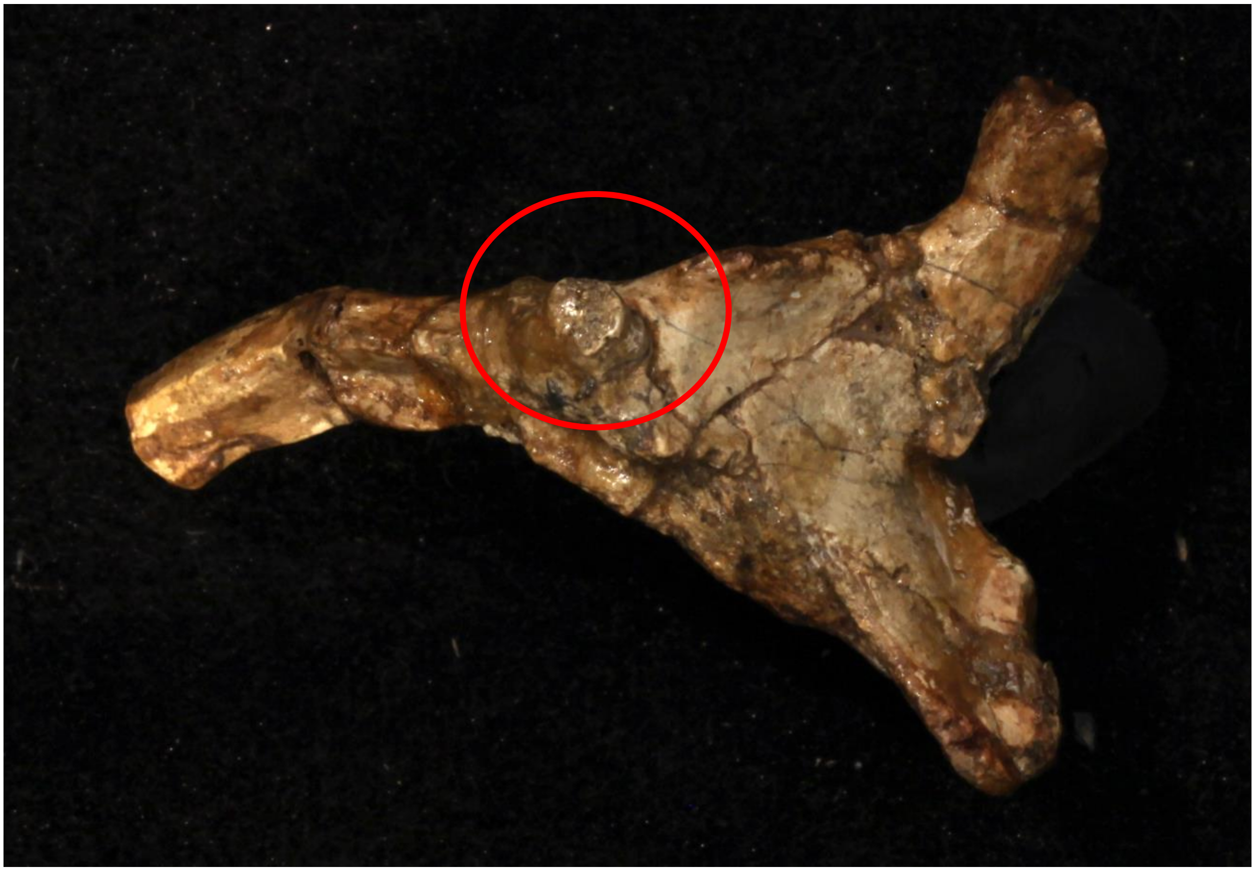

Plateosaurus erlenbergiensis (0) (Image by : Kimberley Chapelle)

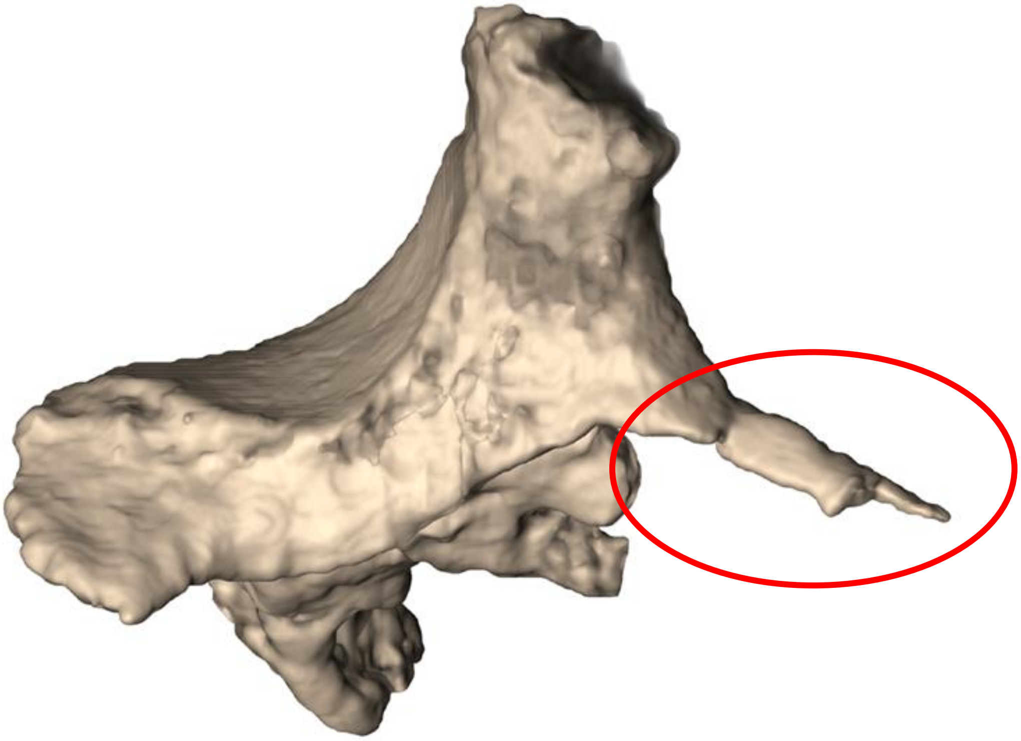

Massospondylus (1) (Image by: Kimberley Chapelle)

The following character was added as part of the break down character 81 in the Yates 2007 matrix ("shape of floor of the braincase") to insure the correct capture of every morphological combinations possible for the floor of the braincase.
104. Basioccipital: ventral margin of basioccipital condyle (New character). In some taxa, such as *Massospondylus*, the ventral margin of the basioccipital is aligned with or ventral to the level of the proximal base of the basipterygoid processes. In other taxa, such as *Coloradisaurus*, the basioccipital ventral margin is dorsal to the level of the proximal base of the basipterygoid processes.

0 aligned with or ventral to proximal base of basipterygoid processes

1 dorsal to proximal base of basipterygoid processes

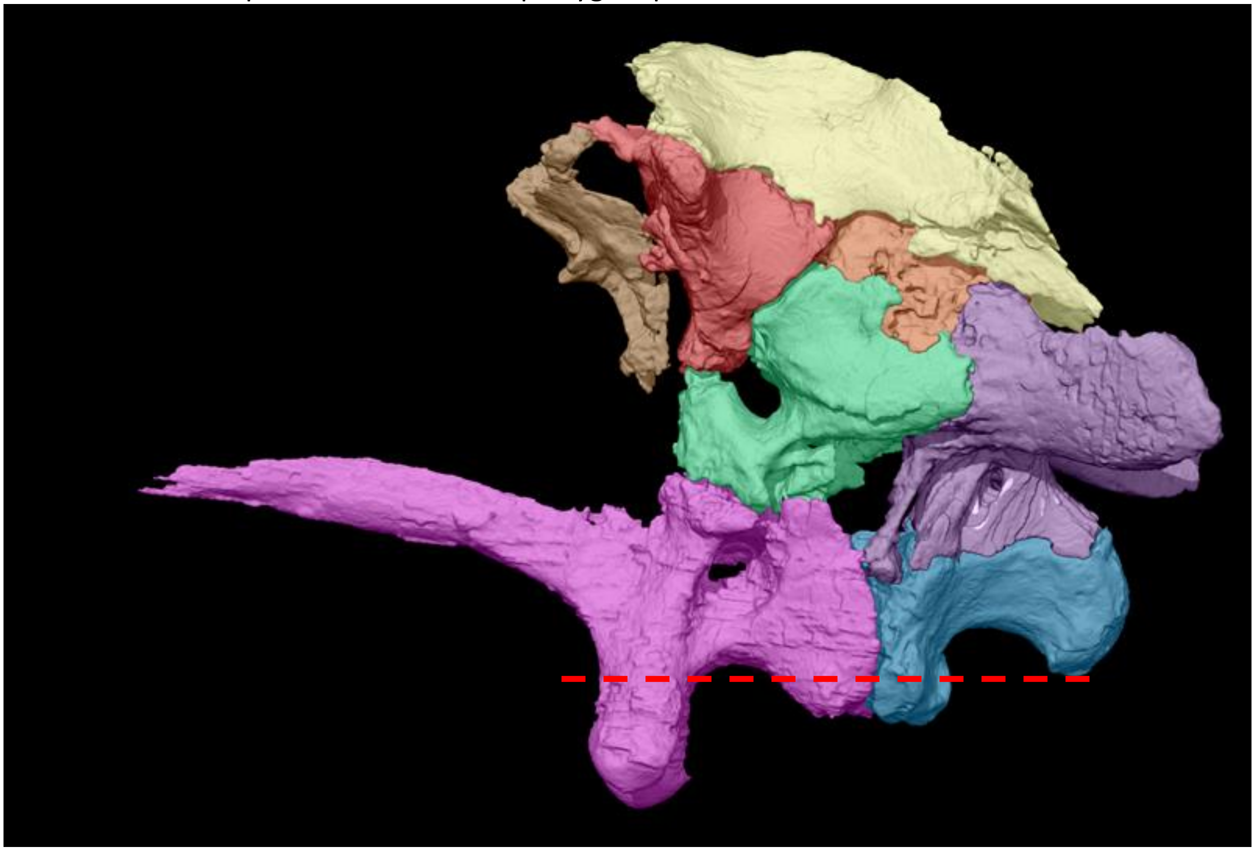

Massospondylus (0) (Image by: Kimberley Chapelle)

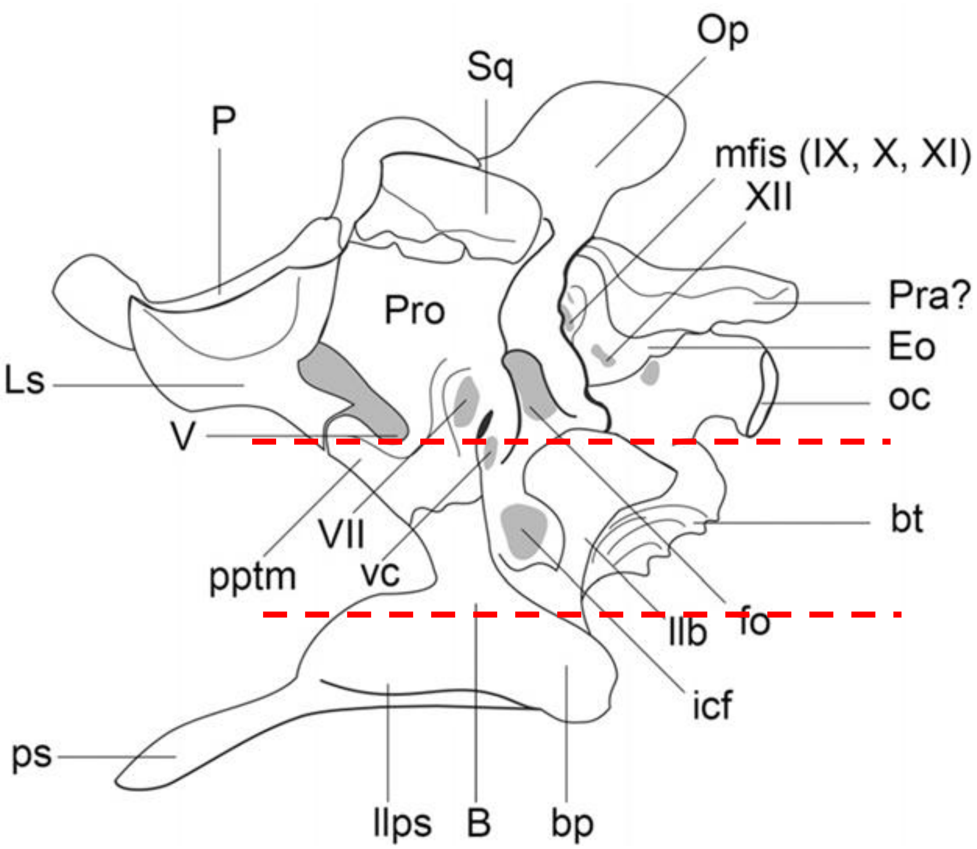

Coloradisaurus (1) (Image from: Apaldetti et al. 2014; copyright © Society of Vertebrate Paleontology, www.vertpaleo.org, reprinted by permission of Taylor & Francis Ltd, http://www.tandfonline.com on behalf of Society of Vertebrate Paleontology)

105. Ectoperygoid: Shape of jugal ramus of ectopterygoid (Yates 2003b; Yates 2007 ch. 87)

 0 gently curved

 1 strongly recurved and hook-like

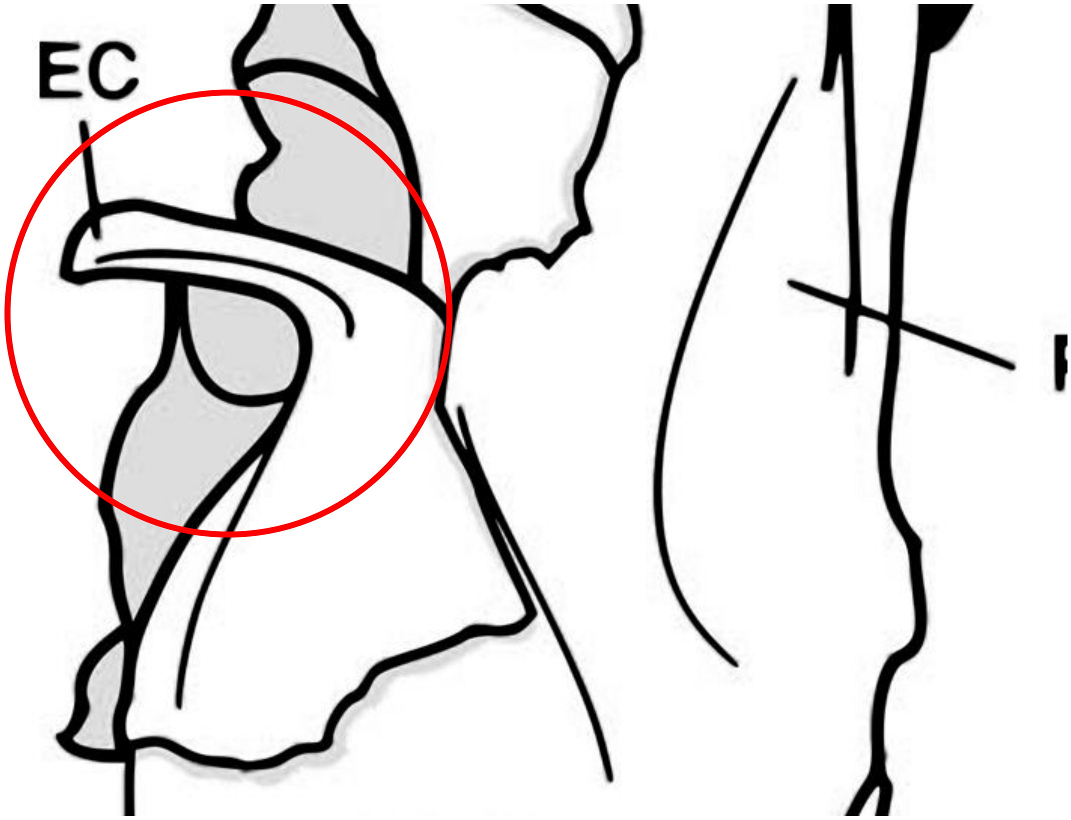

Adeopapposaurus (0) (Image from: Martinez & Alcober 2009)

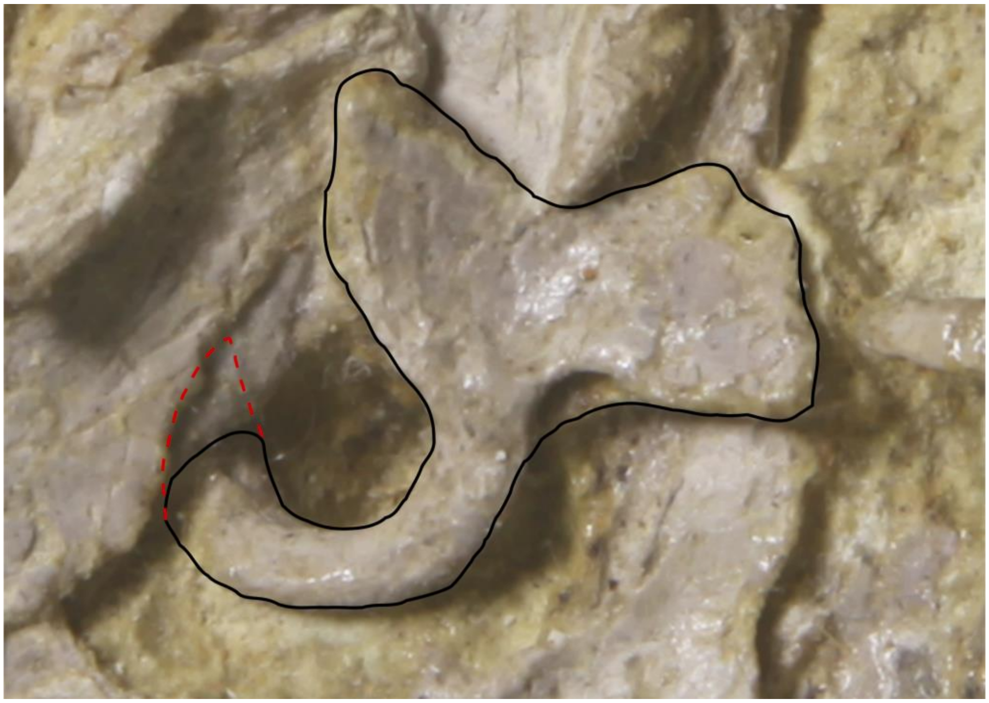

Pantydraco (1) (Image by: Kimberley Chapelle)

106. Ectopterygoid: Distal end of jugal ramus of ectopterygoid (New character). The distal end of the jugal ramus of the ectopterygoid varies between taxa. In *Massospondylus*, this distal end is tapering, whereas in *Herrerasaurus* it is a broad an rectangular distal end and in *Plateosaurus erlenbergiensis*, the distal end of the ramus expands to form a T shape in dorsal and ventral views.

0 tapering

1 non tapering, broad rectangular distal end

2 expands anteroposteriorly, forming a T-shaped dorsal and ventral profile

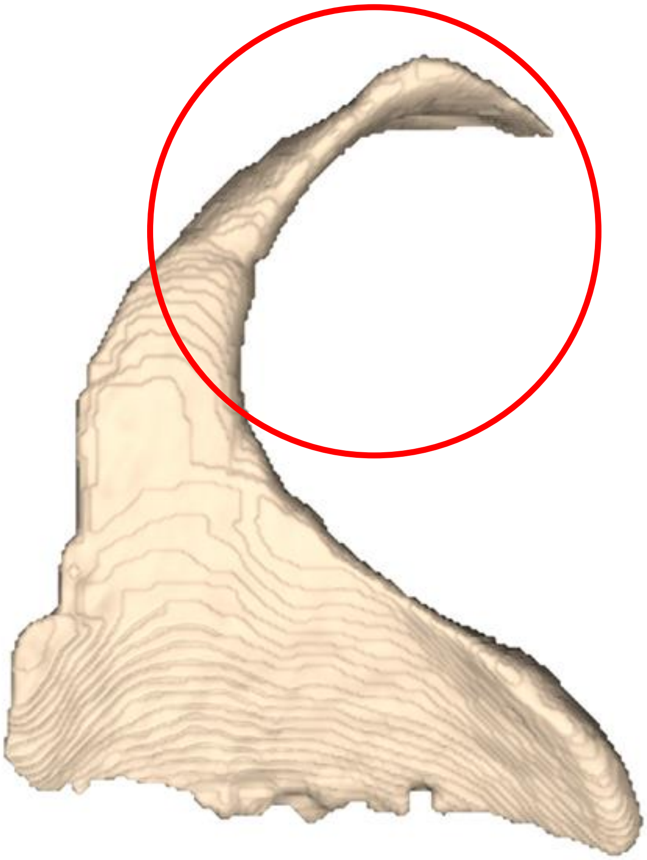

Massospondylus (0) (Image by: Kimberley Chapelle)

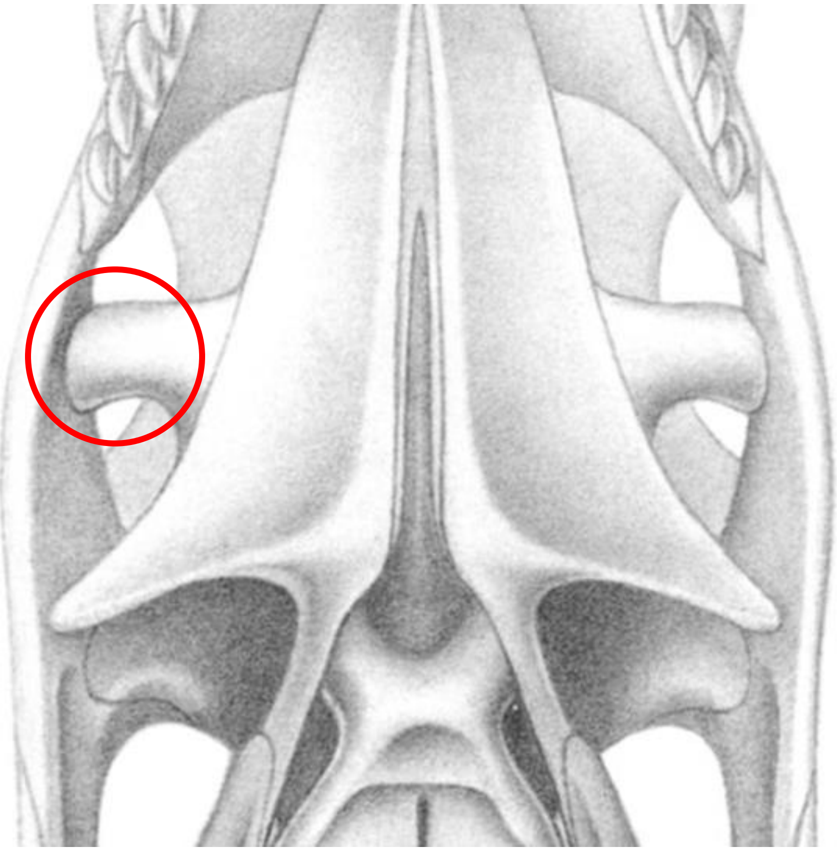

Herrerasaurus (1) (Image from: Sereno & Novas 1993; copyright © Society of Vertebrate Paleontology, www.vertpaleo.org, reprinted by permission of Taylor & Francis Ltd, http://www.tandfonline.com on behalf of Society of Vertebrate Paleontology)

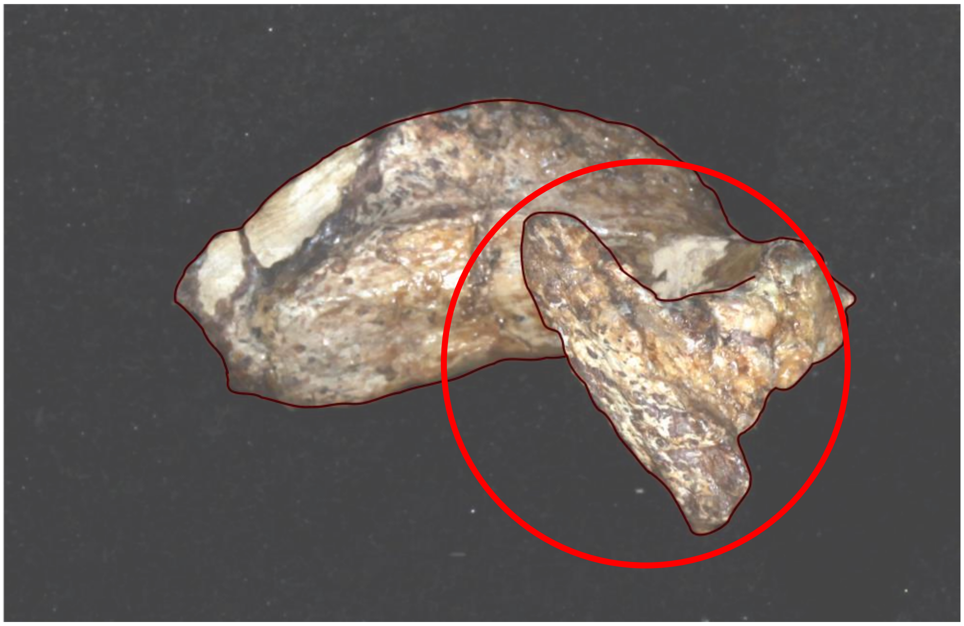

Plateosaurus erlenbergiensis (2) (Image by: Kimberley Chapelle)

107. Ectopterygoid: Pneumatic fossa on the ventral surface of the ectopterygoid (Sereno et al. 1996; Yates 2007 ch. 88)

0 present

1 absent

108. Ectopterygoid: Relationship of the ectopterygoid to the pterygoid (Sereno et al. 1993; Yates 2007 ch. 89)

0 ectopterygoid overlapping the ventral surface of the pterygoid

1 ectopterygoid overlapping the dorsal surface of the pterygoid

109. Palatine: Position of the maxillary articular surface of the palatine (Wilson and Sereno 1998; Yates 2007 ch. 90)

0 along the lateral margin of the bone

1 at the end of a narrow anterolateral process due to the absence of the posterolateral process

110. Palatine: Centrally located tubercle on the ventral surface of palatine (Yates 2007 - ch. 91)

0 absent

1 present

111. Pterygoid: Medial process of the pterygoid forming a hook around the basipterygoid process (New character)

0 absent

1 present

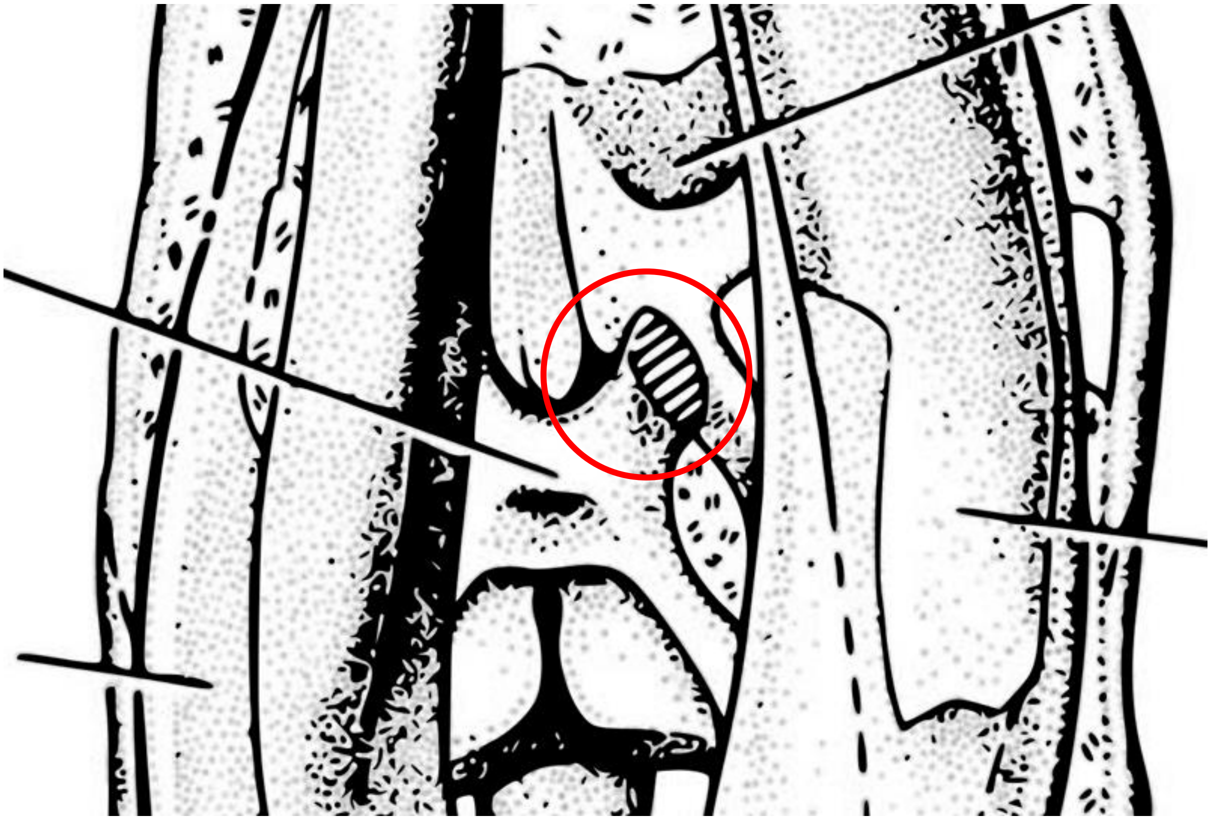

Herrerasaurus (0) (Image from: Sereno & Novas 1993; copyright © Society of Vertebrate Paleontology, www.vertpaleo.org, reprinted by permission of Taylor & Francis Ltd, http://www.tandfonline.com on behalf of Society of Vertebrate Paleontology)

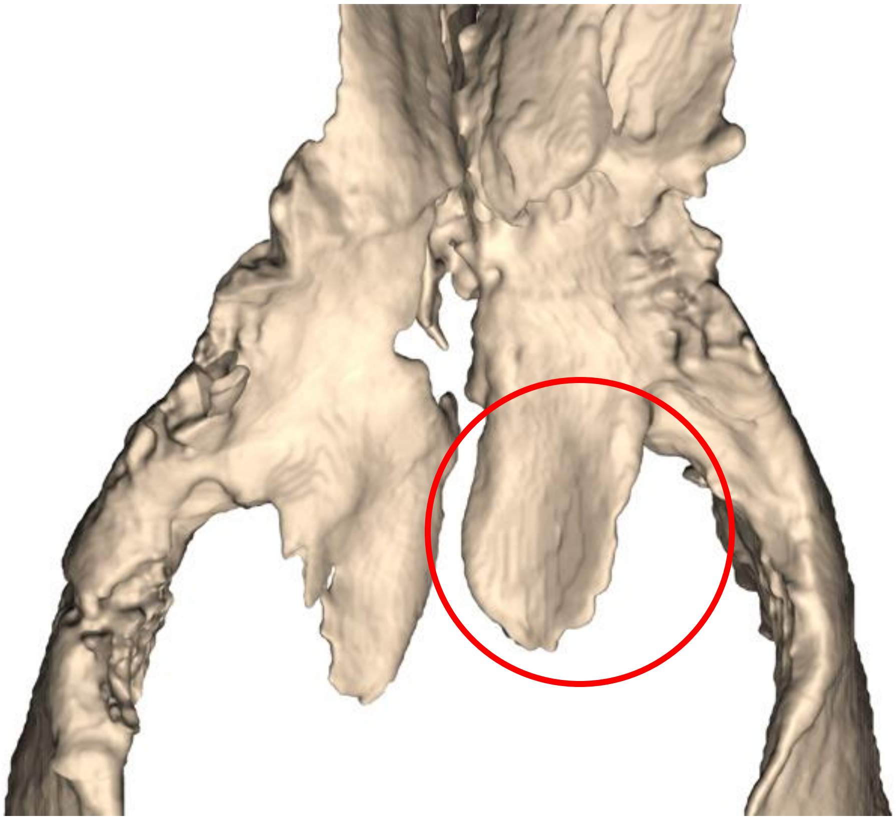

Massospondylus (1) (Image by: Kimberley Chapelle)

112. Pterygoid: Orientation of medial process of the pterygoid forming a hook around the basipterygoid process (modified from Wilson and Sereno 1998. Yates 2007 ch. 92)

  0 horizontally oriented and blunt-ended
  1 rising dorsomedially and tapering
  2 horizontally oriented and tapering

113. Vomer: Length (Yates 2007 ch. 93)

  0 less than 0.25 of the total skull length
  1 more than 0.25 of the total skull length

114. Jaw: Position of jaw joint in lateral view (Sereno 1999; Yates 2007 ch. 94)

  0 no lower than the level of the dorsal margin of the dentary
  1 depressed well below this level

115. Jaw: Shape articulated premaxillae and maxillae in ventral view (Wilson and Sereno 1998; Yates 2007 ch. 95)

  0 narrow with an acute anterior apex
  1 broad and U-shaped

116. Mandibular fenestra: anteroposterior length of the external mandibular fenestra (modified from Upchurch 1995; Yates 2007 ch. 96)

0 more than 0.1 of the length of the mandible
1 less than 0.1 of the length of the mandible

117. Tooth row: Posterior end of dentary tooth row medially inset with a thick lateral ridge on the dentary forming a buccal emargination (Gauthier 1986; Yates 2007 ch. 97)
0 absent
1 present

118. Dentary: Maximum dorsoventral height to anteroposterior length ratio of the dentary (modified from Benton et al. 2000; Yates 2007 ch. 98)
0 less than 0.2
1 greater than 0.2

119. Dentary: Orientation of ventral margin of dentary in lateral view (Sereno 1999; Yates 2007 ch. 99)
0 in line with the long axis of the dentary
1 strongly curved ventrally

120. Dentary: Position of first dentary tooth (Sereno 1999; Yates 2007 ch. 100)
0 adjacent to symphysis
1 inset one tooth's width from the symphysis

121. Dentary: Dorsoventral expansion at the symphyseal end of the (Wilson and Sereno 1998; Yates 2007 ch. 101)
0 absent
1 present

122. Splenial foramen (New character)
0 absent
1 present

123. Splenial foramen (Rauhut 2003. Ordered, Yates 2007 ch. 102)
0 present and enclosed
1 present and open anteriorly

124. Splenial-angular joint (Sereno et al. 1993; Yates 2007 ch. 103)
0 flattened sutured contact
1 synovial joint surface between tongue-like process of angular fitting in groove of the splenial

125. Articular: Stout, triangular, medial process of the articular, behind the glenoid (Yates 2003b; Yates 2007 ch. 104)
0 present
1 absent

126. Retroarticular process: anteroposterior length of the retroarticular process (Yates 2003b; Yates 2007 ch. 105)
    0 less than the depth of the mandible below the glenoid
    1 greater than the depth of the mandible below the glenoid

127. Articular: Strong medial embayment behind glenoid of the articular in dorsal view (Yates and Kitching 2003; Yates 2007 ch. 106)
    0 absent
    1 present

128. Teeth: Number of premaxillary teeth (Galton 1990; Yates 2007 ch. 107)
    0 four
    1 more than four

129. Teeth: Number of dentary teeth (in adults) (modified from Wilson and Sereno 1998; Yates 2007 ch. 108)
    0 less than 18
    1 18 or more

130. Teeth: Arrangement of teeth within the jaws (Yates 2007 ch. 109)
    0 linearly placed, crowns not overlapping
    1 imbricated with distal side of tooth overlapping mesial side of the succeeding tooth

131. Teeth: Orientation of the maxillary tooth crowns (modified from Gauthier 1986; Yates 2007 ch. 110)
    0 erect
    1 procumbent

132. Teeth: Orientation of the dentary tooth crowns (modified from Gauthier 1986; Yates 2007 ch. 111)
    0 erect
    1 procumbent

133. Teeth: Teeth with basally constricted crowns (Gauthier 1986; Yates 2007 ch. 112)
    0 absent
    1 present

134. Tooth: tooth occlusal wear facets (Wilson and Sereno 1998; Yates 2007 ch. 113)
    0 absent
    1 present

135. Teeth: Mesial and distal serrations of the teeth (Benton et al. 2000; Yates 2007 ch. 114)
    0 fine and set at right angles to the margin of the tooth
    1 coarse and angled upwards at an angle of 45 degrees to the margin of the tooth

136. Teeth: Serrations on maxillary and dentary teeth (New character)
 0 absent
 1 present

137. Teeth: Distribution of serrations on the maxillary and dentary teeth (Wilson 2002. Unordered, Yates 2007 ch. 115)
 0 present on both the mesial and distal carinae
 1 absent on the distal carinae

138. Teeth: Long axis of the tooth crowns distally recurved (Gauthier 1986; Yates 2007 ch. 116)
 0 present
 1 absent

139. Teeth: Texture of the enamel surface (modified from Wilson and Sereno 1998, Ordered, Yates 2007 ch. 117)
 0 entirely smooth
 1 finely wrinkled in some patches
 2 extensively and coarsely wrinkled

140. Teeth: Lingual concavities of the teeth (Upchurch 1995; Yates 2007 ch. 118)
 0 absent
 1 present

141. Teeth: Longitudinal labial grooves on the teeth (Upchurch 1998; Yates 2007 ch. 119)
 0 absent
 1 present

142. Teeth: Distribution of the serrations along the mesial and distal carinae of the tooth (Yates 2003b; Yates 2007 ch. 120)
 0 extend along most of the length of the crown
 1 restricted to the upper half of the crown

References:

Apaldetti C, Martinez RN, Pol D, and Souter T. 2014. Redescription of the skull of *Coloradisaurus brevis* (Dinosauria, Sauropodomorpha) from the Late Triassic Los Colorados Formation of the Ischigualasto-Villa Union Basin, northwestern Argentina. *Journal of Vertebrate Paleontology* 34:1113-1132.

Barrett PM, Upchurch P, and Wang X-L. 2005. Cranial osteology of *Lufengosaurus huenei* Young (Dinosauria: Prosauropoda) from the Lower Jurassic of Yunnan, People's Republic of China. *Journal of Vertebrate Paleontology* 25:806-822.

Benton MJ, Juul L, Storrs GW, and Galton PM. 2000. Anatomy and systematics of the prosauropod dinosaur *Thecodontosaurus antiquus* from the Upper Triassic of southwest England. *Journal of Vertebrate Paleontology* 20:77-108.

Ewer RF. 1965. The anatomy of the thecodont reptile *Euparkeria capensis* Broom. *Philosophical Transactions of the Royal Society of London Series B, Biological Sciences* 248:379–435.

Galton PM. 1990. Prosauropoda. In: Weishampel DB, Dodson P, and Osmolska H, eds. *The Dinosauria, second edition*. Berkeley: University of California Press, 320-344

Galton PM, and Bakker RT. 1985. The Cranial Anatomy of the Prosauropod Dinosaur" *Efraasia diagnostica*", a Juvenile Individual of *Sellosaurus Gracilis* from the Upper Triassic of Nordwürttemberg, West Germany. *Staatl Musum f Naturkunde* 117:1-15.

Galton PM, and Upchurch P. 2004. Prosauropoda. In: Dodson P, Osmolska H, and Weishampel DB, eds. *The Dinosauria*. Berkeley: University of California Press, 232-258.

Gauthier J. 1986. Saurischian monophyly and the origin of birds. *Memoirs of the California Academy of Sciences* 8:1–55.

He X, Li K, and Cai K. 1988. *Middle Jurassic Dinosaur Faunas from Dashanpu, Zigong, Sichuan*. Chengdu, China: Sichuan Science and Technology Publishing House.

Holtz TR, Jr. 1994. The phylogenetic position of the tyrannosauridae: implications for theropod systematics. *Journal of Paleontology* 68:1100–1117.

Knoll F, Witmer LM, Ortega F, Ridgely RC, and Schwarz-Wings D. 2012. The braincase of the basal sauropod dinosaur *Spinophorosaurus* and 3D reconstructions of the cranial endocast and inner ear. *PLoS ONE* 7:e30060. 10.1371/journal.pone.0030060

Langer MC. 2004. Basal Saurischia. In: Weishampel DB, Dodson P, and Osmolska H, eds. *The Dinosauria, second edition*. Berkeley: University of California Press, 25-46.

Leal LA, Azevedo SAK, Kellner AWA, and Rosa ÁASD. 2004. A new early dinosaur (Sauropodomorpha) from the Caturrita Formation (Late Triassic), Paraná Basin, Brazil. *Zootaxa* 690:1-24.

Martinez RN, and Alcober OA. 2009. A basal sauropodomorph (Dinosauria: Saurischia) from the Ischigualasto Formation (Triassic, Carnian) and the early evolution of Sauropodomorpha. *PLoS ONE* 4:e4397. 10.1371/journal.pone.0004397

Ouyang H, and Ye Y. 2001. *The first mamenchisaurian skeleton with complete skull: Mamenchisaurus youngi*: Sichuan Science Technology Press.

Rauhut OWM. 2003. The interrelationships and evolution of basal theropod dinosaurs. *Special Papers in Palaeontology* 69:1–213.

Sereno PC. 1999. The evolution of dinosaurs. *Science* 284:2137-2147.

Sereno PC, Duthiel DB, Gado B, Larsson HCE, Lyon GH, Magwene PM, Sidor CA, Varrichio DJ, and Wilson JA. 1996. Predatory dinosaurs from the Sahara and Late Cretaceous faunal differentiation. *Science* 258:1137–1140.

Sereno PC, Forster CA, Rogers RR, and Monetti AM. 1993. Primitive dinosaur skeleton from Argentina and the early evolution of the Dinosauria. *Nature* 361:64-66.

Sereno PC, Martínez RN, and Alcober OA. 2012. Osteology of *Eoraptor lunensis* (Dinosauria, Sauropodomorpha). *Journal of Vertebrate Paleontology*:83-179.

Sereno PC, and Novas FE. 1993. The skull and neck of the basal theropod *Herrerasaurus ischigualastensis*. *Journal of Vertebrate Paleontology* 13:451–456.

Strong EE, and Lipscomb D. 1999. Character coding and inapplicable data. *Cladistics* 15:363–371.

Upchurch P. 1995. The evolutionary history of sauropod dinosaurs. *Philosophical Transactions of the Royal Society of London B: Biological Sciences* 349:365-390.

Upchurch P. 1998. The phylogenetic relationships of sauropod dinosaurs. *Zoological Journal of the Linnean Society* 124:43-103.

Wilson JA. 2002. Sauropod dinosaur phylogeny: critique and cladistic analysis. *Zoological Journal of the Linnean Society* 136:215-275.

Wilson JA, and Sereno PC. 1998. Early evolution and higher-level phylogeny of sauropod dinosaurs. *Journal of Vertebrate Paleontology* 18:1-79.

Yates AM. 2003a. A new species of the primitive dinosaur *Thecodontosaurus* (Saurischia: Sauropodomorpha) and its implications for the systematics of early dinosaurs. *Journal of Systematic Palaeontology* 1:1-42.

Yates AM. 2003b. The species taxonomy of the sauropodomorph dinosaurs from the Löwenstein Formation (Norian, Late Triassic) of Germany. *Palaeontology* 46:317-337.

Yates AM. 2007. The first complete skull of the Triassic dinosaur *Melanorosaurus* Haughton (Sauropodomorpha: Anchisauria). *Evolution and palaeobiology of early sauropodomorph dinosaurs*:9-55.

Yates AM, Bonnan MF, Neveling J, Chinsamy A, and Blackbeard MG. 2010. A new transitional sauropodomorph dinosaur from the Early Jurassic of South Africa and the evolution of sauropod feeding and quadrupedalism. *Proc Biol Sci* 277:787-794. 10.1098/rspb.2009.1440

Zheng Z. 1996. Cranial anatomy of *Shunosaurus* and *Camarasaurus* (Dinosauria: Sauropoda) and the phylogeny of the Sauropoda. Texas Tech University.
